# Supplementary material for: Longitudinal study of patients’ health-related quality of life using EQ-5D-3L in 11 Swedish National Quality Registers
Source: BMJ Open. 2022 Jan 6;12(1):e048176. doi: 10.1136/bmjopen-2020-048176 (PMC8739074; doi:10.1136/bmjopen-2020-048176)
Supplement: Supplementary data [file bmjopen-2020-048176supp001.pdf]

Longitudinal study of patients' health-related quality of life using EQ-5D-3L in 11 Swedish National Quality Registers

Fitsum Sebsibe Teni[1*], Ola Rolfson[1,2,3], Nancy Devlin[4,5], David Parkin[5,6], Emma Nauclér[3], Kristina Burström[1,7,8], The Swedish Quality Register (SWEQR) Study Group

Authors' affiliations

[1] Health Outcomes and Economic Evaluation Research Group, Stockholm Centre for Healthcare Ethics, Department of Learning, Informatics, Management and Ethics, Karolinska Institutet, Stockholm, Sweden.

[2] Department of Orthopaedics, Institute of Clinical Sciences, Sahlgrenska Academy, University of Gothenburg, Gothenburg, Sweden.

[3] Swedish Hip Arthroplasty Register, Gothenburg, Sweden.

[4] Centre for Health Policy, University of Melbourne, Melbourne, Australia.

[5] Office of Health Economics, London, United Kingdom.

[6] City University of London, London, United Kingdom.

[7] Equity and Health Policy Research Group, Department of Global Public Health, Karolinska Institutet, Stockholm; Sweden.

[8] Health Care Services, Region Stockholm, Stockholm, Sweden

*Corresponding author
Email: fitsum.teni@ki.se

Table S1: Sampling procedure followed in combining the data sets from each of the 11 National Quality Registers (NQRs), baseline to 1-year follow-up and the general population data

Category	Patient groups in NQRs	A	B	C	D	E
		n (%)	n (%)	n (%)	n (%)	n (%)
Intervention-based registers	Spine	53,418 (15.8)	53,015 (15.9)	51,047 (15.7)	48,960 (15.5)	44,196 (17.6)
	Hip	95,887 (28.3)	93,684 (28.0)	90,669 (27.9)	90,660 (28.7)	90,658 (36.1)
	Knee	17,600 (5.2)	16,324 (4.9)	16,324 (5.0)	16,324 (5.2)	16,324 (6.5)
	Ankle	794 (0.2)	789 (0.2)	725 (0.2)	700 (0.2)	668 (0.3)
	Cruciate ligament	10,532 (3.1)	10,532 (3.2)	10,465 (3.2)	8,430 (2.7)	8,155 (3.2)
	Osteoarthritis (BOA)	15,238 (4.5)	14,877 (4.5)	13,965 (4.3)	13,647 (4.3)	6,690 (2.7)
	Fracture	55,459 (16.4)	55,428 (16.6)	54,108 (16.6)	50,892 (16.1)	40,787 (16.2)
Diagnosis-based registers	Heart failure	1,789 (0.5)	1,746 (0.5)	1,691 (0.5)	1,436 (0.5)	1,044 (0.4)
	Respiratory failure	1,287 (0.4)	1,063 (0.3)	1,050 (0.3)	1,050 (0.3)	725 (0.3)
	Psoriasis	3,041 (0.9)	2,878 (0.9)	2,789 (0.9)	2,680 (0.8)	-
	Rheumatology	34,036 (10.1)	33,686 (10.1)	32,565 (10.0)	31,462 (10.0)	-
NQRs (total, count)		289,081 (85.4)	284,022 (85.0)	275,398 (84.6)	266,241 (84.4	209,247 (83.4)
General population data		49,499 (14.6)	49,999 (15.0)	49,999 (15.4)	49,169 (15.6	41,761 (16.6)
Total		338,580 (100.0)	334,021 (100.0)	325,397 (100.0)	315,410 (100.0)	251,008 (100.0)

A= Data on baseline and 1-year follow-up
B= After selection of data with information on diagnosis and intervention
C= After de-duplication of records occurring in more than one patient group (duplicate records taken out keeping one record)
D= Included in the analyses after exclusion of missing values on age at baseline, or on any of the EQ-5D dimensions
E= Included in the analyses of EQ VAS values
BOA=Better management of osteoarthritis register, referred to as BOA in whole manuscript
Proportions rounded off to one decimal using MS Excel

Table S2: Demographic characteristics of patients across diagnoses in the 11 National Quality Registers (NQRs)

Patient group in NQRs		Diagnosis	Age (years) Mean (SD)	Age group							Sex
				<30	30-39	40-49	50-59	60-69	70-79	80+	Women
				n (%)	n (%)	n (%)	n (%)	n (%)	n (%)	n (%)	n (%)
Intervention-based registers	**Spine**	Disc hernia (n=12,582)	46.1 (13.5)	1,216 (9.7)	3,089 (24.6)	3,708 (29.5)	2,312 (18.4)	1,519 (12.1)	624 (5.0)	114 (0.9)	5,784 (46.0)
		Other spinal conditions (n=1,431)	58.2 (13.1)	32 (2.2)	98 (6.8)	207 (14.5)	382 (26.7)	411 (28.7)	260 (18.2)	41 (2.9)	891 (62.3)
		Segmental instability (n=4,290)	45.6 (10.2)	245 (5.7)	932 (21.7)	1,674 (39.0)	1,034 (24.1)	345 (8.0)	59 (1.4)	1 (0.0)	2,326 (54.2)
		Spinal stenosis (n=28,194)	67.2 (10.5)	56 (0.2)	245 (0.9)	1,390 (4.9)	4,518 (16.0)	9,101 (32.3)	9,914 (35.2)	2,970 (10.5)	15,046 (53.4)
		Spondylolysis (n=2,463)	50.9 (12.5)	125 (5.1)	288 (11.7)	716 (29.1)	718 (29.2)	459 (18.6)	142 (5.8)	15 (0.6)	1,281 (52.0)
	**Hip**	Avascular necrosis (n=1,594)	66.0 (13.6)	13 (0.8)	68 (4.3)	127 (8.0)	228 (14.3)	444 (27.9)	458 (28.7)	256 (16.1)	935 (58.7)
		Childhood hip disorder (n=1,846)	54.5 (12.4)	23 (1.2)	169 (9.2)	492 (26.7)	533 (28.9)	390 (21.1)	199 (10.8)	40 (2.2)	1,139 (61.7)
		Hip osteoarthritis (n=86,023)	68.9 (9.9)	61 (0.1)	348 (0.4)	2,706 (3.1)	10,814 (12.6)	29,020 (33.7)	30,868 (35.9)	12,206 (14.2)	48,329 (56.2)
		Inflammatory joint disorder (n=1,197)	64.5 (12.5)	25 (2.1)	40 (3.3)	69 (5.8)	191 (16.0)	431 (36.0)	344 (28.7)	97 (8.1)	860 (71.8)
	**Knee**	Knee osteoarthritis (n=16,020)	69.0 (8.7)	0 (0.0)	9 (0.1)	262 (1.6)	2085 (13.0)	5,695 (35.5)	6,203 (38.7)	1,766 (11.0)	9,173 (57.3)
		Knee rheumatoid arthritis (n=304)	66.2 (10.2)	1 (0.3)	1 (0.3)	16 (5.3)	51 (16.8)	119 (39.1)	93 (30.6)	23 (7.6)	219 (72.0)
	**Ankle**	Others (n=85)	57.7 (12.6)	1 (1.2)	7 (8.2)	14 (16.5)	22 (25.9)	26 (30.6)	14 (16.5)	1 (1.2)	31 (36.5)
		Posttraumatic OA (n=308)	62.0 (12.1)	7 (2.3)	10 (3.2)	24 (7.8)	76 (24.7)	98 (31.8)	83 (26.9)	10 (3.2)	134 (43.5)
		Primary OA (n=234)	67.1 (8.9)	0 (0.0)	1 (0.4)	6 (2.6)	37 (15.8)	85 (36.3)	92 (39.3)	13 (5.6)	86 (36.8)
		Rheumatoid arthritis (n=73)	62.1 (13.4)	2 (2.7)	3 (4.1)	8 (11.0)	10 (13.7)	24 (32.9)	26 (35.6)	0 (0.0)	60 (82.2)
	**Cruciate ligament**	Cruciate ligament injury (n=8,430)	29.8 (10.0)	4,964 (58.9)	1,797 (21.3)	1,299 (15.4)	347 (4.1)	22 (0.3)	1 (0.0)	0 (0.0)	3,772 (44.7)
	**BOA**	BOA hip (n=3,440)	65.9 (9.2)	1 (0.0)	27 (0.8)	158 (4.6)	552 (16.0)	1,484 (43.1)	1,043 (30.3)	175 (5.1)	2474 (71.9)
		BOA knee (n=10.207)	65.4 (9.1)	5 (0.0)	49 (0.5)	466 (4.6)	1,985 (19.4)	4,365 (42.8)	2,793 (27.4)	544 (5.3)	7370 (72.2)
	**Fracture**	Ankle and foot (n=3,728)	51.7 (17.4)	530 (14.3)	424 (11.4)	605 (16.3)	819 (22.0)	738 (19.8)	439 (11.8)	163 (4.4)	2,208 (59.4)
		Elbow and forearm (n=13,996)	61.6 (15.9)	794 (5.7)	708 (5.1)	1,189 (8.5)	2,678 (19.1)	4,071 (29.1)	2,999 (21.4)	1,557 (11.1)	10942 (78.2)
		Hip and femur (n=8,378)	77.2 (12.0)	51 (0.6)	58 (0.7)	116 (1.4)	377 (4.5)	1,233 (14.7)	2,442 (29.1)	4,101 (48.9)	5655 (67.5)
		Knee and lower leg (n=10,866)	57.4 (17.0)	960 (8.8)	804 (7.4)	1,377 (12.7)	2,180 (20.1)	2,774 (25.5)	1,962 (18.1)	809 (7.4)	6714 (61.8)
		Lumbar, spine and pelvis (n=1,128)	72.2 (16.8)	36 (3.2)	31 (2.7)	44 (3.9)	111 (9.8)	153 (13.6)	299 (26.5)	454 (40.2)	780 (69.1)
		Shoulder and upper arm (n=7,835)	63.6 (16.6)	464 (5.9)	317 (4.0)	621 (7.9)	1,195 (15.3)	2,064 (26.3)	1,946 (24.8)	1,228 (15.7)	5,291(67.5)
		Unspecified part of trunk, extremities or other body region (n=749)	61.3 (19.2)	76 (10.1)	43 (5.7)	74 (9.9)	106 (14.2)	142 (19.0)	189 (25.2)	119 (15.9)	327 (43.7)
		Wrist and hand (n=4,222)	49.9 (19.2)	882 (20.9)	485 (11.5)	625 (14.8)	750 (17.8)	743 (17.6)	523 (12.4)	214 (5.1)	1,864 (44.1)

**Diagnosis-based registers**	**Heart failure**	Heart failure (n=1,436)	72.8 (11.1)	2 (0.5)	7 (2.6)	37 (9.5)	137 (20.5)	294 (35.7)	512 (31.1)	447 (0.5)	507 (35.3)
	**Respiratory failure**	COPD (n=900)	73.3 (7.6)	0 (0.0)	0 (0.0)	1 (0.1)	39 (4.3)	237 (26.3)	425 (47.2)	198 (22.0)	555 (61.7)
		Lung fibrosis (n=150)	75.9 (7.7)	0 (0.7)	1 (1.3)	(0.0)	2 (18.7)	28 (46.0)	69 (33.3)	50 (0.7)	68 (45.3)
	**Psoriasis**	Psoriasis vulgaris (n=1,704)	49.4 (15.2)	191 (11.2)	273 (16.0)	370 (21.7)	374 (21.9)	346 (20.3)	123 (7.2)	27 (1.6)	576 (33.8)
		Psoriasis vulgaris + anthropathic (n=537)	51.8 (13.2)	21 (3.9)	82 (15.3)	131 (24.4)	132 (24.6)	127 (23.6)	37 (6.9)	7 (1.3)	237 (44.1)
		Psoriasis unspecified (n=439)	51.6 (14.9)	39 (8.9)	67 (15.3)	80 (18.2)	110 (25.1)	95 (21.6)	41 (9.3)	7 (1.6)	181 (41.2)
	**Rheumatol-ogy**	Rheumatoid arthritis (n=17,870)	59.2 (13.9)	585 (3.3)	1,233 (6.9)	2,362 (13.2)	3,846 (21.5)	5,604 (31.4)	3,430 (19.2)	810 (4.5)	12,954 (72.5)
		Spondyloarthropathy (n=8,782)	47.6 (13.9)	1,009 (11.5)	1,596 (18.2)	2,228 (25.4)	2,058 (23.4)	1,380 (15.7)	448 (5.1)	63 (0.7)	4,271 (48.6)
		Inflammatory systemic disorder (n=1,614)	51.9 (15.8)	155 (9.6)	243 (15.1)	295 (18.3)	337 (20.9)	349 (21.6)	206 (12.8)	29 (1.8)	1,249 (77.4)
		Juvenile rheumatoid diseases (n=1,088)	29.7 (12.8)	676 (62.1)	186 (17.1)	134 (12.3)	43 (4.5)	33 (3.0)	9 (0.8)	1 (0.1)	865 (79.5)
		Vasculitis (n=410)	60.0 (14.0)	22 (5.4)	18 (4.4)	40 (9.8)	66 (16.1)	149 (36.3)	105 (25.6)	10 (2.4)	234 (57.1)
		Other arthritis (n=1,698)	54.0 (15.9)	146 (8.6)	207 (12.2)	294 (17.3)	365 (21.5)	378 (22.3)	250 (14.7)	58 (3.4)	1,095 (64.5)

*Percent based on non-missing values;
Proportions rounded off to one decimal using MS Excel

Table S3: Prevalence of reported problems on EQ-5D-3L by diagnosis, at baseline and 1-year follow-up in the 11 National Quality Registers (NQRs) and in the general population

	Patient group in NQRs	Diagnosis	Severity level	Baseline EQ-5D-3L dimension					1-year follow-up EQ-5D-3L dimension				
				Mobility	Self-care	Usual activities	Pain/ discomfort	Anxiety/ depression	Mobility	Self-care	Usual activities	Pain/ discomfort	Anxiety/ Depression
				n (%)	n (%)	n (%)	n (%)	n (%)	n (%)	n (%)	n (%)	n (%)	n (%)
Intervention-based registers	Spine	Disc hernia (n=12,582)	Level 1	1,996 (15.9)	8,535 (**67.8**)	2,353 (18.7)	89 (0.7)	4,603 (36.6)	9,258 (**73.6**)	11,659 (**92.7**)	9,015 (**71.6**)	3,596 (28.6)	8,144 (**64.7**)
			Level 2	9,931 (**78.9**)	3,788 (30.1)	6,373 (**50.7**)	5,470 (43.5)	6,954 (**55.3**)	3,293 (26.2)	864 (6.9)	3,154 (25.1)	7,799 (**62.0**)	3,903 (31.0)
			Level 3	655 (5.2)	259 (2.1)	3,856 (30.6)	7,023 (**55.8**)	1,025 (8.1)	31 (0.2)	59 (0.5)	413 (3.3)	1,187 (9.4)	535 (4.3)
		Other spinal conditions (n=1,431)	Level 1	279 (19.5)	1,144 (**79.9**)	414 (28.9)	14 (1.0)	586 (41.0)	813 (**56.8**)	1,311 (**91.6**)	905 (**63.2**)	328 (22.9)	893 (**62.4**)
			Level 2	1,113 (**77.8**)	272 (19.0)	689 (**48.1**)	610 (42.6)	742 (**51.9**)	612 (42.8)	111 (7.8)	451 (31.5)	869 (**60.7**)	472 (33.0)
			Level 3	39 (2.7)	15 (1.0)	328 (22.9)	807 **56.4**)	103 (7.2)	6 (0.4)	9 (0.6)	75 (5.2)	234 (16.4)	66 (4.6)
		Segmental instability (n=4,290)	Level 1	1,419 (33.1)	3,277 (**76.4**)	900 (21.0)	21 (0.5)	1,514 (35.3)	3,084 (**71.9**)	3,902 (**91.0**)	2,641 (**61.6**)	977 (22.8)	2,525 (**58.9**)
			Level 2	2,843 (**66.3**)	984 (22.9)	2,463 (**57.4**)	2,160 (**50.3**)	2,462 (**57.4**)	1,192 (27.8)	371 (8.6)	1,364 (31.8)	2,663 (**62.1**)	1,537 (35.8)
			Level 3	28 (0.7)	29 (0.7)	927 (21.6)	2,109 (49.2)	314 (7.3)	14 (0.3)	17 (0.4)	285 (6.6)	650 (15.2)	228 (5.3)
		Spinal stenosis (n=28,194)	Level 1	3,028 (10.7)	24,047 (**85.3**)	9321 (33.1)	162 (0.6)	12,977 (46.0)	13,069 (46.4)	25,673 (**91.1**)	17,978 (**63.8**)	5,697 (20.2)	17,464 (**61.9**)
			Level 2	24,966 (**88.6**)	3,877 (13.8)	15,161 (**53.8**)	14,520 (**51.5**)	13,874 (**49.2**)	15,049 (**53.4**)	2,330 (8.3)	8,926 (31.7)	18,071 (**64.1**)	9,650 (34.2)
			Level 3	200 (0.7)	270 (1.0)	3,712 (13.2)	13,512 (47.9)	1,343 (4.8)	76 (0.3)	191 (0.7)	1,290 (4.6)	4426 (15.7)	1,080 (3.8)
		Spondylolysis (n=2,463)	Level 1	675 (27.4)	2,120 (**86.1**)	703 (28.5)	16 (0.6)	980 (39.8)	1,657 (**67.3**)	2,274 (**92.3**)	1,601 (**65.0**)	581 (23.6)	1,513 (**61.4**)
			Level 2	1,769 (**71.8**)	330 (13.4)	1,373 (**55.7**)	1,316 (**53.4**)	1,319 (**53.6**)	804 (32.6)	181 (7.3)	742 (30.1)	1,552 (**63.0**)	815 (33.1)
			Level 3	19 (0.8)	13 (0.5)	387 (15.7)	1,131 (45.9)	164 (6.7)	2 (0.1)	8 (0.3)	120 (4.9)	330 (13.4)	135 (5.5)
	Hip	Avascular necrosis (n=594)	Level 1	79 (5.0)	985 (**61.8**)	358 (22.5)	30 (1.9)	741 (**46.5**)	857 (**53.8**)	1,383 (**86.8**)	1,085 (**68.1**)	624 (39.1)	1,076 (**67.5**)
			Level 2	1,476 (**92.6**)	570 (35.8)	838 (**52.6**)	592 (37.1)	716 (44.9)	732 (45.9)	198 (12.4)	434 (27.2)	869 (**54.5**)	477 (29.9)
			Level 3	39 (2.4)	39 (2.4)	398 (25.0)	972 (**61.0**)	137 (8.6)	5 (0.3)	13 (0.8)	75 (4.7)	101 (6.3)	41 (2.6)
		Childhood hip disorder (n=1,846)	Level 1	127 (6.9)	1,390 (**75.3**)	653 (35.4)	22 (1.2)	1,014 (**54.9**)	1,278 (**69.2**)	1,672 (**90.6**)	1,449 (**78.5**)	989 (**53.6**)	1,463 (**79.3**)
			Level 2	1,707 (**92.5**)	438 (23.7)	976 (**52.9**)	998 (**54.1**)	758 (41.1)	568 (30.8)	162 (8.8)	348 (18.9)	781 (42.3)	345 (18.7)
			Level 3	12 (0.7)	18 (1.0)	217 (11.8)	826 (44.7)	74 (4.0)	0 (0.0)	12 (0.7)	49 (2.7)	76 (4.1)	38 (2.1)
		Hip osteoarthritis (n=86,023)	Level 1	7,079 (8.2)	67,411 (**78.4**)	35,009 (40.7)	1,335 (1.6)	51,727 (**60.1**)	53,290 (**61.9**)	79,823 (**92.8**)	67,451 (**78.4**)	39,300 (45.7)	67,755 (**78.8**)
			Level 2	78,719 (**91.5**)	17,838 (20.7)	42,897 (**49.9**)	50,371 (**58.6**)	31,621 (36.8)	32,620 (37.9)	5,719 (6.6)	16,947 (19.7)	43,037 (**50.0**)	17,003 (19.8)
			Level 3	225 (0.3)	774 (0.9)	8,117 (9.4)	34,317 (39.9)	2,675 (3.1)	113 (0.1)	481 (0.6)	1,625 (1.9)	3,686 (4.3)	1,265 (1.5)
		Inflammatory joint disorder (n=1,197)	Level 1	55 (4.6)	697 (**58.2**)	344 (28.7)	13 (1.1)	638 (**53.3**)	545 (45.5)	922 (**77.0**)	698 (**58.3**)	357 (29.8)	808 (**67.5**)
			Level 2	1,124 (**93.9**)	461 (38.5)	660 (**55.1**)	558 (46.6)	502 (41.9)	645 (**53.9**)	240 (20.1)	432 (36.1)	735 (**61.4**)	363 (30.3)
			Level 3	18 (1.5)	39 (3.3)	193 (16.1)	626 (**52.3**)	57 (4.8)	7 (0.6)	35 (2.9)	67 (5.6)	105 (8.8)	26 (2.2)
	Knee	Knee osteoarthritis (n=16,020)	Level 1	1,884 (11.8)	15,010 (**93.7**)	8,605 (**53.7**)	290 (1.8)	10,484 (**65.4**)	10,127 (**63.2**)	15,275 (**95.3**)	12,513 (**78.1**)	5,818 (36.3)	12,620 (**78.8**)
			Level 2	14,097 (**88.0**)	869 (5.4)	6,585 (41.1)	10,139 (**63.3**)	5,155 (32.2)	5,867 (36.6)	661 (4.1)	3,246 (20.3)	9,380 (**58.6**)	3,133 (19.6)
			Level 3	39 (0.2)	141 (0.9)	830 (5.2)	5,591 (34.9)	381 (2.4)	26 (0.2)	84 (0.5)	261 (1.6)	822 (5.1)	267 (1.7)
		Knee rheumatoid arthritis (n=304)	Level 1	11 (3.6)	231 (**76.0**)	114 (37.5)	0 (0.0)	185 (**60.9**)	122 (40.1)	247 (**81.3**)	183 (**60.2**)	66 (21.7)	210 (**69.1**)
			Level 2	291 (**95.7**)	69 (22.7)	157 (**51.6**)	189 (**62.2**)	106 (34.9)	181 (**59.5**)	49 (16.1)	108 (35.5)	208 (**68.4**)	89 (29.3)
			Level 3	2 (0.7)	4 (1.3)	33 (10.9)	115 (37.8)	13 (4.3)	1 (0.3)	8 (2.6)	13 (4.3)	30 (9.9)	5 (1.6)

**Intervention-based registers**	**Ankle**	Other (n=85)	Level 1	6 (7.1)	73 (**85.9**)	31 (36.5)	2 (2.4)	46 (**54.1**)	27 (31.8)	75 (**88.2**)	47 (**55.3**)	22 (25.9)	56 (**65.9**)
			Level 2	77 (**90.6**)	11 (12.9)	39 (**45.9**)	42 (**49.4**)	36 (42.4)	57 (**67.1**)	9 (10.6)	33 (38.8)	56 (**65.9**)	29 (34.1)
			Level 3	2 (2.4)	1 (1.2)	15 (17.6)	41 (48.2)	3 (3.5)	1 (1.2)	1 (1.2)	5 (5.9)	7 (8.2)	0 (0.0)
		Posttraumatic osteoarthritis (n=308)	Level 1	16 (5.2)	285 (**92.5**)	125 (40.6)	4 (1.3)	166 (**53.9**)	109 (35.4)	290 (**94.2**)	200 (**64.9**)	68 (22.1)	217 (**70.5**)
			Level 2	288 (**93.5**)	20 (6.5)	147 (**47.7**)	168 (**54.5**)	123 (39.9)	198 (**64.3**)	15 (4.9)	93 (30.2)	211 (**68.5**)	76 (24.7)
			Level 3	4 (1.3)	3 (1.0)	36 (11.7)	136 (44.2)	19 (6.2)	1 (0.3)	3 (1.0)	15 (4.9)	29 (9.4)	15 (4.9)
		Primary osteoarthritis (n=234)	Level 1	7 (3.0)	214 (**91.5**)	91 (38.9)	2 (0.9)	152 (**65.0**)	97 (41.5)	219 (**93.6**)	165 (**70.5**)	70 (29.9)	182 (**77.8**)
			Level 2	224 (**95.7**)	18 (7.7)	118 (**50.4**)	118 (**50.4**)	78 (33.3)	136 (58.1)	13 (5.6)	65 (27.8)	137 (**58.5**)	51 (21.8)
			Level 3	3 (1.3)	2 (0.9)	25 (10.7)	114 (48.7)	4 (1.7)	1 (0.4)	2 (0.9)	4 (1.7)	27 (11.5)	1 (0.4)
		Rheumatoid arthritis (n=73)	Level 1	70 (0.0)	45 (**61.6**)	17 (23.3)	0 (0.0)	40 (**54.8**)	20 (27.4)	57 (**78.1**)	36 (**49.3**)	10 (13.7)	48 (**65.8**)
			Level 2	3 (**95.9**)	27 (37.0)	42 (**57.5**)	23 (31.5)	29 (39.7)	52 (**71.2**)	14 (19.2)	32 (43.8)	55 (**75.3**)	24 (32.9)
			Level 3	6 (4.1)	1 (1.4)	14 (19.2)	50 (**68.5**)	4 (5.5)	1 (1.4)	2 (2.7)	5 (6.8)	8 (11.0)	1 (1.4)
	**Cruciate ligament**	Cruciate ligament injury (n=8,430)	Level 1	5,645 (**67.0**)	8,209 (**97.4**)	4,503 (**53.4**)	1,294 (15.3)	4,190 (**49.7**)	7,308 (**86.7**)	8,337 (**98.9**)	6,663 (**79.0**)	2,943 (34.9)	5,501 (**65.3**)
			Level 2	2,762 (32.8)	178 (2.1)	3,172 (37.6)	6,658 (**79.0**)	3,779 (44.8)	1,117 (13.3)	69 (0.8)	1,636 (19.4)	5,223 (**62.0**)	2,649 (31.4)
			Level 3	23 (0.3)	43 (0.5)	755 (9.0)	478 (5.7)	461 (5.5)	5 (0.1)	24 (0.3)	131 (1.6)	264 (3.1)	280 (3.3)
	**BOA**	BOA hip (n=3,440)	Level 1	1,499 (43.6)	3,234 (**94.0**)	2,600 (**75.6**)	61 (1.8)	2,230 (**64.8**)	1,708 (**49.7**)	3,209 (**93.3**)	2,662 (**77.4**)	261 (7.6)	2,305 (**67.0**)
			Level 2	1,938 (**56.3**)	195 (5.7)	799 (23.2)	3,014 (**87.6**)	1,159 (33.7)	1,729 (50.3)	218 (6.3)	741 (21.5)	2,874 (**83.5**)	1,096 (31.9)
			Level 3	3 (0.1)	11 (0.3)	41 (1.2)	365 (10.6)	51 (1.5)	3 (0.1)	13 (0.4)	37 (1.1)	305 (8.9)	39 (1.1)
		BOA knee (n=10,207)	Level 1	4,282 (42.0)	9,886 (**96.9**)	7,674 (**75.2**)	290 (2.8)	6,751 (**66.1**)	5,388 (**52.8**)	9,884 (**96.8**)	8,218 (**80.5**)	1,003 (9.8)	7,248 (**71.0**)
			Level 2	5,916 (**58.0**)	275 (2.7)	2,401 (23.5)	8,741 (**85.6**)	3,314 (32.5)	4,813 (47.2)	276 (2.7)	1,905 (18.7)	8,404 (**82.3**)	2,834 (27.8)
			Level 3	9 (0.1)	46 (0.5)	132 (1.3)	1,176 (11.5)	142 (1.4)	6 (0.1)	47 (0.5)	84 (0.8)	800 (7.8)	125 (1.2)
	**Fracture**	Ankle and foot (n=3,718)	Level 1	3,048 (**82.0**)	3,570 (**96.0**)	3,265 (**87.8**)	2,549 (**68.6**)	3,095 (**83.2**)	2,717 (**73.1**)	3,578 (**96.2**)	3,112 (**83.7**)	1,832 (**49.3**)	3,005 (**80.8**)
			Level 2	655 (17.6)	126 (3.4)	322 (8.7)	1,058 (28.5)	556 (15.0)	991 (26.7)	106 (2.9)	537 (14.4)	1,733 (46.6)	645 (17.3)
			Level 3	15 (0.4)	22 (0.6)	131 (3.5)	111 (3.0)	67 (1.8)	10 (0.3)	34 (0.9)	69 (1.9)	153 (4.1)	68 (1.8)
		Elbow and forearm (n=13,996)	Level 1	12,548 (**89.7**)	12,981 (**92.7**)	12,304 (**87.9**)	9,502 (**67.9**)	11,699 (**83.6**)	12,104 (**86.5**)	13,107 (**93.6**)	11,649 (**83.2**)	6,686 (47.8)	11,390 (**81.4**)
			Level 2	1,434 (10.2)	885 (6.3)	1,189 (8.5)	4,161 (29.7)	2,127 (15.2)	1,848 (13.2)	748 (5.3)	2,026 (14.5)	6,924 (**49.5**)	2,406 (17.2)
			Level 3	14 (0.1)	130 (0.9)	503 (3.6)	333 (2.4)	170 (1.2)	44 (0.3)	141 (1.0)	321 (2.3)	386 (2.8)	200 (1.4)
		Hip and femur (n=8,378)	Level 1	4,490 (**53.6**)	6,379 (**76.1**)	5,444 (**65.0**)	3,740 (44.6)	5,486 (**65.5**)	2,419 (28.9)	5,679 (**67.8**)	4,243 (**50.6**)	2,286 (27.3)	4,818 (**57.5**)
			Level 2	3,746 (44.7)	1,526 (18.2)	1,846 (22.0)	4,092 (**48.8**)	2,570 (30.7)	5,584 (**66.7**)	1,879 (22.4)	2,636 (31.5)	5,458 (**65.1**)	3,205 (38.3)
			Level 3	142 (1.7)	473 (5.6)	1,088 (13.0)	546 (6.5)	322 (3.8)	375 (4.5)	820 (9.8)	1,499 (17.9)	634 (7.6)	355 (4.2)
		Knee and lower leg (n=10,866)	Level 1	9,117 (**83.9**)	10,177 (**93.7**)	9,499 (**87.4**)	7,736 (**71.2**)	9,029 (**83.1**)	7,023 (**64.6**)	10,228 (**94.1**)	8,584 (**79.0**)	4,212 (38.8)	8,354 (**76.9**)
			Level 2	1,661 (15.3)	570 (5.2)	871 (8.0)	2,835 (26.1)	1,657 (15.2)	3,762 (34.6)	471 (4.3)	1,939 (17.8)	6,208 (**57.1**)	2,286 (21.0)
			Level 3	88 (0.8)	119 (1.1)	496 (4.6)	295 (2.7)	180 (1.7)	81 (0.7)	167 (1.5)	343 (3.2)	446 (4.1)	226 (2.1)
		Lumbar spine and pelvis (n=1,128)	Level 1	708 (**62.8**)	902 (**80.0**)	775 (**68.7**)	547 (48.5)	790 (**70.0**)	548 (48.6)	77.5 (**77.5**)	682 (**60.5**)	412 (36.5)	741 (**65.7**)
			Level 2	400 (35.5)	179 (15.9)	236 (20.9)	478 (42.4)	293 (26.0)	561 (**49.7**)	16.1 (16.1)	296 (26.2)	621 (**55.1**)	348 (30.9)
			Level 3	20 (1.8)	47 (4.2)	117 (10.4)	103 (9.1)	45 (4.0)	19 (1.7)	6.4 (6.4)	150 (13.3)	95 (8.4)	39 (3.5)
		Shoulder and upper arm (n=7,835)	Level 1	6,615 (**84.4**)	6,926 (**88.4**)	6,547 (**83.6**)	4,891 (**62.4**)	6,248 (**79.7**)	6,237 (**79.6**)	6,721 (**85.8**)	5,877 (**75.0**)	2,912 (37.2)	5,876 (**75.0**)
			Level 2	1,189 (15.2)	743 (9.5)	863 (11.0)	2,607 (33.3)	1,447 (18.5)	1,548 (19.8)	940 (12.0)	1,616 (20.6)	4,573 (**58.4**)	1,794 (22.9)
			Level 3	31 (0.4)	166 (2.1)	425 (5.4)	337 (4.3)	140 (1.8)	50 (0.6)	174 (2.2)	342 (4.4)	350 (4.5)	165 (2.1)
		Unspecified part of trunk, extremities or other body region (n=749)	Level 1	569 (**76.0**)	655 (**87.4**)	583 (**77.8**)	448 (**59.8**)	577 (**77.0**)	463 (**61.8**)	641 (**85.6**)	470 (**62.8**)	172 (23.0)	492 (**65.7**)
			Level 2	171 (22.8)	78 (10.4)	99 (13.2)	249 (33.2)	147 (19.6)	273 (36.4)	86 (11.5)	211 (28.2)	494 (**66.0**)	224 (29.9)
			Level 3	9 (1.2)	16 (2.1)	67 (8.9)	52 (6.9)	25 (3.3)	13 (1.7)	22 (2.9)	68 (9.1)	83 (11.1)	33 (4.4)
		Wrist and hand (n=4,222)	Level 1	3,927 (**93.0**)	3,922 (**92.9**)	3,656 (**86.6**)	2,950 (**69.9**)	3,538 (**83.8**)	3,803 (**90.1**)	4,024 (**95.3**)	3,544 (**83.9**)	2,046 (**48.5**)	3,505 (**83.0**)
			Level 2	286 (6.8)	266 (6.3)	389 (9.2)	1,178 (27.9)	622 (14.7)	403 (9.5)	163 (3.9)	603 (14.3)	2,048 (**48.5**)	658 (15.6)
			Level 3	9 (0.2)	34 (0.8)	177 (4.2)	94 (2.2)	62 (1.5)	16 (0.4)	35 (0.8)	75 (1.8)	128 (3.0)	59 (1.4)

**Diagnosis-based registers**	**Heart failure**	Heart failure (n=1,436)	Level 1	866 (**60.3**)	1,315 (**91.6**)	1,041 (**72.5**)	689 (**48.0**)	865 (**60.2**)	837 (**58.3**)	1,298 (90.**4**)	1,025 (**71.4**)	685 (**47.7**)	896 (**62.4**)
			Level 2	567 (39.5)	116 (8.1)	355 (24.7)	679 (47.3)	521 (36.3)	593 (41.3)	126 (8.8)	371 (25.8)	655 (45.6)	499 (34.7)
			Level 3	3 (0.2)	5 (0.3)	40 (2.8)	68 (4.7)	50 (3.5)	6 (0.4)	12 (0.8)	40 (2.8)	96 (6.7)	41 (2.9)
	**Respir-atory failure**	COPD (n=900)	Level 1	199 (22.1)	551 (**61.2**)	215 (23.9)	250 (27.8)	357 (39.7)	162 (18.0)	479 (**53.2**)	179 (19.9)	212 (23.6)	320 (35.6)
			Level 2	683 (**75.9**)	296 (32.9)	492 (**54.7**)	517 (**57.4**)	463 (**51.4**)	696 (**77.3**)	343 (38.1)	465 (**51.7**)	541 (**60.1**)	489 (**54.3**)
			Level 3	18 (2.0)	53 (5.9)	193 (21.4)	133 (14.8)	80 (8.9)	42 (4.7)	78 (8.7)	256 (28.4)	147 (16.3)	91 (10.1)
		Lung fibrosis (n=150)	Level 1	44 (29.3)	99 (**66.0**)	57 (38.0)	55 (36.7)	75 (**50.0**)	25 (16.7)	72 (**48.0**)	32 (21.3)	44 (29.3)	67 (44.7)
			Level 2	103 (**68.7**)	47 (31.3)	65 (**43.3**)	83 (**55.3**)	69 (46.0)	118 (**78.7**)	66 (44.0)	61 (**40.7**)	86 (**57.3**)	70 (**46.7**)
			Level 3	3 (2.0)	4 (2.7)	28 (18.7)	12 (8.0)	6 (4.0)	7 (4.7)	12 (8.0)	57 (38.0)	20 (13.3)	13 (8.7)
	**Psoria-sis**	Psoriasis vulgaris (n=1,704)	Level 1	1,425 (**83.6**)	1,645 (**96.5**)	1,469 (**86.2**)	666 (39.1)	881 (**51.7**)	1,444 (**84.7**)	1,659 (**97.4**)	1,525 (**89.5**)	884 (**51.9**)	1,113 (**65.3**)
			Level 2	279 (16.4)	54 (3.2)	214 (12.6)	914 (**53.6**)	719 (42.2)	258 (15.1)	41 (2.4)	161 (9.4)	755 (44.3)	529 (31.0)
			Level 3	0 (0.0)	5 (0.3)	21 (1.2)	124 (7.3)	104 (6.1)	2 (0.1)	4 (0.2)	18 (1.1)	65 (3.8)	62 (3.6)
		Psoriasis vulgaris + anthropathic (n=537)	Level 1	311 (**57.9**)	484 (**90.1**)	368 (**68.5**)	91 (16.9)	234 (43.6)	356 (**66.3**)	494 (**92.0**)	422 (**78.6**)	141 (26.3)	314 (**58.5**)
			Level 2	226 (42.1)	50 (9.3)	154 (28.7)	359 (**66.9**)	262 (**48.8**)	180 (33.5)	41 (7.6)	101 (18.8)	342 (**63.7**)	194 (36.1)
			Level 3	0 (0.0)	3 (0.6)	15 (2.8)	87 (16.2)	41 (7.6)	1 (0.2)	2 (0.4)	14 (2.6)	54 (10.1)	29 (5.4)
		Psoriasis unspecified (n=439)	Level 1	328 (**74.7**)	425 (**96.8**)	346 (**78.8**)	128 (29.2)	203 (46.2)	351 (**80.0**)	425 (**96.8**)	382 (**87.0**)	206 (46.9)	298 (**67.9**)
			Level 2	110 (25.1)	11 (2.5)	86 (19.6)	263 (**59.9**)	204 (**46.5**)	88 (20.0)	13 (3.0)	50 (11.4)	210 (**47.8**)	124 (28.2)
			Level 3	1 (0.2)	3 (0.7)	7 (1.6)	48 (10.9)	32 (7.3)	0 (0.0)	1 (0.2)	7 (1.6)	23 (5.2)	17 (3.9)
	**Rheum-atology**	Rheumatoid arthritis (n=17,870)	Level 1	8,758 (49.0)	14,099 (**78.9**)	10,564 (**59.1**)	2,203 (12.3)	10,107 (**56.6**)	9,741 (**54.5**)	14,912 (**83.4**)	11,825 (**66.2**)	3,173 (17.8)	10,970 (**61.4**)
			Level 2	9,065 (**50.7**)	3,539 (19.8)	6,519 (36.5)	13,138 (**73.5**)	7,046 (39.4)	8,060 (45.1)	2,727 (15.3)	5,447 (30.5)	12,734 (**71.3**)	6,196 (34.7)
			Level 3	47 (0.3)	232 (1.3)	787 (4.4)	2,529 (14.2)	717 (4.0)	69 (0.4)	231 (1.3)	598 (3.3)	1,963 (11.0)	704 (3.9)
		Spondyloarthropathy (n=8,782)	Level 1	4,548 (**51.8**)	7,292 (**83.0**)	4,929 (**56.1**)	955 (10.9)	4,316 (**49.1**)	5,051 (**57.5**)	7,470 (**85.1**)	5,502 (**62.7**)	1,263 (14.4)	4,733 (**53.9**)
			Level 2	4,225 (48.1)	1,422 (16.2)	3,392 (38.6)	6,154 (**70.1**)	3,900 (44.4)	3,714 (42.3)	1,245 (14.2)	2,925 (33.3)	6,189 (**70.5**)	3,489 (39.7)
			Level 3	9 (0.1)	68 (0.8)	461 (5.2)	1,673 (19.1)	566 (6.4)	17 (0.2)	67 (0.8)	355 (4.0)	1,330 (15.1)	560 (6.4)
		Inflammatory systemic disorder (n=1,614)	Level 1	920 (**57.0**)	1,355 (**84.0**)	942 (**58.4**)	302 (18.7)	792 (**49.1**)	969 (**60.0**)	1,393 (**86.3**)	1,009 (**62.5**)	341 (21.1)	834 (**51.7**)
			Level 2	686 (42.5)	245 (15.2)	563 (34.9)	1,103 (**68.3**)	730 (45.2)	639 (39.6)	207 (12.8)	521 (32.3)	1,084 (**67.2**)	683 (42.3)
			Level 3	8 (0.5)	14 (0.9)	109 (6.8)	209 (12.9)	92 (5.7)	6 (0.4)	14 (0.9)	84 (5.2)	189 (11.7)	97 (6.0)
		Juvenile rheumatoid diseases (n=1,088)	Level 1	552 (**50.7**)	866 (**79.6**)	611 (**56.2**)	158 (14.5)	545 (**50.1**)	593 (**54.5**)	879 (**80.8**)	660 (**60.7**)	198 (18.2)	571 (**52.5**)
			Level 2	532 (48.9)	205 (18.8)	425 (39.1)	755 (**69.4**)	482 (44.3)	490 (45.0)	193 (17.7)	377 (34.7)	731 (**67.2**)	458 (42.1)
			Level 3	4 (0.4)	17 (1.6)	52 (4.8)	175 (16.1)	61 (5.6)	5 (0.5)	16 (1.5)	51 (4.7)	159 (14.6)	59 (5.4)
		Vasculitis (n=410)	Level 1	249 (**60.7**)	368 (**89.8**)	276 (**67.3**)	91 (22.2)	227 (**55.4**)	239 (**58.3**)	374 (**91.2**)	279 (**68.0**)	96 (23.4)	241 (**58.8**)
			Level 2	161 (39.3)	34 (8.3)	117 (28.5)	285 (**69.5**)	169 (41.2)	170 (41.5)	29 (7.1)	123 (30.0)	275 (**67.1**)	154 (37.6)
			Level 3	0 (0.0)	8 (2.0)	17 (4.1)	34 (8.3)	14 (3.4)	1 (0.2)	7 (1.7)	8 (2.0)	39 (9.5)	15 (3.7)
		Other arthritis (n=1698)	Level 1	856 (**50.4**)	1,414 (**83.3**)	982 (**57.8**)	182 (10.7)	930 (**54.8**)	992 (**58.4**)	1,495 (**88.0**)	1,168 (**68.8**)	302 (17.8)	1,059 (**62.4**)
			Level 2	837 (49.3)	273 (16.1)	629 (37.0)	1,268 (**74.7**)	687 (40.5)	703 (41.4)	190 (11.2)	480 (28.3)	1,193 (**70.3**)	573 (33.7)
			Level 3	5 (0.3)	11 (0.6)	87 (5.1)	248 (14.6)	81 (4.8)	3 (0.2)	13 (0.8)	50 (2.9)	203 (12.0)	66 (3.9)
**General population data** (n=49,169)			Level 1	44,279 (**90.1**)	48,371 (**98.4**)	44,848 (**91.2**)	24,946 (**50.8**)	32,721 (**66.5**)	**-**	**-**	**-**	-	**-**
			Level 2	4,840 (9.8)	600 (1.2)	3,785 (7.7)	22,185 (45.1)	15,126 (30.8)	-	-	-	**-**	-
			Level 3	50 (0.1)	198 (0.4)	536 (1.1)	2,038 (4.1)	1,322 (2.7)	-	-	-	-	-

The highest proportion in bold; Level 1: No problems; Level 2: Some/moderate problems; Level 3: confined to bed/unable to/extreme problems; Proportions rounded off to one decimal using MS Excel

Table S4: Median (IQR) EQ VAS value at baseline, 1-year follow-up and the change in the 9 National Quality Registers (NQRs) and in the general population

Time	Variable	Median EQ VAS value (IQR)									
		Intervention-based registers							Diagnosis-based registers		General population
		Spine	Hip	Knee	Ankle	Cruciate ligament	BOA	Fracture	Heart failure	Respiratory failure	
**Baseline**	**Overall**	50.0 (35.0)	58.0 (35.0)	70.0 (30.0)	57.5 (30.0)	68.0 (32.0)	70.0 (23.0)	90.0 (20.0)	70.0 (30.0)	50.0 (25.0)	85.0 (15.0)
	**Sex**										
	Men	50.0 (40.0)	60.0 (30.0)	71.0 (32.0)	60.0 (35.0)	70.0 (30.0)	75.0 (25.0)	90.0 (20.0)	70.0 (25.0)	50.0 (25.0)	85.0 (15.0)
	Women	45.0 (30.0)	50.0 (31.0)	65.0 (30.0)	50.0 (32.5)	66.0 (34.0)	70.0 (25.0)	90.0 (21.0)	60.0 (25.0)	50.0 (28.0)	84.0 (20.0)
	**Age group**										
	<30	45.0 (35.0)	43.0 (40.8)	-	52.5 (23.8)	69.0 (31.0)	70.0 (1.3)	95.0 (15.0)	-	-	85.0 (15.0)
	30-39	41.0 (30.0)	50.0 (35.0)	60.5 (36.8)	48.0 (26.3)	68.0 (30.0)	65.0 (30.0)	93.0 (15.0)	70.0 (30.0)	-	85.0 (15.0)
	40-49	45.0 (32.0)	55.0 (35.0)	59.5 (40.0)	47.5 (30.0)	65.0 (36.0)	70.0 (30.0)	92.0 (15.0)	67.5 (20.0)	-	85.0 (15.0)
	50-59	50.0 (35.0)	55.0 (34.0)	62.0 (39.0)	55.0 (35.0)	66.0 (34.0)	70.0 (30.0)	92.0 (20.0)	62.0 (25.0)	50.0 (45.0)	84.0 (20.0)
	60-69	50.0 (36.5)	60.0 (35.0)	70.0 (30.0)	55.0 (35.0)	69.0 (40.0)	70.0 (27.0)	91.0 (20.0)	70.0 (20.0)	50.0 (29.0)	83.0 (20.0)
	70-79	50.0 (40.0)	60.0 (35.0)	70.0 (31.0)	65.0 (30.8)	-	70.0 (20.3)	90.0 (24.0)	70.0 (30.0)	50.0 (25.0)	80.0 (20.0)
	80+	50.0 (36.0)	52.0 (30.0)	70.0 (30.0)	50.0 (30.0)	-	70.0 (27.0)	75.0 (35.0)	60.0 (25.0)	50.0 (30.0)	80.0 (40.0)
	**BMI (Kg/m^2)**										
	Under-weight	40.0 (34.8)	50.0 (30.0)	70.0 (25.3)	27.0 (27.5)	76.5 (30.0)	67.5 (20.0)	-	-	50.0 (26.3)	80.0 (20.0)
	Normal	50.0 (35.0)	60.0 (35.0)	70.0 (33.0)	60.0 (31.2)	70.0 (31.0)	75.0 (25.0)	-	-	50.0 (30.0)	85.0 (15.0)
	Over-weight	50.0 (36.0)	60.0 (35.0)	70.0 (31.0)	60.0 (32.0)	70.0 (31.0)	70.0 (20.0)	-	-	50.0 (25.0)	80.0 (20.0)
	Obesity class I	50.0 (35.0)	53.0 (30.0)	65.0 (30.0)	55.0 (30.0)	62.0 (38.0)	69.0 (30.0)	-	-	50.0 (29.0)	80.0 (30.0)
	Obesity class II	40.0 (33.0)	50.0 (37.0)	60.0 (38.0)	50.0 (25.0)	62.0 (37.5)	65.0 (29.0)	-	-	50.0 (25.0)	75.0 (35.0)
	Obesity class III	40.0 (30.0)	50.0 (40.0)	60.0 (32.3)	42.0 (22.5)	64.5 (16.5)	58.0 (30.0)	-	-	60.0 (20.5)	70.0 (35.0)
**1-year follow-up**	**Overall**	70.0 (35.0)	80.0 (23.0)	80.0 (20.0)	75.0 (22.0)	80.0 (24.0)	75.0 (25.0)	80.0 (23.0)	70.0 (20.3)	50.0 (28.0)	-
	**Sex**										
	Men	75.0 (28.0)	81.0 (23.0)	80.0 (20.0)	75.0 (20.0)	80.0 (23.0)	75.0 (25.0)	84.0 (24.0)	70.0 (20.0)	50.0 (25.0)	-
	Women	70.0 (35.0)	80.0 (30.0)	80.0 (25.0)	70.0 (26.5)	78.0 (24.0)	74.0 (25.0)	80.0 (23.0)	70.0 (30.0)	50.0 (25.0)	-
	**Age group**										
	<30	80.0 (25.0)	80.0 (23.8)	-	67.5 (42.3)	80.0 (24.0)	76.5 (26.0)	88.0 (20.0)	-	-	-
	30-39	75.0 (29.0)	80.0 (20.0)	82.5 (10.0)	70.0 (25.5)	80.0 (25.0)	78.0 (15.0)	88.0 (20.0)	90.0 (30.0)	-	-
	40-49	75.0 (30.0)	85.0 (23.0)	75.0 (28.0)	70.0 (27.5)	80.0 (23.0)	70.0 (23.0)	85.0 (20.0)	73.0 (20.0)	-	-
	50-59	75.0 (36.0)	83.0 (24.0)	80.0 (28.0)	75.0 (20.0)	78.0 (24.0)	73.0 (25.0)	85.0 (25.0)	70.0 (24.0)	40.0 (35.0)	-
	60-69	73.0 (34.0)	85.0 (25.0)	80.0 (20.0)	75.0 (20.8)	84.0 (22.0)	74.0 (25.0)	86.0 (21.0)	75.0 (24.3)	50.0 (25.0)	-
	70-79	70.0 (30.0)	80.0 (25.0)	80.0 (20.0)	75.0 (25.0)	-	75.0 (26.0)	80.0 (25.0)	70.0 (25.0)	50.0 (27.3)	-
	80+	60.0 (29.0)	75.0 (34.0)	78.0 (30.0)	60.0 (30.0)	-	71.0 (30.0)	65.0 (30.0)	65.0 (30.0)	50.0 (23.8)	-
	**BMI**										
	Underweight	70.0 (38.5)	76.0 (30.0)	70.0 (18.8)	45.0 (15.0)	78.5 (25.5)	74.5 (17.8)	-	-	42.5 (26.3)	-
	Normal weight	75.0 (30.0)	85.0 (25.0)	80.0 (20.0)	75.0 (20.0)	80.0 (23.0)	78.0 (24.0)	-	-	50.0 (25.0)	-
	Over-weight	75.0 (31.0)	80.0 (21.0)	80.0 (20.0)	75.0 (10.0)	80.0 (25.0)	75.0 (25.0)	-	-	50.0 (23.0)	-
	Obesity class I	70.0 (30.0)	80.0 (30.0)	80.0 (25.0)	70.0 (23.5)	75.0 (25.0)	70.0 (29.0)	-	-	50.0 (29.5)	-
	Obesity class II	65.0 (40.0)	75.0 (30.0)	75.0 (30.0)	75.0 (37.5)	75.0 (32.0)	70.0 (28.0)	-	-	50.0 (23.8)	-
	Obesity class III	66.0 (36.3)	70.0 (37.8)	70.0 (34.0)	35.0 (21.3)	68.0 (34.8)	60.0 (35.0)	-	-	50.0 (15.0)	-

**Change**	**Overall**	20.0 (40.0)	20.0 (35.0)	10.0 (26.0)	11.5 (30.0)	10.0 (30.0)	0.0 (19.0)	-5.0 (15.0)	0.0 (20.0)	0.0 (28.0)	-
	**Sex**										
	Men	17.0 (36.0)	18.0 (32.0)	8.0 (22.0)	10.0 (30.0)	10.0 (29.0)	0.0 (20.0)	-5.0 (15.0)	0.0 (16.0)	0.0 (25.0)	-
	Women	20.0 (38.0)	20.0 (35.0)	10.0 (31.0)	15.0 (35.0)	10.0 (30.0)	2.0 (21.0)	-5.0 (15.0)	0.0 (22.0)	0.0 (30.0)	-
	**Age group**										
	<30	25.0 (35.0)	30.0 (40.0)	-	12.5 (45.3)	9.0 (30.0)	4.0 (22.3)	-5.0 (15.0)	-	-	-
	30-39	25.0 (37.0)	24.0 (31.0)	14.5 (35.3)	17.5 (22.3)	10.0 (29.0)	10.0 (21.0)	-5.0 (15.0)	0.0 (0.0)	-	-
	40-49	25.0 (40.0)	24.0 (35.0)	15.0 (35.0)	20.0 (27.5)	13.0 (32.0)	5.0 (20.0)	-5.0 (15.0)	7.5 (14.0)	-	-
	50-59	20.0 (36.0)	20.0 (34.0)	10.0 (31.0)	15.0 (30.0)	10.0 (27.0)	4.0 (20.0)	-5.0 (15.0)	9.0 (20.0)	-10.0 (25.0)	-
	60-69	20.0 (38.0)	20.0 (35.0)	10.0 (29.0)	15.0 (32.0)	7.0 (41.0)	1.0 (18.0)	-5.0 (13.0)	0.0 (15.0)	0.0 (28.3)	-
	70-79	13.0 (30.0)	18.0 (35.0)	9.0 (25.0)	10.0 (25.0)	-	0.0 (20.0)	-5.0 (15.0)	0.0 (20.0)	0.0 (29.5)	-
	80+	10.0 (33.0)	15.0 (31.0)	7.0 (25.0)	10.0 (27.0)	-	0.0 (21.0)	-8.0 (22.0)	0.0 (20.0)	0.0 (28.3)	-
	**BMI**										
	Underweight	20.0 (36.5)	20.0 (30.0)	9.5 (27.5)	18.0 (12.5)	1.5 (22.3)	-0.5 (21.5)	-	-	-5.0 (28.5)	-
	Normal weight	20.0 (35.0)	20.0 (34.0)	10.0 (26.0)	10.0 (30.0)	9.0 (30.0)	1.0 (15.0)	-	-	0.0 (30.0)	-
	Over-weight	20.0 (39.0)	20.0 (32.0)	10.0 (26.0)	12.5 (30.0)	9.0 (30.0)	0.0 (19.0)	-	-	0.0 (22.8)	-
	Obesity class I	16.0 (35.0)	20.0 (39.0)	10.0 (30.0)	13.0 (30.0)	11.0 (35.0)	0.0 (23.0)	-	-	0.0 (33.0)	-
	Obesity class II	15.0 (35.0)	20.0 (39.0)	10.0 (32.0)	25.0 (42.5)	18.0 (35.5)	3.0 (25.0)	-	-	-2.5 (30.0)	-
	Obesity class III	15.0 (40.0)	20.0 (40.0)	10.0 (38.0)	0.0 (13.8)	-4.0 (27.5)	4.0 (20.0)	-	-	0.0 (20.0)	-

BMI categories: Underweight: <18.5; Normal weight: 18.5–24.9; Overweight: 25.0–29.9; Obesity class I: 30.0–34.9; Obesity class II: 35.0–39.9; Obesity class III: ≥40

Table S5: Two-level random intercept model of predictive effect of diagnoses on EQ VAS score at baseline and at 1-year follow-up, adjusted for sex and age

Variable	Baseline		1-year follow-up	
Category	Estimate	SE	Estimate	SE
Intercept	83.58	0.1894	82.01	0.1822
Spine, disc hernia	-34.61	0.2170	-7.52	0.2087
Spine, other spinal conditions	-33.03	0.5872	-11.57	0.5647
Spine, segmental instability	-32.85	0.3414	-11.07	0.3283
Spine, spinal stenosis	-29.91	0.1816	-11.77	0.1746
Spine, spondylolysis	-30.49	0.4469	-10.18	0.4298
Hip, avascular necrosis	-29.65	0.5306	-4.84	0.5104
Hip, childhood hip disorder	-23.21	0.4920	0.80	0.4731
Hip, hip osteoarthritis	-22.37	0.1474	-0.30	0.1418
Hip, inflammatory joint disorder	-30.74	0.6082	-9.12	0.5850
Knee osteoarthritis	-14.22	0.2088	-0.91	0.2008
Knee rheumatoid arthritis	-22.56	1.1891	-9.71	1.1436
Ankle, others	-21.57	2.2660	-10.41	2.1790
Ankle, posttraumatic OA	-23.77	1.2164	-8.54	1.1698
Ankle, primary OA	-21.97	1.3805	-4.64	1.3277
Ankle, rheumatoid arthritis	-32.78	2.4670	-15.98	2.3726
Cruciate ligament injury	-18.18	0.2627	-6.08	0.2527
BOA hip	-10.97	0.5088	-9.42	0.4893
BOA knee	-9.88	0.3193	-6.55	0.3071
Fracture, ankle and foot	7.98	0.3836	2.46	0.3689
Fracture, elbow and forearm	9.33	0.2245	4.25	0.2159
Fracture, hip and femur	-3.47	0.3032	-9.98	0.2916
Fracture, knee and lower leg	7.88	0.2427	0.37	0.2334
Fracture, lumbar, spine and pelvis	-0.83	0.7293	-5.84	0.7014
Fracture, shoulder and upper arm	6.16	0.2855	0.40	0.2746
Fracture, unspecified part of trunk, extremities or other body region	3.98	0.8303	-7.40	0.7986
Fracture, wrist and hand	8.06	0.3601	3.97	0.3463
Heart failure	-14.14	0.6524	-9.14	0.6274
Respiratory failure, COPD	-27.81	0.8386	-27.11	0.8065
Respiratory failure, pulmonary fibrosis	-25.88	2.0265	-27.06	1.9490
n	251,008		251,008	
AIC	2,231,535		2,211,956	
BIC	2,231,942		2,212,363	

AIC: Akaike information criterion; AIC: Baysian information criterion; SE: Standard error; reference group: General population

Teni FS, *et al. BMJ Open* 2022; 12:e048176. doi: 10.1136/bmjopen-2020-048176

Table S6: Prevalence of reported problems on EQ-5D-3L by diagnosis and mean EQ VAS, and EQ-5D indices (TTO, VAS) among patients with complete data at baseline regardless of follow-up status and in the general population

	Patient group in NQRs	Diagnosis	Severity level	Mobility	Self-care	Usual activities	Pain/ discomfort	Anxiety/ depression	Mean (SD)		
				n (%)	n (%)	n (%)	n (%)	n (%)	EQ VAS*	EQ-5D index (TTO) **	EQ-5D index (VAS) **
Intervention-based registers	**Spine**	Disc hernia (n=19,214)	Level 1	2,925 (15.2)	12,853 (**66.9**)	3,464 (18.0)	135 (0.7)	6,634 (34.5)			
			Level 2	15,295 (**79.6**)	5,958 (31.0)	9,815 (**51.1**)	8,181 (42.6)	10,811 (**56.3**)			
			Level 3	994 (5.2)	403 (2.1)	5,935 (30.9)	10,898 (**56.7**)	1,769 (9.2)	45.0 (22.5)	0.66 (0.13)	45.2 (15.9)
		Other spinal conditions (n=2,209)	Level 1	435 (19.7)	1,746 (**79.0**)	615 (27.8)	21 (1.0)	876 (39.7)			
			Level 2	1,714 (**77.6**)	435 (19.7)	1,085 (**49.1**)	922 (41.7)	1,150 (**52.1**)			
			Level 3	60 (2.7)	28 (1.3)	509 (23.0)	1,266 (**57.3**)	183 (8.3)	45.8 (22.9)	0.69 (0.13)	47.9 (16.8)
		Segmental instability (n=5,892)	Level 1	1,851 (31.4)	4,473 (**75.9**)	1,184 (20.1)	28 (0.5)	1,965 (33.4)			
			Level 2	4,000 (**67.9**)	1,374 (23.3)	3,402 (**57.7**)	2,852 (48.4)	3,442 (**58.4**)			
			Level 3	41 (0.7)	45 (0.8)	1,306 (22.2)	3,012 (**51.1**)	485 (8.2)	45.8 (20.9)	0.69 (0.12)	48.3 (16.3)
		Spinal stenosis (n=38,449)	Level 1	3,985 (10.4)	32,361 (**84.2**)	12,289 (32.0)	237 (0.6)	17,157 (44.6)			
			Level 2	34,146 (**88.8**)	5,703 (14.8)	20,734 (**53.9**)	19,231 (**50.0**)	19,155 (**49.8**)			
			Level 3	318 (0.8)	385 (1.0)	5,426 (14.1)	18,981 (49.4)	2,137 (5.6)	48.6 (22.2)	0.71 (0.12)	50.0 (15.8)
		Spondylolysis (n=3,362)	Level 1	887 (26.4)	2,859 (**85.0**)	928 (27.6)	21 (0.6)	1,271 (37.8)			
			Level 2	2,449 (**72.8**)	482 (14.3)	1,877 (**55.8**)	1,723 (**51.2**)	1,847 (**54.9**)			
			Level 3	26 (0.8)	21 (0.6)	557 (16.6)	1,618 (48.1)	244 (7.3)	48.1 (21.8)	0.71 (0.13)	50.1 (16.7)
	**Hip**	Avascular necrosis (n=2,070)	Level 1	106 (5.1)	1,252 (**60.5**)	455 (22.0)	40 (1.9)	930 (44.9)			
			Level 2	1,908 (**92.2**)	761 (36.8)	1,065 (**51.4**)	748 (36.1)	958 (**46.3**)			
			Level 3	56 (2.7)	57 (2.8)	550 (26.6)	1,282 (**61.9**)	182 (8.8)	49.0 (24.0)	0.66 (0.13)	45.4 (15.8)
		Childhood hip disorder (n=2,294)	Level 1	165 (7.2)	1,698 (**74.0**)	776 (33.8)	26 (1.1)	1,226 (**53.4**)			
			Level 2	2,110 (**92.0**)	567 (24.7)	1,227 (**53.5**)	1,210 (**52.7**)	963 (42.0)			
			Level 3	19 (0.8)	29 (1.3)	291 (12.7)	1,058 (46.1)	105 (4.6)	55.2 (22.8)	0.71 (0.12)	51.4 (15.8)
		Hip osteoarthritis (n=103,286)	Level 1	8,320 (8.1)	80,304 (**77.7**)	41,136 (39.8)	1,562 (1.5)	61,196 (**59.2**)			
			Level 2	94,660 (**91.6**)	21,997 (21.3)	51,946 (**50.3**)	59,506 (**57.6**)	38,663 (37.4)			
			Level 3	306 (0.3)	985 (1.0)	10,204 (9.9)	42,218 (40.9)	3,427 (3.3)	56.3 (22.2)	0.73 (0.11)	54.0 (15.4)
		Inflammatory joint disorder (n=1,377)	Level 1	64 (4.6)	795 (**57.7**)	388 (28.2)	15 (1.1)	723 (**52.5**)			
			Level 2	1,292 (**93.8**)	538 (39.1)	762 (**55.3**)	634 (46.0)	586 (42.6)			
			Level 3	21 (1.5)	44 (3.2)	227 (16.5)	728 (**52.9**)	68 (4.9)	47.6 (22.6)	0.69 (0.12)	49.0 (15.2)
	**Knee**	Knee osteoarthritis (n=22,612)	Level 1	2,511 (11.1)	20,989 (**92.8**)	11,596 (**51.3**)	404 (1.8)	14,393 (**63.7**)			
			Level 2	20,047 (**88.7**)	1,408 (6.2)	9,705 (42.9)	14,073 (**62.2**)	7,604 (33.6)			
			Level 3	54 (0.2)	215 (1.0)	1,311 (5.8)	8,135 (36.0)	615 (2.7)	63.9 (22.4)	0.76 (0.11)	57.3 (15.3)
		Knee rheumatoid arthritis (n=450)	Level 1	14 (3.1)	330 (**73.3**)	160 (35.6)	2 (0.4)	261 (**58.0**)			
			Level 2	433 (**96.2**)	116 (25.8)	241 (**53.6**)	256 (**56.9**)	171 (38.0)			
			Level 3	3 (0.7)	4 (0.9)	49 (10.9)	192 (42.7)	18 (4.0)	53.8 (22.2)	0.72 (0.11)	52.3 (15.3)

Teni FS, *et al. BMJ Open* 2022; 12:e048176. doi: 10.1136/bmjopen-2020-048176

Intervention-based registers	**Ankle**	Other (n=147)	Level 1	7 (4.8)	120 (**81.6**)	43 (29.3)	8 (2.4)	73 (**49.7**)			
			Level 2	133 (**90.5**)	23 (15.6)	71 (**48.3**)	72 (**49.4**)	64 (43.5)			
			Level 3	7 (4.8)	4 (2.7)	33 (22.4)	67 (48.2)	10 (6.8)	56.3 (22.5)	0.70 (0.12)	49.4 (16.6)
		Posttraumatic osteoarthritis (n=517)	Level 1	26 (5.0)	476 (**92.1**)	194 (37.5)	4 (1.3)	273 (**52.8**)			
			Level 2	487 (**94.2**)	36 (7.0)	260 (**50.3**)	276 (**54.5**)	214 (41.4)			
			Level 3	4 (0.8)	5 (1.0)	63 (12.2)	237 (44.2)	30 (5.8)	56.9 (20.9)	0.72 (0.12)	51.5 (15.5)
		Primary osteoarthritis (n=374)	Level 1	9 (2.4)	339 (**90.6**)	141 (37.7)	4 (0.9)	236 (**63.1**)			
			Level 2	360 (**96.3**)	32 (8.6)	193 (**51.6**)	196 (**50.4**)	127 (34.0)			
			Level 3	5 (1.3)	3 (0.8)	40 (10.7)	174 (48.7)	11 (2.9)	57.5 (22.0)	0.73 (0.11)	52.7 (14.8)
		Rheumatoid arthritis (n=94)	Level 1	0 (0.0)	60 (**63.8**)	22 (23.4)	0 (0.0)	52 (**55.3**)			
			Level 2	91 (**96.8**)	32 (34.0)	57 (**60.6**)	33 (31.5)	37 (39.4)			
			Level 3	3 (3.2)	2 (2.1)	15 (16.0)	61 (**68.5**)	5 (5.3)	47.9 (22.4)	0.68 (0.11)	46.6 (14.2)
	**Cruciate ligament**	Cruciate ligament injury (n=18,530)	Level 1	12,195 (**65.8**)	17,997 (**97.1**)	9,878 (**53.3**)	2,851 (15.4)	9,046 (**48.8**)			
			Level 2	6,285 (33.9)	423 (2.3)	6,846 (36.9)	14,599 (**78.8**)	8,359 (45.1)			
			Level 3	50 (0.3)	110 (0.6)	1,806 (9.7)	1,080 (5.8)	1,125 (6.1)	62.4 (22.8)	0.82 (0.12)	65.6 (16.4)
	**BOA**	BOA hip (n=8,044)	Level 1	3,271 (40.7)	7,509 (**93.3**)	5,886 (**73.2**)	183 (2.3)	5,040 (**62.7**)			
			Level 2	4,769 (**59.3**)	503 (6.3)	2,036 (25.3)	6,778 (**84.3**)	2,864 (35.6)			
			Level 3	4 (0.0)	32 (0.4)	122 (1.5)	1,083 (13.5)	140 (1.7)	65.8 (19.1)	0.83 (0.10)	66.7 (14.4)
		BOA knee (n=24,846)	Level 1	9,557 (38.5)	23,888 (**96.1**)	17,812 (**71.7**)	713 (2.9)	15,824 (**63.7**)			
			Level 2	15,270 (**61.5**)	837 (3.4)	6,582 (26.5)	20,510 (**82.5**)	8,505 (34.2)			
			Level 3	19 (0.1)	121 (0.5)	452 (1.8)	3,623 (14.6)	517 (2.1)	66.6 (19.4)	0.83 (0.11)	66.2 (14.9)
	**Fracture**	Ankle and foot (n=6,918)	Level 1	5,522 (**79.8**)	6,567 (**94.9**)	5,916 (**85.5**)	4,639 (**67.1**)	5,590 (**80.8**)			
			Level 2	1,352 (19.5)	290 (4.2)	711 (10.3)	2,034 (29.4)	1,196 (17.3)			
			Level 3	44 (0.6)	61 (0.9)	291 (4.2)	245 (3.5)	132 (1.9)	86.2 (17.3)	0.91 (0.11)	79.4 (15.1)
		Elbow and forearm (n=25,061)	Level 1	22,079 (**88.1**)	22,724 (**90.7**)	21,352 (**85.2**)	16,414 (**65.5**)	20,355 (**81.2**)			
			Level 2	2,938 (11.7)	1972 (7.9)	2,512 (10.0)	7,885 (31.5)	4,321 (17.2)			
			Level 3	44 (0.2)	365 (1.5)	1,197 (4.8)	762 (3.0)	385 (1.5)	86.2 (17.2)	0.91 (0.10)	80.1 (14.1)
		Hip and femur (n=1,609)	Level 1	7,769 (**46.0**)	11,190 (**66.2**)	9,314 (**55.1**)	6,756 (40.0)	9,865 (**58.4**)			
			Level 2	8,535 (50.5)	3,982 (23.6)	4,078 (24.1)	8,738 (**51.7**)	6,023 (35.6)			
			Level 3	600 (3.5)	1,732 (10.2)	3,512 (20.8)	1,410 (8.3)	1,016 (6.0)	68.9 (24.5)	0.80 (0.16)	64.9 (21.0)
		Knee and lower leg (n=18,872)	Level 1	15,311 (**81.1**)	17,313 (**91.7**)	15,987 (**84.7**)	13,022 (**69.0**)	15,195 (**80.5**)			
			Level 2	3,283 (17.4)	1,182 (6.3)	1,736 (9.2)	5,229 (27.7)	3,283 (17.4)			
			Level 3	278 (1.5)	377 (2.0)	1,149 (6.1)	621 (3.3)	394 (2.1)	85.7 (17.9)	0.91 (0.12)	79.3 (15.9)
		Lumbar, spine and pelvis (n=2,405)	Level 1	1,298 (**54.0**)	1,735 (**72.1**)	1,444 (**60.0**)	1,049 (43.6)	1,507 (**62.7**)			
			Level 2	1,058 (44.0)	501 (20.8)	550 (22.9)	1,115 (**46.4**)	765 (31.8)			
			Level 3	49 (2.0)	169 (7.0)	411 (17.1)	241 (10.0)	133 (5.5)	73.0 (24.3)	0.82 (0.16)	67.5 (21.3)
		Shoulder and upper arm (n=14,240)	Level 1	11,546 (**81.1**)	12,159 (**85.4**)	11,323 (**79.5**)	8,519 (**59.8**)	10,806 (**75.9**)			
			Level 2	2,606 (18.3)	1,624 (11.4)	1841 (12.9)	4,995 (35.1)	3,103 (21.8)			
			Level 3	88 (0.6)	457 (3.2)	1,076 (7.6)	726 (5.1)	331 (2.3)	82.8 (20.0)	0.89 (0.12)	77.1 (16.9)
		Unspecified part of trunk, extremities or other body region (n=1,698)	Level 1	1,202 (**70.8**)	1,431 (**84.3**)	1,253 (**73.8**)	947 (**55.8**)	1,207 (**71.1**)			
			Level 2	474 (27.9)	213 (12.5)	263 (15.5)	610 (35.9)	430 (25.3)			
			Level 3	22 (1.3)	54 (3.2)	182 (10.7)	141 (8.3)	61 (3.6)	80.6 (22.0)	0.87 (0.15)	73.7 (19.9)
		Wrist and hand (n=8,215)	Level 1	7,533 (**91.7**)	7,522 (**91.6**)	6,947 (**84.6**)	5,617 (**68.4**)	6,783 (**82.6**)			
			Level 2	661 (8.0)	596 (7.3)	847 (10.3)	2,386 (29.0)	1,288 (15.7)			
			Level 3	21 (0.3)	97 (1.2)	421 (5.1)	212 (2.6)	144 (1.8)	87.6 (16.1)	0.92 (0.10)	80.7 (13.6)

Diagnosis-based registers	**Heart failure**	Heart failure (n=20,771)	Level 1	12,331 (**59.4**)	18,741 (**90.2**)	14,424 (**69.4**)	10,754 (**51.8**)	12,213 (**58.8**)			
			Level 2	8,334 (40.1)	1,817 (8.7)	5,548 (26.7)	8,920 (42.9)	7,748 (37.3)			
			Level 3	106 (0.5)	213 (1.0)	799 (3.8)	1,097 (5.3)	810 (3.9)	64.6 (19.2)	0.85 (0.12)	71.5 (16.5)
	**Respiratory failure**	COPD (n=3,407)	Level 1	688 (20.2)	1,819 (**53.4**)	830 (24.4)	980 (28.8)	1,267 (37.2)			
			Level 2	2,583 (**75.8**)	1,334 (39.2)	1,724 (**50.6**)	1,941 (**57.0**)	1,830 (**53.7**)			
			Level 3	136 (4.0)	254 (7.5)	853 (25.0)	486 (14.3)	310 (9.1)	47.8 (21.6)	0.72 (0.13)	53.9 (17.4)
		Lung fibrosis (n=892)	Level 1	206 (23.1)	536 (**60.1**)	257 (28.8)	298 (33.4)	405 (45.4)			
			Level 2	655 (**73.4**)	300 (33.6)	413 (**46.3**)	484 (**54.3**)	427 (**47.9**)			
			Level 3	31 (3.5)	56 (6.3)	222 (24.9)	110 (12.3)	60 (6.7)	48.1 (22.0)	0.74 (0.14)	56.6 (17.7)
	**Psoriasis**	Psoriasis vulgaris (n=3,943)	Level 1	3,242 (**82.2**)	3,774 (**95.7**)	3,359 (**85.2**)	1,492 (37.8)	2,000 (**50.7**)			
			Level 2	698 (17.7)	148 (3.8)	522 (13.2)	2,140 (**54.3**)	1,682 (42.7)			
			Level 3	3 (0.1)	21 (0.5)	62 (1.6)	311 (7.9)	261(6.6)	**-**	0.87 (0.11)	73.3 (15.7)
		Psoriasis vulgaris + anthropathic (n=1,128)	Level 1	667 (**59.1**)	1,018 (**90.2**)	773 (**68.5**)	173 (15.3)	526 (**46.6**)			
			Level 2	459 (40.7)	101 (9.0)	321 (28.5)	780 (**69.1**)	515 (45.7)			
			Level 3	2 (0.2)	9 (0.8)	34 (3.0)	175 (15.5)	87 (7.7)	-	0.82 (0.14)	65.7 (18.2)
		Psoriasis unspecified (n=1,090)	Level 1	810 (**74.3**)	1,017 (**93.3**)	842 (**77.2**)	345 (31.7)	539 (**49.4**)			
			Level 2	278 (25.5)	64 (5.9)	216 (19.8)	627 (**57.5**)	469 (43.0)			
			Level 3	2 (0.2)	9 (0.8)	32 (2.9)	118 (10.8)	82 (7.5)	-	0.85 (0.13)	70.3 (17.8)
	**Rheumatology**	Rheumatoid arthritis (n=29,015)	Level 1	14,345 (**49.4**)	22,963 (**79.1**)	17,249 (**59.4**)	3,709 (12.8)	16,449 (**56.7**)			
			Level 2	14,588 (50.3)	5,674 (19.6)	10,483 (36.1)	21,165 (**72.9**)	11,327 (39.0)			
			Level 3	82 (0.3)	378 (1.3)	1,283 (4.4)	4,141 (14.3)	1,239 (4.3)	**-**	0.81 (0.13)	65.1 (17.7)
		Spondyloarthropathy (n=15,175)	Level 1	7,892 (**52.0**)	12,697 (**83.7**)	8,672 (**57.1**)	1,622 (10.7)	7,504 (**49.4**)			
			Level 2	7,257 (47.8)	2,382 (15.7)	5,735 (37.8)	10,680 (**70.4**)	6,645 (43.8)			
			Level 3	26 (0.2)	96 (0.6)	768 (5.1)	2,873 (18.9)	1,026 (6.8)	**-**	0.80 (0.14)	63.1 (18.9)
		Inflammatory systemic disorder (n=2,733)	Level 1	1,597 (**58.4**)	2,308 (**84.4**)	1,635 (**59.8**)	524 (19.2)	1,361 (**49.8**)			
			Level 2	1,121 (41.0)	392 (14.3)	924 (33.8)	1,850 (**67.7**)	1,218 (44.6)			
			Level 3	15 (0.5)	33 (1.2)	174 (6.4)	359 (13.1)	154 (5.6)	**-**	0.81 (0.14)	65.6 (18.3)
		Juvenile rheumatoid diseases (n=1,872)	Level 1	982 (**52.5**)	1,513 (**80.8**)	1,067 (**57.0**)	287 (15.3)	961 (**51.3**)			
			Level 2	880 (47.0)	324 (17.3)	715 (38.2)	1,281 (**68.4**)	814 (43.5)			
			Level 3	10 (0.5)	35 (1.9)	90 (4.8)	304 (16.2)	97 (5.2)	**-**	0.80 (0.14)	64.3 (18.6)
		Vasculitis (n=754)	Level 1	456 (**60.5**)	675 (**89.5**)	503 (**66.7**)	179 (23.7)	402 (**53.3**)			
			Level 2	297 (39.4)	68 (9.0)	219 (29.0)	508 (**67.4**)	326 (43.2)			
			Level 3	1 (0.1)	11 (1.5)	32 (4.2)	67 (8.9)	26 (3.4)	**-**	0.84 (0.12)	68.4 (19.7)
		Other arthritis (n=2,996)	Level 1	1,498 (**50.0**)	2,497 (**83.3**)	1,752 (**58.5**)	322 (10.7)	1,637 (**54.6**)			
			Level 2	1,487 (49.6)	481 (16.1)	1,111 (37.1)	2,245 (**74.9**)	1,198 (40.0)			
			Level 3	11 (0.4)	18 (0.6)	133 (4.4)	429 (14.3)	161 (5.4)	**-**	0.81 (0.13)	64.5 (17.5)
**General population data** (n=49,169)			Level 1	44,279 (**90.1**)	48,371 (**98.4**)	44,848 (**91.2**)	24,946 (**50.8**)	32,721 (**66.5**)			
			Level 2	4,840 (9.8)	600 (1.2)	3,785 (7.7)	22,185 (45.1)	15,126 (30.8)			
			Level 3	50 (0.1)	198 (0.4)	536 (1.1)	2,038 (4.1)	1,322 (2.7)	79.5 (18.3)	0.91 (0.09)	78.6 (12.9)

*EQ VAS calculated based on a subset of the data set with compete data on EQ VAS; Proportions rounded off to one decimal using MS Excel

** The value sets used to calculate EQ-5D index are based on the EQ-5D-3L TTO and VAS value sets (Burström K, Sun S, Gerdtham U-G, *et al.* Swedish experience-based value sets for EQ-5D health states. *Qual Life Res* 2014;**23**:431–42.)

Table S7: Prevalence of reported problems on EQ-5D-3L by diagnosis and mean EQ VAS, and EQ-5D indices (TTO, VAS) among patients with complete data at 1-year follow-up regardless of baseline status and in the general population

	Patient group in NQRs	Diagnosis	Severity level	Mobility	Self-care	Usual activities	Pain/ discomfort	Anxiety/ depression	Mean (SD)		
				n (%)	n (%)	n (%)	n (%)	n (%)	EQ VAS*	EQ-5D index (TTO)**	EQ-5D index (VAS)**
Intervention-based registers	**Spine**	Disc hernia (n=16,196)	Level 1	11,627 (**71.8**)	14,915 (**92.1**)	11,399 (**70.4**)	4,476 (27.6)	10,272 (**63.4**)			
			Level 2	4,514 (27.9)	1189 (7.3)	4223 (26.1)	10,091 (**62.3**)	5,137 (31.7)			
			Level 3	55 (0.3)	92 (0.6)	574 (3.5)	1,629 (10.1)	787 (4.9)	71.5 (21.5)	0.85 (0.13)	71.0 (18.3)
		Other spinal conditions (n=1,828)	Level 1	1,004 (**54.9**)	1,641 (**89.8**)	1,119 (**61.2**)	392 (21.4)	1,087 (**59.5**)			
			Level 2	816 (44.6)	172 (9.4)	599 (32.8)	1,114 (**60.9**)	639 (35.0)			
			Level 3	8 (0.4)	15 (0.8)	110 (6.0)	322 (17.6)	102 (5.6)	66.0 (23.3)	0.82 (0.14)	66.0 (19.7)
		Segmental instability (n=4,670)	Level 1	3,323 (**71.2**)	4,222 (**90.4**)	2,845 (**60.9**)	1,049 (22.5)	2,734 (**58.5**)			
			Level 2	1,331 (28.5)	429 (9.2)	1,505 (32.2)	2,889 (**61.9**)	1,666 (35.7)			
			Level 3	16 (0.3)	19 (0.4)	320 (6.9)	732 (15.7)	270 (5.8)	68.1 (23.3)	0.83 (0.14)	67.8 (19.7)
		Spinal stenosis (n=32,840)	Level 1	14,943 (45.5)	29,720 (**90.5**)	20,679 (**63.0**)	6,529 (19.9)	20,126 (**61.3**)			
			Level 2	17,793 (**54.2**)	2,845 (8.7)	10,533 (32.1)	21,040 (**64.1**)	11,360 (34.6)			
			Level 3	104 (0.3)	275 (0.8)	1,628 (5.0)	5,271 (16.1)	1,354 (4.1)	65.3 (22.8)	0.82 (0.14)	66.0 (18.8)
		Spondylolysis (n=2,763)	Level 1	1,851(**67.0**)	2,558 (**92.6**)	1,783 (**64.5**)	640 (23.2)	1,690 (**61.2**)			
			Level 2	909 (32.9)	197 (7.1)	852 (30.8)	1,740 (**63.0**)	921 (33.3)			
			Level 3	3 (0.1)	8 (0.3)	128 (4.6)	383 (13.9)	152 (5.5)	68.9 (22.5)	0.84 (0.14)	68.6 (19.1)
	**Hip**	Avascular necrosis (n=2,149)	Level 1	1,069 (49.7)	1,811 (**84.3**)	1,394 (**64.9**)	789 (36.7)	1,415 (**65.8**)			
			Level 2	1,071 (**49.8**)	303 (14.1)	621 (28.9)	1,211 (**56.4**)	676 (31.5)			
			Level 3	9 (0.4)	35 (1.6)	134 (6.2)	149 (6.9)	58 (2.7)	71.0 (22.7)	0.84 (0.13)	69.5 (18.0)
		Childhood hip disorder (n=2,236)	Level 1	1,522 (**68.1**)	2,021 (**90.4**)	1,726 (**77.2**)	1,174 (**52.5**)	1,742 (**77.9**)			
			Level 2	712 (31.8)	198 (8.9)	442 (19.8)	965 (43.2)	444 (19.9)			
			Level 3	2 (0.1)	17 (0.8)	68 (3.0)	97 (4.3)	50 (2.2)	78.9 (19.5)	0.88 (0.12)	75.9 (16.5)
		Hip osteoarthritis (n=101,136)	Level 1	61,928 (**61.2**)	93,392 (**92.3**)	78,639 (**77.8**)	45,889 (45.4)	79,190 (**78.3**)			
			Level 2	39,045 (38.6)	7,113 (7.0)	20,384 (20.2)	50,826 (**50.3**)	20,409 (20.2)			
			Level 3	163 (0.2)	631 (0.6)	2,113 (2.1)	4,421 (4.4)	1,537 (1.5)	76.5 (20.1)	0.88 (0.11)	75.1 (15.6)
		Inflammatory joint disorder (n=1,540)	Level 1	666 (43.2)	1,157 (**75.1**)	866 (**56.2**)	440 (28.6)	1,035 (**67.2**)			
			Level 2	865 (**56.2**)	335 (21.8)	583 (37.9)	956 (**62.1**)	468 (30.4)			
			Level 3	9 (0.6)	48 (3.1)	91 (5.9)	144 (9.4)	37 (2.4)	67.8 (22.1)	0.82 (0.13)	67.1 (17.8)
	**Knee**	Knee osteoarthritis (n=18,231)	Level 1	11,460 (**62.9**)	17,338 (**95.1**)	14,143 (**77.6**)	6,610 (36.3)	14,276 (**78.3**)			
			Level 2	6,740 (37.0)	788 (4.3)	3,768 (20.7)	10,668 (**58.5**)	3,639 (20.0)			
			Level 3	31 (0.2)	105 (0.6)	320 (1.8)	953 (5.2)	316 (1.7)	76.1 (19.5)	0.88 (0.11)	74.5 (15.4)
		Knee rheumatoid arthritis (n=372)	Level 1	140 (37.6)	303 (**81.5**)	208 (**55.9**)	79 (21.2)	245 (**65.9**)			
			Level 2	230 (**61.8**)	60 (16.1)	146 (39.2)	254 (**68.3**)	118 (31.7)			
			Level 3	2 (0.5)	9 (2.4)	18 (4.8)	39 (10.5)	9 (2.4)	67.1 (21.0)	0.81 (0.13)	65.8 (17.4)

Intervention-based registers	**Ankle**	Other (n=152)	Level 1	41 (27.0)	130 (**85.5**)	82 (**53.9**)	37 (24.3)	92 (**60.5**)			
			Level 2	108 (**71.1**)	21 (13.8)	64 (42.1)	98 (**64.5**)	55 (36.2)			
			Level 3	3 (2.0)	1 (0.7)	6 (3.9)	17 (11.2)	5 (3.3)	65.0 (21.6)	0.80 (0.13)	64.1 (17.9)
		Posttraumatic osteoarthritis (n=510)	Level 1	178 (34.9)	472 (**92.5**)	314 (**61.6**)	120 (23.5)	338 (**66.3**)			
			Level 2	329 (**64.5**)	34 (6.7)	165 (32.4)	328 (**64.3**)	140 (27.5)			
			Level 3	3 (0.6)	4 (0.8)	31 (6.1)	62 (12.2)	32 (6.3)	68.6 (21.3)	0.81 (0.14)	65.8 (19.1)
		Primary osteoarthritis (n=388)	Level 1	166 (42.8)	364 (**93.8**)	277 (**71.4**)	121 (31.2)	302 (**77.8**)			
			Level 2	221 (**57.0**)	22 (5.7)	95 (24.5)	222 (**57.2**)	80 (20.6)			
			Level 3	1 (0.3)	2 (0.5)	16 (4.1)	45 (11.6)	6 (1.5)	72.4 (18.6)	0.85 (0.12)	70.3 (17.6)
		Rheumatoid arthritis (n=117)	Level 1	32 (27.4)	94 (**80.3**)	60 (**51.3**)	25 (21.4)	73 (**62.4**)			
			Level 2	83 (**70.9**)	21 (17.9)	51 (43.6)	78 (**66.7**)	40 (34.2)			
			Level 3	2 (1.7)	2 (1.7)	6 (5.1)	14 (12.0)	4 (3.4)	64.3 (19.9)	0.80 (0.13)	63.7 (17.4)
	**Cruciate ligament**	Cruciate ligament injury (n=15,731)	Level 1	13,492 (**85.8**)	15,534 (**98.7**)	12,269 (**78.0**)	5,356 (34.0)	10,105 (**64.2**)			
			Level 2	2,230 (14.2)	142 (0.9)	3,207 (20.4)	9,812 (**62.4**)	5,085 (32.3)			
			Level 3	9 (0.1)	55 (0.3)	255 (1.6)	563 (3.6)	541 (3.4)	74.2 (20.1)	0.88 (0.11)	75.2 (15.0)
	**BOA**	BOA hip (n=3,437)	Level 1	1,726 (**50.2**)	3,212 (**93.5**)	2,659 (**77.4**)	258 (7.5)	2,301 (**66.9**)			
			Level 2	1,708 (49.7)	214 (6.2)	742 (21.6)	2,887 (**84.0**)	1,093 (31.8)			
			Level 3	3 (0.1)	11 (0.3)	36 (1.0)	292 (8.5)	43 (1.3)	68.0 (18.8)	0.85 (0.10)	69.8 (14.4)
		BOA knee (n=10,248)	Level 1	5,418 (**52.9**)	9,926 (**96.9**)	8,258 (**80.6**)	1,015 (9.9)	7,297 (**71.2**)			
			Level 2	4,824 (47.1)	272 (2.7)	1,906 (18.6)	8,437 (**82.3**)	2,830 (27.6)			
			Level 3	6 (0.1)	50 (0.5)	84 (0.8)	796 (7.8)	121 (1.2)	70.7 (18.6)	0.86 (0.10)	71.2 (14.0)
	**Fracture**	Ankle and foot (n=3,898)	Level 1	2,832 (**72.7**)	3,740 (**95.9**)	3,248 (**83.3**)	1,917 (**49.2**)	3,139 (**80.5**)			
			Level 2	1,055 (27.1)	124 (3.2)	569 (14.6)	1,820 (46.7)	684 (17.5)			
			Level 3	11 (0.3)	34 (0.9)	81 (2.1)	161 (4.1)	75 (1.9)	81.0 (19.0)	0.90 (0.11)	77.3 (15.3)
		Elbow and forearm (n=14,576)	Level 1	12,585 (**86.3**)	13,594 (**93.3**)	12,045 (**82.6**)	6,886 (47.2)	11,816 (**81.1**)			
			Level 2	1,944 (13.3)	828 (5.7)	2,155 (14.8)	7,270 (**49.9**)	2,547 (17.5)			
			Level 3	47 (0.3)	154 (1.1)	376 (2.6)	420 (2.9)	213 (1.5)	81.3 (18.5)	0.90 (0.10)	78.7 (13.6)
		Hip and femur (n=8,858)	Level 1	2,521 (28.5)	5,926 (**66.9**)	4,424 (**49.9**)	2,403 (27.1)	5,072 (**57.3**)			
			Level 2	5,914 (**66.8**)	2,027 (22.9)	2,791 (31.5)	5,770 (**65.1**)	3,407 (38.5)			
			Level 3	423 (4.8)	905 (10.2)	1,643 (18.5)	685 (7.7)	379 (4.3)	64.0 (23.5)	0.78 (0.14)	62.2 (18.8)
		Knee and lower leg (n=11,360)	Level 1	7,279 (**64.1**)	10,644 (**93.7**)	8,896 (**78.3**)	4,384 (38.6)	8,690 (**76.5**)			
			Level 2	3,981 (35.0)	527 (4.6)	2,055 (18.1)	6,507 (**57.3**)	2,424 (21.3)			
			Level 3	100 (0.9)	189 (1.7)	409 (3.6)	469 (4.1)	246 (2.2)	78.2 (19.4)	0.88 (0.12)	74.5 (16.1)
		Lumbar, spine and pelvis (n=1,232)	Level 1	583 (47.3)	945 (**76.7**)	731 (**59.3**)	434 (35.2)	794 (**64.4**)			
			Level 2	626 (**50.8**)	208 (16.9)	332 (26.9)	696 (**56.5**)	390 (31.7)			
			Level 3	23 (1.9)	79 (6.4)	169 (13.7)	102 (8.3)	48 (3.9)	68.7 (23.4)	0.82 (0.15)	67.1 (19.7)
		Shoulder and upper arm (n=8,273)	Level 1	6,566 (**79.4**)	7,041 (**85.1**)	6,141 (**74.2**)	3,055 (36.9)	6,162 (**74.5**)			
			Level 2	1,652 (20.0)	1,035 (12.5)	1,727 (20.9)	4,834 (**58.4**)	1,925 (23.3)			
			Level 3	55 (0.7)	197 (2.4)	405 (4.9)	384 (4.6)	186 (2.2)	77.0 (20.3)	0.88 (0.12)	74.9 (15.9)
		Unspecified part of trunk, extremities or other body region (n=860)	Level 1	513 (**59.7**)	719 (**83.6**)	526 (**61.2**)	190 (22.1)	552 (**64.2**)			
			Level 2	329 (38.3)	111 (12.9)	248 (28.8)	565 (**65.7**)	270 (31.4)			
			Level 3	18 (2.1)	30 (3.5)	86 (10.0)	105 (12.2)	38 (4.4)	69.9 (22.0)	0.83 (0.15)	67.6 (19.6)
		Wrist and hand (n=4,532)	Level 1	4,069 (**89.8**)	4,301 (**94.9**)	3,775 (**83.3**)	2,171 (47.9)	3,745 (82.6)			
			Level 2	445 (9.8)	189 (4.2)	667 (14.7)	2,225 (49.1)	722 (15.9)			
			Level 3	18 (0.4)	42 (0.9)	90 (2.0)	136 (3.0)	65 (1.4)	82.9 (17.7)	0.91 (0.09)	79.4 (13.2)

Diagnosis-based registers	**Heart failure**	Heart failure (n=1,849)	Level 1	1,094 (**59.2**)	1,680 (**90.9**)	1,338 (**72.4**)	911 (**49.3**)	1,157 (**62.6**)			
			Level 2	748 (40.5)	153 (8.3)	458 (24.8)	819 (44.3)	645 (34.9)			
			Level 3	7 (0.4)	16 (0.9)	53 (2.9)	119 (6.4)	47 (2.5)	66.7 (19.5)	0.86 (0.12)	72.3 (16.3)
	**Respiratory failure**	COPD (n=895)	Level 1	160 (17.9)	472 (**52.7**)	175 (19.6)	208 (23.2)	315 (35.2)			
			Level 2	693 (**77.4**)	344 (38.4)	460 (**51.4**)	541 (**60.4**)	488 (**54.5**)			
			Level 3	42 (4.7)	79 (8.8)	260 (29.1)	146 (16.3)	92 (10.3)	48.1 (20.4)	0.70 (0.14)	51.7 (17.5)
		Lung fibrosis (n=149)	Level 1	25 (16.8)	72 (**48.3**)	32 (21.5)	43 (28.9)	68 (**45.6**)			
			Level 2	118 (**79.2**)	66 (44.3)	61 (**40.9**)	86 (**57.7**)	68 (**45.6**)			
			Level 3	6 (4.0)	11 (7.4)	56 (37.6)	20 (13.4)	13 (8.7)	48.5 (19.7)	0.71 (0.14)	53.1 (18.2)
	**Psoriasis**	Psoriasis vulgaris (n=1,880)	Level 1	1,612 (**85.7**)	1,829 (**97.3**)	1,687 (**89.7**)	1,004 (**53.4**)	1,236 (**65.7**)			
			Level 2	267 (14.2)	48 (2.6)	172 (9.1)	806 (42.9)	573 (30.5)			
			Level 3	1 (0.1)	3 (0.2)	21 (1.1)	70 (3.7)	71 (3.8)	**-**	0.90 (0.10)	78.0 (13.9)
		Psoriasis vulgaris + anthropathic (n=663)	Level 1	406 (**64.3**)	584 (**92.6**)	497 (**78.8**)	161 (25.5)	358 (**56.7**)			
			Level 2	223 (35.3)	41 (6.5)	118 (18.7)	405 (**64.2**)	236 (37.4)			
			Level 3	2 (0.3)	6 (1.0)	16 (2.5)	65 (10.3)	37 (5.9)	-	0.85 (0.12)	70.3 (17.1)
		Psoriasis unspecified (n=363)	Level 1	283 (**78.0**)	351 (**96.7**)	316 (**87.1**)	160 (44.1)	244 (**67.2**)			
			Level 2	80 (22.0)	11 (3.0)	45 (12.4)	183 (**50.4**)	104 (28.7)			
			Level 3	1,612 (0.0)	1 (0.3)	2 (0.6)	20 (5.5)	15 (4.1)	-	0.89 (0.11)	76.1 (14.9)
	**Rheumatology**	Rheumatoid arthritis (n=18,141)	Level 1	9,904 (54.6)	15,143 (83.5)	12,032 (66.3)	3,199 (17.6)	11,123 (61.3)			
			Level 2	8,165 (45.0)	2,763 (15.2)	5,506 (30.4)	12,974 (71.5)	6,294 (34.7)			
			Level 3	72 (0.4)	235 (1.3)	603 (3.3)	1,968 (10.8)	724 (4.0)	**-**	0.83 (0.13)	67.9 (17.4)
		Spondyloarthropathy (n=8,916)	Level 1	5,142 (**57.7**)	7,578 (**85.0**)	5,582 (**62.6**)	1,293 (14.5)	4,813 (**54.0**)			
			Level 2	3,757 (42.1)	1,270 (14.2)	2,973 (33.3)	6,277 (**70.4**)	3,533 (39.6)			
			Level 3	17 (0.2)	68 (0.8)	361 (4.0)	1,346 (15.1)	570 (6.4)	**-**	0.82 (0.14)	65.7 (18.8)
		Inflammatory systemic disorder (n1,654)	Level 1	984 (**59.5**)	1,425 (**86.2**)	1,029 (**62.2**)	350 (21.2)	849 (**51.3**)			
			Level 2	664 (40.1)	214 (12.9)	540 (32.6)	1,109 (**67.0**)	705 (42.6)			
			Level 3	6 (0.4)	15 (0.9)	85 (5.1)	195 (11.8)	100 (6.0)	**-**	0.82 (0.13)	66.4 (18.2)
		Juvenile rheumatoid diseases (n=1,112)	Level 1	605 (**54.4**)	894 (**80.4**)	670 (**60.3**)	204 (18.3)	580 (**52.2**)			
			Level 2	502 (45.1)	202 (18.2)	387 (34.8)	747 (**67.2**)	470 (42.3)			
			Level 3	5 (0.4)	16 (1.4)	55 (4.9)	161 (14.5)	62 (5.6)	**-**	0.81 (0.14)	65.3 (18.9)
		Vasculitis (n=413)	Level 1	242 (**58.6**)	375 (**90.8**)	278 (**67.3**)	92 (22.3)	242 (**58.6**)			
			Level 2	170 (41.2)	31 (7.5)	125 (30.3)	281 (**68.0**)	156 (37.8)			
			Level 3	1 (0.2)	7 (1.7)	10 (2.4)	40 (9.7)	15 (3.6)	**-**	0.84 (0.12)	68.7 (16.6)
		Other arthritis (n=1,728)	Level 1	1,004 (**58.1**)	1,521 (**88.0**)	1,185 (**68.6**)	303 (17.5)	1,066 (**61.7**)			
			Level 2	721 (41.7)	193 (11.2)	493 (28.5)	1,212 (**70.1**)	595 (34.4)			
			Level 3	3 (0.2)	14 (0.8)	50 (2.9)	213 (12.3)	67 (3.9)	**-**	0.84 (0.13)	68.4 (17.4)
**General population data** (n=49,169)			Level 1	44,279 (**90.1**)	48,371 (**98.4**)	44,848 (**91.2**)	24,946 (**50.8**)	32,721 (**66.5**)			
			Level 2	4,840 (9.8)	600 (1.2)	3,785 (7.7)	22,185 (45.1)	15,126 (30.8)			
			Level 3	50 (0.1)	198 (0.4)	536 (1.1)	2,038 (4.1)	1,322 (2.7)	79.5 (18.3)	0.91 (0.09)	78.6 (12.9)

*EQ VAS calculated based on a subset of the data set with compete data on EQ VAS; Proportions rounded off to one decimal using MS Excel

** The value sets used to calculate EQ-5D index are based on the EQ-5D-3L TTO and VAS value sets (Burström K, Sun S, Gerdtham U-G, *et al.* Swedish experience-based value sets for EQ-5D health states. *Qual Life Res* 2014;**23**:431–42.)

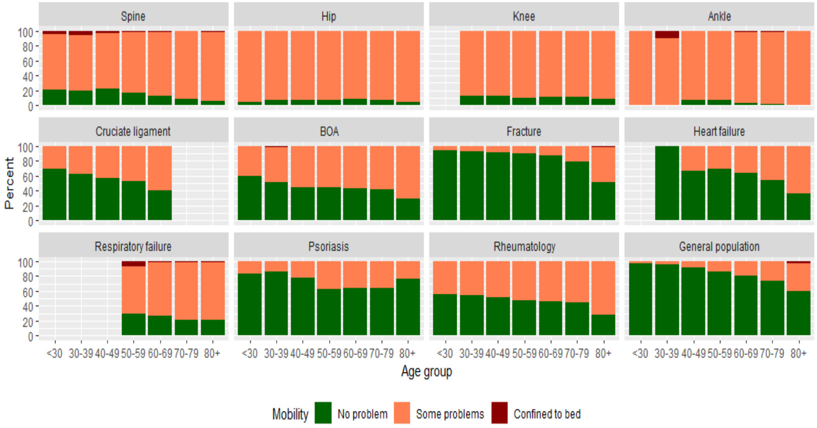
Spine
Hip
Knee
Ankle
Cruciate ligament
BOA
Fracture
Heart failure
Respiratory failure
Psoriasis
Rheumatology
General population
Percent
Age group
<30 30-39 40-49 50-59 60-69 70-79 80+
Mobility
No problem
Some problems
Confined to bed

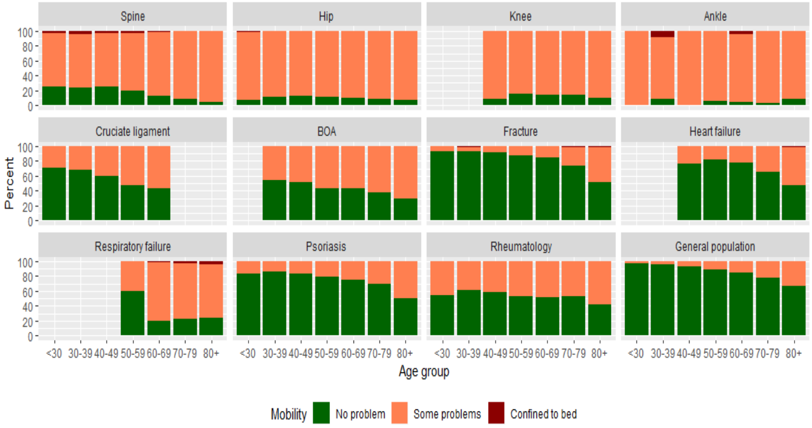
Spine
Hip
Knee
Ankle
Cruciate ligament
BOA
Fracture
Heart failure
Respiratory failure
Psoriasis
Rheumatology
General population
Percent
Age group
<30 30-39 40-49 50-59 60-69 70-79 80+
Mobility
No problem
Some problems
Confined to bed

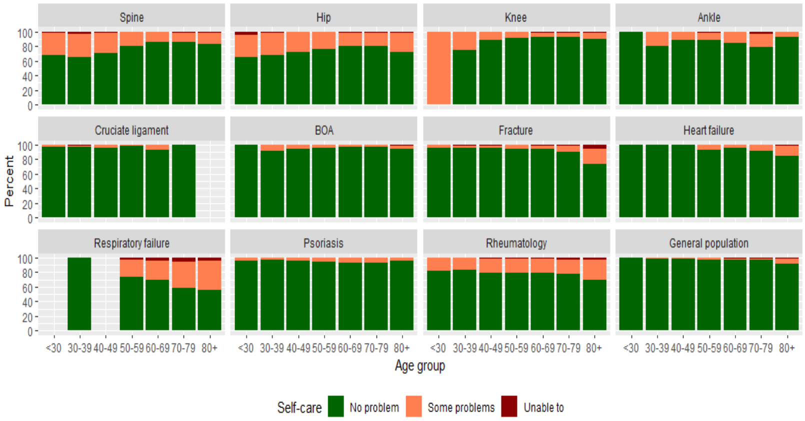
Spine
Hip
Knee
Ankle
Cruciate ligament
BOA
Fracture
Heart failure
Respiratory failure
Psoriasis
Rheumatology
General population
Percent
Age group
<30 30-39 40-49 50-59 60-69 70-79 80+
Self-care
No problem
Some problems
Unable to

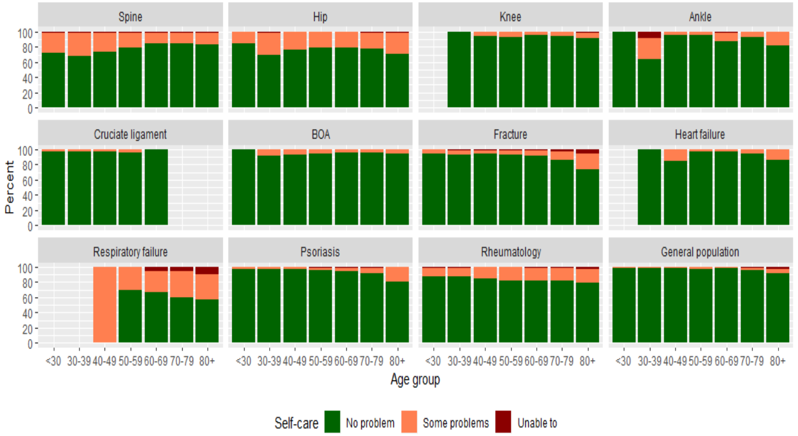
Spine
Hip
Knee
Ankle
Cruciate ligament
BOA
Fracture
Heart failure
Respiratory failure
Psoriasis
Rheumatology
General population
Percent
Age group
<30 30-39 40-49 50-59 60-69 70-79 80+
Self-care
No problem
Some problems
Unable to

Women Men

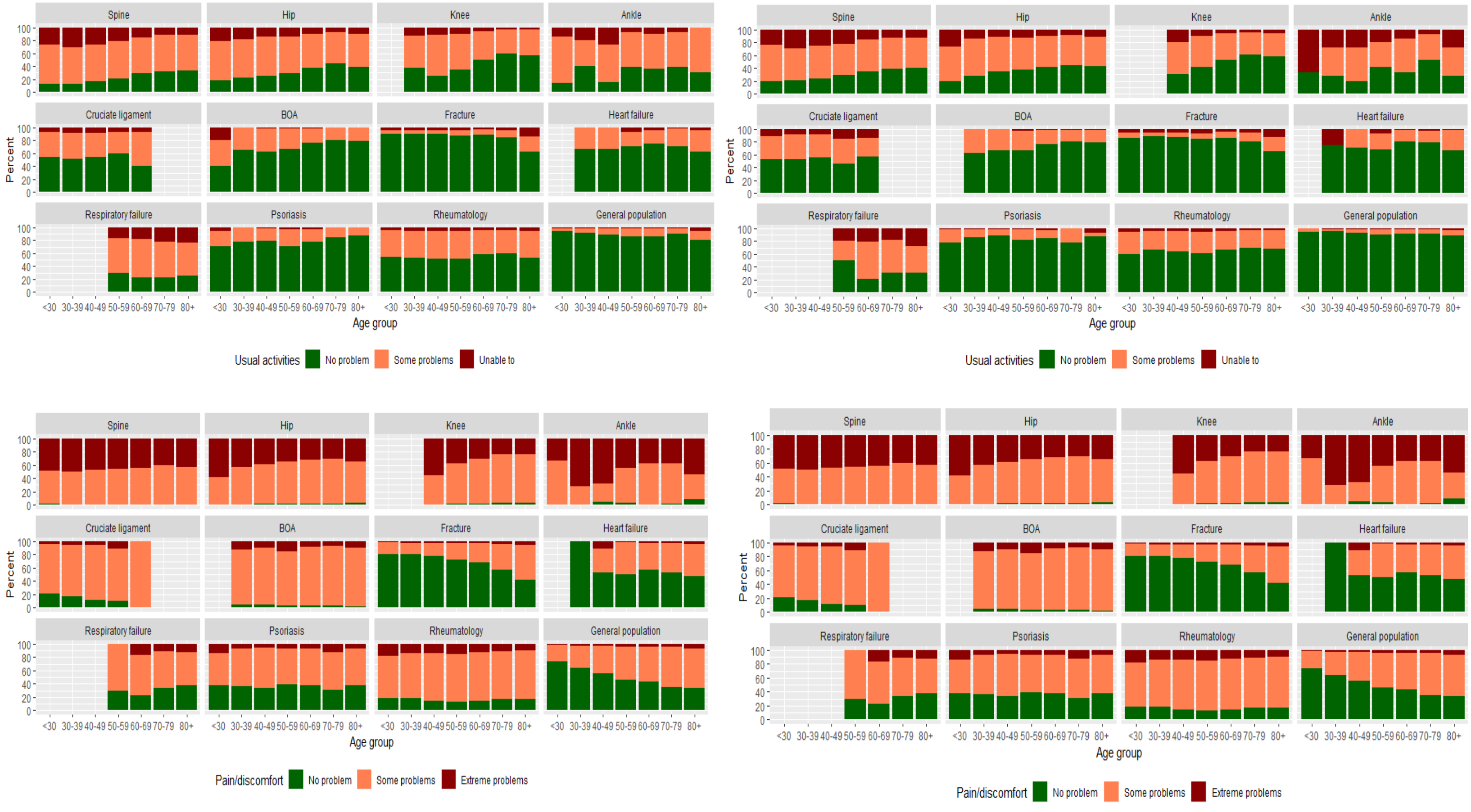
Spine
Hip
Knee
Ankle
Cruciate ligament
BOA
Fracture
Heart failure
Respiratory failure
Psoriasis
Rheumatology
General population
Percent
Age group
<30 30-39 40-49 50-59 60-69 70-79 80+
Usual activities
No problem
Some problems
Unable to
Pain/discomfort
No problem
Some problems
Extreme problems

Women

Men

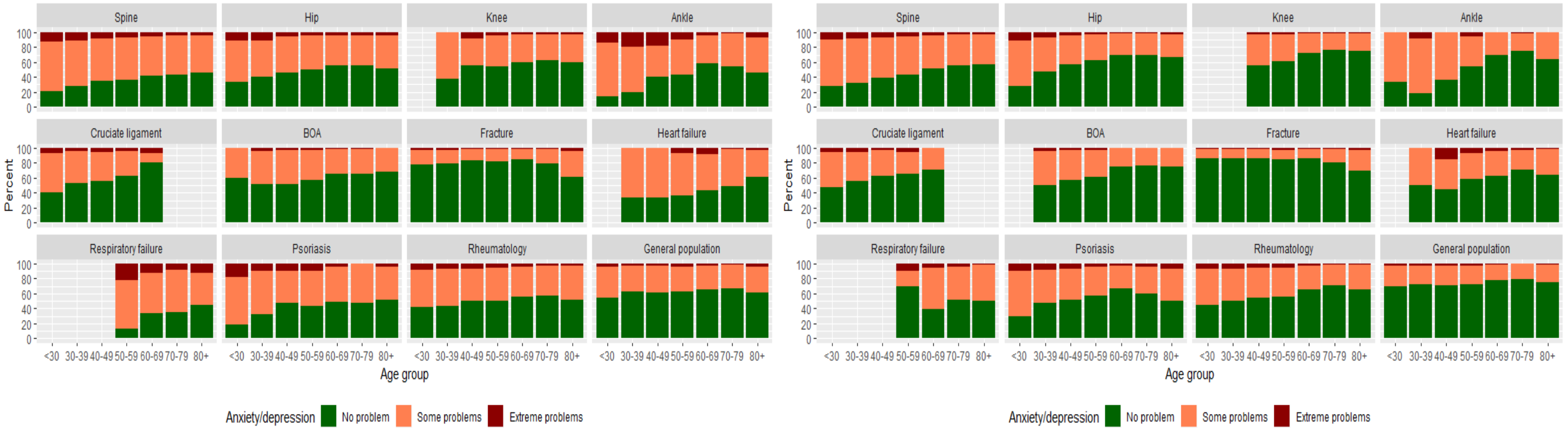

Women   Men

Figure S1: Problems reported on the EQ-5D-3L dimensions among patients in the different registers at baseline and the general population by age group and sex

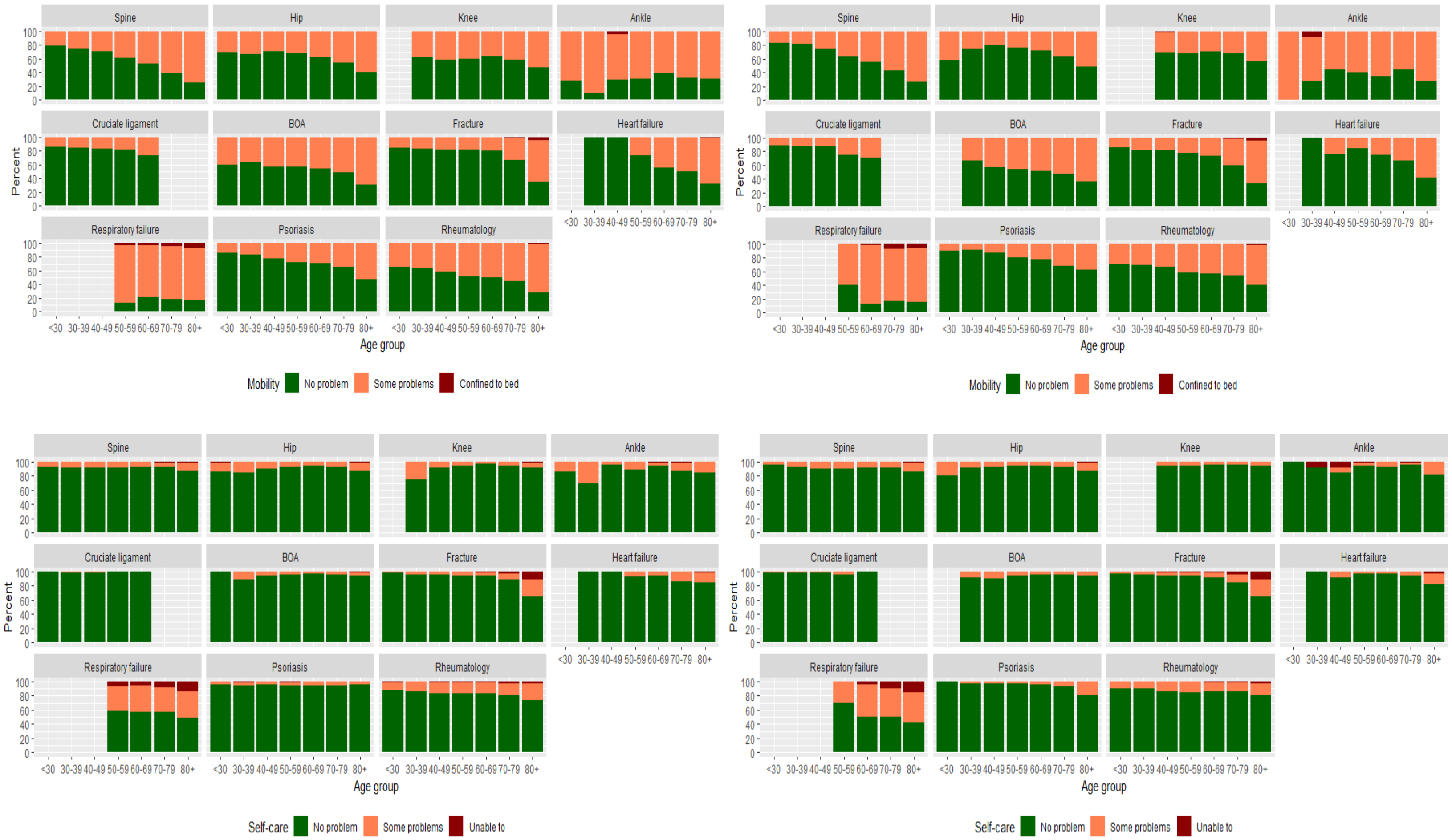

Women Men

Teni FS, *et al. BMJ Open* 2022; 12:e048176. doi: 10.1136/bmjopen-2020-048176

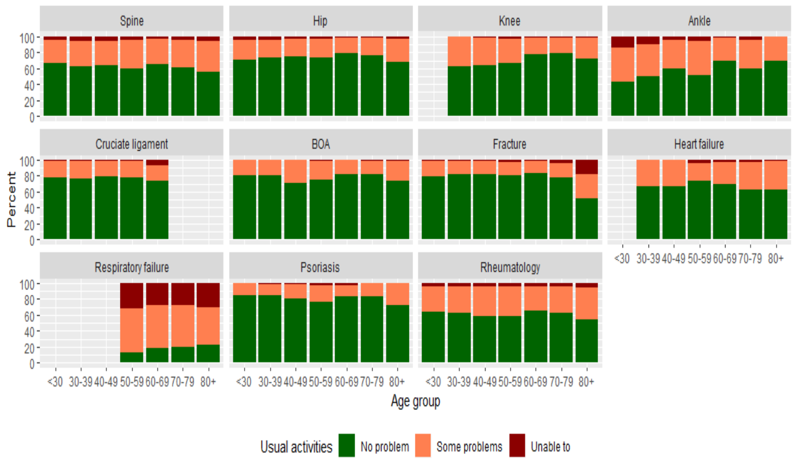
Spine
Hip
Knee
Ankle
Cruciate ligament
BOA
Fracture
Heart failure
Respiratory failure
Psoriasis
Rheumatology
Percent
Age group
Usual activities
No problem
Some problems
Unable to

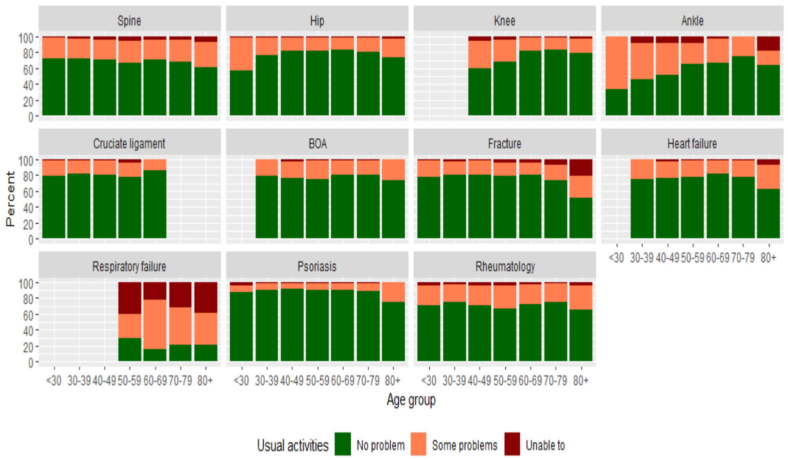
Spine
Hip
Knee
Ankle
Cruciate ligament
BOA
Fracture
Heart failure
Respiratory failure
Psoriasis
Rheumatology
Percent
Age group
Usual activities
No problem
Some problems
Unable to

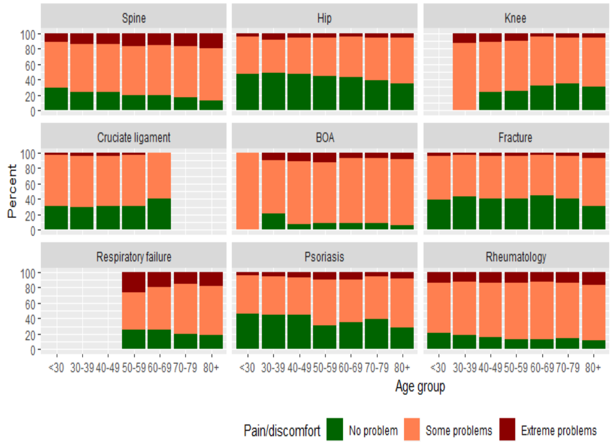
Spine
Hip
Knee
Ankle
Cruciate ligament
BOA
Fracture
Heart failure
Respiratory failure
Psoriasis
Rheumatology
Percent
Age group
Pain/discomfort
No problem
Some problems
Extreme problems

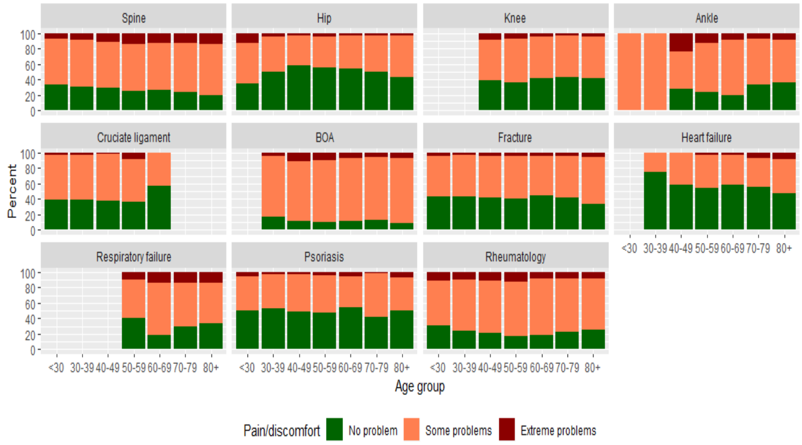
Spine
Hip
Knee
Ankle
Cruciate ligament
BOA
Fracture
Heart failure
Respiratory failure
Psoriasis
Rheumatology
Percent
Age group
Pain/discomfort
No problem
Some problems
Extreme problems

Women

Men

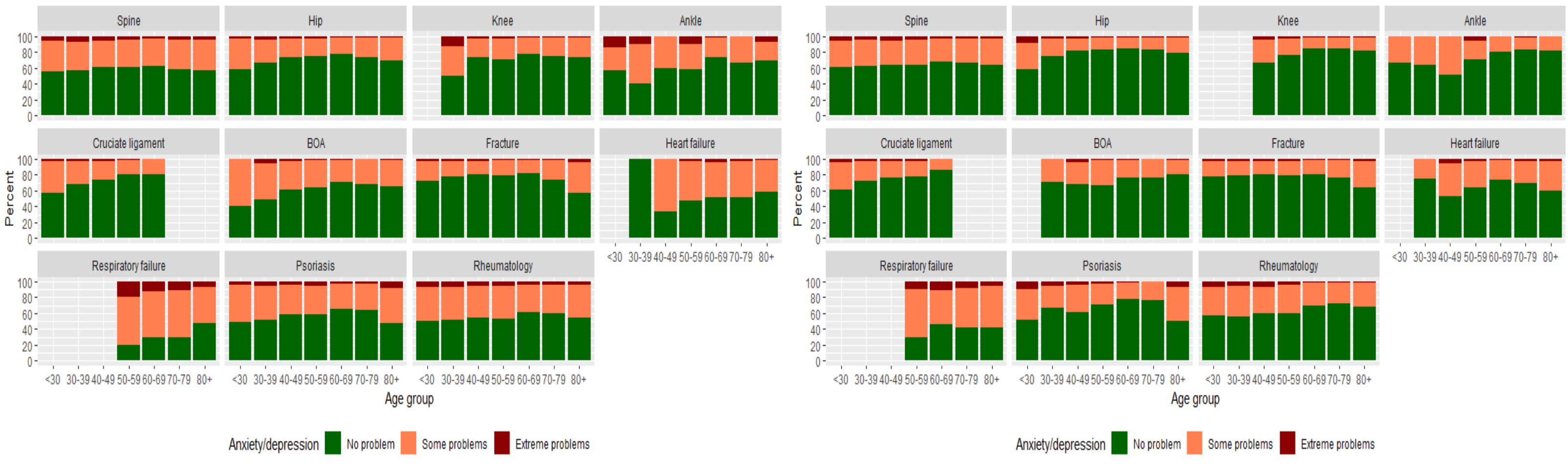

Women Men

Figure S2: Problems reported on the EQ-5D-3L dimensions among patients in the different registers at 1-year follow-up by age group and sex

Teni FS, *et al. BMJ Open* 2022; 12:e048176. doi: 10.1136/bmjopen-2020-048176
