# Supplementary material for: Longitudinal study of patients’ health-related quality of life using EQ-5D-3L in 11 Swedish National Quality Registers
Source: BMJ Open. 2022 Jan 6;12(1):e048176. doi: 10.1136/bmjopen-2020-048176 (PMC8739074; doi:10.1136/bmjopen-2020-048176)
Supplement: Author's manuscript [file bmjopen-2020-048176.draft_revisions.pdf]

BMJ Open is committed to open peer review. As part of this commitment we make the peer review history of every article we publish publicly available.

When an article is published we post the peer reviewers' comments and the authors' responses online. We also post the versions of the paper that were used during peer review. These are the versions that the peer review comments apply to.

The versions of the paper that follow are the versions that were submitted during the peer review process. They are not the versions of record or the final published versions. They should not be cited or distributed as the published version of this manuscript.

BMJ Open is an open access journal and the full, final, typeset and author-corrected version of record of the manuscript is available on our site with no access controls, subscription charges or pay-per-view fees (http://bmjopen.bmj.com).

If you have any questions on BMJ Open's open peer review process please email info.bmjopen@bmj.com

BMJ Open

A longitudinal study of patients' health-related quality of life using EQ-5D-3L in 11 Swedish National Quality Registers

Journal:	*BMJ Open*
Manuscript ID	bmjopen-2020-048176
Article Type:	Original research
Date Submitted by the Author:	21-Dec-2020
Complete List of Authors:	Teni, Fitsum; Karolinska Institutet, Health Outcomes and Economic Evaluation Research Group, Stockholm Centre for Healthcare Ethics, Department of Learning, Informatics, Management and Ethics<br>Rolfson, O; Sahlgrenska Academy, University of Gothenburg, Department of Orthopaedics, Institute of Clinical Sciences; Swedish Hip Arthroplasty Register<br>Devlin, Nancy; The University of Melbourne School of Population and Global Health, Health Economics; Office of Health Economics<br>Parkin, David; Office of Health Economics; City University of London<br>Nauclér, Emma; Swedish Hip Arthroplasty Register<br>Burström, Kristina; Karolinska Institutet, Health Outcomes and Economic Evaluation Research Group, Stockholm Centre for Healthcare Ethics, Department of Learning, Informatics, Management and Ethics; Region Stockholm, Health Care Services<br>the Swedish Quality Register (SWEQR) Study Group, na
Keywords:	ORTHOPAEDIC & TRAUMA SURGERY, HEALTH ECONOMICS, INTERNAL MEDICINE

SCHOLARONE™
Manuscripts

I, the Submitting Author has the right to grant and does grant on behalf of all authors of the Work (as defined in the below author licence), an exclusive licence and/or a non-exclusive licence for contributions from authors who are: i) UK Crown employees; ii) where BMJ has agreed a CC-BY licence shall apply, and/or iii) in accordance with the terms applicable for US Federal Government officers or employees acting as part of their official duties; on a worldwide, perpetual, irrevocable, royalty-free basis to BMJ Publishing Group Ltd ("BMJ") its licensees and where the relevant Journal is co-owned by BMJ to the co-owners of the Journal, to publish the Work in this journal and any other BMJ products and to exploit all rights, as set out in our licence.

The Submitting Author accepts and understands that any supply made under these terms is made by BMJ to the Submitting Author unless you are acting as an employee on behalf of your employer or a postgraduate student of an affiliated institution which is paying any applicable article publishing charge ("APC") for Open Access articles. Where the Submitting Author wishes to make the Work available on an Open Access basis (and intends to pay the relevant APC), the terms of reuse of such Open Access shall be governed by a Creative Commons licence – details of these licences and which Creative Commons licence will apply to this Work are set out in our licence referred to above.

Other than as permitted in any relevant BMJ Author's Self Archiving Policies, I confirm this Work has not been accepted for publication elsewhere, is not being considered for publication elsewhere and does not duplicate material already published. I confirm all authors consent to publication of this Work and authorise the granting of this licence.

For peer review only

A longitudinal study of patients' health-related quality of life using EQ-5D-3L in 11 Swedish National Quality Registers

Fitsum Sebsibe Teni[1*] (Doctoral student, ORCID 0000-0002-6182-499X, fitsum.teni@ki.se),

Ola Rolfson[1,2,3] (Professor, ORCID 0000-0001-6534-1242, ola.rolfson@vgregion.se),

Nancy Devlin[4,5] (Professor, ORCID 0000-0002-1561-5361, nancy.devlin@unimelb.edu.au),

David Parkin[5,6] (Senior Visiting Fellow, ORCID 0000-0002-9990-8208, d.parkin@city.ac.uk),

Emma Nauclér[3] (Senior Statistician, ORCID 0000-0003-1079-5655, emma.naucler@vgregion.se),

Kristina Burström[1,7,8] (Associate Professor, ORCID 0000-0001-9996-4317, kristina.burstrom@ki.se) and

The Swedish Quality Register (SWEQR) Study Group

Authors' affiliations

[1] Health Outcomes and Economic Evaluation Research Group, Stockholm Centre for Healthcare Ethics, Department of Learning, Informatics, Management and Ethics, Karolinska Institutet, Stockholm, Sweden.

[2] Department of Orthopaedics, Institute of Clinical Sciences, Sahlgrenska Academy, University of Gothenburg, Gothenburg, Sweden.

[3] Swedish Hip Arthroplasty Register, Gothenburg, Sweden.

[4] Centre for Health Policy, University of Melbourne, Melbourne, Australia.

[5] Office of Health Economics, London, United Kingdom.

[6] City University of London, London, United Kingdom.

[7] Equity and Health Policy Research Group, Department of Global Public Health, Karolinska Institutet, Stockholm; Sweden.

[8] Health Care Services, Region Stockholm, Stockholm, Sweden

The Swedish Quality Register (SWEQR) Study Group:

Allan Abbott (Associate Professor, Linköping University, Linköping, Sweden; ORCID 0000-0002-4318-9216; allan.abbott@liu.se),

Magnus Ekström (Associate Professor, Lund University, Lund, Sweden; ORCID 0000-0002-7227-5113; pmekstrom@gmail.com),

Magnus Forssblad (Associate Professor, Karolinska Institutet, Stockholm, Sweden; ORCID 0000-0002-5870-7477; magnus@forssblad.se),

Peter Fritzell (Associate Professor, Futurum Academy for Health and Care, Jönköping, Sweden/RKC Centre for spine surgery, Stockholm, Sweden; ORCID 0000-0002-2043-6376; peterfritzell@gmail.com),

Åsa Jonsson (RN, PhD, Ryhov County Hospital, Jönköping, Sweden; ORCID 0000-0002-3046-6333; asa.jonsson@rikssvikt.se),

Mikael Landén (Professor, University of Gothenburg, Gothenburg, Sweden; ORCID 0000-0002-4496-6451; mikael.landen@neuro.gu.se),

Michael Möller (Associate Professor, University of Gothenburg, Gothenburg, Sweden; ORCID 0000-0001-7394-6057; michael.moller@vgregion.se),

Malin Regardt (Head of Research and Development, Karolinska University Hospital, Stockholm; Karolinska Institutet, Stockholm, Sweden; ORCID 0000-0002-1908-5708; malin.regardt@sll.se, malin.regardt@ki.se),

For peer review only

For peer review only - http://bmjopen.bmj.com/site/about/guidelines.xhtml

Björn Rosengren (Professor, Lund University, Lund, Sweden; ORCID 0000-0001-5588-6927; bjorn.rosengren@med.lu.se),

Marcus Schmitt-Egenolf (Professor, Umeå University, Umeå, Sweden; ORCID 0000-0002-3858-8474; marcus.schmitt-egenolf@umu.se),

Johanna Vinblad (Project leader, Centre of Registers, Gothenburg, Sweden; ORCID 0000-0003-1842-7355; johanna.vinblad@vgregion.se),

Annette W-Dahl (Associate Professor, Lund University, Lund, Sweden; ORCID 0000-0003-4053-7358; annette.w-dahl@med.lu.se).

*Corresponding author

Fitsum Sebsibe Teni

Health Outcomes and Economic Evaluation Research Group
Stockholm Centre for Healthcare Ethics
Department of Learning, Informatics, Management and Ethics
Karolinska Institutet
SE-171 77 Stockholm, Sweden
E-mail: fitsum.teni@ki.se
Tel: +46764027138
ORCID: 0000 0002 6182 499X

For peer review only - http://bmjopen.bmj.com/site/about/guidelines.xhtml

Abstract

Objective: To compare problems reported in the five EQ-5D-3L dimensions and EQ VAS scores at baseline and at 1-year follow-up among different patient groups and specific diagnoses in 11 National Quality Registers (NQRs) and to compare these with the general population.

Design: Longitudinal, descriptive study

Participants: 261,241 patients from 11 NQRs and 49,169 participants from the general population were included in the study.

Primary and secondary outcome measures: Proportions of problems reported on the five EQ-5D-3L dimensions, EQ VAS scores of participants' own health and proportions of participants and mean/median EQ VAS score in the Paretian Classification of Health Change (PCHC) categories.

Results: In most of the included registers, and the general population, problems with pain/discomfort were the most frequently reported dimension at baseline and at 1-year follow-up. Mean EQ VAS score (SD) ranged from 45.2 (22.4) among disc hernia patients to 88.1 (15.3) in wrist and hand fracture patients at baseline. They ranged from 48.9 (20.9) in pulmonary fibrosis patients to 83.3 (17.4) in wrist and hand fracture patients at follow-up. The study demonstrated the consistency between problems reported on the EQ-5D-3L dimensions and the EQ VAS scores. The *improved* category of PCHC, improvement in at least one dimension without deterioration in any other, accounted for the highest proportion of observations in several diagnoses, corresponding with highest improvement in mean EQ VAS score. The mean EQ VAS score of the selected health states corresponded with their severity, but the score for the same health state differed across diagnoses.

Conclusions: The EQ-5D-3L descriptive system and EQ VAS have an important role in providing a 'common denominator', allowing comparisons across NQRs and specific diagnoses.

Trial registration number: ClinicalTrials.gov (NCT04359628)

For peer review only - http://bmjopen.bmj.com/site/about/guidelines.xhtml

Strength and limitations

- The study presents findings including a large number of diagnoses across many patient groups.
- Data on health-related quality of life from large National Quality Registers and from the general population were included.
- Change in health-related quality of life from baseline to 1-year follow-up was reported using a longitudinal design.
- Differences in health-related quality of life due to different modes of data collection is possible.
- The analysis focused on patients with data both at baseline and 1-year follow-up.

For peer review only - http://bmjopen.bmj.com/site/about/guidelines.xhtml

Introduction

Patient-reported outcomes (PROs) are increasingly used in health care [1]. The health-related quality of life (HRQoL) instrument EQ-5D [2] is a generic (not disease-specific) PRO measure used worldwide for a wide range of conditions and treatments. The EQ-5D descriptive system consists of five dimensions of health: mobility, self-care, usual activities, pain/discomfort, and anxiety/depression [3]. The EQ VAS, a vertical line scaled from 0 ('worst imaginable health') to 100 ('best imaginable health') is a component of the EQ-5D. Although EQ VAS plays a distinctive role in capturing patients' overall assessment of their own health it is seldom the focus of analyses. It can also provide insights into whether the problems patients experience are (or are not) captured by the five dimensions in the EQ-5D descriptive system. More knowledge is needed on what lies behind patients' reporting of HRQoL and to what extent the EQ VAS, can be applied for clinical decision making using summarized data [3]. A study in the UK that assessed routine collection of EQ VAS found a consistent relationship between problems reported on the EQ-5D dimensions and the EQ VAS score [4]. Another study found it feasible to use the EQ VAS in clinical routine in patient diaries [5]. EQ VAS has also been employed in studies as part of the EQ-5D instrument in the assessment of HRQoL among patients with different diagnoses and in different settings [6–12]. The correspondence between EQ-5D dimensions and the EQ VAS components and its variation across four medical conditions has also been reported [13,14]. Studies are needed on how the EQ-5D descriptive system and EQ VAS perform in capturing the level and variation in HRQoL across a wider range of diagnoses, and on health care intervention responsiveness on HRQoL among patients with different diagnoses. The perception of problems experienced in the different dimensions and their responsiveness to health care intervention may differ depending on the nature and burden of the condition. Such studies could provide useful information to clinicians as well as policy makers in guiding the use of the EQ-5D descriptive system including the EQ VAS component in patient care which in some circumstances has led to improved care and patient outcome [15]. Improved care could be achieved through increased understanding of patients' conditions and monitoring improvements after treatment, with due consideration for PROs.

In Sweden, National Quality Registers (NQRs) hold data on individual patients' diseases treatment/intervention and outcomes [16]. There are over 100 NQRs across a wide range of clinical areas, dating as far back as 1975, when the Swedish Knee Arthroplasty Register was established. Most of the NQRs have been initiated by health care professionals [17]. A

For peer review only - http://bmjopen.bmj.com/site/about/guidelines.xhtml

number of clinical areas are covered by the NQRs, with the aim of helping provide patients with the best possible care through, for example, monitoring of health care performance and quality, ongoing learning, and research [14]. The use of a personal identification number (PIN) in Sweden provides an advantage when linking data on patients from the NQRs with those collected within other government administered registers, such as the National Patient Register (NPR) [16]. There have been a number of efforts to use the various NQRs for quality improvement [18]. Most of the NQRs collect PRO data. The most commonly used generic HRQoL instrument is the EQ-5D, which is included in 40 registers [17]. Generic measures provide a means of comparing health problems among patients in different NQRs, to understand the overall severity of problems experienced by patients with different diseases and treatment pathways. Many studies have used EQ VAS as part of the assessment of HRQoL among patients in NQRs. For example, the influence of different surgical approaches to the hip on HRQoL [19], comparison after out-of-hospital and in-hospital cardiopulmonary resuscitation [20], as well as after hip replacement among patients born in Sweden and abroad [21], and self-reported health in patients with congenital heart diseases [22].

Despite studies on HRQoL in different patient groups using EQ-5D and EQ VAS, few studies assessed EQ VAS in comparing HRQoL across a number of diagnoses [9,23,24]. The present descriptive study provides comprehensive information on HRQoL, using the three-level EQ-5D-3L (no, some/moderate, confined to bed/unable to/extreme problems) resulting in 243 health profiles or health states, and the EQ VAS, of several patient groups with various diagnoses, a subject that has not been extensively examined in previous research. The overall aim of this study was to compare problems reported in the five EQ-5D-3L dimensions and EQ VAS scores at baseline and 1-year follow-up among different patient groups and specific diagnoses in 11 NQRs and to compare these with the general population. The objectives were:

- To document the prevalence of problems reported according to level of severity within the EQ-5D-3L dimensions and EQ VAS scores among different patient groups and diagnoses
- To document changes in the prevalence of problems reported according to level of severity within the EQ-5D-3L dimensions, and the change in EQ VAS scores among different patient groups and diagnoses
- To relate the prevalence of problems and mean EQ VAS scores to the characteristics of patients, diagnoses and interventions, at baseline, at 1-year follow-up, and changes over the follow-up and to make comparisons with the general population

For peer review only - http://bmjopen.bmj.com/site/about/guidelines.xhtml

- To assess the variation of mean EQ VAS scores for selected health states across diagnoses and in the general population
- To calculate time-trade-off (TTO) and VAS indices for the diagnoses, and to establish EQ-5D-3L reference data for patients with the different diagnoses

For peer review only

For peer review only - http://bmjopen.bmj.com/site/about/guidelines.xhtml

Methods

Study Design

This is a descriptive study of longitudinal cohorts from 11 Swedish NQRs and comparison with data from the general population. The study is reported in line with the RECORD checklist [25].

Swedish National Quality Registers included in the study

The NQRs included in this study comprise a selection of contrasting disease areas where EQ-5D-3L data are collected. In addition to musculoskeletal conditions, where EQ-5D-3L is most used, our sample also represents patients with heart failure, respiratory failure and psoriasis. The Better Management of Patients with Osteoarthritis (BOA) register is referred to as BOA throughout the manuscript (Supplementary Table S1).

Data from the registers on PROs, Body Mass Index (BMI) and demographic characteristics, at baseline, and at 1-year follow-up were included. A 1-year follow-up was chosen as it is the most common evaluation time in the NQRs which facilitated comparison between them. In registers with no such information, we used the date of data collection to determine the time points. EQ-5D-3L data collected within 90 days of one calendar year after baseline were categorized as 1-year follow-up data, following the approach employed in the Swedish Hip Arthroplasty Register. In the case of patients from the fracture register, baseline data on EQ-5D-3L were collected within four weeks from the fracture date, by recall of the week before the fracture occurred.

Sampling and data

Baseline/first visit and 1-year follow-up data from 2001 to 2019 were retrieved from the registers. Patient records including EQ-5D-3L questionnaire data were selected. Records with complete data on main diagnosis/diagnosis groups and interventions were selected among these. Prior to pooling data from the registers, patients with records in more than one register were randomly de-duplicated. After the exclusion of duplicate records, a total of 266,241 patient records, with complete data on age and sex at baseline, at time of diagnosis and intervention, and on EQ-5D-3L dimensions were included in the analysis of EQ-5D-3L dimensions. In the analysis of EQ VAS data, 209,247 records with complete data were included (Supplementary Table S1). The patient data were compared with the general population, using data on 49,169 individuals with complete data on EQ-5D-3L dimensions

For peer review only - http://bmjopen.bmj.com/site/about/guidelines.xhtml

and 41,761 with complete EQ VAS data. The general population data were available from another study; more details can be found elsewhere [26].

Age was categorized into <30, 30–39, 40–49, 50–59, 60–69, 70–79 and ≥80 years. BMI was categorized as underweight (<18.5), normal (18.5–24.9), overweight (25.0–29.9) and obesity class I (30–34.9), II (35.0–39.9) or III (40+) [27].

Data analysis

Descriptive analysis was performed on age, sex, BMI categories and data on EQ-5D-3L dimensions. The prevalence of problems reported by patients were presented by register (patient group) and by diagnosis, at baseline and 1-year follow-up, and for data from the general population.

Descriptive statistics (mean (SD) and median (IQR)) were also used to present EQ VAS score at baseline, at 1-year follow-up, as well as for the change in score, by register, diagnosis, intervention and by age group, sex, and BMI category in each register. An analysis of covariance (ANCOVA) was performed to make comparisons of the estimated mean EQ VAS scores by sex, adjusted for age in each register. Descriptive analysis of EQ VAS scores was also performed for nine health states (out of the 243), including those occurring most frequently across registers.

For the data at baseline and at 1-year follow-up, a two-level random intercept model (as there could be variations based on the register a diagnosis is grouped in) was conducted to assess how each diagnosis in the different registers influences mean EQ VAS score with the general population data as reference group. The estimates were adjusted for age group and sex. In the statistical analyses, a 95% confidence interval (CI) was employed.

The methods described above for analyses of the NQR data were applied on the general population data.

Paretian Classification of Health Change (PCHC), which categorizes changes in health states over time into *no problem*, *no change*, *improved*, *worsened* and *mixed* was performed using baseline and 1-year follow-up data on health states [28]. Individuals who reported the same health state at both time points (including, but not limited to, health state 11111, i.e. no problems in any of the dimensions) were included in the *no change* category. Improvement in any of the EQ-5D-3L dimensions with no deterioration in the remaining ones leads to categorization as *improved.* On the contrary, a worsening in any of the EQ-5D-3L dimensions with no improvement in the remaining dimensions was categorized as *worsened.* Health states

For peer review only - http://bmjopen.bmj.com/site/about/guidelines.xhtml

characterized by mixed types of changes (both improved and worsened) were grouped in the *mixed* category [28]. By using this categorisation, the proportion of improvements and worsening, as well as the other categories, were calculated for each diagnosis. Similarly, the mean self-assessed EQ VAS scores in these PCHC groups at baseline and 1-year follow-up were calculated according to diagnosis.

As a sensitivity analysis, the demographic characteristics of patients with complete baseline and 1-year follow up data included in the analysis, and those with missing records at 1-year follow-up were compared for each register.

In order to assess the HRQoL profile of patients with complete data at baseline (regardless of follow-up status) and also patients with complete data at 1-year follow-up (regardless of their baseline status), prevalence of problems reported on the EQ-5D-3L dimensions and mean EQ VAS score were calculated. In addition, the EQ-5D-3L data were summarized as EQ-5D-3L indices using the Swedish experience-based EQ-5D-3L TTO and VAS value sets [26].

Patient and public involvement

Neither patients nor the general public were involved in the design, conduct, reporting, or dissemination plans of our research.

For peer review only

Results

Demographic characteristics

A total of 266,241 patient records and 49,169 records from general population were included in the study (Supplementary Table S1). In total, 224 health states were reported both at baseline and at 1-year follow-up in the NQR data, and 156 health states in the general population data. The mean age of included participants ranged from 29.8 years in patients with cruciate ligament injury, to 73.7 years for patients with respiratory failure and 72.8 years in heart failure patients (Table 1). Most of the patients were in their 50s to 70s. However, among heart failure patients nearly one-third were 80 years or older and among the cruciate ligament injury patients the majority were below 30 years of age. The mean age of the general population was 46.2 years (Table 1). A majority of the participants were women, with the exception of patients with psoriasis, heart failure, as well as ankle and cruciate ligament injury. In almost all of the NQRs, a majority of patients had a BMI in the normal and overweight categories (Table 1; Supplementary Table S2 and Supplementary Table S3).

Problems reported on the EQ-5D-3L dimensions

Table 2 presents the prevalence of problems reported on the EQ-5D-3L dimensions across NQRs, at baseline and 1-year follow-up. At both time points, similar to the general population, most problems reported were in the pain/discomfort dimension, with the exception of the respiratory failure patients, where most problems reported concerned mobility. Patients in BOA and those with spine, ankle, knee and hip had the highest prevalence of pain/discomfort of the NQRs included. At the 1-year follow-up, the highest proportion of problems reported were among BOA, rheumatology and spine patients. In most registers, at both baseline and 1-year follow-up, problems with mobility were the second most prevalent.

At both time points, the highest proportion of severe problems reported regards pain/discomfort. This was the case for all registers as well as in the general population, with the exception of patients with fracture and respiratory failure, for which the highest proportion of severe problems concerned the usual activities dimension. In addition, patients with cruciate ligament injury at 1-year follow-up reported the highest proportion of severe problems in the anxiety/depression dimension (Table 2).

The prevalence of problems reported in the EQ-5D-3L dimensions by patients with different diagnoses is presented at baseline and at 1-year follow-up (Supplementary Table S4). For all diagnoses, with exception of COPD and pulmonary fibrosis, most problems were reported

For peer review only - http://bmjopen.bmj.com/site/about/guidelines.xhtml

with pain/discomfort. This was most commonly seen at baseline in the intervention-based registers covering musculoskeletal diseases, with the highest proportion among patients concerned having a diagnosis of rheumatoid arthritis in the knee (100%). Patients with rheumatoid arthritis of the ankle had the highest prevalence of severe problems in the pain/discomfort (69%) dimension. Among patients with COPD and pulmonary fibrosis, the highest prevalence of problems was in the mobility dimension. At 1-year follow-up, the highest proportion of severe problems were reported in the pain/discomfort dimension for most diagnoses, except for patients with lower extremity and axial fractures and for patients with COPD and pulmonary fibrosis.

Severe problems in the anxiety/depression dimension were most prevalent among patients with COPD, both at baseline (9%) and 1-year follow-up (10%). The response, "confined to bed", within the mobility dimension was very uncommon among all diagnoses, with the largest proportion occurring in patients with disc hernia (5%) at baseline and those with fracture on hip and thigh (5%) as well as COPD and pulmonary fibrosis (5%) at 1-year follow-up. The prevalence of severe problems with self-care was highest at 1-year follow-up in patients with a hip or femoral fracture (10%) (Supplementary Table S4).

Paretian Classification of Health Change (PCHC)

Both at baseline and 1-year follow-up, a total of 224 (of 243 possible) health states were reported. Figure 1 presents the proportions of patients by PCHC group for the different diagnoses. The highest proportions of improvements were shown for patients in the intervention-based ankle, hip, knee and spine registers. For most of the other diagnoses, the highest proportions of patients were classified as *improved*. The respiratory failure patient group was an exception, of whom most were classified as *worsened*. Another exception was the fracture patient group where the highest proportion of patients were classified as *worsened*, as their baseline data were collected by recall of the week before the fracture occurred. Most fracture patients had a better health state at baseline than at 1-year follow-up. The heart failure patients had similar proportions of patients classified as *improved* and *worsened*.

Mean self-assessed EQ VAS score

Mean (Table 3) and median (Supplementary Table S5) self-assessed EQ VAS scores by sex, age groups, and BMI categories are described for each register at baseline, 1-year follow-up, the change over the period, and for the general population. The highest mean score at both time points was found among fracture patients (84.7 (baseline) and 77.7 (1 year)

For peer review only - http://bmjopen.bmj.com/site/about/guidelines.xhtml

respectively). The lowest scores were found among spine patients (47.8) at baseline, and respiratory failure patients (49.1) at 1-year follow-up. The mean EQ VAS score in the general population was 79.5. The scores were found to have increased in all patients at the 1-year follow-up, with the exception of respiratory failure patients and fracture patients. The highest increases were recorded among hip (20.5) and spine surgery (20.1) patients.

Similar to the general population, in most registers, women had statistically significantly lower mean EQ VAS scores after adjustment for age in the ANCOVA. Exceptions were seen among fracture and respiratory failure patients at baseline and BOA, fracture, heart failure and respiratory failure patients at 1-year follow-up (Supplementary Table S6). The mean EQ VAS score showed greater improvements among women in most of the registers, with the exception of ankle, cruciate ligament injury, heart and respiratory failure patients.

In most registers with BMI data, the highest mean EQ VAS scores were found in the normal and overweight categories, at both time points. However, no clear pattern was shown in terms of the change in mean EQ VAS score (Table 3).

Table 4 describes mean EQ VAS score by diagnosis at baseline, 1-year follow-up, and the change over the period. Mean EQ VAS scores had more similarity among diagnoses within the same patient group (register) than with diagnoses in other patient groups. Some differences were shown by diagnosis in some patient groups such as fracture (e.g. hip and thigh) and hip (e.g. inflammatory joint disorder) which were illustrated to be statistically significant in the results of the two-level random intercept model in Figure 2. For most of the diagnoses, EQ VAS scores increased over the follow-up period; however, they decreased among patients with respiratory failure and fracture. Mean EQ VAS scores by interventions, across all NQRs, are presented in Supplementary Table S7.

Figure 2 illustrates the two-level random intercept model estimates of mean EQ VAS scores for the different diagnoses, adjusted for age and sex, with the general population as a reference group. At baseline, patients with diagnoses other than fractures showed statistically significantly lower mean EQ VAS scores as compared with the general population. At 1-year follow-up, all patients, except those with BOA hip, fracture, and respiratory failure diagnosis showed improvement in mean EQ VAS, while remaining lower than in the general population with the exception of fracture patients.

Table 5 presents mean EQ VAS scores, at baseline and 1-year follow-up, in the various PCHC categories within the different diagnoses. Mean EQ VAS scores showed corresponding

For peer review only - http://bmjopen.bmj.com/site/about/guidelines.xhtml

increases and decreases in the categories, *improved* and *worsened*. The largest increases in the *improved* category were found among diagnoses in the hip and spine patient groups. In the *worsened* category, diagnoses in the fracture patient groups showed the largest decrements, based on their recall of their health status before the fracture occurred. In the categories, *no change* and *mixed*, increase in mean EQ VAS score was found in all diagnoses, with the exception of the fracture and respiratory failure patient groups, which instead showed decrements.

The mean (Table 6 for baseline, Table 7 for 1-year follow-up) and median (Supplementary Table S8 for baseline, Supplementary Table S9 for 1-year follow-up) self-assessed EQ VAS scores are presented for nine selected health states across diagnoses, and in the general population. The mean scores were generally consistent with the level of severity of the health states at baseline and at 1-year follow-up. The mean EQ VAS scores for health state 11111 (no problem any dimension) showed more similarity across most of the diagnoses at 1-year follow-up than at baseline. At baseline, the mean EQ VAS scores for health state 22222 (some problems in all dimensions) ranged from 38.2 for patients with other spine conditions, to 62.9 for patients with fractures of the knee and the lower leg. At 1-year follow-up, the range was from 41.7 for patients with cruciate ligament injury to 57.9 for patients with rheumatoid arthritis of the knee. In the general population, the mean EQ VAS score for health state 22222 was 44.4.

A comparison of patient records with complete data at baseline and at 1-year follow-up with data from patients with only baseline data is presented (Supplementary Table S10 and Supplementary Table S11). The results showed that higher proportions of women and older patients had complete baseline and 1-year follow-up data in many of the registers.

Problems reported on EQ-5D-3L dimensions and mean self-assessed EQ VAS score among patients at baseline (regardless of follow up status), among those with 1-year follow-up data (regardless of baseline status), and for the general population were calculated (Supplementary Table S12 and Supplementary Table S13). The tables also present EQ-5D-3L indices based on the Swedish experience-based TTO and VAS value sets [26]. The results presented in Table S11 can serve as EQ-5D-3L reference data for patients with these diagnoses, with the exception of the fracture patients as their baseline data comes from before the fracture date. The pattern of problems reported, and mean EQ VAS scores, were comparable to those reported at the corresponding time point among patients with data both at baseline and at 1-year follow-up.

For peer review only - http://bmjopen.bmj.com/site/about/guidelines.xhtml

Discussion

Main findings

This study documented self-reported health status in different patient groups, and for specific diagnoses in the NQRs, as well as in the general population. Higher proportions of problems and lower self-assessed EQ VAS scores (worse overall HRQoL) were reported among patients with diagnoses with more symptoms experienced captured by the EQ-5D-3L dimensions. In general, most problems were reported in the pain/discomfort dimension. Findings on the EQ VAS scores showed correspondence with problems reported using the EQ-5D-3L descriptive system, with lower mean EQ VAS scores corresponding with a greater prevalence of reported problems.

Comparisons with other studies

The proportions of any problems reported on the EQ-5D-3L dimensions, and distribution between *some* and *severe* levels of problem, seem to vary depending on the differences in the symptoms of the diseases as well as the way in which they are experienced by the patients [29–33]. Specifically, patients in the intervention-based registers, such as spine, hip, and knee, showed the highest proportions of problems in the EQ-5D-3L dimensions. This was particularly the case regarding the pain/discomfort dimension, which reflects the pain symptoms associated with the diseases [30–33]. Pain/discomfort was similarly the dimension with the highest proportion of problems reported, also in the general population. Substantially less reported problems were noted after surgical interventions in patients from intervention-based registers. The high proportions of problems within the mobility and usual activities dimensions in COPD and pulmonary fibrosis patients might be due to limitations in mobility and other functions and, in some cases, the need to use breathing equipment. Other studies of patients with respiratory diseases also reported comparably high proportions of problems within the mobility, usual activities and pain/discomfort dimensions [29,34].

The EQ-5D-3L dimensions for which higher proportions of problems were reported seem to be partly determined by the type of symptoms/manifestations of the disease in patients, as indicated above. For instance, when comparing spine and psoriasis patients, the proportion and severity of problems in both the pain/discomfort and mobility dimensions were much lower for the psoriasis patients. This provided evidence for the ability of the EQ-5D-3L to discriminate among conditions that affect specific aspects of health differently, although some dimensions more relevant to some diseases may not be covered. Even in comparison to heart failure patients, who were on average much older than spine patients, higher proportions of

For peer review only - http://bmjopen.bmj.com/site/about/guidelines.xhtml

problems with pain/discomfort and mobility were reported in spine patients. The low prevalence of severe problems ("confined to bed") with mobility might indicate the importance of applying the five-level EQ-5D-5L among these patient groups [35].

Regarding the anxiety/depression dimension, in most of the registers fewer than half of the patients reported problems, with the exception of spine, cruciate ligament injury, respiratory failure and psoriasis patients at baseline, and respiratory failure patients at 1-year follow-up. Compared to the general population, the proportion of problems with anxiety/depression was higher than in most of the registers at baseline. NQRs in which patients reported higher proportions of problems mainly on the pain/discomfort and mobility dimensions also had higher proportions of problems with anxiety/depression. This may be related to the pain, reduced walking ability and impaired ability to perform everyday activities they reported, which might affect the mood dimension. The interrelated nature of the EQ-5D dimensions has been reported for the EQ-5D-5L assessing causal and effect relationships, where empirical evidence showed mobility to be a causal indicator [36]. The greatest problems in the mood dimension were reported among respiratory and spine patients at baseline and 1-year follow-up, and in rheumatology patients at 1-year follow-up. This could be related to the very restrictive nature of the diseases [30,34,37]. Other studies have also shown that more than half of patients with severe COPD, as well as those undergoing spinal surgery, report problems on the anxiety/depression dimension [38,39].

The longitudinal change in reported problems in the dimensions showed that, while improvement was observed in most of the NQRs, larger improvements were reported in intervention-based registers. These patients also reported the highest proportions of problems at baseline and were subsequently provided with surgical interventions. Among these were hip, ankle, knee and spine patients. This pattern was also observed in the specific diagnoses within the various patient groups and PCHC categories. PCHC has been employed in other studies to document change over time in various diseases, such as heart failure, prostate cancer, diabetes mellitus and overactive bladder [40–44]. The change for the fracture patients cannot be compared directly to changes for other patient groups due to the recall technique used for health state before injury.

The mean self-assessed EQ VAS scores showed variation across NQRs and diagnoses, with the lowest scores reported among patient groups with symptoms captured by the EQ-5D-3L dimensions. In almost all registers, the EQ VAS scores were lower, both at baseline and at 1-year follow-up, compared to findings in the general population data, for which the mean score

For peer review only - http://bmjopen.bmj.com/site/about/guidelines.xhtml

was 79.5 [26]. The EQ VAS scores from the registers demonstrated consistency with problems reported in the EQ-5D-3L dimensions. For example, spine and respiratory failure patients, who reported the highest proportions of problems at baseline, also had the lowest mean EQ VAS scores. In a study that assessed the performance of EQ-5D among patients with irritable bowel syndrome, EQ VAS score also was found to differ by level of severity [45]. The change in mean EQ VAS score also corresponded with changes in the problems reported in the descriptive system. This was shown by the largest increases in EQ VAS score among spine and hip patients. As the baseline level determines the room for improvement, the low baseline scores among these patient groups may have partly contributed to the large increments shown. The findings were in line with previous studies demonstrating the consistency of the EQ-5D descriptive system with the mean EQ VAS score [4,13].

The consistent pattern of changes in the prevalence of problems reported in the EQ-5D-3L and the corresponding mean EQ VAS scores was also shown in the PCHC categories. This was particularly evident in an increase in the proportion of patients that were classified as *improved*, and a decrease in the proportion classified as *worsened*, in terms of EQ VAS scores. However, as also discussed in previous studies [4,46], a pattern which might partly be attributed to the broader construct in EQ VAS than the EQ-5D-3L descriptive system, was shown in the *no problem* category. In this category, mean EQ VAS scores increased over the 1-year follow-up, though they differed by diagnosis. The PCHC is potentially useful as a simple means of summarising changes using profile data without need for a value set. But, if there is a high proportion of patients classified as *mixed*, the method is less useful. PCHC does not inform on the magnitude of improvement or worsening in HRQoL.

The lower mean EQ VAS scores, consistently lower among women in most of the NQRs, were similar to findings reported in the literature among patients with different diseases, as well as in the general population [47–49]. However, comparable findings on EQ VAS by sex were also reported [50]. Greater improvement was nevertheless found among women in many of the patient groups over the period up to the 1-year follow-up.

The variation in EQ VAS scores may be due to differences in the symptoms experienced in the different patient groups, as indicated by age- and sex-adjusted two-level random intercept model estimates. In most diagnoses, lower EQ VAS score than in the general population were shown at baseline, and these improved at 1-year follow-up. The mean EQ VAS score across diagnoses also showed a general pattern of more similar scores among diagnoses within the

For peer review only - http://bmjopen.bmj.com/site/about/guidelines.xhtml

same NQR (patient group) than those across the different NQRs. One possible reason for this could be attributed to the similarity of diagnoses and symptoms in the same register.

The relationship between EQ-5D-3L and EQ VAS scores discussed above was observed in the mean EQ VAS scores of the nine selected health states across specific diagnoses. Mean EQ VAS scores were consistent with severity of health states, with lower scores for the more severe health states going from 11111 to 33333. In a study of EQ VAS scores of selected same health states, a similar pattern of lower scores among more severe health states was observed [51]. Mean EQ VAS scores for the same health states across different diagnoses and the general population showed variations, with the lowest scores observed among diagnoses such as COPD. For example, for health state 11111, the general population had a mean score of 88.8 while hip patients with avascular necrosis 60.3 and patients with hip inflammatory joint disorder had 84.4 at baseline. This provides further evidence for the discussion above, which EQ VAS has a broader nature than the EQ-5D-3L dimensions. Further study is warranted to explore the reasons for this. Mean EQ VAS scores for the same health states also showed an increase in most of the diagnoses over the 1-year follow-up period.

Strengths and limitations of the study

One of the strengths of the present study is the coverage of several diagnoses across different patient groups in the NQRs. The use of large data sets, providing information on the HRQoL of patients, together with data from the general population, made it possible to conduct relevant comparisons. Furthermore, the study assessed longitudinal change in HRQoL of patients from baseline to 1-year follow-up, making comparisons across the different NQRs. In addition, the study adds new data on patients with diagnoses that have previously been less studied. One limitation of the present study may be related to possible differences in the reported HRQoL arising due to different modes of data collection employed across NQRs. Another limitation relates to the inclusion of patients having both data at baseline and at 1-year follow-up, while excluding non-respondents, those with only baseline or 1-year follow-up data, and patients with incomplete entries. This may introduce bias. However, separate analyses of baseline data (regardless of 1-year follow-up) and 1-year follow-up data (regardless of baseline status) showed findings consistent with those included in the main analysis. Furthermore, in a study among patients who underwent THR, 1-year and 6-years follow-up data showed generally comparable prevalence of problems in EQ-5D-3L dimensions and similar mean EQ VAS scores [52]. Analyses of non-responders at the 1-year

For peer review only - http://bmjopen.bmj.com/site/about/guidelines.xhtml

follow-up showed statistically significant but small differences in sex and age, as compared to respondents with follow-up data in many registers.

Implications of the findings

The present study contributes by documenting HRQoL among several patient groups, as compared to the general population. It adds to the literature in the area where studies covering various diagnoses have not been widely conducted. By using routinely collected comprehensive data from a number of NQRs in Sweden, the study also adds to previous findings on the consistency between problems reported on the EQ-5D-3L dimensions and the EQ VAS. This is a useful addition to the literature, showing the importance of using the EQ VAS to describe the overall health from the patient perspective, using relatively concise data. This can be valuable in monitoring the health status and progress of different subgroups (age, sex, clinical stages) of patients. The present study demonstrated an important feature of the EQ-5D-3L dimensions and the EQ VAS, showing differences across diagnoses. Inconsistencies in EQ-5D profiles and EQ VAS scores between diagnosis groups indicate that the quality of symptoms described by the EQ-5D dimensions may affect the influence on patients' overall assessment of their own health as measured with the EQ VAS, which warrants further investigations. Nevertheless, this strengthens the role of the EQ VAS as an important PRO measure in various patient groups.

For peer review only

For peer review only - http://bmjopen.bmj.com/site/about/guidelines.xhtml

Conclusions

The present study contributes to the literature by documenting self-reported health status for several different patient groups and diagnoses using data collected through the EQ-5D-3L questionnaire, and comparing these with data on the general population. The consistency between problems reported on the EQ-5D-3L dimension and the EQ VAS score demonstrated component corresponding patterns at baseline and at 1-year follow-up.

The purported broader construct, the EQ VAS component, was compared to the EQ-5D-3L descriptive system. The same health states were found to exhibit variations in self-assessed EQ VAS scores across diagnoses, as well as over the follow-up period. This needs to be investigated further, in order to deepen the understanding of patients' condition and monitor improvements after treatments, with due consideration for the patients' overall assessment of their own health status as rated on the EQ VAS.

This study shows the important role of PROs in routine clinical care, to assess and follow-up health status and progress within different groups of patients. Together with clinical data, this could provide crucial information for understanding and improving the patients' health status.

Contributors: KB and OR, conceived the study. FST, OR, KB, ND, DP, EN and members of the SWEQR Study Group (AA, ME, MF, PF, ÅJ, ML, MM, MR, BR, MSE, JV, AWD) designed the study. FST performed the data analysis and preliminary interpretation of the data. KB, OR, ND, DP and EN supervised data analysis and interpretation of findings. FST drafted the manuscript. KB, OR, ND, DP, EN and members of the SWEQR Study Group (AA, ME, MF, PF, ÅJ, ML, MM, MR, BR, MSE, JV, AWD) revised the draft manuscript for important intellectual content. The corresponding author attests that all the authors in the list fulfil the authorship criteria and no other who fulfil the criteria were left out. All authors approved the submission of the manuscript. All the authors agree to be responsible for all aspects of the manuscript.

Funding: This research project is supported by a grant from The EuroQol Research Foundation (EQ Project # 2016480) and Stockholm County Council (# 4-3464/2018), Stockholm, Sweden. The funding bodies did not influence the design, conduct and reporting of the study.

Competing interests: KB, ND and DP are members of the EuroQol Group. KB reports grants from EuroQol Research Foundation, grants from Stockholm County Council, during the conduct of the study; personal fees from Region Stockholm, outside the submitted work. ML

For peer review only - http://bmjopen.bmj.com/site/about/guidelines.xhtml

reports personal fees from Lundbeck pharmaceuticals, outside the submitted work. OR reports institutional compensation for educational consultancy from Link Sweden; institutional compensation for research consultancy from Pfizer, outside the submitted work. AA, ME, MF, PF, ÅJ, MM, EN, MR, BR, MSE, FST, AWD and JV declare no competing interests.

Ethical approval: Ethical approval (# 1185-18/2019-00812 and #2020-04369) was obtained from the Regional Ethics Review Board in Gothenburg, Sweden, for the data from the National Quality Registers and from the Swedish Ethical Review Authority (# 2020-03090) for the general population data.

Data availability statement: Data sharing is not possible according to Swedish law.

Author License: I, the Submitting Author has the right to grant and does grant on behalf of all authors of the Work (as defined in the author licence), an exclusive licence and/or a non-exclusive licence for contributions from authors who are: i) UK Crown employees; ii) where BMJ has agreed a CC-BY licence shall apply, and/or iii) in accordance with the terms applicable for US Federal Government officers or employees acting as part of their official duties; on a worldwide, perpetual, irrevocable, royalty-free basis to BMJ Publishing Group Ltd ("BMJ") its licensees.

For peer review only

References

1 Appleby J, Devlin N, Parkin D. *Using Patient Reported Outcomes to Improve Health Care*. John Wiley & Sons 2015.

2 Brooks R, Boye KS, Slaap B. EQ-5D: a plea for accurate nomenclature. *Journal of Patient-Reported Outcomes* 2020;**4**:52. doi:10.1186/s41687-020-00222-9

3 Rabin R, de Charro F. EQ-5D: a measure of health status from the EuroQol Group. *Ann Med* 2001;**33**:337–43. doi:10.3109/07853890109002087

4 Feng Y, Parkin D, Devlin NJ. Assessing the performance of the EQ-VAS in the NHS PROMs programme. *Qual Life Res* 2014;**23**:977–89.

5 Parkin D, Rice N, Jacoby A, *et al.* Use of a visual analogue scale in a daily patient diary: modelling cross-sectional time-series data on health-related quality of life. *Soc Sci Med* 2004;**59**:351–60. doi:10.1016/j.socscimed.2003.10.015

6 Sari S, Bilberg R, Søgaard Nielsen A, *et al.* The effect of exercise as adjunctive treatment on quality of life for individuals with alcohol use disorders: a randomized controlled trial. *BMC Public Health* 2019;**19**:727. doi:10.1186/s12889-019-7083-8

7 Boczor S, Daubmann A, Eisele M, *et al.* Quality of life assessment in patients with heart failure: validity of the German version of the generic EQ-5D-5L™. *BMC Public Health* 2019;**19**:1464. doi:10.1186/s12889-019-7623-2

8 Roman A, Barbera JA, Castillo MJ, *et al.* Health-Related Quality of Life in a National Cohort of Patients With Pulmonary Arterial Hypertension or Chronic Thromboembolic Pulmonary Hypertension. *Arch Bronconeumol* 2013;**49**:181–8. doi:10.1016/j.arbr.2012.12.002

9 Tan Z, Liang Y, Liu S, *et al.* Health-related quality of life as measured with EQ-5D among populations with and without specific chronic conditions: a population-based survey in Shaanxi Province, China. *PLoS ONE* 2013;**8**:e65958. doi:10.1371/journal.pone.0065958

10 Stark RG, Reitmeir P, Leidl R, *et al.* Validity, reliability, and responsiveness of the EQ-5D in inflammatory bowel disease in Germany. *Inflamm Bowel Dis* 2010;**16**:42–51. doi:10.1002/ibd.20989

11 Szentes BL, Schwarzkopf L, Kirsch F, *et al.* Measuring quality of life in COPD patients: comparing disease-specific supplements to the EQ-5D-5L. *Expert Rev Pharmacoecon Outcomes Res* 2019;:1–7. doi:10.1080/14737167.2019.1662302

12 von Vogelsang A-C, Burström K, Wengström Y, *et al.* Health-Related Quality of Life 10 Years After Intracranial Aneurysm Rupture: A Retrospective Cohort Study Using EQ-5D. *Neurosurgery* 2013;**72**:397–406. doi:10.1227/NEU.0b013e3182804686

13 Whynes DK. Correspondence between EQ-5D health state classifications and EQ VAS scores. *Health Qual Life Outcomes* 2008;**6**:94. doi:10.1186/1477-7525-6-94

14 Whynes DK. Does the correspondence between EQ-5D health state description and VAS score vary by medical condition? *Health Qual Life Outcomes* 2013;**11**:155.

15 Guyatt GH, Ferrans CE, Halyard MY, *et al.* Exploration of the value of health-related quality-of-life information from clinical research and into clinical practice. *Mayo Clin Proc* 2007;**82**:1229–39. doi:10.4065/82.10.1229

16 Nationella Kvalitetsregister. About the National Quality Registries. 2019.http://kvalitetsregister.se/englishpages/aboutqualityregistries.2422.html (accessed 22 Jul 2020).

17 Emilsson L, Lindahl B, Köster M, *et al.* Review of 103 Swedish Healthcare Quality Registries. *J Intern Med* 2015;**277**:94–136. doi:10.1111/joim.12303

For peer review only - http://bmjopen.bmj.com/site/about/guidelines.xhtml

18 Fredriksson M, Halford C, Eldh AC, *et al.* Are data from national quality registries used in quality improvement at Swedish hospital clinics? *Int J Qual Health Care* 2017;**29**:909–15. doi:10.1093/intqhc/mzx132

19 Lindgren JV, Wretenberg P, Kärrholm J, *et al.* Patient-reported outcome is influenced by surgical approach in total hip replacement: a study of the Swedish Hip Arthroplasty Register including 42,233 patients. *Bone Joint J* 2014;**96-B**:590–6. doi:10.1302/0301-620X.96B5.32341

20 Djärv T, Bremer A, Herlitz J, *et al.* Health-related quality of life after surviving an out-of-hospital compared to an in-hospital cardiac arrest: A Swedish population-based registry study. *Resuscitation* 2020;**151**:77–84. doi:10.1016/j.resuscitation.2020.04.002

21 Krupic F, Garellick G, Gordon M, *et al.* Different patient-reported outcomes in immigrants and patients born in Sweden. *Acta Orthopaedica* 2014;**85**:221–8. doi:10.3109/17453674.2014.919556

22 Berghammer M, Karlsson J, Ekman I, *et al.* Self-reported health status (EQ-5D) in adults with congenital heart disease. *Int J Cardiol* 2013;**165**:537–43. doi:10.1016/j.ijcard.2011.10.002

23 Cook EL, Harman JS. A Comparison of Health-Related Quality of Life for Individuals with Mental Health Disorders and Common Chronic Medical Conditions. *Public Health Rep* 2008;**123**:45–51.

24 Singh K, Kondal D, Shivashankar R, *et al.* Health-related quality of life variations by sociodemographic factors and chronic conditions in three metropolitan cities of South Asia: the CARRS study. *BMJ Open* 2017;**7**. doi:10.1136/bmjopen-2017-018424

25 Benchimol EI, Smeeth L, Guttmann A, *et al.* The REporting of studies Conducted using Observational Routinely-collected health Data (RECORD) Statement. *PLOS Medicine* 2015;**12**:e1001885. doi:10.1371/journal.pmed.1001885

26 Burström K, Sun S, Gerdtham U-G, *et al.* Swedish experience-based value sets for EQ-5D health states. *Qual Life Res* 2014;**23**:431–42.

27 WHO consultation on obesity. Obesity: Preventing and managing the global epidemic, Report of a WHO Consultation. Geneva, Switzerland: : WHO 2000. http://whqlibdoc.who.int/trs/WHO_TRS_894.pdf. (accessed 22 Jul 2020).

28 Devlin NJ, Parkin D, Browne J. Patient-reported outcome measures in the NHS: new methods for analysing and reporting EQ-5D data. *Health Econ* 2010;**19**:886–905. doi:10.1002/hec.1608

29 Hazell M, Frank T, Frank P. Health related quality of life in individuals with asthma related symptoms. *Respiratory Medicine* 2003;**97**:1211–8. doi:10.1016/S0954-6111(03)00249-X

30 Melancia JL, Francisco AF, Antunes JL. Spinal stenosis. *Handb Clin Neurol* 2014;**119**:541–9. doi:10.1016/B978-0-7020-4086-3.00035-7

31 Humphreys SC, Eck JC. Clinical Evaluation and Treatment Options for Herniated Lumbar Disc. *AFP* 1999;**59**:575.

32 Lespasio MJ, Sultan AA, Piuzzi NS, *et al.* Hip Osteoarthritis: A Primer. *Perm J* 2018;**22**. doi:10.7812/TPP/17-084

33 Lespasio MJ, Piuzzi NS, Husni ME, *et al.* Knee Osteoarthritis: A Primer. *Perm J* 2017;**21**. doi:10.7812/TPP/16-183

34 Kim S-H, Oh YM, Jo M-W. Health-related quality of life in chronic obstructive pulmonary disease patients in Korea. *Health Qual Life Outcomes* 2014;**12**:57. doi:10.1186/1477-7525-12-57

35 Herdman M, Gudex C, Lloyd A, *et al.* Development and preliminary testing of the new five-level version of EQ-5D (EQ-5D-5L). *Qual Life Res* 2011;**20**:1727–36. doi:10.1007/s11136-011-9903-x

36 Gamst-Klaussen T, Gudex C, Olsen JA. Exploring the causal and effect nature of EQ-5D dimensions: an application of confirmatory tetrad analysis and confirmatory factor analysis. *Health Qual Life Outcomes* 2018;**16**:153. doi:10.1186/s12955-018-0975-y

For peer review only - http://bmjopen.bmj.com/site/about/guidelines.xhtml

37 Lee DM, Weinblatt ME. Rheumatoid arthritis. *The Lancet* 2001;**358**:903–11. doi:10.1016/S0140-6736(01)06075-5

38 Chotai S, Khan I, Nian H, *et al.* Utility of Anxiety/Depression Domain of EQ-5D to Define Psychological Distress in Spine Surgery. *World Neurosurg* 2019;**126**:e1075–80. doi:10.1016/j.wneu.2019.02.211

39 Menn P, Weber N, Holle R. Health-related quality of life in patients with severe COPD hospitalized for exacerbations - comparing EQ-5D, SF-12 and SGRQ. *Health Qual Life Outcomes* 2010;**8**:39. doi:10.1186/1477-7525-8-39

40 Jonsson Å, Orwelius L, Dahlstrom U, *et al.* Evaluation of the usefulness of EQ-5D as a patient-reported outcome measure using the Paretian classification of health change among patients with chronic heart failure. *Journal of Patient-Reported Outcomes* 2020;**4**:50. doi:10.1186/s41687-020-00216-7

41 Devlin N, Herdman M, Pavesi M, *et al.* Health-related quality of life effects of enzalutamide in patients with metastatic castration-resistant prostate cancer: an in-depth post hoc analysis of EQ-5D data from the PREVAIL trial. *Health Qual Life Outcomes* 2017;**15**. doi:10.1186/s12955-017-0704-y

42 Pavesi M, Devlin N, Hakimi Z, *et al.* Understanding the effects on HR-QoL of treatment for overactive bladder: a detailed analysis of EQ-5D clinical trial data for mirabegron. *J Med Econ* 2013;**16**:866–76. doi:10.3111/13696998.2013.802240

43 McClure NS, Sayah FA, Ohinmaa A, *et al.* Minimally Important Difference of the EQ-5D-5L Index Score in Adults with Type 2 Diabetes. *Value Health* 2018;**21**:1090–7. doi:10.1016/j.jval.2018.02.007

44 Hakimi Z, Herdman M, Pavesi M, *et al.* Using EQ-5D-3L and OAB-5D to assess changes in the health-related quality of life of men with lower urinary tract symptoms associated with benign prostatic hyperplasia. *Qual Life Res* 2017;**26**:1187–95. doi:10.1007/s11136-016-1460-x

45 Bushnell DM, Martin ML, Ricci J-F, *et al.* Performance of the EQ-5D in patients with irritable bowel syndrome. *Value Health* 2006;**9**:90–7. doi:10.1111/j.1524-4733.2006.00086.x

46 McCaffrey N, Kaambwa B, Currow DC, *et al.* Health-related quality of life measured using the EQ-5D–5L: South Australian population norms. *Health Qual Life Outcomes* 2016;**14**:133.

47 Burström K, Johannesson M, Diderichsen F. Health-related quality of life by disease and socio-economic group in the general population in Sweden. *Health Policy* 2001;**55**:51–69.

48 Faxén UL, Hage C, Donal E, *et al.* Patient reported outcome in HFpEF: Sex-specific differences in quality of life and association with outcome. *International Journal of Cardiology* 2018;**267**:128–32. doi:10.1016/j.ijcard.2018.04.102

49 Janssen MF, Szende A, Cabases J, *et al.* Population norms for the EQ-5D-3L: a cross-country analysis of population surveys for 20 countries. *Eur J Health Econ* 2019;**20**:205–16. doi:10.1007/s10198-018-0955-5

50 Stavem K, Augestad LA, Kristiansen IS, *et al.* General population norms for the EQ-5D-3 L in Norway: comparison of postal and web surveys. *Health Qual Life Outcomes* 2018;**16**. doi:10.1186/s12955-018-1029-1

51 Heijink R, Reitmeir P, Leidl R. International comparison of experience-based health state values at the population level. *Health Qual Life Outcomes* 2017;**15**.

52 Teni FS, Burström K, Berg J, *et al.* Predictive ability of the American Society of Anaesthesiologists physical status classification system on health-related quality of life of patients after total hip replacement: comparisons across eight EQ-5D-3L value sets. *BMC Musculoskeletal Disorders* 2020;**21**:441. doi:10.1186/s12891-020-03399-8

Supplementary material 1: Supplementary tables

For peer review only - http://bmjopen.bmj.com/site/about/guidelines.xhtml

Tables and figures

Table 1: Demographic characteristics and Body Mass Index (BMI) in the 11 National Quality Registers (NQRs) and in the general population

Variable	Intervention-based registers							Diagnosis-based registers				General population
	Spine	Hip	Knee	Ankle	Cruciate ligament	BOA	Fracture	Heart failure	Respiratory failure	Psoriasis	Rheumatology	
	n=48,960	n=90,660	n=16,324	n=700	n=8,430	n=13,647	n=50,892	n=1,436	n=1,050	n=2,680	n=31,462	n=49,169
	%	%	%	%	%	%	%	%	%	%	%	%
**Age in years** [mean (SD)]	58.8 (15.3)	68.5 (10.3)	68.9 (8.7)	63.2 (11.7)	29.8 (10.0)	65.5 (9.1)	62.1 (18.1)	72.8 (11.1)	73.7 (7.7)	50.3 (14.8)	54.3 (15.7)	46.2 (15.3)
**Age group**												
<30	1,674 (3.4)	122 (0.1)	1 (0.0)	10 (1.4)	4,964 (58.9)	6 (0.0)	3,793 (7.5)	2 (0.1)	-	251 (9.4)	2,593 (8.2)	8,160 (16.6)
30-39	4,652 (9.5)	625 (0.7)	10 (0.1)	21 (3.0)	1,797 (21.3)	76 (0.6)	2,870 (5.6)	7 (0.5)	1 (0.1)	422 (15.7)	3,483 (11.1)	9,708 (19.7)
40-49	7,695(15.7)	3,394 (3.7)	278 (1.7)	52 (7.4)	1,299 (15.4)	624 (4.6)	4,651 (9.1)	37 (2.6)	1 (0.1)	581 (21.7)	5,353 (17.0)	9,964 (20.3)
50-59	8,964(18.3)	11,766 (13.0)	2,136 (13.1)	145 (20.7)	347 (4.1)	2,537(18.6)	8,216 (16.1)	137 (9.5)	41 (3.9)	616 (23.0)	6,721 (21.4)	10,491 (21.3)
60-69	11,835(24.2)	30,285 (33.4)	5,814 (35.6)	233 (33.3)	22 (0.3)	5,849 (42.9)	11,918 (23.4)	294 (20.5)	265 (25.2)	568 (21.2)	6,893(25.1)	7,602 (15.5)
70-79	10,999 (22.5)	31,869 (35.2)	6,296 (38.6)	215 (30.7)	1 (0.0)	3,836 (28.1)	10,799 (21.2)	512 (35.7)	494 (47.0)	201 (7.5)	4,448 (14.1)	2,978 (6.1)
80+	3,141 (6.4)	12,599 (13.9)	1,789 (11.0)	24 (3.4)	-	719 (5.3)	8,645 (17.0)	447 (31.1)	248 (23.6)	41 (1.5)	971 (3.1)	266 (0.5)
**Sex**												
Women	25,328 (51.7)	51,263 (56.5)	9,392 (57.5)	311 (44.4)	3,772 (44.7)	9,844 (72.1)	33,781 (66.4)	507(35.3)	623 (59.3)	994 (37.1)	20,668 (65.7)	27,700 (56.3)
**BMI (Kg/m²)***	n=45,343	n=69,206	n=15,937	n=447	n=4,331	n=13,366			n=868	n=2,620		n=22,094
Underweight	264 (0.6)	501 (0.7)	26 (0.2)	3 (0.7)	14 (0.3)	40 (0.3)	-	-	118 (13.6)	36 (1.4)	-	461 (2.1)
Normal	15,371 (33.9)	22,103 (31.9)	3,106 (19.5)	97 (21.7)	2,547 (58.8)	3,728 (27.9)	-	-	361 (41.6)	760 (29.0)	-	12,599 (57.0)
Overweight	19,823 (43.7)	30,164 (43.6)	7,240 (45.4)	200 (44.7)	1,466 (33.8)	5,875 (44.0)	-	-	220 (25.3)	999 (38.1)	-	6,837 (30.9)
Obesity class I	8,089 (17.8)	12,596 (18.2)	4,217 (26.5)	107 (23.9)	252 (5.8)	2,672 (20.0)	-	-	110 (12.7)	534 (20.4)	-	1,683 (7.6)
Obesity class II	1,549 (3.4)	3,200 (4.6)	1,138 (7.1)	32 (7.2)	39 (0.9)	801 (6.0)	-	-	46 (5.3)	202 (7.7)	-	366 (1.7)
Obesity class III	247 (0.5)	642 (0.9)	210 (1.3)	8 (1.8)	13 (0.3)	250 (1.9)	-	-	13 (1.5)	89 (3.4)	-	148 (0.7)
Missing (count)	3,617	21,454	387	253	4,099	281	-	-	182	60	-	27,075

BMI categories: Underweight: <18.5; Normal: 18.5–24.9; Overweight: 25.0–29.9; Obese class I: 30.0–34.9; Obese class II: 35.0–39.9; Obese class III: ≥40

*Percentage based on non-missing values; Proportions rounded off to one decimal using MS Excel

For peer review only

For peer review only - http://bmjopen.bmj.com/site/about/guidelines.xhtml

Table 2: Prevalence of problems reported on EQ-5D-3L, at baseline and 1-year follow-up in the 11 National Quality Registers (NQRs) and in the general population

	Patient group in NQRs/	Severity level	EQ-5D-3L dimension									
			Baseline					1-year follow-up				
			Mobility	Self-care	Usual activities	Pain/ discomfort	Anxiety/ depression	Mobility	Self-care	Usual activities	Pain/ discomfort	Anxiety/ depression
			%	%	%	%	%	%	%	%	%	%
Intervention-based registers	**Spine** (n=48,960)	Level 1	7,397(15.1)	39,123 (**79.9**)	13,691 (28.0)	302 (0.6)	20,660 (42.2)	27,881 (**56.9**)	44,819 (**91.5**)	32,140 (**65.6**)	11,179 (22.8)	30,539 (**62.4**)
		Level 2	40,622 (**83.0**)	9,251 (18.9)	26,059 (**53.2**)	24,076 (49.2)	25,351 (**51.8**)	20,950 (42.8)	3,857 (7.9)	14,637 (29.9)	30,954 (**63.2**)	16,377 (33.4)
		Level 3	941 (1.9)	586 (1.2)	9,210 (18.8)	24,582 (**50.2**)	2,949 (6.0)	129 (0.3)	284 (0.6)	2,183 (4.5)	6,827 (13.9)	2,044 (4.2)
	**Hip** (n=90,660)	Level 1	7,340 (8.1)	70,483 (**77.7**)	36,364 (40.1)	1,400 (1.5)	54,120 (**59.7**)	55,970 (**61.7**)	83,800 (**92.4**)	70,683 (**78.0**)	41,270 (45.5)	71,102 (**78.4**)
		Level 2	83,026 (**91.6**)	19,307 (21.3)	45,371 (**50.0**)	52,519 (**57.9**)	33,597 (37.1)	34,565 (38.1)	6,319 (7.0)	18,161 (20.0)	45,422 (**50.1**)	18,188 (20.1)
		Level 3	294 (0.3)	870 (1.0)	8,925 (9.8)	36,741 (40.5)	2,943 (3.2)	125 (0.1)	541 (0.6)	1,816 (2.0)	3,968 (4.4)	1,370 (1.5)
	**Knee** (n=16,324)	Level 1	1,895 (11.6)	15,241 (**93.4**)	8,719 (**53.4**)	290 (1.8)	10,669 (**65.4**)	10,249 (**62.8**)	15,522 (**95.1**)	12,696 (**77.8**)	5,884 (36.0)	12,830 (**78.6**)
		Level 2	14,388 (**88.1**)	938 (5.7)	6,742 (41.3)	10,328 (**63.3**)	5,261 (32.2)	6,048 (37.0)	710 (4.3)	3,354 (20.5)	9,588 (**58.7**)	3,222 (19.7)
		Level 3	41 (0.3)	145 (0.9)	863 (5.3)	5,706 (35.0)	394 (2.4)	27 (0.2)	92 (0.6)	274 (1.7)	852 (5.2)	272 (1.7)
	**Ankle** (n=700)	Level 1	29 (4.1)	617 (**88.1**)	264 (37.7)	8 (1.1)	404 (**57.7**)	253 (36.1)	641 (**91.6**)	448 (**64.0**)	170 (24.3)	503 (**71.9**)
		Level 2	659 (**94.1**)	76 (10.9)	346 (**49.4**)	351 (**50.1**)	266 (38.0)	443 (**63.3**)	51 (7.3)	223 (31.9)	459 (**65.6**)	180 (25.7)
		Level 3	12 (1.7)	7 (1.0)	90 (12.9)	341 (48.7)	30 (4.3)	4 (0.6)	8 (1.1)	29 (4.1)	71 (10.1)	17 (2.4)
	**Cruciate ligament** (n=8,430)	Level 1	5,645 (**67.0**)	8,209 (**97.4**)	4,503 (**53.4**)	1,294 (15.3)	4,190 (**49.7**)	7,308 (**86.7**)	8,337 (**98.9**)	6,663 (**79.0**)	2,943 (34.9)	5,501 (**65.3**)
		Level 2	2,762 (32.8)	178 (2.1)	3,172 (37.6)	6,658 (**79.0**)	3,779 (44.8)	1,117 (13.3)	69 (0.8)	1,636 (19.4)	5,223 (**62.0**)	2,649 (31.4)
		Level 3	23 (0.3)	43 (0.5)	755 (9.0)	478 (5.7)	461 (5.5)	5 (0.1)	24 (0.3)	131 (1.6)	264 (3.1)	280 (3.3)
	**BOA** (n=13,647)	Level 1	5,781 (42.4)	13,120 (**96.1**)	10,274 (**75.3**)	351 (2.6)	8,981 (**65.8**)	7,096 (**52.0**)	13,093 (**95.9**)	10,880 (**79.7**)	1,264 (9.3)	9,553 (**70.0**)
		Level 2	7,854 (**57.6**)	470 (3.4)	3,200 (23.4)	11,755 (**86.1**)	4,473 (32.8)	6,542 (47.9)	494 (3.6)	2,646 (19.4)	11,278 (**82.6**)	3,930 (28.8)
		Level 3	12 (0.1)	57 (0.4)	173 (1.3)	1,541 (11.3)	193 (1.4)	9 (0.1)	60 (0.4)	121 (0.9)	1,105 (8.1)	164 (1.2)
	**Fracture** (n=50,892)	Level 1	41,022 (**80.6**)	45,512 (**89.4**)	42,073 (**82.7**)	32,363 (**63.6**)	40,462 (**79.5**)	35,314 (**69.4**)	44,852 (**88.1**)	38,161 (**75.0**)	20,558 (40.4)	38,181 (**75.0**)
		Level 2	9,542 (18.7)	4,373 (8.6)	5,815 (11.4)	16,658 (32.7)	9,419 (18.5)	14,970 (29.4)	4,575 (9.0)	9,864 (19.4)	28,059 (**55.1**)	11,566 (22.7)
		Level 3	328 (0.6)	1,007 (2.0)	3,004 (5.9)	1,871 (3.7)	1,011 (2.0)	608 (1.2)	1,465 (2.9)	2,867 (5.6)	2,275 (4.5)	1,145 (2.2)
Diagnosis-based registers	**Heart failure** (n=1,436)	Level 1	866 (**60.3**)	1,315 (**91.6**)	1,041 (**72.5**)	689 (**48.0**)	865 (**60.2**)	837 (**58.3**)	1,298 (**90.4**)	1,025 (**71.4**)	685 (**47.7**)	896 (**62.4**)
		Level 2	567(39.5)	116 (8.1)	355 (24.7)	679 (47.3)	521 (36.3)	593 (41.3)	126 (8.8)	371 (25.8)	655 (45.6)	499 (34.7)
		Level 3	3 (0.2)	5 (0.3)	40 (2.8)	68 (4.7)	50 (3.5)	6 (0.4)	12 (0.8)	40 (2.8)	96 (6.7)	41 (2.9)
	**Respiratory failure** (n=1,050)	Level 1	243 (23.1)	650 (**61.9**)	272 (25.9)	305 (29.0)	432 (41.1)	187 (17.8)	551 (**52.5**)	211 (20.1)	256 (24.4)	387 (36.9)
		Level 2	786 (**74.9**)	343 (32.7)	557 (**53.0**)	600 (**57.1**)	532 (**50.7**)	814 (**77.5**)	409 (39.0)	526 (**50.1**)	627 (**59.7**)	559 (**53.2**)
		Level 3	21 (2.0)	57 (5.4)	221 (21.0)	145 (13.8)	86 (8.2)	49 (4.7)	90 (8.6)	313 (29.8)	167 (15.9)	104 (9.9)
	**Psoriasis** (n=2,680)	Level 1	2,064 (**77.0**)	2,554 (**95.3**)	2,183 (**81.5**)	885 (33.0)	1,318 (**49.2**)	2,151 (**80.3**)	2,578 (**96.2**)	2,329 (86.**9**)	1,231 (45.9)	1,725 (**64.4**)
		Level 2	615 (22.9)	115 (4.3)	454 (16.9)	1,536 (**57.3**)	1,185 (44.2)	526 (19.6)	95 (3.5)	312 (11.6)	1,307(**48.8**)	847 (31.6)
		Level 3	1 (0.0)	11 (0.4)	43 (1.6)	259 (9.7)	177 (6.6)	3 (0.1)	7 (0.3)	39 (1.5)	142 (5.3)	108 (4.0)
	**Rheumatology** (n=31,462)	Level 1	15,883 (**50.5**)	25,394 (**80.7**)	18,304 (**58.2**)	3,891 (12.4)	16,917 (**53.8**)	17,585 (**55.9**)	26,523 (**84.3**)	20,443 (**65.0**)	5,373 (17.1)	18,408 (**58.5**)
		Level 2	15,506 (49.3)	5,718 (18.2)	11,645 (37.0)	22,703 (**72.2**)	13,014 (41.4)	13,776 (43.8)	4,591 (14.6)	9,873 (31.4)	22,206 (**70.6**)	11,553 (36.7)
		Level 3	73 (0.2)	350 (1.1)	1,513 (4.8)	4,868 (15.5)	1,531 (4.9)	101 (0.3)	348 (1.1)	1,146 (3.6)	3,883 (12.3)	1,501 (4.8)
**General population** (n=49,169)		Level 1	44,279 (**90.1**)	48,371 (**98.4**)	44,848 (**91.2**)	24,946 (**50.8**)	32,721 (**66.5**)	-	-	-	-	-
		Level 2	4,840 (9.8)	600 (1.2)	3,785 (7.7)	22,185 (45.1)	15,126 (30.8)	-	-	-	-	-
		Level 3	50 (0.1)	198 (0.4)	536 (1.1)	2038 (4.1)	1,322 (2.7)	-	-	-	-	-

The highest proportion in bold; Level 1: No problems; Level 2: Some/moderate problems; Level 3: confined to bed/unable to/extreme problems; Proportions rounded off to one decimal using MS Excel

Table 3: Mean (SD) EQ VAS score at baseline, 1-year follow-up and the change in the 11 National Quality Registers (NQRs) and in the general population

Time	Variable	Mean EQ VAS score (SD)									
		Intervention-based registers							Diagnosis-based registers		General population
		Spine	Hip	Knee	Ankle	Cruciate ligament	BOA	Fracture	Heart failure	Respiratory failure	
**Baseline**	**Overall**	47.8 (22.1)	56.2 (22.2)	64.6 (22.1)	55.8 (21.4)	62.8 (23.0)	68.3 (18.6)	84.7 (18.4)	65.4 (18.9)	51.1 (21.3)	79.5 (18.3)
	**Sex**										
	Men	50.4 (22.1)	59.6 (21.3)	68.1 (20.9)	58.5 (20.5)	63.9 (23.0)	70.8 (17.4)	86.1 (17.0)	66.5 (18.9)	50.9 (20.2)	80.4 (17.4)
	Women	45.2 (21.8)	53.7 (22.5)	62.0 (22.5)	52.5 (22.2)	61.4 (23.0)	67.3 (18.9)	84.0 (19.0)	62.9 (18.7)	51.2 (22.0)	78.8 (18.9)
	**Age group**										
	<30	46.6 (21.3)	46.2 (24.3)	-	49.9 (17.5)	63.3 (23.1)	71.3 (2.5)	90.3 (13.8)	-	-	81.2 (16.8)
	30-39	45.1 (22.0)	52.2 (22.3)	58.9 (25.6)	45.6 (18.1)	63.4 (22.4)	62.6 (22.2)	88.8 (14.7)	74.0 (20.7)	-	80.9 (17.1)
	40-49	45.9 (21.7)	54.3 (22.5)	56.7 (23.3)	51.4 (21.3)	60.7 (23.5)	63.9 (19.6)	88.3 (15.6)	67.2 (16.6)	-	79.4 (18,3)
	50-59	46.8 (22.3)	55.0 (22.6)	60.6 (23.4)	55.0 (20.7)	60.7 (22.8)	66.1 (19.5)	87.6 (16.3)	62.1 (19.7)	47.4 (27.8)	78.5 (19.2)
	60-69	48.7 (22.2)	56.8 (22.6)	64.5 (22.5)	56.4 (21.8)	59.0 (28.0)	69.3 (18.3)	87.4 (16.4)	69.5 (19.8)	50.0 (21.2)	78.5 (19.4)
	70-79	49.9 (22.2)	57.1 (22.7)	66.3 (21.3)	58.7 (22.0)	-	69.3 (18.0)	83.3 (18.6)	65.2 (19.4)	50.7 (20.0)	76.1 (19.3)
	80+	49.6 (21.7)	54.8 (21.4)	64.8 (20.7)	50.3 (17.6)	-	66.6 (18.1)	71.2 (22.1)	63.2 (16.8)	53.8 (22.5)	71.1 22.3()
	**BMI (Kg/m²)**										
	Underweight	42.6 (22.0)	53.6 (22.1)	62.3 (24.9)	40.7 (29.9)	72.6 (20.6)	64.8 (20.4)	-	-	48.9 (21.6)	78.2 (19.1)
	Normal	48.1 (22.2)	57.9 (22.2)	66.7 (21.9)	56.1 (23.3)	64.8 (23.0)	71.1 (17.9)	-	-	52.5 (21.9)	82.1 (16.1)
	Overweight	48.6 (22.0)	57.6 (21.9)	66.3 (21.7)	57.4 (19.8)	63.7 (22.5)	69.2 (18.2)	-	-	52.0 (19.1)	78.7 (17.9)
	Obesity class I	46.8 (21.7)	54.3 (22.1)	62.4 (21.9)	54.5 (20.9)	59.2 (23.3)	65.1 (19.2)	-	-	51.4 (21.2)	72.9 (20.3)
	Obesity class II	43.8 (22.3)	51.6 (22.8)	58.7 (23.1)	53.0 (19.9)	51.4 (24.2)	63.2 (18.8)	-	-	54.1 (19.2)	68.4 (21.1)
	Obesity class III	44.0 (21.8)	49.4 (23.6)	57.5 (23.5)	40.5 (14.5)	66.2 (20.2)	56.7 (19.3)	-	-	55.6 (17.3)	67.5 (21.9)
**1-year follow-up**	**Overall**	67.9 (22.5)	76.7 (20.0)	76.2 (19.4)	70.2 (18.9)	74.5 (19.9)	70.4 (18.7)	77.7 (20.4)	67.9 (18.8)	49.1 (20.4)	-
	**Sex**										-
	Men	69.4 (22.0)	78.6 (19.0)	77.8 (18.8)	72.3 (17.1)	75.3 (20.1)	70.8 (18.3)	78.7 (19.8)	68.4 (19.0)	49.4 (19.9)	-
	Women	66.5 (22.9)	75.3 (20.7)	75.1 (19.8)	67.6 (20.5)	73.6 (19.5)	70.3 (18.9)	77.2 (20.8)	65.4 (18.2)	48.9 (20.7)	-
	**Age group**										-
	<30	73.2 (20.3)	74.2 (20.1)	-	62.5 (23.7)	74.5 (19.8)	69.5 (28.2)	82.5 (17.1)	-	-	-
	30-39	70.9 (21.7)	77.4 (20.4)	75.5 (20.6)	64.4 (20.4)	74.5 (19.5)	71.7 (20.9)	82.0 (17.4)	76.0 (19.5)	-	-
	40-49	70.5 (22.6)	78.8 (19.5)	73.3 (20.7)	67.3 (17.4)	74.8 (20.4)	69.8 (19.1)	81.2 (17.7)	71.7 (17.4)	-	-
	50-59	68.3 (23.1)	78.5 (19.8)	73.6 (20.8)	69.4 (20.2)	73.4 (21.1)	69.5 (19.7)	80.7 (18.6)	70.5 (18.1)	48.8 (22.7)	-
	60-69	68.5 (22.3)	79.1 (19.4)	78.0 (18.6)	72.6 (17.2)	79.7 (19.1)	71.9 (18.1)	81.3 (18.3)	72.1 (17.8)	47.7 (20.5)	-
	70-79	64.8 (22.3)	75.8 (20.1)	76.5 (19.4)	71.0 (18.6)	-	69.9 (18.5)	76.1 (21.0)	66.6 (18.4)	49.1 (19.6)	-
	80+	60.7 (21.5)	71.1 (20.5)	73.1 (19.6)	58.6 (23.0)	-	64.9 (19.3)	63.1 (23.0)	63.0 (19.5)	50.3 (21.4)	-
	**BMI (Kg/m²)**										-
	Underweight	66.2 (24.0)	72.7 (22.0)	70.3 (16.9)	51.7 (16.1)	70.8 (19.8)	69.4 (14.7)	-	-	44.9 (20.7)	-
	Normal	70.3 (22.2)	78.6 (19.6)	77.9 (18.9)	68.9 (19.2)	75.6 (20.0)	73.9 (17.6)	-	-	49.9 (20.5)	-
	Overweight	68.6 (22.1)	77.7 (19.4)	77.6 (18.9)	72.4 (17.6)	74.6 (20.5)	70.9 (18.4)	-	-	50.9 (20.2)	-
	Obesity class I	64.5 (22.9)	74.5 (20.6)	74.4 (19.7)	67.3 (18.4)	72.1 (20.9)	66.9 (19.3)	-	-	48.5 (20.8)	-
	Obesity class II	60.3 (23.3)	70.9 (21.6)	72.7 (20.2)	69.1 (22.8)	64.8 (25.6)	65.7 (18.9)	-	-	47.2 (16.0)	-
	Obesity class III	61.4 (24.8)	68.1 (23.0)	66.7 (22.6)	39.4 (18.6)	61.8 (27.2)	58.6 (21.1)	-	-	54.3 (15.1)	-

For peer review only

**Change**	**Overall**	20.1 (27.1)	20.5 (25.8)	11.7 (24.6)	14.4 (24.7)	11.8 (26.4)	2.1 (18.7)	-7.0 (19.4)	2.1 (18.3)	-2.0 (24.1)	-
	**Sex**										-
	Men	19.0 (26.7)	19.0 (24.5)	9.7 (23.5)	13.8 (22.5)	11.4 (26.4)	0.0 (17.3)	-7.4 (18.9)	1.9 (18.3)	-1.4 (24.1)	-
	Women	21.2 (26.4)	21.6 (26.7)	13.1 (25.2)	15.1 (27.2)	12.2 (26.3)	3.0 (19.2)	-6.8 (19.6)	2.5 (18.2)	-2.3 (24.2)	-
	**Age group**										-
	<30	26.6 (26.3)	28.0 (29.0)	-	12.6 (32.3)	11.3 (26.6)	-1.8 (27.5)	-7.8 (19.5)	-	-	-
	30-39	25.9 (27.3)	25.2 (25.4)	16.6 (21.3)	18.8 (17.8)	11.2 (24.6)	9.0 (17.9)	-6.8 (18.3)	2.0 (11.0)	-	-
	40-49	24.6 (27.4)	24.5 (26.0)	16.6 (27.4)	15.9 (25.7)	14.1 (27.2)	5.9 (19.0)	-7.1 (18.4)	4.5 (19.2)	-	-
	50-59	21.4 (27.2)	23.5 (25.9)	13.0 (26.0)	14.4 (25.1)	12.7 (27.9)	3.4 (19.2)	-6.9 (18.8)	8.4 (19.1)	1.4 (26.1)	-
	60-69	19.8 (26.7)	22.3 (25.8)	13.6 (24.6)	16.2 (25.0)	20.6 (31.4)	2.6 (18.6)	-6.1 (18.2)	2.6 (17.5)	-2.3 (22.9)	-
	70-79	15.0 (26.1)	18.7 (25.5)	10.2 (23.9)	12.3 (24.3)	-	0.6 (18.4)	-7.2 (19.7)	1.4 (19.2)	-1.6 (23.9)	-
	80+	11.1 (25.8)	16.3 (25.6)	8.4 (23.8)	8.3 (23.3)	-	-1.7 (17.8)	-8.1 (22.3)	-0.3 (16.8)	-3.5 (25.8)	-
	**BMI (Kg/m^2)**										-
	Underweight	23.6 (29.0)	19.1 (26.1)	8.0 (24.2)	11.0 (13.9)	-1.7 (22.0)	4.6 (20.9)	-	-	-4.0 (23.5)	-
	Normal	22.2 (27.4)	20.8 (25.5)	11.2 (23.5)	12.8 (25.9)	10.8 (26.6)	2.8 (17.6)	-	-	-2.6 (24.2)	-
	Overweight	20.0 (26.7)	20.1 (25.3)	11.3 (24.1)	14.9 (23.1)	10.9 (27.2)	1.8 (18.5)	-	-	-1.1 (23.0)	-
	Obesity class I	17.7 (27.2)	20.2 (26.4)	12.0 (25.2)	12.8 (23.9)	12.9 (27.1)	1.8 (20.0)	-	-	-2.9 (24.6)	-
	Obesity class II	16.5 (26.9)	19.3 (27.4)	14.0 (26.7)	16.1 (34.0)	13.4 (28.5)	2.5 (20.3)	-	-	-6.9 (21.0)	-
	Obesity class III	17.3 (28.7)	18.7 (28.6)	9.2 (28.5)	-1.1 (17.0)	-4.3 (21.1)	1.9 (18.6)	-	-	-1.3 (13.6)	-

For peer review only - http://bmjopen.bmj.com/site/about/guidelines.xhtml

Table 4: Number of health states reported and mean (SD) EQ VAS score by diagnosis, at baseline, 1-year follow-up and the change in the 11 National Quality Registers (NQRs) and in the general population

Patient group in NQRs		Diagnosis	n*	Number of health states		Mean EQ VAS score (SD)		
		Category		Baseline	1-year follow-up	Baseline	1-year follow-up	Change
Intervention-based registers	Spine	Disc hernia	12,582	140	125	45.2 (22.4)	72.1 (21.1)	+26.9 (27.6)
		Other spinal conditions	1,431	85	69	45.8 (22.5)	66.9 (22.8)	+21.1 (26.9)
		Segmental instability	4,290	107	91	46.4 (20.9)	68.4 (23.2)	+22.0 (26.1)
		Spinal stenosis	28,194	145	148	49.2 (22.0)	65.8 (22.7)	+16.6 (26.4)
		Spondylolysis	2,463	91	81	48.8 (21.6)	69.1 (22.6)	+20.3 (26.7)
	Hip	Avascular necrosis	1,594	71	83	49.0 (24.1)	72.3 (22.4)	+23.3 (28.7)
		Childhood hip disorder	1,846	72	71	55.5 (22.6)	79.5 (19.3)	+24.0 (26.3)
		Hip osteoarthritis	86,023	166	179	56.5 (22.1)	76.9 (19.9)	+20.3 (25.7)
		Inflammatory joint disorder	1,197	71	82	47.7 (22.6)	68.4 (22.2)	+20.8 (27.6)
	Knee	Knee osteoarthritis	16,020	106	122	64.7 (22.1)	76.4 (19.5)	+11.7 (24.5)
		Knee rheumatoid arthritis	304	40	40	55.8 (21.8)	67.8 (21.4)	+12.0 (25.7)
	Ankle	Others	85	28	21	58.3 (22.1)	68.7 (18.7)	+10.4 (22.7)
		Posttraumatic OA	308	39	40	55.9 (20.7)	70.1 (19.7)	+14.2 (25.3)
		Primary OA	234	33	26	58.0 (21.2)	73.6 (17.2)	+15.6 (24.0)
		Rheumatoid arthritis	73	22	23	45.5 (21.9)	61.8 (18.2)	+16.4 (26.4)
	Cruciate ligament	Cruciate ligament injury	8,430	106	83	62.8 (23.0)	74.5 (19.9)	+11.8 (26.4)
	BOA	BOA hip	3,440	67	62	67.5 (18.5)	68.3 (19.0)	+0.7 (18.5)
		BOA knee	10,207	87	90	68.6 (18.6)	71.2 (18.5)	+2.6 (18.7)
	Fracture	Ankle and foot	3,718	97	91	87.2 (16.1)	81.4 (18.4)	-5.8 (18.8)
		Elbow and forearm	13,996	138	148	87.5 (15.8)	81.9 (18.1)	-5.6 (17.2)
		Hip and femur	8,378	161	157	74.0 (22.7)	64.5 (23.4)	-9.5 (23.3)
		Knee and lower leg	10,866	140	142	86.9 (16.8)	78.7 (19.0)	-8.1 (19.4)
		Lumbar, spine and pelvis	1,128	89	81	76.9 (22.4)	69.5 (23.5)	-7.4 (21.6)
		Shoulder and upper arm	7,835	140	140	84.7 (18.3)	77.8 (19.8)	-6.9 (19.7)
		Unspecified part of trunk, extremities or other body region	749	69	65	83.6 (19.0)	70.6 (21.9)	-13.0 (21.6)
		Wrist and hand	4,222	96	98	88.1 (15.3)	83.3 (17.4)	-4.8 (17.0)
Diagnosis-based registers	Heart failure	Heart failure	1,436	75	84	65.4 (18.9)	67.4 (18.8)	+2.1 (18.3)
	Respiratory failure	COPD	895	98	102	50.7 (21.3)	49.1 (20.4)	-1.6 (23.7)
		Pulmonary fibrosis	149	48	50	53.2 (21.2)	48.9 (20.6)	-4.3 (26.4)
	Psoriasis	Psoriasis vulgaris	1,704	68	60	-	-	-
		Psoriasis vulgaris + anthropathic	537	52	51	-	-	-
		Psoriasis unspecified	439	46	40	-	-	-
	Rheumatology	Rheumatoid arthritis	17,870	150	153			
		Spondyloarthropathy	8,782	115	116			
		Inflammatory systemic disorder	1,614	87	86	-	-	-
		Juvenile rheumatoid diseases	1,088	75	72	-	-	-
		Vasculitis	410	49	47	-	-	-
		Other arthritis	1,698	80	73	-	-	-
General population			49,169	156	-	79.5	-	-

*Sample size used to calculate the number of health states, EQ VAS scores are calculated based on subsets of observations with non-missing EQ VAS scores

No data available on EQ VAS in the Psoriasis and Rhematolgy data sets

For peer review only

For peer review only - http://bmjopen.bmj.com/site/about/guidelines.xhtml

Table 5: Mean (SD) EQ VAS score by Paretian Classification of Health Change (PCHC), by diagnosis in the 11 National Quality Registers (NQRs)

PCHC	Spine (n=44,196)									
	Disc hernia		Other spinal conditions		Segmental instability		Spinal stenosis		Spondylolysis	
	Baseline Mean (SD)	1-year follow-up Mean (SD)	Baseline Mean (SD)	1-year follow-up Mean (SD)	Baseline Mean (SD)	1-year follow-up Mean (SD)	Baseline Mean (SD)	1-year follow-up Mean (SD)	Baseline Mean (SD)	1-year follow-up Mean (SD)
No change	54.0 (22.0)	58.5 (22.8)	50.2 (23.8)	57.9 (24.3)	50.0 (21.8)	59.2 (24.2)	53.7 (21.7)	58.1 (21.8)	53.7 (21.6)	60.2 (20.7)
Improved	44.3 (22.5)	76.7 (18.1)	44.7 (22.4)	72.5 (20.3)	46.2 (20.7)	75.3 (19.0)	48.4 (22.2)	72.8 (19.5)	48.1 (21.4)	75.9 (18.7)
Worsened	51.0 (21.1)	44.8 (21.6)	49.0 (21.7)	46.4 (22.2)	45.6 (21.4)	40.2 (21.6)	50.7 (21.3)	44.1 (21.5)	51.9 (22.0)	43.9 (23.2)
Mixed	43.7 (21.2)	56.4 (20.8)	45.9 (22.7)	56.8 (20.1)	45.4 (20.6)	54.9 (21.0)	47.2 (21.5)	53.6 (20.4)	45.7 (21.4)	53.4 (21.1)

PCHC	Hip (n=91,658)								Knee (n=16,324)	
	Avascular necrosis		Childhood hip disorder		Hip osteoarthritis		Inflammatory joint disorder		Knee osteoarthritis	
	Baseline Mean (SD)	1-year follow-up Mean (SD)	Baseline Mean (SD)	1-year follow-up Mean (SD)	Baseline Mean (SD)	1-year follow-up Mean (SD)	Baseline Mean (SD)	1-year follow-up Mean (SD)	Baseline Mean (SD)	1-year follow-up Mean (SD)
No change	55.9 (22.3)	63.3 (23.0)	58.9 (23.0)	67.1 (24.0)	61.0 (20.3)	68.6 (20.0)	51.9 (20.8)	59.6 (21.6)	68.0 (20.5)	71.4 (19.4)
Improved	48.2 (24.3)	76.3 (20.2)	55.3 (22.7)	83.4 (15.8)	56.3 (22.3)	81.1 (17.0)	46.7 (22.7)	73.8 (20.0)	64.7 (22.3)	80.9 (16.3)
Worsened	51.1 (23.7)	48.3 (24.0)	55.3 (21.6)	50.5 (23.2)	58.6 (20.4)	52.8 (21.9)	55.0 (22.5)	48.7 (22.9)	65.5 (21.1)	57.3 (22.2)
Mixed	52.3 (22.9)	58.6 (22.5)	56.1 (22.6)	64.2 (21.1)	53.5 (21.5)	61.5 (21.2)	45.5 (21.5)	56.9 (20.8)	60.8 (22.3)	63.4 (21.3)

PCHC	Knee (n=16,324)		Ankle (n=668)							
	Knee rheumatoid arthritis		Other		Posttraumatic osteoarthritis		Primary osteoarthritis		Rheumatoid arthritis	
	Baseline Mean (SD)	1-year follow-up Mean (SD)	Baseline Mean (SD)	1-year follow-up Mean (SD)	Baseline Mean (SD)	1-year follow-up Mean (SD)	Baseline Mean (SD)	1-year follow-up Mean (SD)	Baseline Mean (SD)	1-year follow-up Mean (SD)
No change	57.4 (21.2)	60.6 (20.4)	65.0 (18.7)	62.9 (22.9)	59.7 (17.0)	60.9 (20.5)	64.8 (19.5)	70.1 (17.1)	45.7 (16.4)	58.0 (22.5)
Improved	56.3 (22.4)	73.4 (19.1)	56.6 (22.6)	72.1 (16.5)	53.6 (21.3)	75.6 (16.6)	55.4 (21.5)	77.8 (14.7)	44.1 (23.2)	68.5 (15.5)
Worsened	57.7 (17.1)	54.8 (20.6)	63.8 (18.5)	58.2 (18.5)	58.1 (22.2)	51.2 (19.7)	71.9 (12.8)	59.5 (13.9)	45.0 (26.2)	34.6 (8.7)
Mixed	49.6 (21.7)	56.8 (22.8)	54.8 (28.9)	66.7 (27.1)	62.2 (18.0)	66.4 (20.1)	52.8 (21.4)	58.9 (23.9)	50.7 (19.5)	50.1 (10.5)

PCHC	Cruciate ligament (n=8,155)		BOA (n=6,690)				Fracture (n=40,787)			
			BOA hip		BOA knee		Ankle and foot		Elbow and forearm	
	Baseline Mean (SD)	1-year follow-up Mean (SD)	Baseline Mean (SD)	1-year follow-up Mean (SD)	Baseline Mean (SD)	1-year follow-up Mean (SD)	Baseline Mean (SD)	1-year follow-up Mean (SD)	Baseline Mean (SD)	1-year follow-up Mean (SD)
No change	69.2 (21.7)	76.0 (18.8)	71.8 (17.4)	72.4 (16.7)	72.8 (17.5)	74.4 (16.6)	91.1 (12.1)	88.9 (14.1)	91.5 (11.7)	88.7 (14.1)
Improved	60.2 (22.8)	78.6 (17.5)	64.9 (19.8)	72.9 (18.3)	66.5 (19.1)	75.1 (16.9)	78.5 (19.8)	81.9 (17.5)	78.8 (19.5)	81.2 (17.9)
Worsened	67.0 (22.6)	62.4 (22.2)	66.4 (17.7)	58.1 (18.8)	67.9 (17.8)	60.6 (19.8)	89.1 (14.6)	74.7 (19.4)	88.7 (14.6)	76.5 (18.7)
Mixed	58.5 (23.7)	64.1 (21.7)	63.7 (16.9)	61.3 (18.4)	61.1 (18.2)	60.7 (18.4)	72.7 (20.2)	67.6 (20.3)	71.6 (20.7)	65.7 (20.2)

PCHC	Fracture (n=40,787)									
	Hip and femur		Knee and lower leg		Lumbar, spine and pelvis		Shoulder and upper arm		Unspecified	
	Baseline Mean (SD)	1-year follow-up Mean (SD)	Baseline Mean (SD)	1-year follow-up Mean (SD)	Baseline Mean (SD)	1-year follow-up Mean (SD)	Baseline Mean (SD)	1-year follow-up Mean (SD)	Baseline Mean (SD)	1-year follow-up Mean (SD)
No change	81.8 (20.3)	77.7 (21.9)	90.6 (13.2)	88.1 (14.6)	86.6 (16.4)	82.3 (19.7)	89.7 (14.0)	86.8 (15.9)	87.9 (15.9)	83.6 (17.8)
Improved	62.3 (24.4)	68.4 (21.4)	75.7 (21.7)	78.9 (19.2)	66.4 (25.6)	70.3 (21.9)	75.4 (22.3)	78.9 (18.9)	76.8 (21.9)	72.9 (18.8)
Worsened	78.1 (20.3)	60.6 (22.7)	88.9 (14.6)	73.8 (18.6)	80.0 (19.1)	63.3 (22.1)	86.4 (16.6)	72.7 (19.9)	86.3 (16.0)	66.4 (21.6)
Mixed	60.7 (22.3)	56.4 (21.9)	71.8 (21.9)	64.9 (21.3)	55.7 (22.1)	52.8 (23.7)	69.1 (21.7)	65.2 (20.4)	66.6 (26.5)	59.0 (24.3)

PCHC	Fracture (n=40,787)		Heart failure (n=1,044)		Respiratory failure (n=725)			
	Wrist and hand				COPD		Pulmonary fibrosis	
	Baseline Mean (SD)	1-year follow-up Mean (SD)	Baseline Mean (SD)	1-year follow-up Mean (SD)	Baseline Mean (SD)	1-year follow-up Mean (SD)	Baseline Mean (SD)	1-year follow-up Mean (SD)
No change	91.6 (11.1)	89.4 (12.4)	71.7 (17.5)	73.7 (17.5)	53.8 (21.6)	52.2 (20.1)	59.6 (15.2)	54.7 (13.7)
Improved	80.6 (19.5)	83.2 (17.4)	60.2 (18.3)	68.9 (16.9)	48.0 (19.6)	54.4 (18.7)	52.8 (21.0)	64.7 (18.0)
Worsened	89.8 (13.6)	78.4 (19.0)	64.9 (19.9)	60.2 (19.7)	51.3 (21.1)	43.8 (20.2)	51.1 (23.5)	44.6 (22.6)
Mixed	74.4 (22.1)	70.5 (20.2)	61.2 (17.1)	62.9 (17.9)	50.2 (23.0)	51.0 (20.5)	53.0 (20.4)	41.0 (14.7)

For peer review only - http://bmjopen.bmj.com/site/about/guidelines.xhtml

Table 6: Mean (SD) EQ VAS score in selected health states across diagnoses, at baseline in the 11 National Quality Registers (NQRs) and in the general population

Health state*	Mean (SD)							
	Spine					Hip		
	Disc hernia	Other spinal conditions	Segmental instability	Spinal stenosis	Spondylolysis	Avascular necrosis	Childhood hip disorder	Hip osteoarthritis
11111	76.6 (18.9)	86.0 (8.2)	91.7 (2.9)	77.5 (18.3)	79.9 (7.1)	60.3 (13.1)	82.2 (18.8)	76.1 (21.1)
11121	69.7 (16.4)	68.4 (20.0)	66.7 (16.9)	71.2 (16.5)	66.5 (18.9)	73.8 (21.3)	73.0 (18.7)	73.6 (17.2)
21121	63.5 (17.0)	63.4 (18.3)	60.9 (17.8)	64.2 (17.4)	63.2 (18.4)	67.2 (18.5)	66.4 (17.9)	67.7 (17.8)
21122	56.9 (16.9)	55.5 (16.1)	52.9 (17.5)	56.4 (17.1)	56.9 (17.0)	59.9 (17.7)	63.4 (15.8)	59.8 (16.9)
21221	57.4 (17.2)	54.5 (17.5)	55.2 (17.3)	57.5 (17.8)	56.7 (18.2)	60.8 (18.3)	62.9 (18.8)	61.9 (18.0)
21222	50.6 (17.0)	49.9 (16.4)	48.0 (16.3)	49.9 (16.6)	47.8 (16.8)	57.6 (17.9)	54.4 (17.8)	54.2 (16.9)
21232	40.8 (19.3)	38.4 (19.5)	39.3 (19.0)	39.5 (19.8)	40.0 (18.5)	44.3 (20.9)	46.7 (20.5)	45.1 (20.6)
22222	47.3 (18.1)	38.2 (16.7)	46.5 (17.7)	44.8 (17.5)	45.4 (13.2)	54.4 (20.3)	47.6 (18.1)	51.7 (17.0)
33333	12.3 (19.0)	9.8 (8.4)	-	18.1 (27.6)	-	15.6 (14.6)	-	27.0 (30.3)

Health state*	Mean (SD)							
	Hip	Knee		Ankle				Cruciate ligament
	Inflammatory joint disorder	Knee osteoarthritis	Knee rheumatoid arthritis	Other	Posttraumatic osteoarthritis	Primary osteoarthritis	Rheumatoid arthritis	
11111	84.4 (13.8)	81.7 (15.7)	-	-	-	-	-	78.3 (18.2)
11121	65.2 (23.5)	80.8 (15.2)	77.0 (10.7)	65.0 (32.8)	74.4 (18.1)	81.7 (17.6)	-	72.8 (19.4)
21121	62.1 (19.1)	73.2 (17.9)	70.5 (17.1)	74.2 (12.2)	67.1 (17.4)	72.5 (14.2)	56.4 (17.3)	64.1 (20.7)
21122	57.0 (16.2)	63.9 (17.8)	60.8 (13.9)	-	59.4 (18.7)	70.0 (7.1)	-	54.5 (21.9)
21221	53.8 (17.4)	67.3 (18.5)	60.9 (16.0)	69.2 (18.6)	57.0 (16.8)	61.5 (18.2)	62.0 (20.2)	60.6 (22.5)
21222	49.3 (17.4)	57.9 (18.4)	57.0 (14.9)	60.0 (18.3)	56.7 (14.4)	58.5 (11.6)	54.7 (13.1)	51.9 (20.7)
21232	44.4 (20.7)	49.5 (21.6)	42.8 (18.7)	49.8 (17.6)	47.2 (19.7)	50.4 (19.7)	26.0 (4.9)	43.4 (22.5)
22222	52.4 (19.9)	50.7 (18.2)	39.8 (12.8)	-	-	-	-	46.6 (20.1)
33333	-	-	-	-	-	-	-	-

Health state*	Mean (SD)							
	BOA		Fracture					
	BOA hip	BOA knee	Ankle and foot	Elbow and forearm	Hip and femur	Knee and lower leg	Lumbar, spine and pelvis	Shoulder and upper arm
11111	78.9 (20.6)	88.5 (9.7)	93.2 (9.7)	93.5 (9.3)	91.1 (11.7)	92.8 (10.6)	92.3 (10.6)	92.3 (11.2)
11121	77.9 (13.9)	78.2 (14.5)	84.5 (11.9)	84.4 (12.7)	79.7 (14.0)	84.1 (12.8)	81.8 (11.9)	83.3 (14.8)
21121	71.2 (15.7)	71.1 (16.3)	78.5 (19.1)	74.6 (14.6)	72.4 (16.5)	77.5 (17.2)	73.1 (13.2)	73.1 (15.6)
21122	62.1 (15.5)	63.2 (16.5)	72.3 (19.1)	66.2 (15.1)	62.5 (17.6)	68.3 (17.5)	60.4 (17.6)	63.9 (15.6)
21221	60.4 (18.3)	65.0 (15.0)	76.5 (22.6)	65.5 (16.8)	66.0 (17.8)	74.0 (20.6)	60.8 (15.6)	65.5 (15.3)
21222	54.2 (16.2)	54.7 (16.2)	70.5 (20.8)	59.1 (16.3)	59.1 (16.8)	62.4 (20.1)	59.4 (17.1)	58.5 (17.7)
21232	46.5 (18.2)	50.6 (19.6)	56.2 (24.6)	48.6 (21.8)	40.8 (16.1)	52.7 (23.2)	59.8 (14.4)	46.3 (18.5)
22222	49.8 (17.5)	52.0 (17.3)	62.0 (20.3)	56.4 (16.1)	52.7 (18.2)	62.9 (23.2)	52.3 (17.0)	55.6 (21.8)
33333	-	-	-	-	25.0 (22.0)	41.3 (22.5)	-	39.3 (27.2)

Health state*	Mean (SD)							
	Fracture		Heart failure	Respiratory failure		General population		
	Unspecified	Wrist and hand		COPD	Pulmonary fibrosis			
11111	92.5 (10.6)	93.3 (8.9)	76.4 (15.7)	66.7 (25.4)	76.1 (10.8)	88.8 (9.7)		
11121	83.1 (12.4)	84.9 (13.5)	72.1 (16.4)	61.1 (24.3)	-	82.5 (11.6)		
21121	67.7 (19.4)	72.4 (17.2)	66.8 (13.2)	62.0 (15.4)	50.0 (15.0)	72.0 (17.0)		
21122	63.1 (18.9)	69.4 (13.2)	57.1 (16.7)	63.9 (24.0)	60.8 (17.0)	59.7 (18.2)		
21221	68.2 (20.5)	69.3 (20.6)	57.7 (14.7)	52.5 (19.3)	64.0 (18.2)	59.8 (18.7)		
21222	64.6 (18.9)	59.5 (24.7)	52.0 (15.2)	47.5 (14.5)	56.3 (15.1)	51.7 (17.7)		
21232	-	40.6 (22.3)	42.7 (14.9)	51.4 (26.1)	-	38.4 (16.5)		
22222	62.5 (18.5)	57.2 (19.3)	51.5 (24.0)	50.2 (18.2)	50.7 (15.9)	44.4 (17.3)		
33333	-	-	-	-	-	36.7 (45.9)		

As an example, the health state (health profile) 21232 shows that mobility=level 2 (some problems), self-care=level 1 (no problems), usual activities=level 2 (some problems), pain/discomfort=level 3 (severe problems) and anxiety/depression=level 2 (some problems). *Beside health states 11111 and 33333, the health states selected were among the most frequently reported in many of the patient groups

For peer review only - http://bmjopen.bmj.com/site/about/guidelines.xhtml

Table 7: Mean (SD) EQ VAS score in selected health states across diagnoses, at 1-year follow-up in the 11 National Quality Registers (NQRs)

Health state*	Mean (SD)							
	Spine					Hip		
	Disc hernia	Other spinal conditions	Segmental instability	Spinal stenosis	Spondylolysis	Avascular necrosis	Childhood hip disorder	Hip osteoarthritis
11111	89.1 (10.4)	88.6 (12.3)	89.8 (10.3)	89.2 (10.7)	90.0 (9.3)	89.5 (13.0)	90.5 (9.9)	89.9 (11.4)
11121	80.4 (12.0)	78.7 (13.1)	79.6 (11.9)	79.0 (12.6)	79.6 (12.3)	81.2 (12.7)	82.6 (11.4)	81.5 (12.3)
21121	71.3 (14.9)	66.8 (15.8)	67.8 (16.8)	68.9 (14.6)	72.9 (14.1)	71.9 (16.1)	74.4 (14.9)	73.4 (14.8)
21122	62.7 (14.4)	62.1 (12.9)	62.2 (16.5)	59.5 (15.2)	61.2 (13.5)	63.8 (14.3)	68.2 (16.4)	63.4 (15.4)
21221	61.3 (16.8)	59.7 (16.1)	61.8 (15.5)	59.0 (15.7)	59.2 (14.7)	64.0 (16.7)	65.2 (16.8)	64.0 (16.0)
21222	53.4 (15.1)	52.1 (13.5)	53.0 (16.2)	52.1 (15.0)	53.5 (15.4)	54.8 (16.9)	57.0 (16.9)	55.2 (15.6)
21232	40.5 (17.1)	39.7 (18.1)	40.8 (16.4)	38.7 (17.9)	38.6 (13.6)	36.9 (24.1)	43.3 (24.2)	40.6 (17.4)
22222	49.3 (15.3)	43.2 (15.1)	51.8 (13.5)	47.7 (16.0)	44.5 (18.6)	52.0 (16.2)	56.5 (17.8)	51.5 (16.7)
33333	26.3 (38.1)	-	-	14.5 (17.4)	-	-	-	26.3 (31.6)

Health state*	Mean (SD)							
	Hip	Knee		Ankle				Cruciate ligament
	Inflammatory joint disorder	Knee osteoarthritis	Knee rheumatoid arthritis	Others	Posttraumatic osteoarthritis	Primary osteoarthritis	Rheumatoid arthritis	
11111	88.7 (11.6)	88.9 (11.9)	86.4 (14.0)	82.8 (8.9)	84.4 (10.6)	84.5 (11.2)	69.0 (12.4)	85.5 (14.4)
11121	79.2 (12.6)	82.0 (12.4)	80.0 (15.1)	78.6 (9.4)	82.2 (8.6)	84.5 (8.1)	80.6 (8.6)	78.9 (15.1)
21121	73.8 (13.6)	73.7 (14.7)	70.9 (12.5)	76.9 (8.3)	71.7 (18.5)	74.9 (14.0)	71.3 (15.1)	71.4 (17.3)
21122	64.5 (14.7)	63.5 (16.1)	61.0 (17.1)	58.8 (16.5)	70.4 (10.2)	67.8 (10.0)	-	59.8 (20.6)
21221	61.5 (17.8)	66.8 (16.5)	57.9 (17.1)	59.0 (28.1)	67.6 (14.9)	70.2 (13.5)	57.9 (14.1)	64.6 (17.7)
21222	54.9 (16.2)	57.1 (15.9)	60.9 (18.1)	60.0 (14.1)	60.4 (12.1)	58.9 (18.0)	57.1 (15.7)	55.8 (18.7)
21232	33.9 (19.7)	45.5 (20.7)	42.0 (13.0)	33.3 (2.9)	46.8 (27.6)	53.1 (16.5)	-	42.7 (19.0)
22222	49.4 (14.0)	52.0 (17.3)	57.9 (18.6)	56.3 (18.9)	-	56.8 (6.3)	55.0 (5.0)	41.7 (18.9)
33333	-	39.8 (42.0)	-	-	-	-	-	-

Health state*	Mean (SD)							
	BOA		Fracture					
	BOA hip	BOA knee	Ankle and foot	Elbow and forearm	Hip and femur	Knee and lower leg	Lumbar, spine and pelvis	Shoulder and upper arm
11111	89.5 (11.4)	89.1 (11.4)	90.3 (11.9)	91.0 (11.5)	88.3 (13.1)	90.1 (12.5)	88.7 (12.1)	90.6 (11.7)
11121	79.5 (11.9)	80.1 (12.3)	85.5 (11.6)	84.3 (12.8)	82.5 (12.6)	84.5 (12.1)	82.6 (12.6)	83.2 (12.8)
21121	66.8 (15.2)	70.1 (15.0)	77.7 (15.4)	72.0 (16.0)	73.4 (14.0)	76.7 (13.8)	70.7 (18.1)	72.0 (14.6)
21122	59.8 (14.1)	60.1 (14.2)	65.0 (16.0)	63.4 (16.7)	65.1 (14.9)	67.4 (14.7)	61.8 (16.6)	63.3 (16.4)
21221	60.7 (17.1)	61.3 (15.6)	69.2 (15.9)	62.7 (16.3)	62.2 (15.4)	70.0 (15.0)	61.9 (16.0)	67.7 (15.5)
21222	53.5 (13.8)	53.3 (15.2)	60.2 (16.7)	56.9 (14.1)	55.3 (15.9)	59.8 (16.2)	59.0 (17.7)	56.7 (13.6)
21232	42.8 (15.6)	44.9 (20.3)	46.4 (19.5)	46.5 (14.7)	44.8 (18.5)	47.4 (14.4)	37.8 (20.5)	46.4 (15.9)
22222	50.6 (16.5)	50.4 (11.3)	56.9 (17.2)	51.4 (17.2)	50.6 (15.7)	51.6 (17.4)	47.8 (11.3)	49.3 (15.0)
33333	-	-	-	-	11.6 (8.1)	13.7 (11.8)	-	9.9 (7.6)

Health state*	Mean (SD)							
	Fracture		Heart failure	Respiratory failure		General population		
	Unspecified	Wrist and hand		COPD	Pulmonary fibrosis			
11111	88.4 (12.6)	90.7 (11.2)	78.5 (14.3)	62.3 (25.4)	71.7 (7.8)	88.8 (9.7)		
11121	82.3 (12.6)	85.8 (13.1)	75.3 (11.5)	72.9 (14.8)	78.3 (12.6)	82.5 (11.6)		
21121	70.9 (12.8)	73.4 (14.8)	69.8 (14.8)	66.9 (11.3)	50.0 (35.4)	72.0 (17.0)		
21122	64.0 (15.5)	67.1 (11.6)	59.5 (16.1)	51.6 (25.3)	-	59.7 (18.2)		
21221	64.4 (13.3)	63.4 (15.7)	59.8 (16.6)	51.9 (17.7)	58.6 (15.8)	59.8 (18.7)		
21222	52.9 (12.6)	67.8 (15.3)	54.4 (14.6)	49.4 (15.6)	49.5 (7.6)	51.7 (17.7)		
21232	57.1 (19.3)	48.8 (15.9)	55.6 (14.7)	45.8 (6.2)	-	38.4 (16.5)		
22222	49.5 (11.7)	47.1 (10.7)	42.0 (20.5)	43.3 (16.9)	48.8 (11.8)	44.4 (17.3)		
33333	-	-	-	-	-	36.7 (45.9)		

As an example, the health state (health profile) 21232 shows that mobility=level 2 (some problems), self-care=level 1 (no problems), usual activities=level 2 (some problems), pain/discomfort=level 3 (severe problems) and anxiety/depression=level 2 (some problems). *The same health states as those selected for the baseline in Table 6 are presented here

For peer review only

Figure 1: Proportion of patients by Paretian Classification of Health Change (PCHC), by diagnosis in the 11 National Quality Registers (NQRs)

Figure 2: Two-level random intercept model estimates of EQ VAS score by diagnosis adjusted for age and sex at baseline and 1-year in the 11 National Quality Registers (NQRs) [reference group: general population]

For peer review only - http://bmjopen.bmj.com/site/about/guidelines.xhtml

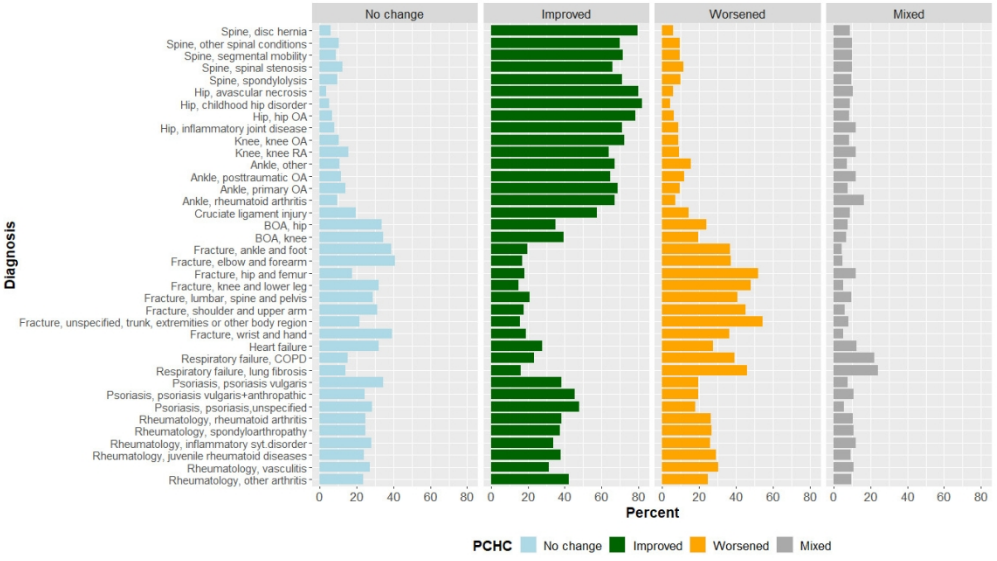

Figure 1: Proportion of patients by Paretian Classification of Health Change (PCHC), by diagnosis in the 11 National Quality Registers (NQRs)

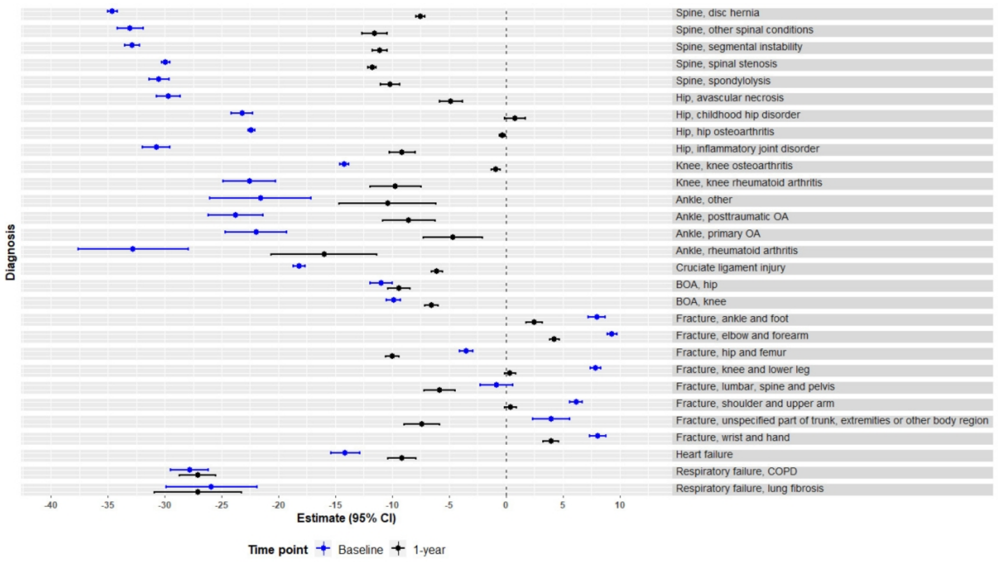

Figure 2: Two-level random intercept model estimates of EQ VAS score by diagnosis adjusted for age and sex at baseline and 1-year in the 11 National Quality Registers (NQRs) [reference group: general population]

Table S1: Sampling procedure followed in combining the data sets from each of the 11 National Quality Registers (NQRs), baseline to 1-year follow-up and the general population data

Category	Patient groups in NQRs	A	B	C	D	E
		n (%)	n (%)	n (%)	n (%)	n (%)
Intervention-based registers	Spine	53,418 (15.8)	53,015 (15.9)	51,047 (15.7)	48,960 (15.5)	44,196 (17.6)
	Hip	95,887 (22.3)	93,684 (28.0)	90,669 (27.9)	90,660 (28.7)	90,658 (36.1)
	Knee	17,600 (5.2)	16,324 (4.9)	16,324 (5.0)	16,324 (5.2)	16,324 (6.5)
	Ankle	794 (0.2)	789 (0.2)	725 (0.2)	700 (0.2)	668 (0.3)
	Cruciate ligament	10,532 (3.1)	10,532 (3.2)	10,465 (3.2)	8,430 (2.7)	8,155 (3.2)
	Osteoarthritis (BOA)	15,238 (4.5)	14,877 (4.5)	13,965 (4.3)	13,647 (4.3)	6,690 (2.7)
	Fracture	55,459 (16.4)	55,428 (16.6)	54,108 (16.6)	50,892 (16.1)	40,787 (16.2)
Diagnosis-based registers	Heart failure	1,789 (0.5)	1,746 (0.5)	1,691 (0.5)	1,436 (0.5)	1,044 (0.4)
	Respiratory failure	1,287 (0.4)	1,063 (0.3)	1,050 (0.3)	1,050 (0.3)	725 (0.3)
	Psoriasis	3,041 (0.9)	2,878 (0.9)	2,789 (0.9)	2,680 (0.8)	-
	Rheumatology	34,036 ()	33,686 (10.1)	32,565 (10.0)	31,462 (10.0)	-
NQRs (total, count)		289,081 (85.4)	284,022 (85.0)	275,398 (84.6)	266,241 (84.4	209,247 (83.4)
General population data		49,499 (14.6)	49,999 (15.0)	49,999 (15.4)	49,169 (15.6	41,761 (16.6)
Total		338,580 (100.0)	334,021 (100.0)	325,397 (100.0)	315,410 (100.0)	251,008 (100.0)

A= Data on baseline and 1-year follow-up
B= After selection of data with information on diagnosis and intervention
C= After de-duplication of records occurring in more than one patient group (duplicate records taken out keeping one record)
D= Included in the analyses after exclusion of missing values on age at baseline, or on any of the EQ-5D dimensions
E= Included in the analyses of EQ VAS values
BOA=Better management of osteoarthritis register, referred to as BOA in whole manuscript
Proportions rounded off to one decimal using MS Excel

For peer review only

Table S2: Demographic characteristics of patients across diagnoses in the 11 National Quality Registers (NQRs)

Patient group in NQRs		Diagnosis	Age (years) Mean (SD)	Age group							Sex
				<30	30-39	40-49	50-59	60-69	70-79	80+	Women
				n (%)	n (%)	n (%)	n (%)	n (%)	n (%)	n (%)	n (%)
Intervention-based registers	Spine	Disc hernia (n=12,582)	46.1 (13.5)	1,216 (9.7)	3,089 (24.6)	3,708 (29.5)	2,312 (18.4)	1,519 (12.1)	624 (5.0)	114 (0.9)	5,784 (46.0)
		Other spinal conditions (n=1,431)	58.2 (13.1)	32 (2.2)	98 (6.8)	207 (14.5)	382 (26.7)	411 (28.7)	260 (18.2)	41 (2.9)	891 (62.3)
		Segmental instability (n=4,290)	45.6 (10.2)	245 (5.7)	932 (21.7)	1,674 (39.0)	1,034 (24.1)	345 (8.0)	59 (1.4)	1 (0.0)	2,326 (54.2)
		Spinal stenosis (n=28,194)	67.2 (10.5)	56 (0.2)	245 (0.9)	1,390 (4.9)	4,518 (16.0)	9,101 (32.3)	9,914 (35.2)	2,970 (10.5)	15,046 (53.4)
		Spondylolysis (n=2,463)	50.9 (12.5)	125 (5.1)	288 (11.7)	716 (29.1)	718 (29.2)	459 (18.6)	142 (5.8)	15 (0.6)	1,281 (52.0)
	Hip	Avascular necrosis (n=1,594)	66.0 (13.6)	13 (0.8)	68 (4.3)	127 (8.0)	228 (14.3)	444 (27.9)	458 (28.7)	256 (16.1)	935 (58.7)
		Childhood hip disorder (n=1,846)	54.5 (12.4)	23 (1.2)	169 (9.2)	492 (26.7)	533 (28.9)	390 (21.1)	199 (10.8)	40 (2.2)	1,139 (61.7)
		Hip osteoarthritis (n=86,023)	68.9 (9.9)	61 (0.1)	348 (0.4)	2,706 (3.1)	10,814 (12.6)	29,020 (33.7)	30,868 (35.9)	12,206 (14.2)	48,329 (56.2)
		Inflammatory joint disorder (n=1,197)	64.5 (12.5)	25 (2.1)	40 (3.3)	69 (5.8)	191 (16.0)	431 (36.0)	344 (28.7)	97 (8.1)	860 (71.8)
	Knee	Knee osteoarthritis (n=16,020)	69.0 (8.7)	0 (0.0)	9 (0.1)	262 (1.6)	2085 (13.0)	5,695 (35.5)	6,203 (38.7)	1,766 (11.0)	9,173 (57.3)
		Knee rheumatoid arthritis (n=304)	66.2 (10.2)	1 (0.3)	1 (0.3)	16 (5.3)	51 (16.8)	119 (39.1)	93 (30.6)	23 (7.6)	219 (72.0)
	Ankle	Others (n=85)	57.7 (12.6)	1 (1.2)	7 (8.2)	14 (16.5)	22 (25.9)	26 (30.6)	14 (16.5)	1 (1.2)	31 (36.5)
		Posttraumatic OA (n=308)	62.0 (12.1)	7 (2.3)	10 (3.2)	24 (7.8)	76 (24.7)	98 (31.8)	83 (26.9)	10 (3.2)	134 (43.5)
		Primary OA (n=234)	67.1 (8.9)	0 (0.0)	1 (0.4)	6 (2.6)	37 (15.8)	85 (36.3)	92 (39.3)	13 (5.6)	86 (36.8)
		Rheumatoid arthritis (n=73)	62.1 (13.4)	2 (2.7)	3 (4.1)	8 (11.0)	10 (13.7)	24 (32.9)	26 (35.6)	0 (0.0)	60 (82.2)
	Cruciate ligament	Cruciate ligament injury (n=8,430)	29.8 (10.0)	4,964 (58.9)	1,797 (21.3)	1,299 (15.4)	347 (4.1)	22 (0.3)	1 (0.0)	0 (0.0)	3,772 (44.7)
	BOA	BOA hip (n=3,440)	65.9 (9.2)	1 (0.0)	27 (0.8)	158 (4.6)	552 (16.0)	1,484 (43.1)	1,043 (30.3)	175 (5.1)	2474 (71.9)
		BOA knee (n=10.207)	65.4 (9.1)	5 (0.0)	49 (0.5)	466 (4.6)	1,985 (19.4)	4,365 (42.8)	2,793 (27.4)	544 (5.3)	7370 (72.2)
	Fracture	Ankle and foot (n=3,728)	51.7 (17.4)	530 (14.3)	424 (11.4)	605 (16.3)	819 (22.0)	738 (19.8)	439 (11.8)	163 (4.4)	2,208 (59.4)
		Elbow and forearm (n=13,996)	61.6 (15.9)	794 (5.7)	708 (5.1)	1,189 (8.5)	2,678 (19.1)	4,071 (29.1)	2,999 (21.4)	1,557 (11.1)	10942 (78.2)
		Hip and femur (n=8,378)	77.2 (12.0)	51 (0.6)	58 (0.7)	116 (1.4)	377 (4.5)	1,233 (14.7)	2,442 (29.1)	4,101 (48.9)	5655 (67.5)
		Knee and lower leg (n=10,866)	57.4 (17.0)	960 (8.8)	804 (7.4)	1,377 (12.7)	2,180 (20.1)	2,774 (25.5)	1,962 (18.1)	809 (7.4)	6714 (61.8)
		Lumbar, spine and pelvis (n=1,128)	72.2 (16.8)	36 (3.2)	31 (2.7)	44 (3.9)	111 (9.8)	153 (13.6)	299 (26.5)	454 (40.2)	780 (69.1)
		Shoulder and upper arm (n=7,835)	63.6 (16.6)	464 (5.9)	317 (4.0)	621 (7.9)	1,195 (15.3)	2,064 (26.3)	1,946 (24.8)	1,228 (15.7)	5,291(67.5)
		Unspecified part of trunk, extremities or other body region (n=749)	61.3 (19.2)	76 (10.1)	43 (5.7)	74 (9.9)	106 (14.2)	142 (19.0)	189 (25.2)	119 (15.9)	327 (43.7)
		Wrist and hand (n=4,222)	49.9 (19.2)	882 (20.9)	485 (11.5)	625 (14.8)	750 (17.8)	743 (17.6)	523 (12.4)	214 (5.1)	1,864 (44.1)

For peer review only

Diagnosis-based registers	**Heart failure**	Heart failure (n=1,436)	72.8 (11.1)	2 (0.5)	7 (2.6)	37 (9.5)	137 (20.5)	294 (35.7)	512 (31.1)	447 (0.5)	507 (35.3)
	**Respiratory failure**	COPD (n=900)	73.3 (7.6)	0 (0.0)	0 (0.0)	1 (0.1)	39 (4.3)	237 (26.3)	425 (47.2)	198 (22.0)	555 (61.7)
		Lung fibrosis (n=150)	75.9 (7.7)	0 (0.7)	1 (1.3)	(0.0)	2 (18.7)	28 (46.0)	69 (33.3)	50 (0.7)	68 (45.3)
	**Psoriasis**	Psoriasis vulgaris (n=1,704)	49.4 (15.2)	191 (11.2)	273 (16.0)	370 (21.7)	374 (21.9)	346 (20.3)	123 (7.2)	27 (1.6)	576 (33.8)
		Psoriasis vulgaris + anthropathic (n=537)	51.8 (13.2)	21 (3.9)	82 (15.3)	131 (24.4)	132 (24.6)	127 (23.6)	37 (6.9)	7 (1.3)	237 (44.1)
		Psoriasis unspecified (n=439)	51.6 (14.9)	39 (8.9)	67 (15.3)	80 (18.2)	110 (25.1)	95 (21.6)	41 (9.3)	7 (1.6)	181 (41.2)
	**Rheumatol-ogy**	Rheumatoid arthritis (n=17,870)	59.2 (13.9)	585 (3.3)	1,233 (6.9)	2,362 (13.2)	3,846 (21.5)	5,604 (31.4)	3,430 (19.2)	810 (4.5)	12,954 (72.5)
		Spondyloarthropathy (n=8,782)	47.6 (13.9)	1,009 (11.5)	1,596 (18.2)	2,228 (25.4)	2,058 (23.4)	1,380 (15.7)	448 (5.1)	63 (0.7)	4,271 (48.6)
		Inflammatory systemic disorder (n=1,614)	51.9 (15.8)	155 (9.6)	243 (15.1)	295 (18.3)	337 (20.9)	349 (21.6)	206 (12.8)	29 (1.8)	1,249 (77.4)
		Juvenile rheumatoid diseases (n=1,088)	29.7 (12.8)	676 (62.1)	186 (17.1)	134 (12.3)	43 (4.5)	33 (3.0)	9 (0.8)	1 (0.1)	865 (79.5)
		Vasculitis (n=410)	60.0 (14.0)	22 (5.4)	18 (4.4)	40 (9.8)	66 (16.1)	149 (36.3)	105 (25.6)	10 (2.4)	234 (57.1)
		Other arthritis (n=1,698)	54.0 (15.9)	146 (8.6)	207 (12.2)	294 (17.3)	365 (21.5)	378 (22.3)	250 (14.7)	58 (3.4)	1,095 (64.5)

*Percent based on non-missing values;
Proportions rounded off to one decimal using MS Excel

For peer review only

Table S3: Body Mass Index (BMI) groups of patients across diagnoses in the 11 National Quality Registers (NQRs)

Patient group in NQRs		Diagnosis	BMI						
			Underweight	Normal	Overweight	Obese class I	Obese class II	Obese class III	Missing (count)
Intervention-based registers	Spine	Disc hernia (n=12,582)	79 (0.7)	4,932 (41.6)	4,793 (40.5)	1,627 (13.7)	353 (3.0)	65 (0.5)	733
		Other spinal conditions (n=1,431)	8 (0.6)	506 (38.2)	544 (41.0)	216 (16.3)	42 (3.2)	10 (0.8)	105
		Segmental instability (n=4,290)	22 (0.5)	1,661 (41.4)	1,806 (45.0)	458 (11.4)	61 (1.5)	7 (0.2)	275
		Spinal stenosis (n=28,194)	140 (0.5)	7,506 (29.0)	11,663 (45.1)	5,409 (20.9)	1,015 (3.9)	156 (0.6)	2,305
		Spondylolysis (n=2,463)	15 (0.7)	766 (33.8)	1,017 (44.9)	379 (16.7)	78 (3.4)	9 (0.4)	199
	Hip	Avascular necrosis (n=1,594)	30 (2.3)	486 (37.6)	485 (37.5)	201 (15.5)	70 (5.4)	22 (1.7)	300
		Childhood hip disorder (n=1,846)	15 (1.1)	511 (36.2)	579 (41.1)	215 (15.2)	73 (5.2)	17 (1.2)	436
		Hip osteoarthritis (n=86,023)	426 (0.6)	20,811 (31.6)	28,821 (43.8)	12,079 (18.4)	3,027 (4.6)	592 (0.9)	20,267
		Inflammatory joint disorder (n=1,197)	30 (4.0)	295 (39.5)	279 (37.4)	101 (13.5)	30 (4.0)	11 (1.5)	451
	Knee	Knee osteoarthritis (n=16,020)	22 (0.1)	3,013 (19.3)	7,127 (45.5)	4,158 (26.6)	1,124 (7.2)	207 (1.3)	369
		Knee rheumatoid arthritis (n=304)	4 (1.4)	93 (32.5)	113 (39.5)	59 (20.6)	14 (4.9)	3 (1.0)	18
	Ankle	Others (n=85)	0 (0.0)	9 (15.5)	28 (48.3)	17 (29.3)	2 (3.4)	2 (3.4)	27
		Posttraumatic OA (n=308)	1 (0.5)	40 (20.3)	83 (42.1)	52 (26.4)	16 (8.1)	5 (2.5)	111
		Primary OA (n=234)	1 (0.7)	26 (17.6)	77 (52.0)	31 (20.9)	12 (8.1)	1 (0.7)	86
		Rheumatoid arthritis (n=73)	1 (2.3)	22 (50.0)	12 (27.3)	7 (15.9)	2 (4.5)	0 (0.0)	29
	Cruciate ligament	Cruciate ligament injury (n=8,430)	14 (0.3)	2,547 (58.8)	1,466 (33.8)	252 (5.8)	39 (0.9)	13 (0.3)	4,099
	BOA	BOA hip (n=3,440)	15 (0.4)	1,252 (37.2)	1,458 (43.3)	501 (14.9)	113 (3.4)	27 (0.8)	74
		BOA knee (n=10.207)	25 (0.3)	2,476 (24.8)	4,417 (44.2)	2,171 (21.7)	688 (6.9)	223 (2.2)	207
Diagnosis-based registers	Respiratory failure	COPD (n=900)	114 (15.4)	317 (42.9)	169 (22.9)	89 (12.0)	37 (5.0)	13 (1.8)	161
		Lung fibrosis (n=150)	4 (3.1)	44 (34.1)	51 (39.5)	21 (16.3)	9 (7.0)	0 (0.0)	21
	Psoriasis	Psoriasis vulgaris (n=1,704)	22 (1.3)	511 (30.5)	646 (38.6)	320 (19.1)	123 (7.3)	52 (3.1)	30
		Psoriasis vulgaris + anthropathic (n=537)	8 (1.5)	132 (24.9)	193 (36.3)	129 (24.3)	46 (8.7)	23 (4.3)	6
		Psoriasis unspecified (n=439)	6 (1.4)	117 (28.2)	160 (38.6)	85 (20.5)	33 (8.0)	14 (3.4)	24

BMI categories: Underweight: <18.5; Normal: 18.5–24.9; Overweight: 25.0–29.9; Obese class I: 30.0–34.9; Obese class II: 35.0–39.9; Obese class III: ≥40

*Percent based on non-missing values

Proportions rounded off to one decimal using MS Excel

For peer review only

Table S4: Prevalence of reported problems on EQ-5D-3L by diagnosis, at baseline and 1-year follow-up in the 11 National Quality Registers (NQRs) and in the general population

	Patient group in NQRs	Diagnosis	Severity level	Baseline EQ-5D-3L dimension					1-year follow-up EQ-5D-3L dimension				
				Mobility	Self-care	Usual activities	Pain/ discomfort	Anxiety/ depression	Mobility	Self-care	Usual activities	Pain/ discomfort	Anxiety/ Depression
				n (%)	n (%)	n (%)	n (%)	n (%)	n (%)	n (%)	n (%)	n (%)	n (%)
Intervention-based registers	Spine	Disc hernia (n=12,582)	Level 1	1,996 (15.9)	8,535 (**67.8**)	2,353 (18.7)	89 (0.7)	4,603 (36.6)	9,258 (**73.6**)	11,659 (**92.7**)	9,015 (**71.6**)	3,596 (28.6)	8,144 (**64.7**)
			Level 2	9,931 (**78.9**)	3,788 (30.1)	6,373 (**50.7**)	5,470 (43.5)	6,954 (**55.3**)	3,293 (26.2)	864 (6.9)	3,154 (25.1)	7,799 (**62.0**)	3,903 (31.0)
			Level 3	655 (5.2)	259 (2.1)	3,856 (30.6)	7,023 (**55.8**)	1,025 (8.1)	31 (0.2)	59 (0.5)	413 (3.3)	1,187 (9.4)	535 (4.3)
		Other spinal conditions (n=1,431)	Level 1	279 (19.5)	1,144 (**79.9**)	414 (28.9)	14 (1.0)	586 (41.0)	813 (**56.8**)	1,311 (**91.6**)	905 (**63.2**)	328 (22.9)	893 (**62.4**)
			Level 2	1,113 (**77.8**)	272 (19.0)	689 (**48.1**)	610 (42.6)	742 (**51.9**)	612 (42.8)	111 (7.8)	451 (31.5)	869 (**60.7**)	472 (33.0)
			Level 3	39 (2.7)	15 (1.0)	328 (22.9)	807 **56.4**)	103 (7.2)	6 (0.4)	9 (0.6)	75 (5.2)	234 (16.4)	66 (4.6)
		Segmental instability (n=4,290)	Level 1	1,419 (33.1)	3,277 (**76.4**)	900 (21.0)	21 (0.5)	1,514 (35.3)	3,084 (**71.9**)	3,902 (**91.0**)	2,641 (**61.6**)	977 (22.8)	2,525 (**58.9**)
			Level 2	2,843 (**66.3**)	984 (22.9)	2,463 (**57.4**)	2,160 (**50.3**)	2,462 (**57.4**)	1,192 (27.8)	371 (8.6)	1,364 (31.8)	2,663 (**62.1**)	1,537 (35.8)
			Level 3	28 (0.7)	29 (0.7)	927 (21.6)	2,109 (49.2)	314 (7.3)	14 (0.3)	17 (0.4)	285 (6.6)	650 (15.2)	228 (5.3)
		Spinal stenosis (n=28,194)	Level 1	3,028 (10.7)	24,047 (**85.3**)	9321 (33.1)	162 (0.6)	12,977 (46.0)	13,069 (46.4)	25,673 (**91.1**)	17,978 (**63.8**)	5,697 (20.2)	17,464 (**61.9**)
			Level 2	24,966 (**88.6**)	3,877 (13.8)	15,161 (**53.8**)	14,520 (**51.5**)	13,874 (**49.2**)	15,049 (**53.4**)	2,330 (8.3)	8,926 (31.7)	18,071 (**64.1**)	9,650 (34.2)
			Level 3	200 (0.7)	270 (1.0)	3,712 (13.2)	13,512 (47.9)	1,343 (4.8)	76 (0.3)	191 (0.7)	1,290 (4.6)	4426 (15.7)	1,080 (3.8)
		Spondylolysis (n=2,463)	Level 1	675 (27.4)	2,120 (**86.1**)	703 (28.5)	16 (0.6)	980 (39.8)	1,657 (**67.3**)	2,274 (**92.3**)	1,601 (**65.0**)	581 (23.6)	1,513 (**61.4**)
			Level 2	1,769 (**71.8**)	330 (13.4)	1,373 (**55.7**)	1,316 (**53.4**)	1,319 (**53.6**)	804 (32.6)	181 (7.3)	742 (30.1)	1,552 (**63.0**)	815 (33.1)
			Level 3	19 (0.8)	13 (0.5)	387 (15.7)	1,131 (45.9)	164 (6.7)	2 (0.1)	8 (0.3)	120 (4.9)	330 (13.4)	135 (5.5)
	Hip	Avascular necrosis (n=594)	Level 1	79 (5.0)	985 (**61.8**)	358 (22.5)	30 (1.9)	741 (**46.5**)	857 (**53.8**)	1,383 (**86.8**)	1,085 (**68.1**)	624 (39.1)	1,076 (**67.5**)
			Level 2	1,476 (**92.6**)	570 (35.8)	838 (**52.6**)	592 (37.1)	716 (44.9)	732 (45.9)	198 (12.4)	434 (27.2)	869 (**54.5**)	477 (29.9)
			Level 3	39 (2.4)	39 (2.4)	398 (25.0)	972 (**61.0**)	137 (8.6)	5 (0.3)	13 (0.8)	75 (4.7)	101 (6.3)	41 (2.6)
		Childhood hip disorder (n=1,846)	Level 1	127 (6.9)	1,390 (**75.3**)	653 (35.4)	22 (1.2)	1,014 (**54.9**)	1,278 (**69.2**)	1,672 (**90.6**)	1,449 (**78.5**)	989 (**53.6**)	1,463 (**79.3**)
			Level 2	1,707 (**92.5**)	438 (23.7)	976 (**52.9**)	998 (**54.1**)	758 (41.1)	568 (30.8)	162 (8.8)	348 (18.9)	781 (42.3)	345 (18.7)
			Level 3	12 (0.7)	18 (1.0)	217 (11.8)	826 (44.7)	74 (4.0)	0 (0.0)	12 (0.7)	49 (2.7)	76 (4.1)	38 (2.1)
		Hip osteoarthritis (n=86,023)	Level 1	7,079 (8.2)	67,411 (**78.4**)	35,009 (40.7)	1,335 (1.6)	51,727 (**60.1**)	53,290 (**61.9**)	79,823 (**92.8**)	67,451 (**78.4**)	39,300 (45.7)	67,755 (**78.8**)
			Level 2	78,719 (**91.5**)	17,838 (20.7)	42,897 (**49.9**)	50,371 (**58.6**)	31,621 (36.8)	32,620 (37.9)	5,719 (6.6)	16,947 (19.7)	43,037 (**50.0**)	17,003 (19.8)
			Level 3	225 (0.3)	774 (0.9)	8,117 (9.4)	34,317 (39.9)	2,675 (3.1)	113 (0.1)	481 (0.6)	1,625 (1.9)	3,686 (4.3)	1,265 (1.5)
		Inflammatory joint disorder (n=1,197)	Level 1	55 (4.6)	697 (**58.2**)	344 (28.7)	13 (1.1)	638 (**53.3**)	545 (45.5)	922 (**77.0**)	698 (**58.3**)	357 (29.8)	808 (**67.5**)
			Level 2	1,124 (**93.9**)	461 (38.5)	660 (**55.1**)	558 (46.6)	502 (41.9)	645 (**53.9**)	240 (20.1)	432 (36.1)	735 (**61.4**)	363 (30.3)
			Level 3	18 (1.5)	39 (3.3)	193 (16.1)	626 (**52.3**)	57 (4.8)	7 (0.6)	35 (2.9)	67 (5.6)	105 (8.8)	26 (2.2)
	Knee	Knee osteoarthritis (n=16,020)	Level 1	1,884 (11.8)	15,010 (**93.7**)	8,605 (**53.7**)	290 (1.8)	10,484 (**65.4**)	10,127 (**63.2**)	15,275 (**95.3**)	12,513 (**78.1**)	5,818 (36.3)	12,620 (**78.8**)
			Level 2	14,097 (**88.0**)	869 (5.4)	6,585 (41.1)	10,139 (**63.3**)	5,155 (32.2)	5,867 (36.6)	661 (4.1)	3,246 (20.3)	9,380 (**58.6**)	3,133 (19.6)
			Level 3	39 (0.2)	141 (0.9)	830 (5.2)	5,591 (34.9)	381 (2.4)	26 (0.2)	84 (0.5)	261 (1.6)	822 (5.1)	267 (1.7)
		Knee rheumatoid arthritis (n=304)	Level 1	11 (3.6)	231 (**76.0**)	114 (37.5)	0 (0.0)	185 (**60.9**)	122 (40.1)	247 (**81.3**)	183 (**60.2**)	66 (21.7)	210 (**69.1**)
			Level 2	291 (**95.7**)	69 (22.7)	157 (**51.6**)	189 (**62.2**)	106 (34.9)	181 (**59.5**)	49 (16.1)	108 (35.5)	208 (**68.4**)	89 (29.3)
			Level 3	2 (0.7)	4 (1.3)	33 (10.9)	115 (37.8)	13 (4.3)	1 (0.3)	8 (2.6)	13 (4.3)	30 (9.9)	5 (1.6)

Intervention-based registers

**Ankle**	Other (n=85)	Level 1	6 (7.1)	73 (**85.9**)	31 (36.5)	2 (2.4)	46 (**54.1**)	27 (31.8)	75 (**88.2**)	47 (**55.3**)	22 (25.9)	56 (**65.9**)
		Level 2	77 (**90.6**)	11 (12.9)	39 (**45.9**)	42 (**49.4**)	36 (42.4)	57 (**67.1**)	9 (10.6)	33 (38.8)	56 (**65.9**)	29 (34.1)
		Level 3	2 (2.4)	1 (1.2)	15 (17.6)	41 (48.2)	3 (3.5)	1 (1.2)	1 (1.2)	5 (5.9)	7 (8.2)	0 (0.0)
	Posttraumatic osteoarthritis (n=308)	Level 1	16 (5.2)	285 (**92.5**)	125 (40.6)	4 (1.3)	166 (**53.9**)	109 (35.4)	290 (**94.2**)	200 (**64.9**)	68 (22.1)	217 (**70.5**)
		Level 2	288 (**93.5**)	20 (6.5)	147 (**47.7**)	168 (**54.5**)	123 (39.9)	198 (**64.3**)	15 (4.9)	93 (30.2)	211 (**68.5**)	76 (24.7)
		Level 3	4 (1.3)	3 (1.0)	36 (11.7)	136 (44.2)	19 (6.2)	1 (0.3)	3 (1.0)	15 (4.9)	29 (9.4)	15 (4.9)
	Primary osteoarthritis (n=234)	Level 1	7 (3.0)	214 (**91.5**)	91 (38.9)	2 (0.9)	152 (**65.0**)	97 (41.5)	219 (**93.6**)	165 (**70.5**)	70 (29.9)	182 (**77.8**)
		Level 2	224 (**95.7**)	18 (7.7)	118 (**50.4**)	118 (**50.4**)	78 (33.3)	136 (58.1)	13 (5.6)	65 (27.8)	137 (**58.5**)	51 (21.8)
		Level 3	3 (1.3)	2 (0.9)	25 (10.7)	114 (48.7)	4 (1.7)	1 (0.4)	2 (0.9)	4 (1.7)	27 (11.5)	1 (0.4)
	Rheumatoid arthritis (n=73)	Level 1	70 (0.0)	45 (**61.6**)	17 (23.3)	0 (0.0)	40 (**54.8**)	20 (27.4)	57 (**78.1**)	36 (**49.3**)	10 (13.7)	48 (**65.8**)
		Level 2	3 (**95.9**)	27 (37.0)	42 (**57.5**)	23 (31.5)	29 (39.7)	52 (**71.2**)	14 (19.2)	32 (43.8)	55 (**75.3**)	24 (32.9)
		Level 3	6 (4.1)	1 (1.4)	14 (19.2)	50 (**68.5**)	4 (5.5)	1 (1.4)	2 (2.7)	5 (6.8)	8 (11.0)	1 (1.4)
**Cruciate ligament**	Cruciate ligament injury (n=8,430)	Level 1	5,645 (**67.0**)	8,209 (**97.4**)	4,503 (**53.4**)	1,294 (15.3)	4,190 (**49.7**)	7,308 (**86.7**)	8,337 (**98.9**)	6,663 (**79.0**)	2,943 (34.9)	5,501 (**65.3**)
		Level 2	2,762 (32.8)	178 (2.1)	3,172 (37.6)	6,658 (**79.0**)	3,779 (44.8)	1,117 (13.3)	69 (0.8)	1,636 (19.4)	5,223 (**62.0**)	2,649 (31.4)
		Level 3	23 (0.3)	43 (0.5)	755 (9.0)	478 (5.7)	461 (5.5)	5 (0.1)	24 (0.3)	131 (1.6)	264 (3.1)	280 (3.3)
**BOA**	BOA hip (n=3,440)	Level 1	1,499 (43.6)	3,234 (**94.0**)	2,600 (**75.6**)	61 (1.8)	2,230 (**64.8**)	1,708 (**49.7**)	3,209 (**93.3**)	2,662 (**77.4**)	261 (7.6)	2,305 (**67.0**)
		Level 2	1,938 (**56.3**)	195 (5.7)	799 (23.2)	3,014 (**87.6**)	1,159 (33.7)	1,729 (50.3)	218 (6.3)	741 (21.5)	2,874 (**83.5**)	1,096 (31.9)
		Level 3	3 (0.1)	11 (0.3)	41 (1.2)	365 (10.6)	51 (1.5)	3 (0.1)	13 (0.4)	37 (1.1)	305 (8.9)	39 (1.1)
	BOA knee (n=10,207)	Level 1	4,282 (42.0)	9,886 (**96.9**)	7,674 (**75.2**)	290 (2.8)	6,751 (**66.1**)	5,388 (**52.8**)	9,884 (**96.8**)	8,218 (**80.5**)	1,003 (9.8)	7,248 (**71.0**)
		Level 2	5,916 (**58.0**)	275 (2.7)	2,401 (23.5)	8,741 (**85.6**)	3,314 (32.5)	4,813 (47.2)	276 (2.7)	1,905 (18.7)	8,404 (**82.3**)	2,834 (27.8)
		Level 3	9 (0.1)	46 (0.5)	132 (1.3)	1,176 (11.5)	142 (1.4)	6 (0.1)	47 (0.5)	84 (0.8)	800 (7.8)	125 (1.2)
**Fracture**	Ankle and foot (n=3,718)	Level 1	3,048 (**82.0**)	3,570 (**96.0**)	3,265 (**87.8**)	2,549 (**68.6**)	3,095 (**83.2**)	2,717 (**73.1**)	3,578 (**96.2**)	3,112 (**83.7**)	1,832 (**49.3**)	3,005 (**80.8**)
		Level 2	655 (17.6)	126 (3.4)	322 (8.7)	1,058 (28.5)	556 (15.0)	991 (26.7)	106 (2.9)	537 (14.4)	1,733 (46.6)	645 (17.3)
		Level 3	15 (0.4)	22 (0.6)	131 (3.5)	111 (3.0)	67 (1.8)	10 (0.3)	34 (0.9)	69 (1.9)	153 (4.1)	68 (1.8)
	Elbow and forearm (n=13,996)	Level 1	12,548 (**89.7**)	12,981 (**92.7**)	12,304 (**87.9**)	9,502 (**67.9**)	11,699 (**83.6**)	12,104 (**86.5**)	13,107 (**93.6**)	11,649 (**83.2**)	6,686 (47.8)	11,390 (**81.4**)
		Level 2	1,434 (10.2)	885 (6.3)	1,189 (8.5)	4,161 (29.7)	2,127 (15.2)	1,848 (13.2)	748 (5.3)	2,026 (14.5)	6,924 (**49.5**)	2,406 (17.2)
		Level 3	14 (0.1)	130 (0.9)	503 (3.6)	333 (2.4)	170 (1.2)	44 (0.3)	141 (1.0)	321 (2.3)	386 (2.8)	200 (1.4)
	Hip and femur (n=8,378)	Level 1	4,490 (**53.6**)	6,379 (**76.1**)	5,444 (**65.0**)	3,740 (44.6)	5,486 (**65.5**)	2,419 (28.9)	5,679 (**67.8**)	4,243 (**50.6**)	2,286 (27.3)	4,818 (**57.5**)
		Level 2	3,746 (44.7)	1,526 (18.2)	1,846 (22.0)	4,092 (**48.8**)	2,570 (30.7)	5,584 (**66.7**)	1,879 (22.4)	2,636 (31.5)	5,458 (**65.1**)	3,205 (38.3)
		Level 3	142 (1.7)	473 (5.6)	1,088 (13.0)	546 (6.5)	322 (3.8)	375 (4.5)	820 (9.8)	1,499 (17.9)	634 (7.6)	355 (4.2)
	Knee and lower leg (n=10,866)	Level 1	9,117 (**83.9**)	10,177 (**93.7**)	9,499 (**87.4**)	7,736 (**71.2**)	9,029 (**83.1**)	7,023 (**64.6**)	10,228 (**94.1**)	8,584 (**79.0**)	4,212 (38.8)	8,354 (**76.9**)
		Level 2	1,661 (15.3)	570 (5.2)	871 (8.0)	2,835 (26.1)	1,657 (15.2)	3,762 (34.6)	471 (4.3)	1,939 (17.8)	6,208 (**57.1**)	2,286 (21.0)
		Level 3	88 (0.8)	119 (1.1)	496 (4.6)	295 (2.7)	180 (1.7)	81 (0.7)	167 (1.5)	343 (3.2)	446 (4.1)	226 (2.1)
	Lumbar spine and pelvis (n=1,128)	Level 1	708 (**62.8**)	902 (**80.0**)	775 (**68.7**)	547 (**48.5**)	790 (**70.0**)	548 (48.6)	77.5 (**77.5**)	682 (**60.5**)	412 (36.5)	741 (**65.7**)
		Level 2	400 (35.5)	179 (15.9)	236 (20.9)	478 (42.4)	293 (26.0)	561 (**49.7**)	16.1 (16.1)	296 (26.2)	621 (**55.1**)	348 (30.9)
		Level 3	20 (1.8)	47 (4.2)	117 (10.4)	103 (9.1)	45 (4.0)	19 (1.7)	6.4 (6.4)	150 (13.3)	95 (8.4)	39 (3.5)
	Shoulder and upper arm (n=7,835)	Level 1	6,615 (**84.4**)	6,926 (**88.4**)	6,547 (**83.6**)	4,891 (**62.4**)	6,248 (**79.7**)	6,237 (**79.6**)	6,721 (**85.8**)	5,877 (**75.0**)	2,912 (37.2)	5,876 (**75.0**)
		Level 2	1,189 (15.2)	743 (9.5)	863 (11.0)	2,607 (33.3)	1,447 (18.5)	1,548 (19.8)	940 (12.0)	1,616 (20.6)	4,573 (**58.4**)	1,794 (22.9)
		Level 3	31 (0.4)	166 (2.1)	425 (5.4)	337 (4.3)	140 (1.8)	50 (0.6)	174 (2.2)	342 (4.4)	350 (4.5)	165 (2.1)
	Unspecified part of trunk, extremities or other body region (n=749)	Level 1	569 (**76.0**)	655 (**87.4**)	583 (**77.8**)	448 (**59.8**)	577 (**77.0**)	463 (**61.8**)	641 (**85.6**)	470 (**62.8**)	172 (23.0)	492 (**65.7**)
		Level 2	171 (22.8)	78 (10.4)	99 (13.2)	249 (33.2)	147 (19.6)	273 (36.4)	86 (11.5)	211 (28.2)	494 (**66.0**)	224 (29.9)
		Level 3	9 (1.2)	16 (2.1)	67 (8.9)	52 (6.9)	25 (3.3)	13 (1.7)	22 (2.9)	68 (9.1)	83 (11.1)	33 (4.4)
	Wrist and hand (n=4,222)	Level 1	3,927 (**93.0**)	3,922 (**92.9**)	3,656 (**86.6**)	2,950 (**69.9**)	3,538 (**83.8**)	3,803 (**90.1**)	4,024 (**95.3**)	3,544 (**83.9**)	2,046 (**48.5**)	3,505 (**83.0**)
		Level 2	286 (6.8)	266 (6.3)	389 (9.2)	1,178 (27.9)	622 (14.7)	403 (9.5)	163 (3.9)	603 (14.3)	2,048 (**48.5**)	658 (15.6)
		Level 3	9 (0.2)	34 (0.8)	177 (4.2)	94 (2.2)	62 (1.5)	16 (0.4)	35 (0.8)	75 (1.8)	128 (3.0)	59 (1.4)

Diagnosis-based registers	**Heart failure**	Heart failure (n=1,436)	Level 1	866 (**60.3**)	1,315 (**91.6**)	1,041 (**72.5**)	689 (**48.0**)	865 (**60.2**)	837 (**58.3**)	1,298 (90.**4**)	1,025 (**71.4**)	685 (**47.7**)	896 (**62.4**)
			Level 2	567 (39.5)	116 (8.1)	355 (24.7)	679 (47.3)	521 (36.3)	593 (41.3)	126 (8.8)	371 (25.8)	655 (45.6)	499 (34.7)
			Level 3	3 (0.2)	5 (0.3)	40 (2.8)	68 (4.7)	50 (3.5)	6 (0.4)	12 (0.8)	40 (2.8)	96 (6.7)	41 (2.9)
	**Respir-atory failure**	COPD (n=900)	Level 1	199 (22.1)	551 (**61.2**)	215 (23.9)	250 (27.8)	357 (39.7)	162 (18.0)	479 (**53.2**)	179 (19.9)	212 (23.6)	320 (35.6)
			Level 2	683 (**75.9**)	296 (32.9)	492 (**54.7**)	517 (**57.4**)	463 (**51.4**)	696 (**77.3**)	343 (38.1)	465 (**51.7**)	541 (**60.1**)	489 (**54.3**)
			Level 3	18 (2.0)	53 (5.9)	193 (21.4)	133 (14.8)	80 (8.9)	42 (4.7)	78 (8.7)	256 (28.4)	147 (16.3)	91 (10.1)
		Lung fibrosis (n=150)	Level 1	44 (29.3)	99 (**66.0**)	57 (38.0)	55 (36.7)	75 (**50.0**)	25 (16.7)	72 (**48.0**)	32 (21.3)	44 (29.3)	67 (44.7)
			Level 2	103 (**68.7**)	47 (31.3)	65 (**43.3**)	83 (**55.3**)	69 (46.0)	118 (**78.7**)	66 (44.0)	61 (**40.7**)	86 (**57.3**)	70 (**46.7**)
			Level 3	3 (2.0)	4 (2.7)	28 (18.7)	12 (8.0)	6 (4.0)	7 (4.7)	12 (8.0)	57 (38.0)	20 (13.3)	13 (8.7)
	**Psoria-sis**	Psoriasis vulgaris (n=1,704)	Level 1	1,425 (**83.6**)	1,645 (**96.5**)	1,469 (**86.2**)	666 (39.1)	881 (**51.7**)	1,444 (**84.7**)	1,659 (**97.4**)	1,525 (**89.5**)	884 (**51.9**)	1,113 (**65.3**)
			Level 2	279 (16.4)	54 (3.2)	214 (12.6)	914 (**53.6**)	719 (42.2)	258 (15.1)	41 (2.4)	161 (9.4)	755 (44.3)	529 (31.0)
			Level 3	0 (0.0)	5 (0.3)	21 (1.2)	124 (7.3)	104 (6.1)	2 (0.1)	4 (0.2)	18 (1.1)	65 (3.8)	62 (3.6)
		Psoriasis vulgaris + anthropathic (n=537)	Level 1	311 (**57.9**)	484 (**90.1**)	368 (**68.5**)	91 (16.9)	234 (43.6)	356 (**66.3**)	494 (**92.0**)	422 (**78.6**)	141 (26.3)	314 (**58.5**)
			Level 2	226 (42.1)	50 (9.3)	154 (28.7)	359 (**66.9**)	262 (**48.8**)	180 (33.5)	41 (7.6)	101 (18.8)	342 (**63.7**)	194 (36.1)
			Level 3	0 (0.0)	3 (0.6)	15 (2.8)	87 (16.2)	41 (7.6)	1 (0.2)	2 (0.4)	14 (2.6)	54 (10.1)	29 (5.4)
		Psoriasis unspecified (n=439)	Level 1	328 (**74.7**)	425 (**96.8**)	346 (**78.8**)	128 (29.2)	203 (46.2)	351 (**80.0**)	425 (**96.8**)	382 (**87.0**)	206 (46.9)	298 (**67.9**)
			Level 2	110 (25.1)	11 (2.5)	86 (19.6)	263 (**59.9**)	204 (**46.5**)	88 (20.0)	13 (3.0)	50 (11.4)	210 (**47.8**)	124 (28.2)
			Level 3	1 (0.2)	3 (0.7)	7 (1.6)	48 (10.9)	32 (7.3)	0 (0.0)	1 (0.2)	7 (1.6)	23 (5.2)	17 (3.9)
	**Rheum-atology**	Rheumatoid arthritis (n=17,870)	Level 1	8,758 (49.0)	14,099 (**78.9**)	10,564 (**59.1**)	2,203 (12.3)	10,107 (**56.6**)	9,741 (**54.5**)	14,912 (**83.4**)	11,825 (**66.2**)	3,173 (17.8)	10,970 (**61.4**)
			Level 2	9,065 (**50.7**)	3,539 (19.8)	6,519 (36.5)	13,138 (**73.5**)	7,046 (39.4)	8,060 (45.1)	2,727 (15.3)	5,447 (30.5)	12,734 (**71.3**)	6,196 (34.7)
			Level 3	47 (0.3)	232 (1.3)	787 (4.4)	2,529 (14.2)	717 (4.0)	69 (0.4)	231 (1.3)	598 (3.3)	1,963 (11.0)	704 (3.9)
		Spondyloarthropathy (n=8,782)	Level 1	4,548 (**51.8**)	7,292 (**83.0**)	4,929 (**56.1**)	955 (10.9)	4,316 (**49.1**)	5,051 (**57.5**)	7,470 (**85.1**)	5,502 (**62.7**)	1,263 (14.4)	4,733 (**53.9**)
			Level 2	4,225 (48.1)	1,422 (16.2)	3,392 (38.6)	6,154 (**70.1**)	3,900 (44.4)	3,714 (42.3)	1,245 (14.2)	2,925 (33.3)	6,189 (**70.5**)	3,489 (39.7)
			Level 3	9 (0.1)	68 (0.8)	461 (5.2)	1,673 (19.1)	566 (6.4)	17 (0.2)	67 (0.8)	355 (4.0)	1,330 (15.1)	560 (6.4)
		Inflammatory systemic disorder (n=1,614)	Level 1	920 (**57.0**)	1,355 (**84.0**)	942 (**58.4**)	302 (18.7)	792 (**49.1**)	969 (**60.0**)	1,393 (**86.3**)	1,009 (**62.5**)	341 (21.1)	834 (**51.7**)
			Level 2	686 (42.5)	245 (15.2)	563 (34.9)	1,103 (**68.3**)	730 (45.2)	639 (39.6)	207 (12.8)	521 (32.3)	1,084 (**67.2**)	683 (42.3)
			Level 3	8 (0.5)	14 (0.9)	109 (6.8)	209 (12.9)	92 (5.7)	6 (0.4)	14 (0.9)	84 (5.2)	189 (11.7)	97 (6.0)
		Juvenile rheumatoid diseases (n=1,088)	Level 1	552 (**50.7**)	866 (**79.6**)	611 (**56.2**)	158 (14.5)	545 (**50.1**)	593 (**54.5**)	879 (**80.8**)	660 (**60.7**)	198 (18.2)	571 (**52.5**)
			Level 2	532 (48.9)	205 (18.8)	425 (39.1)	755 (**69.4**)	482 (44.3)	490 (45.0)	193 (17.7)	377 (34.7)	731 (**67.2**)	458 (42.1)
			Level 3	4 (0.4)	17 (1.6)	52 (4.8)	175 (16.1)	61 (5.6)	5 (0.5)	16 (1.5)	51 (4.7)	159 (14.6)	59 (5.4)
		Vasculitis (n=410)	Level 1	249 (**60.7**)	368 (**89.8**)	276 (**67.3**)	91 (22.2)	227 (**55.4**)	239 (**58.3**)	374 (**91.2**)	279 (**68.0**)	96 (23.4)	241 (**58.8**)
			Level 2	161 (39.3)	34 (8.3)	117 (28.5)	285 (**69.5**)	169 (41.2)	170 (41.5)	29 (7.1)	123 (30.0)	275 (**67.1**)	154 (37.6)
			Level 3	0 (0.0)	8 (2.0)	17 (4.1)	34 (8.3)	14 (3.4)	1 (0.2)	7 (1.7)	8 (2.0)	39 (9.5)	15 (3.7)
		Other arthritis (n=1698)	Level 1	856 (**50.4**)	1,414 (**83.3**)	982 (**57.8**)	182 (10.7)	930 (**54.8**)	992 (**58.4**)	1,495 (**88.0**)	1,168 (**68.8**)	302 (17.8)	1,059 (**62.4**)
			Level 2	837 (49.3)	273 (16.1)	629 (37.0)	1,268 (**74.7**)	687 (40.5)	703 (41.4)	190 (11.2)	480 (28.3)	1,193 (**70.3**)	573 (33.7)
			Level 3	5 (0.3)	11 (0.6)	87 (5.1)	248 (14.6)	81 (4.8)	3 (0.2)	13 (0.8)	50 (2.9)	203 (12.0)	66 (3.9)
**General population data** (n=49,169)			Level 1	44,279 (**90.1**)	48,371 (**98.4**)	44,848 (**91.2**)	24,946 (**50.8**)	32,721 (**66.5**)	-	-	-	-	-
			Level 2	4,840 (9.8)	600 (1.2)	3,785 (7.7)	22,185 (45.1)	15,126 (30.8)	-	-	-	-	-
			Level 3	50 (0.1)	198 (0.4)	536 (1.1)	2,038 (4.1)	1,322 (2.7)	-	-	-	-	-

The highest proportion in bold; Level 1: No problems; Level 2: Some/moderate problems; Level 3: confined to bed/unable to/extreme problems; Proportions rounded off to one decimal using MS Excel

For peer review only

For peer review only - http://bmjopen.bmj.com/site/about/guidelines.xhtml

Table S5: Median (IQR) EQ VAS value at baseline, 1-year follow-up and the change in the 11 National Quality Registers (NQRs) and in the general population

Time	Variable	Median EQ VAS value (IQR)									
		Intervention-based registers							Diagnosis-based registers		General population
		Spine	Hip	Knee	Ankle	Cruciate ligament	BOA	Fracture	Heart failure	Respiratory failure	
**Baseline**	**Overall**	50.0 (35.0)	58.0 (35.0)	70.0 (30.0)	57.5 (30.0)	68.0 (32.0)	70.0 (23.0)	90.0 (20.0)	70.0 (30.0)	50.0 (25.0)	85.0 (15.0)
	**Sex**										
	Men	50.0 (40.0)	60.0 (30.0)	71.0 (32.0)	60.0 (35.0)	70.0 (30.0)	75.0 (25.0)	90.0 (20.0)	70.0 (25.0)	50.0 (25.0)	85.0 (15.0)
	Women	45.0 (30.0)	50.0 (31.0)	65.0 (30.0)	50.0 (32.5)	66.0 (34.0)	70.0 (25.0)	90.0 (21.0)	60.0 (25.0)	50.0 (28.0)	84.0 (20.0)
	**Age group**										
	<30	45.0 (35.0)	43.0 (40.8)	-	52.5 (23.8)	69.0 (31.0)	70.0 (1.3)	95.0 (15.0)	-	-	85.0 (15.0)
	30-39	41.0 (30.0)	50.0 (35.0)	60.5 (36.8)	48.0 (26.3)	68.0 (30.0)	65.0 (30.0)	93.0 (15.0)	70.0 (30.0)	-	85.0 (15.0)
	40-49	45.0 (32.0)	55.0 (35.0)	59.5 (40.0)	47.5 (30.0)	65.0 (36.0)	70.0 (30.0)	92.0 (15.0)	67.5 (20.0)	-	85.0 (15.0)
	50-59	50.0 (35.0)	55.0 (34.0)	62.0 (39.0)	55.0 (35.0)	66.0 (34.0)	70.0 (30.0)	92.0 (20.0)	62.0 (25.0)	50.0 (45.0)	84.0 (20.0)
	60-69	50.0 (36.5)	60.0 (35.0)	70.0 (30.0)	55.0 (35.0)	69.0 (40.0)	70.0 (27.0)	91.0 (20.0)	70.0 (20.0)	50.0 (29.0)	83.0 (20.0)
	70-79	50.0 (40.0)	60.0 (35.0)	70.0 (31.0)	65.0 (30.8)	-	70.0 (20.3)	90.0 (24.0)	70.0 (30.0)	50.0 (25.0)	80.0 (20.0)
	80+	50.0 (36.0)	52.0 (30.0)	70.0 (30.0)	50.0 (30.0)	-	70.0 (27.0)	75.0 (35.0)	60.0 (25.0)	50.0 (30.0)	80.0 (40.0)
	**BMI (Kg/m^2)**										
	Under-weight	40.0 (34.8)	50.0 (30.0)	70.0 (25.3)	27.0 (27.5)	76.5 (30.0)	67.5 (20.0)	-	-	50.0 (26.3)	80.0 (20.0)
	Normal	50.0 (35.0)	60.0 (35.0)	70.0 (33.0)	60.0 (31.2)	70.0 (31.0)	75.0 (25.0)	-	-	50.0 (30.0)	85.0 (15.0)
	Over-weight	50.0 (36.0)	60.0 (35.0)	70.0 (31.0)	60.0 (32.0)	70.0 (31.0)	70.0 (20.0)	-	-	50.0 (25.0)	80.0 (20.0)
	Obesity class I	50.0 (35.0)	53.0 (30.0)	65.0 (30.0)	55.0 (30.0)	62.0 (38.0)	69.0 (30.0)	-	-	50.0 (29.0)	80.0 (30.0)
	Obesity class II	40.0 (33.0)	50.0 (37.0)	60.0 (38.0)	50.0 (25.0)	62.0 (37.5)	65.0 (29.0)	-	-	50.0 (25.0)	75.0 (35.0)
	Obesity class III	40.0 (30.0)	50.0 (40.0)	60.0 (32.3)	42.0 (22.5)	64.5 (16.5)	58.0 (30.0)	-	-	60.0 (20.5)	70.0 (35.0)
**1-year follow-up**	**Overall**	70.0 (35.0)	80.0 (23.0)	80.0 (20.0)	75.0 (22.0)	80.0 (24.0)	75.0 (25.0)	80.0 (23.0)	70.0 (20.3)	50.0 (28.0)	-
	**Sex**										
	Men	75.0 (28.0)	81.0 (23.0)	80.0 (20.0)	75.0 (20.0)	80.0 (23.0)	75.0 (25.0)	84.0 (24.0)	70.0 (20.0)	50.0 (25.0)	-
	Women	70.0 (35.0)	80.0 (30.0)	80.0 (25.0)	70.0 (26.5)	78.0 (24.0)	74.0 (25.0)	80.0 (23.0)	70.0 (30.0)	50.0 (25.0)	-
	**Age group**										
	<30	80.0 (25.0)	80.0 (23.8)	-	67.5 (42.3)	80.0 (24.0)	76.5 (26.0)	88.0 (20.0)	-	-	-
	30-39	75.0 (29.0)	80.0 (20.0)	82.5 (10.0)	70.0 (25.5)	80.0 (25.0)	78.0 (15.0)	88.0 (20.0)	90.0 (30.0)	-	-
	40-49	75.0 (30.0)	85.0 (23.0)	75.0 (28.0)	70.0 (27.5)	80.0 (23.0)	70.0 (23.0)	85.0 (20.0)	73.0 (20.0)	-	-
	50-59	75.0 (36.0)	83.0 (24.0)	80.0 (28.0)	75.0 (20.0)	78.0 (24.0)	73.0 (25.0)	85.0 (25.0)	70.0 (24.0)	40.0 (35.0)	-
	60-69	73.0 (34.0)	85.0 (25.0)	80.0 (20.0)	75.0 (20.8)	84.0 (22.0)	74.0 (25.0)	86.0 (21.0)	75.0 (24.3)	50.0 (25.0)	-
	70-79	70.0 (30.0)	80.0 (25.0)	80.0 (20.0)	75.0 (25.0)	-	75.0 (26.0)	80.0 (25.0)	70.0 (25.0)	50.0 (27.3)	-
	80+	60.0 (29.0)	75.0 (34.0)	78.0 (30.0)	60.0 (30.0)	-	71.0 (30.0)	65.0 (30.0)	65.0 (30.0)	50.0 (23.8)	-
	**BMI (Kg/m^2)**										
	Under-weight	70.0 (38.5)	76.0 (30.0)	70.0 (18.8)	45.0 (15.0)	78.5 (25.5)	74.5 (17.8)	-	-	42.5 (26.3)	-
	Normal	75.0 (30.0)	85.0 (25.0)	80.0 (20.0)	75.0 (20.0)	80.0 (23.0)	78.0 (24.0)	-	-	50.0 (25.0)	-
	Over-weight	75.0 (31.0)	80.0 (21.0)	80.0 (20.0)	75.0 (10.0)	80.0 (25.0)	75.0 (25.0)	-	-	50.0 (23.0)	-
	Obesity class I	70.0 (30.0)	80.0 (30.0)	80.0 (25.0)	70.0 (23.5)	75.0 (25.0)	70.0 (29.0)	-	-	50.0 (29.5)	-
	Obesity class II	65.0 (40.0)	75.0 (30.0)	75.0 (30.0)	75.0 (37.5)	75.0 (32.0)	70.0 (28.0)	-	-	50.0 (23.8)	-
	Obesity class III	66.0 (36.3)	70.0 (37.8)	70.0 (34.0)	35.0 (21.3)	68.0 (34.8)	60.0 (35.0)	-	-	50.0 (15.0)	-

For peer review only

**Change**	**Overall**	20.0 (40.0)	20.0 (35.0)	10.0 (26.0)	11.5 (30.0)	10.0 (30.0)	0.0 (19.0)	-5.0 (15.0)	0.0 (20.0)	0.0 (28.0)	-
	**Sex**										
	Men	17.0 (36.0)	18.0 (32.0)	8.0 (22.0)	10.0 (30.0)	10.0 (29.0)	0.0 (20.0)	-5.0 (15.0)	0.0 (16.0)	0.0 (25.0)	-
	Women	20.0 (38.0)	20.0 (35.0)	10.0 (31.0)	15.0 (35.0)	10.0 (30.0)	2.0 (21.0)	-5.0 (15.0)	0.0 (22.0)	0.0 (30.0)	-
	**Age group**										
	<30	25.0 (35.0)	30.0 (40.0)	-	12.5 (45.3)	9.0 (30.0)	4.0 (22.3)	-5.0 (15.0)	-	-	-
	30-39	25.0 (37.0)	24.0 (31.0)	14.5 (35.3)	17.5 (22.3)	10.0 (29.0)	10.0 (21.0)	-5.0 (15.0)	0.0 (0.0)	-	-
	40-49	25.0 (40.0)	24.0 (35.0)	15.0 (35.0)	20.0 (27.5)	13.0 (32.0)	5.0 (20.0)	-5.0 (15.0)	7.5 (14.0)	-	-
	50-59	20.0 (36.0)	20.0 (34.0)	10.0 (31.0)	15.0 (30.0)	10.0 (27.0)	4.0 (20.0)	-5.0 (15.0)	9.0 (20.0)	-10.0 (25.0)	-
	60-69	20.0 (38.0)	20.0 (35.0)	10.0 (29.0)	15.0 (32.0)	7.0 (41.0)	1.0 (18.0)	-5.0 (13.0)	0.0 (15.0)	0.0 (28.3)	-
	70-79	13.0 (30.0)	18.0 (35.0)	9.0 (25.0)	10.0 (25.0)	-	0.0 (20.0)	-5.0 (15.0)	0.0 (20.0)	0.0 (29.5)	-
	80+	10.0 (33.0)	15.0 (31.0)	7.0 (25.0)	10.0 (27.0)	-	0.0 (21.0)	-8.0 (22.0)	0.0 (20.0)	0.0 (28.3)	-
	**BMI (Kg/m^2)**										
	Underweight	20.0 (36.5)	20.0 (30.0)	9.5 (27.5)	18.0 (12.5)	1.5 (22.3)	-0.5 (21.5)	-	-	-5.0 (28.5)	-
	Normal	20.0 (35.0)	20.0 (34.0)	10.0 (26.0)	10.0 (30.0)	9.0 (30.0)	1.0 (15.0)	-	-	0.0 (30.0)	-
	Over-weight	20.0 (39.0)	20.0 (32.0)	10.0 (26.0)	12.5 (30.0)	9.0 (30.0)	0.0 (19.0)	-	-	0.0 (22.8)	-
	Obesity class I	16.0 (35.0)	20.0 (39.0)	10.0 (30.0)	13.0 (30.0)	11.0 (35.0)	0.0 (23.0)	-	-	0.0 (33.0)	-
	Obesity class II	15.0 (35.0)	20.0 (39.0)	10.0 (32.0)	25.0 (42.5)	18.0 (35.5)	3.0 (25.0)	-	-	-2.5 (30.0)	-
	Obesity class III	15.0 (40.0)	20.0 (40.0)	10.0 (38.0)	0.0 (13.8)	-4.0 (27.5)	4.0 (20.0)	-	-	0.0 (20.0)	-

For peer review only

Table S6: Analysis of covariance testing difference in mean EQ VAS value by sex adjusted for age at baseline, 1-year and change in the 11 NQRS and in the general population

Time	Variable	Adjusted mean EQ VAS value (95% CI)									
		Intervention-based registers						Diagnosis-based registers			General population
		Spine	Hip	Knee	Ankle	Cruciate ligament	BOA	Fracture	Heart failure	Respiratory failure	
**Baseline**	**Sex**										
	Men	50.5 (50.2-50.8)	59.7 (59.5-59.9)	68.1 (67.6-68.6)	58.4 (56.2-60.6)	63.9 (63.2-64.6)	70.7 (69.9-71.5)	84.7 (84.4-85.0)	66.5 (65.1-67.9)	50.8 (48.3-53.3)	80.6 (80.3-80.8)
	Women	45.2 (44.9-45.5)	53.6 (53.4-53.8)	62.0 (61.5-62.4)	52.6 (50.2-55.0)	61.4 (60.6-62.1)	67.3 (66.8-67.8)	84.7 (84.5-84.9)	63.0 (60.9-65.0)	51.2 (49.2-53.2)	78.7 (78.4-78.9)
**1-year follow-up**	**Sex**										
	Men	69.3 (69.0-69.6)	78.3 (78.1-78.5)	77.8 (74.7-75.5)	72.3 (70.4-74.2)	75.3 (74.8-75.9)	70.9 (69.7-70.8)	77.2 (76.9-77.5)	68.2 (66.8-69.5)	49.4 (47.0-51.8)	-
	Women	66.5 (66.2-66.8)	75.5 (75.3-75.7)	75.1 (77.4-78.3)	67.7 (65.5-69.8)	73.6 (72.9-74.2)	70.3 (70.1-71.7)	78.0 (77.7-78.2)	65.9 (63.9-67.9)	48.9 (47.0-50.8)	-
**Change**	**Sex**										
	Men	18.7 (21.0-21.7)	18.6 (18.4-18.9)	9.7 (9.1-10.3)	13.9 (12.2-17.9)	11.4 (10.7-12.2)	0.2 (-0.7-1.0)	-7.5 (-7.8--7.2)	1.7 (0.3-3.0)	-1.4 (-4.2-1.4)	-
	Women	21.3 (18.5-19.2)	21.9 (21.6-22.1)	13.1 (12.6-13.6)	15.1 (11.4-16.4)	12.2 (11.3-13.0)	2.9 (2.4-3.5)	-6.8 (-7.0--6.5)	2.9 (1.0-4.9)	-2.4 (-4.6--0.1)	-

Table S7: Mean (SD) EQ VAS value by intervention, at baseline, 1-year follow-up and the change in the 11 National Quality Registers (NQRs)

	Patient group in NQRs	Intervention	Mean EQ VAS value (SD)		
		Category	Baseline	1-year follow-up	Change
Intervention-based registers	Spine	Posterior instrumented fusion	45.4 (20.9)	68.5 (23.0)	+23.0 (25.8)
		Decompression + posterior instrumented fusion	46.0 (22.1)	67.1 (22.9)	+21.1 (26.9)
		Decompression + posterior non-instrumented fusion	46.6 (21.5)	65.1 (21.6)	+18.4 (26.6)
		Midline preserving decompression	49.2 (22.0)	69.0 (20.7)	+19.8 (26.0)
		Decompression (conventional)	48.8 (21.9)	64.8 (23.0)	+16.0 (26.4)
		Decompression (microscopic)	51.5 (21.9)	66.3 (23.0)	+14.8 (26.3)
		Non-midline preserving decompression	50.6 (22.0)	67.1 (21.9)	+16.5 (26.5)
		Decompression + transforaminal lumbar interbody fusion	44.4 (21.1)	66.3 (23.5)	+22.0 (27.9)
		Disc hernia surgery (conventional)	45.2 (22.5)	72.8 (20.8)	+27.6 (27.5)
		Disc hernia surgery (microscopic)	45.3 (22.5)	71.8 (21.3)	+26.5 (27.7)
		Disc prosthesis	49.9 (19.9)	72.3 (21.3)	+22.4 (24.0)
		Others	46.7 (21.9)	67.2 (23.0)	+20.5 (27.0)
		Posterior lumbar interbody fusion	48.6 (21.1)	67.4 (23.5)	+18.9 (25.6)
	Hip	Fully cemented	55.8 (22.0)	75.6 (20.3)	+19.8 (25.9)
		Hybrid	58.7 (23.3)	77.1 (20.9)	+18.4 (26.7)
		Resurfacing	60.0 (20.1)	83.7 (16.0)	+23.8 (22.5)
		Reverse hybrid	55.9 (22.7)	78.3 (19.5)	+22.4 (25.8)
		Uncemented	57.7 (22.5)	79.8 (19.0)	+22.1 (25.5)
	Knee	Total knee arthroplasty	64.6 (22.1)	76.2 (19.4)	+11.7 (24.5)
	Ankle	Arthrodesis	56.2 (21.2)	70.0 (18.8)	+13.8 (23.6)
		Prosthesis	54.9 (21.9)	70.6 (19.1)	+15.7 (26.8)
	Cruciate ligament	Arthroscopic or endoscopic reconstruction of ligament in the knee without foreign material	62.8 (23.0)	74.5 (19.9)	+11.8 (26.4)
	BOA	Arthritis school	68.3 (18.6)	70.4 (18.7)	+2.0 (18.7)
	Fracture	Additional codes	80.4 (20.4)	68.9 (22.1)	-11.4 (21.5)
		Operations on ankle and foot	88.1 (15.8)	78.5 (18.2)	-9.6 (19.5)
		Operations on elbow and forearm	89.3 (14.4)	82.5 (17.3)	-6.8 (17.3)
		Operations on hip joints and femurs	74.0 (22.7)	64.5 (23.3)	-9.5 (23.3)
		Operations on knees and lower legs	87.1 (17.1)	73.6 (19.6)	-13.5 (21.5)
		Minor surgical procedures	85.7 (17.4)	80.4 (19.3)	-5.3 (18.1)
		Operations on pelvis	87.1 (19.1)	70.7 (20.3)	-16.4 (19.3)
		Operations on shoulders and upper arms	86.0 (17.5)	76.6 (18.9)	-9.4 (20.8)
		Operations on vertebrae and neck	88.0 (14.8)	70.1 (21.3)	-17.9 (23.2)
		Operations on wrists and hands	89.4 (13.9)	82.8 (17.2)	-6.7 (17.0)
Diagnosis -based registers	Heart failure	Device therapy	64.6 (18.9)	64.8 (18.1)	+0.2 (17.3)
		No device therapy	65.6 (18.9)	68.0 (18.9)	+2.5 (18.5)
	Respiratory failure	No oxygen assistance	52.0 (21.1)	49.6 (19.6)	-2.3 (23.4)
		Not known/ no information	49.9 (24.2)	41.9 (23.3)	-7.9 (20.9)
		Oxygen assistance	49.2 (21.4)	48.3 (21.8)	-0.9 (25.8)

For peer review only - http://bmjopen.bmj.com/site/about/guidelines.xhtml

Table S8: Median (IQR) EQ VAS value in selected health states across diagnoses, at baseline in the 11 National Quality Registers (NQRs) and in the general population

Health state*	Median (IQR)							
	Spine					Hip		
	Disc hernia	Other spinal conditions	Segmental instability	Spinal stenosis	Spondylolysis	Avascular necrosis	Childhood hip disorder	Hip osteoarthritis
11111	80.0 (20.0)	90.0 (10.0)	90.0 (2.5)	80.0 (20.0)	79.0 (10.0)	65.0 (42.5)	87.5 (25.5)	80.0 (20.0)
11121	70.0 (20.0)	70.0 (20.0)	70.0 (25.0)	75.0 (21.8)	70.0 (20.0)	75.0 (22.0)	73.5 (25.5)	78.0 (21.0)
21121	68.0 (25.0)	65.0 (30.0)	60.5 (22.5)	67.0 (25.0)	61.5 (25.0)	70.0 (25.0)	70.0 (26.5)	70.0 (28.0)
21122	60.0 (25.0)	57.0 (30.0)	50.0 (30.0)	59.0 (25.0)	60.0 (20.0)	57.5 (20.0)	65.0 (20.3)	60.0 (20.0)
21221	60.0 (25.0)	60.0 (25.0)	55.0 (26.5)	60.0 (25.0)	60.0 (30.0)	60.0 (25.0)	63.0 (30.0)	61.0 (25.0)
21222	50.0 (22.0)	50.0 (20.0)	50.0 (25.0)	50.0 (21.0)	50.0 (25.0)	60.0 (20.8)	51.0 (30.0)	50.0 (28.0)
21232	40.0 (21.0)	30.0 (25.0)	35.0 (22.0)	35.0 (25.0)	37.0 (24.5)	40.0 (30.0)	47.5 (30.0)	45.0 (30.0)
22222	50.0 (25.0)	37.5 (17.5)	46.0 (30.0)	40.0 (30.0)	45.0 (15.0)	55.0 (23.5)	50.0 (25.0)	50.0 (28.0)
33333	7.5 (14.3)	9.5 (4.0)	-	5.0 (26.0)	-	20.0 (20.0)	-	15.0 (45.0)

Health state*	Median (IQR)							
	Hip	Knee		Ankle				Cruciate ligament
	Inflammatory joint disorder	Knee osteoarthritis	Knee rheumatoid arthritis	Other	Posttraumatic osteoarthritis	Primary osteoarthritis	Rheumatoid arthritis	
11111	92.0 (13.0)	85.0 (18.3)	-	-	-	-	-	82.0 (20.0)
11121	69.0 (35.0)	84.0 (15.0)	75.5 (16.3)	70.0 (32.5)	70.0 (31.0)	80.0 (17.5)	-	77.0 (22.0)
21121	60.0 (25.0)	77.0 (26.0)	72.0 (28.3)	75.0 (10.0)	70.0 (28.8)	75.0 (21.0)	50.0 (25.0)	67.0 (29.0)
21122	57.0 (20.0)	68.0 (29.0)	60.0 (20.0)	-	50.0 (20.0)	70.0 (0.0)	-	56.0 (31.0)
21221	50.0 (27.5)	70.0 (27.0)	57.0 (20.0)	75.0 (30.0)	60.0 (20.0)	60.0 (25.0)	65.0 (25.0)	65.0 (32.0)
21222	50.0 (25.0)	60.0 (25.0)	60.0 (22.5)	60.0 (25.0)	60.0 (20.0)	50.0 (20.0)	59.0 (16.8)	51.0 (32.0)
21232	40.5 (25.8)	50.0 (39.0)	40.0 (20.0)	50.0 (23.8)	40.0 (22.5)	52.5 (21.3)	27.0 (7.0)	40.0 (38.0)
22222	50.0 (35.0)	50.0 (23.0)	37.5 (19.0)	-	-	-	-	44.5 (23.8)
33333	-	-	-	-	-	-	-	-

Health state	Median (IQR)							
	BOA		Fracture					
	BOA hip	BOA knee	Ankle and foot	Elbow and forearm	Hip and femur	Knee and lower leg	Lumbar, spine and pelvis	Shoulder and upper arm
11111	86.0 (10.0)	90.0 (10.0)	95.0 (10.0)	96.0 (10.0)	95. 0 (12.0)	95.0 (10.0)	95.0 (10.0)	95.0 (10.0)
11121	80.0 (20.0)	80.0 (20.0)	88.0 (10.0)	88.0 (12.0)	80.0 (20.0)	86.0 (11.0)	81.0 (14.5)	88.0 (10.0)
21121	75.0 (20.0)	75.0 (22.0)	80.0 (20.8)	76.0 (19.0)	75.0 (15.0)	80.0 (20.0)	70.0 (20.0)	71.0 (20.0)
21122	65.0 (22.3)	65.0 (25.0)	79.0 (25.0)	69.0 (28.0)	61.0 (25.0)	70.0 (30.0)	60.0 (22.5)	63.0 (24.0)
21221	60.0 (25.0)	65.0 (22.0)	80.0 (30.0)	65.0 (30.0)	69.5 (25.8)	76.0 (31.0)	65.5 (20.0)	68.5 (23.8)
21222	60.0 (26.5)	55.0 (25.0)	70.0 (28.5)	60.0 (20.0)	60.0 (20.0)	65.0 (25.0)	59.0 (16.2)	60.0 (20.2)
21232	50.0 (22.0)	50.0 (25.0)	50.0 (30.0)	40.0 (35.0)	40.0 (20.0)	50.0 (35.0)	55.0 (15.3)	40.0 (27.5)
22222	50.0 (20.0)	50.0 (28.0)	60.0 (30.0)	52.0 (21.8)	50.0 (25.3)	63.0 (31.0)	50.0 (20.0)	50.0 (30.0)
33333	-	-	-	-	20.0 (31.0)	40.0 (13.8)	-	30.0 (41.3)

Health state*	Median (IQR)							
	Fracture		Heart failure	Respiratory failure		General population		
	Unspecified	Wrist and hand		COPD	Lung fibrosis			
11111	95.0 (10.0)	95.0 (10.0)	80.0 (20.0)	70.0 (20.0)	75.0 (10.0)	90.0 (10.0)		
11121	85.0 (15.0)	90.0 (15.0)	71.5 (20.8)	70.0 (15.0)	-	85.0 (10.0)		
21121	70.0 (21.3)	75.0 (25.0)	70.0 (15.0)	60.0 (17.5)	50.0 (15.0)	77.0 (22.0)		
21122	70.0 (30.0)	75.0 (20.0)	55.0 (20.0)	65.0 (34.0)	70.0 (23.8)	60.0 (25.0)		
21221	65.0 (17.5)	75.0 (35.0)	60.0 (20.0)	50.0 (13.8)	50.0 (10.0)	60.0 (25.0)		
21222	65.0 (25.0)	65.0 (32.0)	50.0 (20.0)	50.0 (18.0)	55.0 (12.5)	50.0 (25.0)		
21232	-	52.5 (26.3)	40.0 (20.0)	45.0 (40.0)	-	40.0 (20.0)		
22222	60.0 (15.0)	50.0 (20.5)	55.0 (40.0)	50.0 (16.3)	50.0 (22.5)	45.0 (20.0)		
33333	-	-	-	-	-	15.0 (70.0)		

*Beside health states 11111 and 33333, the health states selected were among the most frequently reported in many of the patient groups; As an example, the health state (health profile) 21232 shows that mobility=level 2 (some problems), self-care=level 1 (no problems), usual activities=level 2 (some problems), pain/discomfort=level 3 (severe problems) and anxiety/depression=level 2 (some problems).

For peer review only - http://bmjopen.bmj.com/site/about/guidelines.xhtml

Table S9: Median (IQR) EQ VAS value in selected health states across diagnoses in the 11 National Quality Registers (NQRs) at 1-year follow-up

Health state*	Median (IQR)							
	Spine					Hip		
	Disc hernia	Other spinal conditions	Segmental instability	Spinal stenosis	Spondylolysis	Avascular necrosis	Childhood hip disorder	Hip osteoarthritis
11111	90.0 (10.0)	90.0 (10.8)	90.0 (12.0)	90.0 (12.0)	90.0 (11.0)	93.0 (15.0)	91.0 (15.0)	92.0 (15.0)
11121	80.0 (15.0)	80.0 (19.0)	80.0 (15.0)	80.0 (16.0)	80.0 (15.0)	82.0 (15.0)	84.0 (15.0)	84.0 (15.0)
21121	75.0 (15.0)	70.0 (22.0)	70.0 (20.0)	70.0 (20.0)	75.0 (12.5)	75.0 (25.0)	77.0 (25.0)	75.0 (20.0)
21122	65.0 (22.0)	65.0 (20.0)	70.0 (26.5)	60.0 (20.0)	64.0 (20.0)	65.0 (20.0)	70.0 (20.0)	65.0 (25.0)
21221	65.0 (20.5)	60.0 (20.0)	62.0 (20.5)	60.0 (20.0)	60.0 (20.0)	65.0 (25.0)	65.0 (26.3)	65.0 (25.0)
21222	51.0 (25.0)	50.0 (21.0)	55.0 (25.0)	50.0 (23.0)	50.0 (25.0)	55.0 (30.0)	60.0 (25.0)	55.0 (21.0)
21232	40.0 (20.0)	40.0 (20.0)	35.5 (20.0)	35.0 (25.0)	40.0 (20.0)	35.0 (23.0)	45.0 (30.0)	40.0 (20.0)
22222	50.0 (20.0)	40.0 (24.0)	50.0 (22.5)	49.0 (25.0)	50.0 (35.0)	50.0 (20.0)	59.0 (27.0)	50.0 (20.0)
33333	9.0 (35.0)	-	-	10.0 (29.5)	-	-	-	12.5 (43.8)

Health state*	Median (IQR)							
	Hip	Knee		Ankle				Cruciate ligament
	Inflammatory joint disorder	Knee osteoarthritis	Knee rheumatoid arthritis	Others	Posttraumatic osteoarthritis	Primary osteoarthritis	Rheumatoid arthritis	
11111	90.0 (15.0)	90.0 (13.0)	90.0 (17.0)	80.0 (10.0)	85.0 (10.0)	85.0 (15.0)	70.0 (15.0)	90.0 (15.0)
11121	80.0 (20.0)	85.0 (15.0)	80.5 (13.0)	80.0 (12.5)	82.5 (15.0)	85.0 (10.0)	80.0 (8.5)	80.0 (17.0)
21121	75.0 (10.0)	75.0 (16.5)	75.0 (10.0)	80.0 (10.0)	75.0 (10.05	75.0 (11.3)	75.0 (11.3)	73.0 (26.0)
21122	67.5 (18.8)	67.0 (25.0)	60.0 (20.0)	65.0 (16.3)	70.0 (7.3)	70.0 (10.0)	-	65.0 (27.5)
21221	62.5 (25.0)	70.0 (25.0)	65.0 (30.0)	71.0 (35.0)	70.0 (20.0)	70.0 (10.0)	60.0 (17.5)	70.0 (18.3)
21222	50.0 (25.0)	58.0 (20.0)	65.0 (26.3)	57.5 (20.0)	60.0 (20.0)	57.5 (28.0)	60.0 (30.0)	57.5 (25.0)
21232	30.0 (18.8)	45.0 (30.0)	50.0 (10.0)	35.0 (2.5)	50.0 (23.3)	50.0 (7.5)	-	40.0 (20.8)
22222	50.0 (20.0)	50.0 (20.0)	60.0 (22.5)	55.0 (18.8)	-	60.0 (3.8)	55.0 (5.0)	43.0 (22.0)
33333	-	30.0 (24.8)	-	-	-	-	-	-

Health state	Median (IQR)							
	BOA		Fracture					
	BOA hip	BOA knee	Lumbar spine and pelvis	Ankle and foot	Elbow and forearm	Hip and femur	Knee and lower leg	Shoulder and upper arm
11111	90.0 (9.0)	90.0 (12.0)	90.0 (17.0)	95.0 (15.0)	95.0 (11.0)	90.0 (19.0)	93.0 (15.0)	95.0 (12.0)
11121	80.0 (16.0)	80.0 (15.0)	80.0 (10.0)	90.0 (15.0)	90.0 (12.0)	85.0 (12.2)	86.0 (10.0)	85.0 (10.0)
21121	70.0 (20.0)	70.0 (20.0)	75.0 (20.0)	80.0 (20.0)	75.0 (15.5)	75.0 (11.0)	80.0 (15.0)	75.0 (15.0)
21122	60.0 (20.0)	60.0 (20.0)	67.0 (25.0)	70.0 (25.0)	65.0 (25.0)	70.0 (23.8)	70.0 (20.0)	65.0 (23.8)
21221	62.5 (20.0)	60.0 (24.3)	60.0 (20.0)	70.0 (20.0)	75.0 (20.0)	63.0 (25.0)	70.0 (20.0)	70.0 (20.0)
21222	55.0 (24.8)	52.0 (25.0)	60.0 (20.0)	60.0 (20.0)	58.5 (20.0)	55.0 (20.0)	60.0 (20.0)	55.0 (15.0)
21232	36.0 (24.0)	40.0 (30.0)	40.0 (35.0)	44.5 (27.5)	50.0 (21.0)	46.5 (28.8)	50.0 (20.0)	49.0 (15.0)
22222	49.0 (19.8)	50.0 (19.5)	50.0 (15.0)	60.0 (26.0)	50.0 (20.8)	50.0 (20.0)	50.0 (20.0)	50.0 (20.0)
33333	-	-	-	-	-	10.0 (15.0)	20.0 (10.5)	10.0 (10.5)

Health state	Median (IQR)							
			Heart failure	Respiratory failure		General population		
	Unspecified	Wrist and hand		COPD	Lung fibrosis			
11111	90.0 (11.8)	95.0 (15.0)	80.0 (20.0)	65.0 (22.5)	71.5 (10.5)	90.0 (10.0)		
11121	85.0 (10.0)	90.0 (15.0)	75.0 (10.0)	75.0 (12.5)	80.0 (12.5)	85.0 (10.0)		
21121	71.0 (19.0)	75.0 (24.3)	70.0 (20.0)	67.5 (16.8)	50.0 (25.0)	77.0 (22.0)		
21122	60.0 (25.5)	70.0 (16.3)	60.0 (20.0)	60.0 (30.0)	-	60.0 (25.0)		
21221	70.0 (11.0)	66.0 (20.0)	60.0 (20.0)	50.0 (25.0)	60.0 (15.0)	60.0 (25.0)		
21222	50.0 (20.0)	70.0 (29.8)	50.0 (12.5)	50.0 (20.0)	50.0 (9.8)	50.0 (25.0)		
21232	62.5 (25.5)	50.0 (20.0)	60.0 (26.5)	45.0 (8.0)	-	40.0 (20.0)		
22222	35.0 (20.0)	46.0 (10.0)	40.0 (25.0)	45.0 (15.0)	45.0 (13.8)	45.0 (20.0)		
33333	-	-	-	5.0 (5.0)	-	15.0 (70.0)		

*The same health states as those selected for the baseline are presented here; As an example, the health state (health profile) 21232 shows that mobility=level 2 (some problems), self-care=level 1 (no problems), usual activities=level 2 (some problems), pain/discomfort=level 3 (severe problems) and anxiety/depression=level 2 (some problems).

For peer review only - http://bmjopen.bmj.com/site/about/guidelines.xhtml

Table S10: Comparison of respondents with only baseline EQ-5D records with respondents with both baseline and 1-year record by sex in each National Quality Register (NQR)

Patient group in NQRs		Variable	Baseline and 1-year follow-up complete n=266,241	Baseline only n=165,615
Intervention-based registers	Spine	Men	25,328 (48.3)	10,285 (51.0)
		Women	23,632 (51.7)	9881 (49.0)
	Hip	Men	39,397 (43.5)	8,272 (45.0)
		Women	51,263 (56.5)	10,095 (55.0)
	Knee	Men	6,932 (42.5)	2,855 (42.4)
		Women	9,392 (57.5)	3,883 (57.6)
	Ankle	Men	389 (55.6)	257 (59.5)
		Women	311 (44.4)	175 (40.5)
	Cruciate ligament	Men	4,658 (55.3)	6,502 (64.4)
		Women	3,772 (44.7)	3,598 (35.6)
	BOA	Men	3,003 (27.9)	6,151 (32.0)
		Women	9,844 (72.1)	13,092 (68.0)
	Fracture	Men	17,111 (33.6)	16,168 (37.2)
		Women	33,781 (64.4)	27,253 (62.8)
Diagnosis-based registers	Heart failure	Men	929 (64.7)	12,978 (67.1)
		Women	507 (35.3)	6,357 (32.9)
	Respiratory	Men	427 (40.7)	1,437 (44.2)
		Women	623 (59.3)	1,812 (55.8)
	Psoriasis	Men	1,686 (62.9)	2,133 (61.3)
		Women	994 (37.1)	1,348 (38.7)
	Rheumatology	Men	10,794 (34.3)	7,588 (36.0)
		Women	20,668 (65.7)	13,495 (64.0)

Proportions rounded off to one decimal using MS Excel

For peer review only

For peer review only - http://bmjopen.bmj.com/site/about/guidelines.xhtml

Table S11: Comparison of respondents with only baseline EQ-5D records with respondents with both baseline and 1-year record, by age in each National Quality Register (NQR)

Patient group in NQRs		Age group	Baseline and 1-year follow-up complete n=266,241	Baseline only n=165,615
Intervention-based registers	Spine	<30	1,674 (3.4)	1,259 (6.2)
		30-39	4,652(9.5)	2,726 (13.5)
		40-49	7,695 (15.7)	3,725 (18.5)
		50-59	8,964 (18.3)	3,803 (18.9)
		60-69	11,835 (24.2)	3,870 (19.2)
		70-79	10,999 (22.5)	3,554 (17.6)
		80+	3,141 (6.4)	1,229 (6.1)
		Mean age (SD)	58.8 (15.3)	55.3 (16.4)
	Hip	<30	122 (0.1)	87 (0.5)
		30-39	625 (0.7)	216 (1.2)
		40-49	3,394 (3.7)	1,088 (5.9)
		50-59	11,766 (13.0)	2,954 (16.1)
		60-69	30,285 (33.4)	5,343 (29.1)
		70-79	31,869 (35.2)	6,154 (33.5)
		80+	12,599 (13.9)	2,525 (13.7)
		Mean age (SD)	68.5 (10.3)	67.3 (11.6)
	Knee	<30	1 (0.0)	1 (0.0)
		30-39	10 (0.1)	10 (0.1)
		40-49	278 (1.7)	164 (2.4)
		50-59	2,136 (13.1)	1,045 (15.5)
		60-69	5,814 (35.6)	2,209 (32.8)
		70-79	6,296 (38.6)	2,593 (38.5)
		80+	1,789 (11.0)	716 (10.6)
		Mean age (SD)	68.9 (8.7)	68.3 (9.3)
	Ankle	<30	10 (1.4)	6 (1.4)
		30-39	21 (3.0)	14 (3.2)
		40-49	52 (7.4)	51 (11.8)
		50-59	145 (20.7)	101 (23.4)
		60-69	233 (33.3)	122 (28.2)
		70-79	215 (30.7)	119 (27.5)
		80+	24 (3.4)	19 (4.4)
		Mean age (SD)	63.2 (11.7)	61.7 (12.5)
	Cruciate ligament	<30	4,964 (58.9)	6,077 (60.2)
		30-39	1,797 (21.3)	2,299 (22.8)
		40-49	1,299 (15.4)	1,427 (14.1)
		50-59	347 (4.1)	272 (2.7)
		60-69	22 (0.3)	25 (0.2)
		70-79	1 (0.0)	0 (0.0)
		80+	0 (0.0)	0 (0.0)
		Mean age (SD)	29.8 (9.1)	29.3 (9.3)
	BOA	<30	6 (0.0)	14 (0.1)
		30-39	76 (0.6)	149 (0.8)
		40-49	624 (4.6)	1,215 (6.3)
		50-59	2,537 (18.6)	3,938 (20.5)
		60-69	5,849 (42.9)	7,289 (37.9)
		70-79	3,836 (28.1)	5,360 (27.9)
		80+	719 (5.3)	1,278 (6.6)
		Mean age (SD)	65.5 (10.0)	65.1 (10.0)
	Fracture	<30	3,793 (7.5)	4,357 (10.0)
		30-39	2,870 (5.6)	2,888 (6.7)
		40-49	4,651 (9.1)	4,072 (9.4)
		50-59	8,216 (16.1)	6,420 (14.8)
		60-69	11,918 (23.4)	7,583 (17.5)
		70-79	10,799 (21.2)	7,877 (18.1)
		80+	8,645 (17.0)	10,244 (23.5)
		Mean age (SD)	62.1 (18.1)	62.2 (20.6)

For peer review only - http://bmjopen.bmj.com/site/about/guidelines.xhtml

Diagnosis-based registers	Heart failure	<30	2 (0.1)	69 (0.4)
		30-39	7 (0.5)	167 (0.9)
		40-49	37 (2.6)	668 (3.5)
		50-59	137 (9.5)	1,873 (9.7)
		60-69	294 (20.5)	4,310 (22.3)
		70-79	512 (35.7)	6,760 (35.0)
		80+	447 (31.1)	5,488 (28.4)
		Mean age (SD)	72.8 (11.1)	71.9 (11.7)
	Respiratory failure	<30	0 (0.1)	0 (0.0)
		30-39	1 (0.1)	2 (0.1)
		40-49	1 (3.9)	15 (0.5)
		50-59	41 (25.2)	96 (3.0)
		60-69	265 (47.0)	668 (20.6)
		70-79	494 (23.6)	1,404 (43.2)
		80+	248 (0.1)	1,064 (32.7)
		Mean age (SD)	73.1 (7.7)	75.7 (8.2)
	Psoriasis	<30	251 (9.4)	375 (10.8)
		30-39	422 (15.7)	538 (15.5)
		40-49	581 (21.7)	681 (19.6)
		50-59	616 (23.0)	771 (22.1)
		60-69	568 (21.2)	696 (20.0)
		70-79	201 (7.5)	360 (10.3)
		80+	41 (1.5)	60 (1.7)
		Mean age (SD)	50.2 (14.8)	50.7 (15.6)
	Rheumatology	<30	2,593 (8.2)	1,967 (9.3)
		30-39	3,483 (11.1)	2,533 (12.0)
		40-49	5,353 (17.0)	3,612 (17.1)
		50-59	6,721 (21.4)	4,282 (20.3)
		60-69	7,893 (25.1)	4,889 (23.2)
		70-79	4,448 (14.1)	3,090 (14.7)
		80+	971 (3.1)	710 (3.4)
		Mean age (SD)	54.3 (15.7)	53.7 (16.2)

Proportions rounded off to one decimal using MS Excel

For peer review only

For peer review only - http://bmjopen.bmj.com/site/about/guidelines.xhtml

Table S12: Prevalence of reported problems on EQ-5D-3L by diagnosis and mean EQ VAS, and EQ-5D indices (TTO, VAS) among patients with complete data at baseline regardless of follow-up status and in the general population

	Patient group in NQRs	Diagnosis	Severity level	Mobility	Self-care	Usual activities	Pain/ discomfort	Anxiety/ depression	Mean (SD)		
				n (%)	n (%)	n (%)	n (%)	n (%)	EQ VAS*	EQ-5D index (TTO) **	EQ-5D index (VAS) **
Intervention-based registers	**Spine**	Disc hernia (n=19,214)	Level 1	2,925 (15.2)	12,853 (**66.9**)	3,464 (18.0)	135 (0.7)	6,634 (34.5)			
			Level 2	15,295 (**79.6**)	5,958 (31.0)	9,815 (**51.1**)	8,181 (42.6)	10,811 (**56.3**)			
			Level 3	994 (5.2)	403 (2.1)	5,935 (30.9)	10,898 (**56.7**)	1,769 (9.2)	45.0 (22.5)	0.66 (0.13)	45.2 (15.9)
		Other spinal conditions (n=2,209)	Level 1	435 (19.7)	1,746 (**79.0**)	615 (27.8)	21 (1.0)	876 (39.7)			
			Level 2	1,714 (**77.6**)	435 (19.7)	1,085 (**49.1**)	922 (41.7)	1,150 (**52.1**)			
			Level 3	60 (2.7)	28 (1.3)	509 (23.0)	1,266 (**57.3**)	183 (8.3)	45.8 (22.9)	0.69 (0.13)	47.9 (16.8)
		Segmental instability (n=5,892)	Level 1	1,851 (31.4)	4,473 (**75.9**)	1,184 (20.1)	28 (0.5)	1,965 (33.4)			
			Level 2	4,000 (**67.9**)	1,374 (23.3)	3,402 (**57.7**)	2,852 (48.4)	3,442 (**58.4**)			
			Level 3	41 (0.7)	45 (0.8)	1,306 (22.2)	3,012 (**51.1**)	485 (8.2)	45.8 (20.9)	0.69 (0.12)	48.3 (16.3)
		Spinal stenosis (n=38,449)	Level 1	3,985 (10.4)	32,361 (**84.2**)	12,289 (32.0)	237 (0.6)	17,157 (44.6)			
			Level 2	34,146 (**88.8**)	5,703 (14.8)	20,734 (**53.9**)	19,231 (**50.0**)	19,155 (**49.8**)			
			Level 3	318 (0.8)	385 (1.0)	5,426 (14.1)	18,981 (49.4)	2,137 (5.6)	48.6 (22.2)	0.71 (0.12)	50.0 (15.8)
		Spondylolysis (n=3,362)	Level 1	887 (26.4)	2,859 (**85.0**)	928 (27.6)	21 (0.6)	1,271 (37.8)			
			Level 2	2,449 (**72.8**)	482 (14.3)	1,877 (**55.8**)	1,723 (**51.2**)	1,847 (**54.9**)			
			Level 3	26 (0.8)	21 (0.6)	557 (16.6)	1,618 (48.1)	244 (7.3)	48.1 (21.8)	0.71 (0.13)	50.1 (16.7)
	**Hip**	Avascular necrosis (n=2,070)	Level 1	106 (5.1)	1,252 (**60.5**)	455 (22.0)	40 (1.9)	930 (44.9)			
			Level 2	1,908 (**92.2**)	761 (36.8)	1,065 (**51.4**)	748 (36.1)	958 (**46.3**)			
			Level 3	56 (2.7)	57 (2.8)	550 (26.6)	1,282 (**61.9**)	182 (8.8)	49.0 (24.0)	0.66 (0.13)	45.4 (15.8)
		Childhood hip disorder (n=2,294)	Level 1	165 (7.2)	1,698 (**74.0**)	776 (33.8)	26 (1.1)	1,226 (**53.4**)			
			Level 2	2,110 (**92.0**)	567 (24.7)	1,227 (**53.5**)	1,210 (**52.7**)	963 (42.0)			
			Level 3	19 (0.8)	29 (1.3)	291 (12.7)	1,058 (46.1)	105 (4.6)	55.2 (22.8)	0.71 (0.12)	51.4 (15.8)
		Hip osteoarthritis (n=103,286)	Level 1	8,320 (8.1)	80,304 (**77.7**)	41,136 (39.8)	1,562 (1.5)	61,196 (**59.2**)			
			Level 2	94,660 (**91.6**)	21,997 (21.3)	51,946 (**50.3**)	59,506 (**57.6**)	38,663 (37.4)			
			Level 3	306 (0.3)	985 (1.0)	10,204 (9.9)	42,218 (40.9)	3,427 (3.3)	56.3 (22.2)	0.73 (0.11)	54.0 (15.4)
		Inflammatory joint disorder (n=1,377)	Level 1	64 (4.6)	795 (**57.7**)	388 (28.2)	15 (1.1)	723 (**52.5**)			
			Level 2	1,292 (**93.8**)	538 (39.1)	762 (**55.3**)	634 (46.0)	586 (42.6)			
			Level 3	21 (1.5)	44 (3.2)	227 (16.5)	728 (**52.9**)	68 (4.9)	47.6 (22.6)	0.69 (0.12)	49.0 (15.2)
	**Knee**	Knee osteoarthritis (n=22,612)	Level 1	2,511 (11.1)	20,989 (**92.8**)	11,596 (**51.3**)	404 (1.8)	14,393 (**63.7**)			
			Level 2	20,047 (**88.7**)	1,408 (6.2)	9,705 (42.9)	14,073 (**62.2**)	7,604 (33.6)			
			Level 3	54 (0.2)	215 (1.0)	1,311 (5.8)	8,135 (36.0)	615 (2.7)	63.9 (22.4)	0.76 (0.11)	57.3 (15.3)
		Knee rheumatoid arthritis (n=450)	Level 1	14 (3.1)	330 (**73.3**)	160 (35.6)	2 (0.4)	261 (**58.0**)			
			Level 2	433 (**96.2**)	116 (25.8)	241 (**53.6**)	256 (**56.9**)	171 (38.0)			
			Level 3	3 (0.7)	4 (0.9)	49 (10.9)	192 (42.7)	18 (4.0)	53.8 (22.2)	0.72 (0.11)	52.3 (15.3)

For peer review only - http://bmjopen.bmj.com/site/about/guidelines.xhtml

Intervention-based registers	Ankle	Other (n=147)	Level 1	7 (4.8)	120 (**81.6**)	43 (29.3)	8 (2.4)	73 (**49.7**)			
			Level 2	133 (**90.5**)	23 (15.6)	71 (**48.3**)	72 (**49.4**)	64 (43.5)			
			Level 3	7 (4.8)	4 (2.7)	33 (22.4)	67 (48.2)	10 (6.8)	56.3 (22.5)	0.70 (0.12)	49.4 (16.6)
		Posttraumatic osteoarthritis (n=517)	Level 1	26 (5.0)	476 (**92.1**)	194 (37.5)	4 (1.3)	273 (**52.8**)			
			Level 2	487 (**94.2**)	36 (7.0)	260 (**50.3**)	276 (**54.5**)	214 (41.4)			
			Level 3	4 (0.8)	5 (1.0)	63 (12.2)	237 (44.2)	30 (5.8)	56.9 (20.9)	0.72 (0.12)	51.5 (15.5)
		Primary osteoarthritis (n=374)	Level 1	9 (2.4)	339 (**90.6**)	141 (37.7)	4 (0.9)	236 (**63.1**)			
			Level 2	360 (**96.3**)	32 (8.6)	193 (**51.6**)	196 (**50.4**)	127 (34.0)			
			Level 3	5 (1.3)	3 (0.8)	40 (10.7)	174 (48.7)	11 (2.9)	57.5 (22.0)	0.73 (0.11)	52.7 (14.8)
		Rheumatoid arthritis (n=94)	Level 1	0 (0.0)	60 (**63.8**)	22 (23.4)	0 (0.0)	52 (**55.3**)			
			Level 2	91 (**96.8**)	32 (34.0)	57 (**60.6**)	33 (31.5)	37 (39.4)			
			Level 3	3 (3.2)	2 (2.1)	15 (16.0)	61 (**68.5**)	5 (5.3)	47.9 (22.4)	0.68 (0.11)	46.6 (14.2)
	Cruciate ligament	Cruciate ligament injury (n=18,530)	Level 1	12,195 (**65.8**)	17,997 (**97.1**)	9,878 (**53.3**)	2,851 (15.4)	9,046 (**48.8**)			
			Level 2	6,285 (33.9)	423 (2.3)	6,846 (36.9)	14,599 (**78.8**)	8,359 (45.1)			
			Level 3	50 (0.3)	110 (0.6)	1,806 (9.7)	1,080 (5.8)	1,125 (6.1)	62.4 (22.8)	0.82 (0.12)	65.6 (16.4)
	BOA	BOA hip (n=8,044)	Level 1	3,271 (40.7)	7,509 (**93.3**)	5,886 (**73.2**)	183 (2.3)	5,040 (**62.7**)			
			Level 2	4,769 (**59.3**)	503 (6.3)	2,036 (25.3)	6,778 (**84.3**)	2,864 (35.6)			
			Level 3	4 (0.0)	32 (0.4)	122 (1.5)	1,083 (13.5)	140 (1.7)	65.8 (19.1)	0.83 (0.10)	66.7 (14.4)
		BOA knee (n=24,846)	Level 1	9,557 (38.5)	23,888 (**96.1**)	17,812 (**71.7**)	713 (2.9)	15,824 (**63.7**)			
			Level 2	15,270 (**61.5**)	837 (3.4)	6,582 (26.5)	20,510 (**82.5**)	8,505 (34.2)			
			Level 3	19 (0.1)	121 (0.5)	452 (1.8)	3,623 (14.6)	517 (2.1)	66.6 (19.4)	0.83 (0.11)	66.2 (14.9)
	Fracture	Ankle and foot (n=6,918)	Level 1	5,522 (**79.8**)	6,567 (**94.9**)	5,916 (**85.5**)	4,639 (**67.1**)	5,590 (**80.8**)			
			Level 2	1,352 (19.5)	290 (4.2)	711 (10.3)	2,034 (29.4)	1,196 (17.3)			
			Level 3	44 (0.6)	61 (0.9)	291 (4.2)	245 (3.5)	132 (1.9)	86.2 (17.3)	0.91 (0.11)	79.4 (15.1)
		Elbow and forearm (n=25,061)	Level 1	22,079 (**88.1**)	22,724 (**90.7**)	21,352 (**85.2**)	16,414 (**65.5**)	20,355 (**81.2**)			
			Level 2	2,938 (11.7)	1972 (7.9)	2,512 (10.0)	7,885 (31.5)	4,321 (17.2)			
			Level 3	44 (0.2)	365 (1.5)	1,197 (4.8)	762 (3.0)	385 (1.5)	86.2 (17.2)	0.91 (0.10)	80.1 (14.1)
		Hip and femur (n=1,609)	Level 1	7,769 (**46.0**)	11,190 (**66.2**)	9,314 (**55.1**)	6,756 (40.0)	9,865 (**58.4**)			
			Level 2	8,535 (50.5)	3,982 (23.6)	4,078 (24.1)	8,738 (**51.7**)	6,023 (35.6)			
			Level 3	600 (3.5)	1,732 (10.2)	3,512 (20.8)	1,410 (8.3)	1,016 (6.0)	68.9 (24.5)	0.80 (0.16)	64.9 (21.0)
		Knee and lower leg (n=18,872)	Level 1	15,311 (**81.1**)	17,313 (**91.7**)	15,987 (**84.7**)	13,022 (**69.0**)	15,195 (**80.5**)			
			Level 2	3,283 (17.4)	1,182 (6.3)	1,736 (9.2)	5,229 (27.7)	3,283 (17.4)			
			Level 3	278 (1.5)	377 (2.0)	1,149 (6.1)	621 (3.3)	394 (2.1)	85.7 (17.9)	0.91 (0.12)	79.3 (15.9)
		Lumbar, spine and pelvis (n=2,405)	Level 1	1,298 (**54.0**)	1,735 (**72.1**)	1,444 (**60.0**)	1,049 (43.6)	1,507 (**62.7**)			
			Level 2	1,058 (44.0)	501 (20.8)	550 (22.9)	1,115 (**46.4**)	765 (31.8)			
			Level 3	49 (2.0)	169 (7.0)	411 (17.1)	241 (10.0)	133 (5.5)	73.0 (24.3)	0.82 (0.16)	67.5 (21.3)
		Shoulder and upper arm (n=14,240)	Level 1	11,546 (**81.1**)	12,159 (**85.4**)	11,323 (**79.5**)	8,519 (**59.8**)	10,806 (**75.9**)			
			Level 2	2,606 (18.3)	1,624 (11.4)	1841 (12.9)	4,995 (35.1)	3,103 (21.8)			
			Level 3	88 (0.6)	457 (3.2)	1,076 (7.6)	726 (5.1)	331 (2.3)	82.8 (20.0)	0.89 (0.12)	77.1 (16.9)
		Unspecified part of trunk, extremities or other body region (n=1,698)	Level 1	1,202 (**70.8**)	1,431 (**84.3**)	1,253 (**73.8**)	947 (**55.8**)	1,207 (**71.1**)			
			Level 2	474 (27.9)	213 (12.5)	263 (15.5)	610 (35.9)	430 (25.3)			
			Level 3	22 (1.3)	54 (3.2)	182 (10.7)	141 (8.3)	61 (3.6)	80.6 (22.0)	0.87 (0.15)	73.7 (19.9)
		Wrist and hand (n=8,215)	Level 1	7,533 (**91.7**)	7,522 (**91.6**)	6,947 (**84.6**)	5,617 (**68.4**)	6,783 (**82.6**)			
			Level 2	661 (8.0)	596 (7.3)	847 (10.3)	2,386 (29.0)	1,288 (15.7)			
			Level 3	21 (0.3)	97 (1.2)	421 (5.1)	212 (2.6)	144 (1.8)	87.6 (16.1)	0.92 (0.10)	80.7 (13.6)

For peer review only - http://bmjopen.bmj.com/site/about/guidelines.xhtml

Diagnosis-based registers	**Heart failure**	Heart failure (n=20,771)	Level 1	12,331 (**59.4**)	18,741 (**90.2**)	14,424 (**69.4**)	10,754 (**51.8**)	12,213 (**58.8**)			
			Level 2	8,334 (40.1)	1,817 (8.7)	5,548 (26.7)	8,920 (42.9)	7,748 (37.3)			
			Level 3	106 (0.5)	213 (1.0)	799 (3.8)	1,097 (5.3)	810 (3.9)	64.6 (19.2)	0.85 (0.12)	71.5 (16.5)
	**Respiratory failure**	COPD (n=3,407)	Level 1	688 (20.2)	1,819 (**53.4**)	830 (24.4)	980 (28.8)	1,267 (37.2)			
			Level 2	2,583 (**75.8**)	1,334 (39.2)	1,724 (**50.6**)	1,941 (**57.0**)	1,830 (**53.7**)			
			Level 3	136 (4.0)	254 (7.5)	853 (25.0)	486 (14.3)	310 (9.1)	47.8 (21.6)	0.72 (0.13)	53.9 (17.4)
		Lung fibrosis (n=892)	Level 1	206 (23.1)	536 (**60.1**)	257 (28.8)	298 (33.4)	405 (45.4)			
			Level 2	655 (**73.4**)	300 (33.6)	413 (**46.3**)	484 (**54.3**)	427 (**47.9**)			
			Level 3	31 (3.5)	56 (6.3)	222 (24.9)	110 (12.3)	60 (6.7)	48.1 (22.0)	0.74 (0.14)	56.6 (17.7)
	**Psoriasis**	Psoriasis vulgaris (n=3,943)	Level 1	3,242 (**82.2**)	3,774 (**95.7**)	3,359 (**85.2**)	1,492 (37.8)	2,000 (**50.7**)			
			Level 2	698 (17.7)	148 (3.8)	522 (13.2)	2,140 (**54.3**)	1,682 (42.7)			
			Level 3	3 (0.1)	21 (0.5)	62 (1.6)	311 (7.9)	261(6.6)	**-**	0.87 (0.11)	73.3 (15.7)
		Psoriasis vulgaris + anthropathic (n=1,128)	Level 1	667 (**59.1**)	1,018 (**90.2**)	773 (**68.5**)	173 (15.3)	526 (**46.6**)			
			Level 2	459 (40.7)	101 (9.0)	321 (28.5)	780 (**69.1**)	515 (45.7)			
			Level 3	2 (0.2)	9 (0.8)	34 (3.0)	175 (15.5)	87 (7.7)	-	0.82 (0.14)	65.7 (18.2)
		Psoriasis unspecified (n=1,090)	Level 1	810 (**74.3**)	1,017 (**93.3**)	842 (**77.2**)	345 (31.7)	539 (**49.4**)			
			Level 2	278 (25.5)	64 (5.9)	216 (19.8)	627 (**57.5**)	469 (43.0)			
			Level 3	2 (0.2)	9 (0.8)	32 (2.9)	118 (10.8)	82 (7.5)	-	0.85 (0.13)	70.3 (17.8)
	**Rheumatology**	Rheumatoid arthritis (n=29,015)	Level 1	14,345 (**49.4**)	22,963 (**79.1**)	17,249 (**59.4**)	3,709 (12.8)	16,449 (**56.7**)			
			Level 2	14,588 (50.3)	5,674 (19.6)	10,483 (36.1)	21,165 (**72.9**)	11,327 (39.0)			
			Level 3	82 (0.3)	378 (1.3)	1,283 (4.4)	4,141 (14.3)	1,239 (4.3)	**-**	0.81 (0.13)	65.1 (17.7)
		Spondyloarthropathy (n=15,175)	Level 1	7,892 (**52.0**)	12,697 (**83.7**)	8,672 (**57.1**)	1,622 (10.7)	7,504 (**49.4**)			
			Level 2	7,257 (47.8)	2,382 (15.7)	5,735 (37.8)	10,680 (**70.4**)	6,645 (43.8)			
			Level 3	26 (0.2)	96 (0.6)	768 (5.1)	2,873 (18.9)	1,026 (6.8)	**-**	0.80 (0.14)	63.1 (18.9)
		Inflammatory systemic disorder (n=2,733)	Level 1	1,597 (**58.4**)	2,308 (**84.4**)	1,635 (**59.8**)	524 (19.2)	1,361 (**49.8**)			
			Level 2	1,121 (41.0)	392 (14.3)	924 (33.8)	1,850 (**67.7**)	1,218 (44.6)			
			Level 3	15 (0.5)	33 (1.2)	174 (6.4)	359 (13.1)	154 (5.6)	**-**	0.81 (0.14)	65.6 (18.3)
		Juvenile rheumatoid diseases (n=1,872)	Level 1	982 (**52.5**)	1,513 (**80.8**)	1,067 (**57.0**)	287 (15.3)	961 (**51.3**)			
			Level 2	880 (47.0)	324 (17.3)	715 (38.2)	1,281 (**68.4**)	814 (43.5)			
			Level 3	10 (0.5)	35 (1.9)	90 (4.8)	304 (16.2)	97 (5.2)	**-**	0.80 (0.14)	64.3 (18.6)
		Vasculitis (n=754)	Level 1	456 (**60.5**)	675 (**89.5**)	503 (**66.7**)	179 (23.7)	402 (**53.3**)			
			Level 2	297 (39.4)	68 (9.0)	219 (29.0)	508 (**67.4**)	326 (43.2)			
			Level 3	1 (0.1)	11 (1.5)	32 (4.2)	67 (8.9)	26 (3.4)	**-**	0.84 (0.12)	68.4 (19.7)
		Other arthritis (n=2,996)	Level 1	1,498 (**50.0**)	2,497 (**83.3**)	1,752 (**58.5**)	322 (10.7)	1,637 (**54.6**)			
			Level 2	1,487 (49.6)	481 (16.1)	1,111 (37.1)	2,245 (**74.9**)	1,198 (40.0)			
			Level 3	11 (0.4)	18 (0.6)	133 (4.4)	429 (14.3)	161 (5.4)	**-**	0.81 (0.13)	64.5 (17.5)
**General population data** (n=49,169)			Level 1	44,279 (**90.1**)	48,371 (**98.4**)	44,848 (**91.2**)	24,946 (**50.8**)	32,721 (**66.5**)			
			Level 2	4,840 (9.8)	600 (1.2)	3,785 (7.7)	22,185 (45.1)	15,126 (30.8)			
			Level 3	50 (0.1)	198 (0.4)	536 (1.1)	2,038 (4.1)	1,322 (2.7)	79.5 (18.3)	0.91 (0.09)	78.6 (12.9)

*EQ VAS calculated based on a subset of the data set with compete data on EQ VAS; Proportions rounded off to one decimal using MS Excel

** The value sets used to calculate EQ-5D index are based on the EQ-5D-5L TTO and VAS value sets (Burström K, Sun S, Gerdtham U-G, *et al.* Swedish experience-based value sets for EQ-5D health states. *Qual Life Res* 2014;**23**:431–42.)

For peer review only - http://bmjopen.bmj.com/site/about/guidelines.xhtml

Table S13: Prevalence of reported problems on EQ-5D-3L by diagnosis and mean EQ VAS, and EQ-5D indices (TTO, VAS) among patients with complete data at 1-year follow-up regardless of baseline status and in the general population

	Patient group in NQRs	Diagnosis	Severity level	Mobility	Self-care	Usual activities	Pain/ discomfort	Anxiety/ depression	Mean (SD)		
				n (%)	n (%)	n (%)	n (%)	n (%)	EQ VAS*	EQ-5D index (TTO)**	EQ-5D index (VAS)**
Intervention-based registers	**Spine**	Disc hernia (n=16,196)	Level 1	11,627 (**71.8**)	14,915 (**92.1**)	11,399 (**70.4**)	4,476 (27.6)	10,272 (**63.4**)			
			Level 2	4,514 (27.9)	1189 (7.3)	4223 (26.1)	10,091 (**62.3**)	5,137 (31.7)			
			Level 3	55 (0.3)	92 (0.6)	574 (3.5)	1,629 (10.1)	787 (4.9)	71.5 (21.5)	0.85 (0.13)	71.0 (18.3)
		Other spinal conditions (n=1,828)	Level 1	1,004 (**54.9**)	1,641 (**89.8**)	1,119 (**61.2**)	392 (21.4)	1,087 (**59.5**)			
			Level 2	816 (44.6)	172 (9.4)	599 (32.8)	1,114 (**60.9**)	639 (35.0)			
			Level 3	8 (0.4)	15 (0.8)	110 (6.0)	322 (17.6)	102 (5.6)	66.0 (23.3)	0.82 (0.14)	66.0 (19.7)
		Segmental instability (n=4,670)	Level 1	3,323 (**71.2**)	4,222 (**90.4**)	2,845 (**60.9**)	1,049 (22.5)	2,734 (**58.5**)			
			Level 2	1,331 (28.5)	429 (9.2)	1,505 (32.2)	2,889 (**61.9**)	1,666 (35.7)			
			Level 3	16 (0.3)	19 (0.4)	320 (6.9)	732 (15.7)	270 (5.8)	68.1 (23.3)	0.83 (0.14)	67.8 (19.7)
		Spinal stenosis (n=32,840)	Level 1	14,943 (45.5)	29,720 (**90.5**)	20,679 (**63.0**)	6,529 (19.9)	20,126 (**61.3**)			
			Level 2	17,793 (**54.2**)	2,845 (8.7)	10,533 (32.1)	21,040 (**64.1**)	11,360 (34.6)			
			Level 3	104 (0.3)	275 (0.8)	1,628 (5.0)	5,271 (16.1)	1,354 (4.1)	65.3 (22.8)	0.82 (0.14)	66.0 (18.8)
		Spondylolysis (n=2,763)	Level 1	1,851(**67.0**)	2,558 (**92.6**)	1,783 (**64.5**)	640 (23.2)	1,690 (**61.2**)			
			Level 2	909 (32.9)	197 (7.1)	852 (30.8)	1,740 (**63.0**)	921 (33.3)			
			Level 3	3 (0.1)	8 (0.3)	128 (4.6)	383 (13.9)	152 (5.5)	68.9 (22.5)	0.84 (0.14)	68.6 (19.1)
	**Hip**	Avascular necrosis (n=2,149)	Level 1	1,069 (49.7)	1,811 (**84.3**)	1,394 (**64.9**)	789 (36.7)	1,415 (**65.8**)			
			Level 2	1,071 (**49.8**)	303 (14.1)	621 (28.9)	1,211 (**56.4**)	676 (31.5)			
			Level 3	9 (0.4)	35 (1.6)	134 (6.2)	149 (6.9)	58 (2.7)	71.0 (22.7)	0.84 (0.13)	69.5 (18.0)
		Childhood hip disorder (n=2,236)	Level 1	1,522 (**68.1**)	2,021 (**90.4**)	1,726 (**77.2**)	1,174 (**52.5**)	1,742 (**77.9**)			
			Level 2	712 (31.8)	198 (8.9)	442 (19.8)	965 (43.2)	444 (19.9)			
			Level 3	2 (0.1)	17 (0.8)	68 (3.0)	97 (4.3)	50 (2.2)	78.9 (19.5)	0.88 (0.12)	75.9 (16.5)
		Hip osteoarthritis (n=101,136)	Level 1	61,928 (**61.2**)	93,392 (**92.3**)	78,639 (**77.8**)	45,889 (45.4)	79,190 (**78.3**)			
			Level 2	39,045 (38.6)	7,113 (7.0)	20,384 (20.2)	50,826 (**50.3**)	20,409 (20.2)			
			Level 3	163 (0.2)	631 (0.6)	2,113 (2.1)	4,421 (4.4)	1,537 (1.5)	76.5 (20.1)	0.88 (0.11)	75.1 (15.6)
		Inflammatory joint disorder (n=1,540)	Level 1	666 (43.2)	1,157 (**75.1**)	866 (**56.2**)	440 (28.6)	1,035 (**67.2**)			
			Level 2	865 (**56.2**)	335 (21.8)	583 (37.9)	956 (**62.1**)	468 (30.4)			
			Level 3	9 (0.6)	48 (3.1)	91 (5.9)	144 (9.4)	37 (2.4)	67.8 (22.1)	0.82 (0.13)	67.1 (17.8)
	**Knee**	Knee osteoarthritis (n=18,231)	Level 1	11,460 (**62.9**)	17,338 (**95.1**)	14,143 (**77.6**)	6,610 (36.3)	14,276 (**78.3**)			
			Level 2	6,740 (37.0)	788 (4.3)	3,768 (20.7)	10,668 (**58.5**)	3,639 (20.0)			
			Level 3	31 (0.2)	105 (0.6)	320 (1.8)	953 (5.2)	316 (1.7)	76.1 (19.5)	0.88 (0.11)	74.5 (15.4)
		Knee rheumatoid arthritis (n=372)	Level 1	140 (37.6)	303 (**81.5**)	208 (**55.9**)	79 (21.2)	245 (**65.9**)			
			Level 2	230 (**61.8**)	60 (16.1)	146 (39.2)	254 (**68.3**)	118 (31.7)			
			Level 3	2 (0.5)	9 (2.4)	18 (4.8)	39 (10.5)	9 (2.4)	67.1 (21.0)	0.81 (0.13)	65.8 (17.4)

For peer review only - http://bmjopen.bmj.com/site/about/guidelines.xhtml

Intervention-based registers	**Ankle**	Other (n=152)	Level 1	41 (27.0)	130 (**85.5**)	82 (**53.9**)	37 (24.3)	92 (**60.5**)			
			Level 2	108 (**71.1**)	21 (13.8)	64 (42.1)	98 (**64.5**)	55 (36.2)			
			Level 3	3 (2.0)	1 (0.7)	6 (3.9)	17 (11.2)	5 (3.3)	65.0 (21.6)	0.80 (0.13)	64.1 (17.9)
		Posttraumatic osteoarthritis (n=510)	Level 1	178 (34.9)	472 (**92.5**)	314 (**61.6**)	120 (23.5)	338 (**66.3**)			
			Level 2	329 (**64.5**)	34 (6.7)	165 (32.4)	328 (**64.3**)	140 (27.5)			
			Level 3	3 (0.6)	4 (0.8)	31 (6.1)	62 (12.2)	32 (6.3)	68.6 (21.3)	0.81 (0.14)	65.8 (19.1)
		Primary osteoarthritis (n=388)	Level 1	166 (42.8)	364 (**93.8**)	277 (**71.4**)	121 (31.2)	302 (**77.8**)			
			Level 2	221 (**57.0**)	22 (5.7)	95 (24.5)	222 (**57.2**)	80 (20.6)			
			Level 3	1 (0.3)	2 (0.5)	16 (4.1)	45 (11.6)	6 (1.5)	72.4 (18.6)	0.85 (0.12)	70.3 (17.6)
		Rheumatoid arthritis (n=117)	Level 1	32 (27.4)	94 (**80.3**)	60 (**51.3**)	25 (21.4)	73 (**62.4**)			
			Level 2	83 (**70.9**)	21 (17.9)	51 (43.6)	78 (**66.7**)	40 (34.2)			
			Level 3	2 (1.7)	2 (1.7)	6 (5.1)	14 (12.0)	4 (3.4)	64.3 (19.9)	0.80 (0.13)	63.7 (17.4)
	**Cruciate ligament**	Cruciate ligament injury (n=15,731)	Level 1	13,492 (**85.8**)	15,534 (**98.7**)	12,269 (**78.0**)	5,356 (34.0)	10,105 (**64.2**)			
			Level 2	2,230 (14.2)	142 (0.9)	3,207 (20.4)	9,812 (**62.4**)	5,085 (32.3)			
			Level 3	9 (0.1)	55 (0.3)	255 (1.6)	563 (3.6)	541 (3.4)	74.2 (20.1)	0.88 (0.11)	75.2 (15.0)
	**BOA**	BOA hip (n=3,437)	Level 1	1,726 (**50.2**)	3,212 (**93.5**)	2,659 (**77.4**)	258 (7.5)	2,301 (**66.9**)			
			Level 2	1,708 (49.7)	214 (6.2)	742 (21.6)	2,887 (**84.0**)	1,093 (31.8)			
			Level 3	3 (0.1)	11 (0.3)	36 (1.0)	292 (8.5)	43 (1.3)	68.0 (18.8)	0.85 (0.10)	69.8 (14.4)
		BOA knee (n=10,248)	Level 1	5,418 (**52.9**)	9,926 (**96.9**)	8,258 (**80.6**)	1,015 (9.9)	7,297 (**71.2**)			
			Level 2	4,824 (47.1)	272 (2.7)	1,906 (18.6)	8,437 (**82.3**)	2,830 (27.6)			
			Level 3	6 (0.1)	50 (0.5)	84 (0.8)	796 (7.8)	121 (1.2)	70.7 (18.6)	0.86 (0.10)	71.2 (14.0)
	**Fracture**	Ankle and foot (n=3,898)	Level 1	2,832 (**72.7**)	3,740 (**95.9**)	3,248 (**83.3**)	1,917 (**49.2**)	3,139 (**80.5**)			
			Level 2	1,055 (27.1)	124 (3.2)	569 (14.6)	1,820 (46.7)	684 (17.5)			
			Level 3	11 (0.3)	34 (0.9)	81 (2.1)	161 (4.1)	75 (1.9)	81.0 (19.0)	0.90 (0.11)	77.3 (15.3)
		Elbow and forearm (n=14,576)	Level 1	12,585 (**86.3**)	13,594 (**93.3**)	12,045 (**82.6**)	6,886 (47.2)	11,816 (**81.1**)			
			Level 2	1,944 (13.3)	828 (5.7)	2,155 (14.8)	7,270 (**49.9**)	2,547 (17.5)			
			Level 3	47 (0.3)	154 (1.1)	376 (2.6)	420 (2.9)	213 (1.5)	81.3 (18.5)	0.90 (0.10)	78.7 (13.6)
		Hip and femur (n=8,858)	Level 1	2,521 (28.5)	5,926 (**66.9**)	4,424 (**49.9**)	2,403 (27.1)	5,072 (**57.3**)			
			Level 2	5,914 (**66.8**)	2,027 (22.9)	2,791 (31.5)	5,770 (**65.1**)	3,407 (38.5)			
			Level 3	423 (4.8)	905 (10.2)	1,643 (18.5)	685 (7.7)	379 (4.3)	64.0 (23.5)	0.78 (0.14)	62.2 (18.8)
		Knee and lower leg (n=11,360)	Level 1	7,279 (**64.1**)	10,644 (**93.7**)	8,896 (**78.3**)	4,384 (38.6)	8,690 (**76.5**)			
			Level 2	3,981 (35.0)	527 (4.6)	2,055 (18.1)	6,507 (**57.3**)	2,424 (21.3)			
			Level 3	100 (0.9)	189 (1.7)	409 (3.6)	469 (4.1)	246 (2.2)	78.2 (19.4)	0.88 (0.12)	74.5 (16.1)
		Lumbar, spine and pelvis (n=1,232)	Level 1	583 (47.3)	945 (**76.7**)	731 (**59.3**)	434 (35.2)	794 (**64.4**)			
			Level 2	626 (**50.8**)	208 (16.9)	332 (26.9)	696 (**56.5**)	390 (31.7)			
			Level 3	23 (1.9)	79 (6.4)	169 (13.7)	102 (8.3)	48 (3.9)	68.7 (23.4)	0.82 (0.15)	67.1 (19.7)
		Shoulder and upper arm (n=8,273)	Level 1	6,566 (**79.4**)	7,041 (**85.1**)	6,141 (**74.2**)	3,055 (36.9)	6,162 (**74.5**)			
			Level 2	1,652 (20.0)	1,035 (12.5)	1,727 (20.9)	4,834 (**58.4**)	1,925 (23.3)			
			Level 3	55 (0.7)	197 (2.4)	405 (4.9)	384 (4.6)	186 (2.2)	77.0 (20.3)	0.88 (0.12)	74.9 (15.9)
		Unspecified part of trunk, extremities or other body region (n=860)	Level 1	513 (**59.7**)	719 (**83.6**)	526 (**61.2**)	190 (22.1)	552 (**64.2**)			
			Level 2	329 (38.3)	111 (12.9)	248 (28.8)	565 (**65.7**)	270 (31.4)			
			Level 3	18 (2.1)	30 (3.5)	86 (10.0)	105 (12.2)	38 (4.4)	69.9 (22.0)	0.83 (0.15)	67.6 (19.6)
		Wrist and hand (n=4,532)	Level 1	4,069 (**89.8**)	4,301 (**94.9**)	3,775 (**83.3**)	2,171 (47.9)	3,745 (82.6)			
			Level 2	445 (9.8)	189 (4.2)	667 (14.7)	2,225 (49.1)	722 (15.9)			
			Level 3	18 (0.4)	42 (0.9)	90 (2.0)	136 (3.0)	65 (1.4)	82.9 (17.7)	0.91 (0.09)	79.4 (13.2)

For peer review only - http://bmjopen.bmj.com/site/about/guidelines.xhtml

Diagnosis-based registers	**Heart failure**	Heart failure (n=1,849)	Level 1	1,094 (**59.2**)	1,680 (**90.9**)	1,338 (**72.4**)	911 (**49.3**)	1,157 (**62.6**)			
			Level 2	748 (40.5)	153 (8.3)	458 (24.8)	819 (44.3)	645 (34.9)			
			Level 3	7 (0.4)	16 (0.9)	53 (2.9)	119 (6.4)	47 (2.5)	66.7 (19.5)	0.86 (0.12)	72.3 (16.3)
	**Respiratory failure**	COPD (n=895)	Level 1	160 (17.9)	472 (**52.7**)	175 (19.6)	208 (23.2)	315 (35.2)			
			Level 2	693 (**77.4**)	344 (38.4)	460 (**51.4**)	541 (**60.4**)	488 (**54.5**)			
			Level 3	42 (4.7)	79 (8.8)	260 (29.1)	146 (16.3)	92 (10.3)	48.1 (20.4)	0.70 (0.14)	51.7 (17.5)
		Lung fibrosis (n=149)	Level 1	25 (16.8)	72 (**48.3**)	32 (21.5)	43 (28.9)	68 (**45.6**)			
			Level 2	118 (**79.2**)	66 (44.3)	61 (**40.9**)	86 (**57.7**)	68 (**45.6**)			
			Level 3	6 (4.0)	11 (7.4)	56 (37.6)	20 (13.4)	13 (8.7)	48.5 (19.7)	0.71 (0.14)	53.1 (18.2)
	**Psoriasis**	Psoriasis vulgaris (n=1,880)	Level 1	1,612 (**85.7**)	1,829 (**97.3**)	1,687 (**89.7**)	1,004 (**53.4**)	1,236 (**65.7**)			
			Level 2	267 (14.2)	48 (2.6)	172 (9.1)	806 (42.9)	573 (30.5)			
			Level 3	1 (0.1)	3 (0.2)	21 (1.1)	70 (3.7)	71 (3.8)	-	0.90 (0.10)	78.0 (13.9)
		Psoriasis vulgaris + anthropathic (n=663)	Level 1	406 (**64.3**)	584 (**92.6**)	497 (**78.8**)	161 (25.5)	358 (**56.7**)			
			Level 2	223 (35.3)	41 (6.5)	118 (18.7)	405 (**64.2**)	236 (37.4)			
			Level 3	2 (0.3)	6 (1.0)	16 (2.5)	65 (10.3)	37 (5.9)	-	0.85 (0.12)	70.3 (17.1)
		Psoriasis unspecified (n=363)	Level 1	283 (**78.0**)	351 (**96.7**)	316 (**87.1**)	160 (44.1)	244 (**67.2**)			
			Level 2	80 (22.0)	11 (3.0)	45 (12.4)	183 (**50.4**)	104 (28.7)			
			Level 3	1,612 (0.0)	1 (0.3)	2 (0.6)	20 (5.5)	15 (4.1)	-	0.89 (0.11)	76.1 (14.9)
	**Rheumatology**	Rheumatoid arthritis (n=18,141)	Level 1	9,904 (54.6)	15,143 (83.5)	12,032 (66.3)	3,199 (17.6)	11,123 (61.3)			
			Level 2	8,165 (45.0)	2,763 (15.2)	5,506 (30.4)	12,974 (71.5)	6,294 (34.7)			
			Level 3	72 (0.4)	235 (1.3)	603 (3.3)	1,968 (10.8)	724 (4.0)	-	0.83 (0.13)	67.9 (17.4)
		Spondyloarthropathy (n=8,916)	Level 1	5,142 (**57.7**)	7,578 (**85.0**)	5,582 (**62.6**)	1,293 (14.5)	4,813 (**54.0**)			
			Level 2	3,757 (42.1)	1,270 (14.2)	2,973 (33.3)	6,277 (**70.4**)	3,533 (39.6)			
			Level 3	17 (0.2)	68 (0.8)	361 (4.0)	1,346 (15.1)	570 (6.4)	-	0.82 (0.14)	65.7 (18.8)
		Inflammatory systemic disorder (n1,654)	Level 1	984 (**59.5**)	1,425 (**86.2**)	1,029 (**62.2**)	350 (21.2)	849 (**51.3**)			
			Level 2	664 (40.1)	214 (12.9)	540 (32.6)	1,109 (**67.0**)	705 (42.6)			
			Level 3	6 (0.4)	15 (0.9)	85 (5.1)	195 (11.8)	100 (6.0)	-	0.82 (0.13)	66.4 (18.2)
		Juvenile rheumatoid diseases (n=1,112)	Level 1	605 (**54.4**)	894 (**80.4**)	670 (**60.3**)	204 (18.3)	580 (**52.2**)			
			Level 2	502 (45.1)	202 (18.2)	387 (34.8)	747 (**67.2**)	470 (42.3)			
			Level 3	5 (0.4)	16 (1.4)	55 (4.9)	161 (14.5)	62 (5.6)	-	0.81 (0.14)	65.3 (18.9)
		Vasculitis (n=413)	Level 1	242 (**58.6**)	375 (**90.8**)	278 (**67.3**)	92 (22.3)	242 (**58.6**)			
			Level 2	170 (41.2)	31 (7.5)	125 (30.3)	281 (**68.0**)	156 (37.8)			
			Level 3	1 (0.2)	7 (1.7)	10 (2.4)	40 (9.7)	15 (3.6)	-	0.84 (0.12)	68.7 (16.6)
		Other arthritis (n=1,728)	Level 1	1,004 (**58.1**)	1,521 (**88.0**)	1,185 (**68.6**)	303 (17.5)	1,066 (**61.7**)			
			Level 2	721 (41.7)	193 (11.2)	493 (28.5)	1,212 (**70.1**)	595 (34.4)			
			Level 3	3 (0.2)	14 (0.8)	50 (2.9)	213 (12.3)	67 (3.9)	-	0.84 (0.13)	68.4 (17.4)
**General population data** (n=49,169)			Level 1	44,279 (**90.1**)	48,371 (**98.4**)	44,848 (**91.2**)	24,946 (**50.8**)	32,721 (**66.5**)			
			Level 2	4,840 (9.8)	600 (1.2)	3,785 (7.7)	22,185 (45.1)	15,126 (30.8)			
			Level 3	50 (0.1)	198 (0.4)	536 (1.1)	2,038 (4.1)	1,322 (2.7)	79.5 (18.3)	0.91 (0.09)	78.6 (12.9)

*EQ VAS calculated based on a subset of the data set with compete data on EQ VAS; Proportions rounded off to one decimal using MS Excel

** The value sets used to calculate EQ-5D index are based on the EQ-5D-5L TTO and VAS value sets (Burström K, Sun S, Gerdtham U-G, *et al.* Swedish experience-based value sets for EQ-5D health states. *Qual Life Res* 2014;**23**:431–42.)

For peer review only - http://bmjopen.bmj.com/site/about/guidelines.xhtml

The RECORD statement – checklist of items, extended from the STROBE statement, that should be reported in observational studies using routinely collected health data.

	Item No.	STROBE items	Location in manuscript where items are reported	RECORD items	Location in manuscript where items are reported
**Title and abstract**					
	1	(a) Indicate the study's design with a commonly used term in the title or the abstract (b) Provide in the abstract an informative and balanced summary of what was done and what was found	**1-3**	RECORD 1.1: The type of data used should be specified in the title or abstract. When possible, the name of the databases used should be included.<br><br>RECORD 1.2: If applicable, the geographic region and timeframe within which the study took place should be reported in the title or abstract.<br><br>RECORD 1.3: If linkage between databases was conducted for the study, this should be clearly stated in the title or abstract.	**1-3**<br><br>**1**
**Introduction**					
Background rationale	2	Explain the scientific background and rationale for the investigation being reported	**5**		
Objectives	3	State specific objectives, including any prespecified hypotheses	**6-7**		
**Methods**					
Study Design	4	Present key elements of study design early in the paper	8		
Setting	5	Describe the setting, locations, and relevant dates, including periods of recruitment, exposure, follow-up, and data collection	8		

For peer review only

Participants	6	*(a) Cohort study* - Give the eligibility criteria, and the sources and methods of selection of participants. Describe methods of follow-up<br>*Case-control study* - Give the eligibility criteria, and the sources and methods of case ascertainment and control selection. Give the rationale for the choice of cases and controls<br>*Cross-sectional study* - Give the eligibility criteria, and the sources and methods of selection of participants<br><br>*(b) Cohort study* - For matched studies, give matching criteria and number of exposed and unexposed<br>*Case-control study* - For matched studies, give matching criteria and the number of controls per case	8	RECORD 6.1: The methods of study population selection (such as codes or algorithms used to identify subjects) should be listed in detail. If this is not possible, an explanation should be provided.<br><br>RECORD 6.2: Any validation studies of the codes or algorithms used to select the population should be referenced. If validation was conducted for this study and not published elsewhere, detailed methods and results should be provided.<br><br>RECORD 6.3: If the study involved linkage of databases, consider use of a flow diagram or other graphical display to demonstrate the data linkage process, including the number of individuals with linked data at each stage.	8
Variables	7	Clearly define all outcomes, exposures, predictors, potential confounders, and effect modifiers. Give diagnostic criteria, if applicable.	8	RECORD 7.1: A complete list of codes and algorithms used to classify exposures, outcomes, confounders, and effect modifiers should be provided. If these cannot be reported, an explanation should be provided.	
Data sources/ measurement	8	For each variable of interest, give sources of data and details of methods of assessment (measurement).<br>Describe comparability of assessment methods if there is more than one group	8		

For peer review only

Bias	9	Describe any efforts to address potential sources of bias	10		
Study size	10	Explain how the study size was arrived at	8		
Quantitative variables	11	Explain how quantitative variables were handled in the analyses. If applicable, describe which groupings were chosen, and why	9		
Statistical methods	12	(a) Describe all statistical methods, including those used to control for confounding	9		
		(b) Describe any methods used to examine subgroups and interactions	9		
		(c) Explain how missing data were addressed	10		
		(d) *Cohort study* - If applicable, explain how loss to follow-up was addressed<br>*Case-control study* - If applicable, explain how matching of cases and controls was addressed<br>*Cross-sectional study* - If applicable, describe analytical methods taking account of sampling strategy			
		(e) Describe any sensitivity analyses	10		
Data access and cleaning methods		..		RECORD 12.1: Authors should describe the extent to which the investigators had access to the database population used to create the study population.	

For peer review only

				RECORD 12.2: Authors should provide information on the data cleaning methods used in the study.	8
Linkage		..		RECORD 12.3: State whether the study included person-level, institutional-level, or other data linkage across two or more databases. The methods of linkage and methods of linkage quality evaluation should be provided.	
**Results**					
Participants	13	(a) Report the numbers of individuals at each stage of the study (*e.g.*, numbers potentially eligible, examined for eligibility, confirmed eligible, included in the study, completing follow-up, and analysed)<br>(b) Give reasons for non-participation at each stage.<br>(c) Consider use of a flow diagram	8	RECORD 13.1: Describe in detail the selection of the persons included in the study (*i.e.*, study population selection) including filtering based on data quality, data availability and linkage. The selection of included persons can be described in the text and/or by means of the study flow diagram.	8
Descriptive data	14	(a) Give characteristics of study participants (*e.g.*, demographic, clinical, social) and information on exposures and potential confounders<br>(b) Indicate the number of participants with missing data for each variable of interest<br>(c) *Cohort study* - summarise follow-up time (*e.g.*, average and total amount)	11		
Outcome data	15	*Cohort study* - Report numbers of outcome events or summary measures over time<br>*Case-control study* - Report numbers in each exposure	11		

For peer review only

		category, or summary measures of exposure<br>*Cross-sectional study* - Report numbers of outcome events or summary measures			
Main results	16	(a) Give unadjusted estimates and, if applicable, confounder-adjusted estimates and their precision (e.g., 95% confidence interval). Make clear which confounders were adjusted for and why they were included<br>(b) Report category boundaries when continuous variables were categorized<br>(c) If relevant, consider translating estimates of relative risk into absolute risk for a meaningful time period	11-14		
Other analyses	17	Report other analyses done—e.g., analyses of subgroups and interactions, and sensitivity analyses	Supplementary material		
**Discussion**					
Key results	18	Summarise key results with reference to study objectives	15		
Limitations	19	Discuss limitations of the study, taking into account sources of potential bias or imprecision. Discuss both direction and magnitude of any potential bias	18	RECORD 19.1: Discuss the implications of using data that were not created or collected to answer the specific research question(s). Include discussion of misclassification bias, unmeasured confounding, missing data, and changing eligibility over time, as they pertain to the study being reported.	18
Interpretation	20	Give a cautious overall interpretation of results considering objectives,	19		

For peer review only

		limitations, multiplicity of analyses, results from similar studies, and other relevant evidence			
Generalisability	21	Discuss the generalisability (external validity) of the study results	19,20		
**Other Information**					
Funding	22	Give the source of funding and the role of the funders for the present study and, if applicable, for the original study on which the present article is based	20		
Accessibility of protocol, raw data, and programming code		..		RECORD 22.1: Authors should provide information on how to access any supplemental information such as the study protocol, raw data, or programming code.	21

*Reference: Benchimol EI, Smeeth L, Guttmann A, Harron K, Moher D, Petersen I, Sørensen HT, von Elm E, Langan SM, the RECORD Working Committee. The REporting of studies Conducted using Observational Routinely-collected health Data (RECORD) Statement. *PLoS Medicine* 2015; in press.

*Checklist is protected under Creative Commons Attribution (CC BY) license.

For peer review only

A longitudinal study of patients' health-related quality of life using EQ-5D-3L in 11 Swedish National Quality Registers

Journal:	*BMJ Open*
Manuscript ID	bmjopen-2020-048176.R1
Article Type:	Original research
Date Submitted by the Author:	29-Sep-2021

Complete List of Authors:	Teni, Fitsum; Karolinska Institutet, Health Outcomes and Economic Evaluation Research Group, Stockholm Centre for Healthcare Ethics, Department of Learning, Informatics, Management and Ethics<br>Rolfson, O; Sahlgrenska Academy, University of Gothenburg, Department of Orthopaedics, Institute of Clinical Sciences; Swedish Hip Arthroplasty Register<br>Devlin, Nancy; The University of Melbourne School of Population and Global Health, Health Economics; Office of Health Economics<br>Parkin, David; Office of Health Economics; City University of London<br>Nauclér, Emma; Swedish Hip Arthroplasty Register<br>Burström, Kristina; Karolinska Institutet, Health Outcomes and Economic Evaluation Research Group, Stockholm Centre for Healthcare Ethics, Department of Learning, Informatics, Management and Ethics; Region Stockholm, Health Care Services<br>the Swedish Quality Register (SWEQR) Study Group, na

**Primary Subject Heading**:	Patient-centred medicine
Secondary Subject Heading:	Patient-centred medicine, Health economics
Keywords:	ORTHOPAEDIC & TRAUMA SURGERY, HEALTH ECONOMICS, INTERNAL MEDICINE

SCHOLARONE™
Manuscripts

I, the Submitting Author has the right to grant and does grant on behalf of all authors of the Work (as defined in the below author licence), an exclusive licence and/or a non-exclusive licence for contributions from authors who are: i) UK Crown employees; ii) where BMJ has agreed a CC-BY licence shall apply, and/or iii) in accordance with the terms applicable for US Federal Government officers or employees acting as part of their official duties; on a worldwide, perpetual, irrevocable, royalty-free basis to BMJ Publishing Group Ltd ("BMJ") its licensees and where the relevant Journal is co-owned by BMJ to the co-owners of the Journal, to publish the Work in this journal and any other BMJ products and to exploit all rights, as set out in our licence.

The Submitting Author accepts and understands that any supply made under these terms is made by BMJ to the Submitting Author unless you are acting as an employee on behalf of your employer or a postgraduate student of an affiliated institution which is paying any applicable article publishing charge ("APC") for Open Access articles. Where the Submitting Author wishes to make the Work available on an Open Access basis (and intends to pay the relevant APC), the terms of reuse of such Open Access shall be governed by a Creative Commons licence – details of these licences and which Creative Commons licence will apply to this Work are set out in our licence referred to above.

Other than as permitted in any relevant BMJ Author's Self Archiving Policies, I confirm this Work has not been accepted for publication elsewhere, is not being considered for publication elsewhere and does not duplicate material already published. I confirm all authors consent to publication of this Work and authorise the granting of this licence.

For peer review only

A longitudinal study of patients' health-related quality of life using EQ-5D-3L in 11 Swedish National Quality Registers

Fitsum Sebsibe Teni[1*] (Doctoral student, ORCID 0000-0002-6182-499X, fitsum.teni@ki.se),

Ola Rolfson[1,2,3] (Professor, ORCID 0000-0001-6534-1242, ola.rolfson@vgregion.se),

Nancy Devlin[4,5] (Professor, ORCID 0000-0002-1561-5361, nancy.devlin@unimelb.edu.au),

David Parkin[5,6] (Senior Visiting Fellow, ORCID 0000-0002-9990-8208, d.parkin@city.ac.uk),

Emma Nauclér[3] (Senior Statistician, ORCID 0000-0003-1079-5655, emma.naucler@vgregion.se),

Kristina Burström[1,7,8] (Associate Professor, ORCID 0000-0001-9996-4317, kristina.burstrom@ki.se) and

The Swedish Quality Register (SWEQR) Study Group

Authors' affiliations

[1] Health Outcomes and Economic Evaluation Research Group, Stockholm Centre for Healthcare Ethics, Department of Learning, Informatics, Management and Ethics, Karolinska Institutet, Stockholm, Sweden.

[2] Department of Orthopaedics, Institute of Clinical Sciences, Sahlgrenska Academy, University of Gothenburg, Gothenburg, Sweden.

[3] Swedish Hip Arthroplasty Register, Gothenburg, Sweden.

[4] Centre for Health Policy, University of Melbourne, Melbourne, Australia.

[5] Office of Health Economics, London, United Kingdom.

[6] City University of London, London, United Kingdom.

[7] Equity and Health Policy Research Group, Department of Global Public Health, Karolinska Institutet, Stockholm; Sweden.

[8] Health Care Services, Region Stockholm, Stockholm, Sweden

The Swedish Quality Register (SWEQR) Study Group:

Allan Abbott (Associate Professor, Linköping University, Linköping, Sweden; ORCID 0000-0002-4318-9216; allan.abbott@liu.se),

Magnus Ekström (Associate Professor, Lund University, Lund, Sweden; ORCID 0000-0002-7227-5113; pmekstrom@gmail.com),

Magnus Forssblad (Associate Professor, Karolinska Institutet, Stockholm, Sweden; ORCID 0000-0002-5870-7477; magnus@forssblad.se),

Peter Fritzell (Associate Professor, Futurum Academy for Health and Care, Jönköping, Sweden/RKC Centre for spine surgery, Stockholm, Sweden; ORCID 0000-0002-2043-6376; peterfritzell@gmail.com),

Åsa Jonsson (RN, PhD, Ryhov County Hospital, Jönköping, Sweden; ORCID 0000-0002-3046-6333; asa.jonsson@rikssvikt.se),

Mikael Landén (Professor, University of Gothenburg, Gothenburg, Sweden; ORCID 0000-0002-4496-6451; mikael.landen@neuro.gu.se),

Michael Möller (Associate Professor, University of Gothenburg, Gothenburg, Sweden; ORCID 0000-0001-7394-6057; michael.moller@vgregion.se),

For peer review only

For peer review only - http://bmjopen.bmj.com/site/about/guidelines.xhtml

Malin Regardt (Reg OT, PhD, Swedish Rheumatology Quality Register (SRQ), Karolinska University Hospital, Stockholm; Karolinska Institutet, Stockholm, Sweden; ORCID 0000-0002-1908-5708; malin.regardt@sll.se, malin.regardt@ki.se),

Björn Rosengren (Professor, Lund University, Lund, Sweden; ORCID 0000-0001-5588-6927; bjorn.rosengren@med.lu.se),

Marcus Schmitt-Egenolf (Professor, Umeå University, Umeå, Sweden; ORCID 0000-0002-3858-8474; marcus.schmitt-egenolf@umu.se),

Johanna Vinblad (Project leader, Centre of Registers, Gothenburg, Sweden; ORCID 0000-0003-1842-7355; johanna.vinblad@vgregion.se),

Annette W-Dahl (Associate Professor, Lund University, Lund, Sweden; ORCID 0000-0003-4053-7358; annette.w-dahl@med.lu.se).

*Corresponding author

Fitsum Sebsibe Teni

Health Outcomes and Economic Evaluation Research Group
Stockholm Centre for Healthcare Ethics
Department of Learning, Informatics, Management and Ethics
Karolinska Institutet
SE-171 77 Stockholm, Sweden
E-mail: fitsum.teni@ki.se
Tel: +46764027138
ORCID: 0000 0002 6182 499X

For peer review only

Abstract

Objective: To compare problems reported in the five EQ-5D-3L dimensions and EQ VAS scores at baseline and at 1-year follow-up among different patient groups and specific diagnoses in 11 National Quality Registers (NQRs) and to compare these with the general population.

Design: Longitudinal, descriptive study

Participants: 261,241 patients from 11 NQRs and 49,169 participants from the general population were included in the study.

Primary and secondary outcome measures: Proportions of problems reported on the five EQ-5D-3L dimensions, EQ VAS scores of participants' own health and proportions of participants and mean/median EQ VAS score in the Paretian Classification of Health Change (PCHC) categories.

Results: In most of the included registers, and the general population, problems with pain/discomfort were the most frequently reported dimension at baseline and at 1-year follow-up. Mean EQ VAS score (SD) ranged from 45.2 (22.4) among disc hernia patients to 88.1 (15.3) in wrist and hand fracture patients at baseline. They ranged from 48.9 (20.9) in pulmonary fibrosis patients to 83.3 (17.4) in wrist and hand fracture patients at follow-up. The *improved* category of PCHC, improvement in at least one dimension without deterioration in any other, accounted for the highest proportion of observations in several diagnoses, corresponding with highest improvement in mean EQ VAS score.

Conclusions: The study documented self-reported health of several different patient groups using the EQ-5D-3L in comparing with the general population. This demonstrated the important role of PROs in routine clinical care, to assess and follow-up health status and progress within different groups of patients. The EQ-5D-3L descriptive system and EQ VAS have an important role in providing a 'common denominator', allowing comparisons across NQRs and specific diagnoses.

Trial registration number: ClinicalTrials.gov (NCT04359628)

For peer review only - http://bmjopen.bmj.com/site/about/guidelines.xhtml

Strengths and limitations

- The study presents findings including a large number of diagnoses across many patient groups.
- Data on health-related quality of life from large National Quality Registers and from the general population were included.
- Change in health-related quality of life from baseline to 1-year follow-up was reported using a longitudinal design.
- Differences in health-related quality of life due to different modes of data collection is possible.
- The analysis focused on patients with data both at baseline and 1-year follow-up.

For peer review only

For peer review only - http://bmjopen.bmj.com/site/about/guidelines.xhtml

Introduction

Patient-reported outcomes (PROs) are increasingly used in health care [1]. The health-related quality of life (HRQoL) instrument EQ-5D [2] is a generic (not disease-specific) PRO measure used worldwide for a wide range of conditions and treatments. The EQ-5D descriptive system consists of five dimensions of health: mobility, self-care, usual activities, pain/discomfort, and anxiety/depression [3]. The EQ VAS, a vertical line scaled from 0 ('worst imaginable health') to 100 ('best imaginable health') is a component of the EQ-5D.

Although EQ VAS plays a distinctive role in capturing patients' overall assessment of their own health it is seldom the focus of analyses. A study in the UK that assessed routine collection of EQ VAS found a consistent relationship between problems reported on the EQ-5D dimensions and the EQ VAS score [4]. Another study found it feasible to use the EQ VAS in clinical routine in patient diaries [5]. EQ VAS has also been employed in studies as part of the EQ-5D instrument in the assessment of HRQoL among patients with different diagnoses and in different settings [6–12].

The perception of problems experienced in the different dimensions and their responsiveness to health care intervention may differ depending on the nature and burden of the condition. Such studies addressing these areas could provide useful information to clinicians as well as policy makers in guiding the use of the EQ-5D descriptive system including the EQ VAS component in patient care which in some circumstances has led to improved care and patient outcome [13]. Improved care could be achieved through increased understanding of patients' conditions and monitoring improvements after treatment, with due consideration for PROs.

In Sweden, National Quality Registers (NQRs) hold data on individual patients' diseases treatment/intervention and outcomes [14]. There are over 100 NQRs across a wide range of clinical areas, dating as far back as 1975, when the Swedish Knee Arthroplasty Register was established. Most of the NQRs have been initiated by health care professionals [15]. A number of clinical areas are covered by the NQRs, with the aim of helping provide patients with the best possible care through, for example, monitoring of health care performance and quality, ongoing learning, and research [16]. The use of a personal identification number (PIN) in Sweden provides an advantage when linking data on patients from the NQRs with those collected within other government administered registers, such as the National Patient Register (NPR) [14]. There have been a number of efforts to use the various NQRs for quality improvement [17]. Most of the NQRs collect PRO data, the most common generic HRQoL instrument being the EQ-5D which is included in 40 registers [15]. Generic measures provide

For peer review only - http://bmjopen.bmj.com/site/about/guidelines.xhtml

a means of comparing health problems among patients in different NQRs, to understand the overall severity of problems experienced by patients with different diseases and treatment pathways. Many studies have included EQ VAS as part of the assessment of HRQoL using the EQ-5D among patients in NQRs. Examples of such studies include the influence of different surgical approaches to the hip on HRQoL [18], comparison after out-of-hospital and in-hospital cardiopulmonary resuscitation [19], as well as after hip replacement among patients born in Sweden and abroad [20], and self-reported health in patients with congenital heart diseases [21].

The present descriptive study provides comprehensive information on HRQoL, using the three-level EQ-5D-3L (no, some/moderate, confined to bed/unable to/extreme problems) resulting in 243 health profiles or health states, and the EQ VAS, of several patient groups with various diagnoses, a subject that has not been extensively examined in previous research. The overall aim of this study was to compare problems reported in the five EQ-5D-3L dimensions and EQ VAS scores at baseline and 1-year follow-up among different patient groups and specific diagnoses in 11 NQRs and to compare these with the general population. The objectives were:

- To document the prevalence of problems reported according to level of severity within the EQ-5D-3L dimensions and EQ VAS scores among different patient groups and diagnoses
- To document changes in the prevalence of problems reported according to level of severity within the EQ-5D-3L dimensions, and the change in EQ VAS scores among different patient groups and diagnoses
- To relate the prevalence of problems and mean EQ VAS scores to the characteristics of patients, diagnoses and interventions, at baseline, at 1-year follow-up, and changes over the follow-up and to make comparisons with the general population
- To calculate time-trade-off (TTO) and VAS indices for the diagnoses, and to establish EQ-5D-3L reference data for patients with the different diagnoses

For peer review only - http://bmjopen.bmj.com/site/about/guidelines.xhtml

Methods

Study Design

This is a descriptive study of longitudinal cohorts from 11 Swedish NQRs and comparison with data from the general population. This study forms part of a research project on the use of the EQ-5D questionnaire in different patient groups in Sweden [22]. The study is reported in line with the RECORD checklist [23].

Swedish National Quality Registers included in the study

The NQRs included in this study comprise a selection of different disease areas where EQ-5D-3L data are collected. In addition to musculoskeletal conditions, where EQ-5D-3L is most used, our sample also represents patients with heart failure, respiratory failure and psoriasis. The Better management of patients with Osteoarthritis (BOA) register is referred to as BOA throughout the manuscript (Supplementary Table S1).

Data from the registers on PROs, Body Mass Index (BMI) and demographic characteristics, at baseline, and at 1-year follow-up were included. A 1-year follow-up was chosen as it is the most common evaluation time in the NQRs which facilitated comparison between them. In registers with no such information, we used the date of data collection to determine the time points. EQ-5D-3L data collected within 90 days of one calendar year after baseline were categorized as 1-year follow-up data, following the approach employed in the Swedish Hip Arthroplasty Register. In the case of patients from the fracture register, baseline data on EQ-5D-3L were collected within four weeks from the fracture date, by recall of the week before the fracture occurred.

Sampling and data

Baseline/first visit and 1-year follow-up data from 2001 to 2019 were retrieved from the registers. Patient records including EQ-5D-3L questionnaire data were selected. Records with complete data on main diagnosis/diagnosis groups and interventions were selected among these. Prior to pooling data from the registers, patients with records in more than one register were randomly de-duplicated. After the exclusion of duplicate records, a total of 266,241 patient records, with complete data on age and sex at baseline, at time of diagnosis and intervention, and on EQ-5D-3L dimensions were included in the analysis of EQ-5D-3L dimensions. In the analysis of EQ VAS data, 209,247 records with complete data were included (Supplementary Table S1).

For peer review only
For peer review only - http://bmjopen.bmj.com/site/about/guidelines.xhtml

The patient data were compared with the general population, using data on 49,169 individuals with complete data on EQ-5D-3L dimensions and 41,761 with complete EQ VAS data. This data was employed to develop the Swedish experience-based EQ-5D-3L value sets [24]. The general population data came from population surveys conducted in two regions in Sweden, Scania in 2004 and Stockholm in 2006, they are in terms of basic characteristics broadly representative of the Swedish population [24]. The self-administered questionnaires covered around 100 questions on living conditions and self-reported health of participants [24–27]. In the present study, for comparison with the findings from the different patient groups, data on demographic and the EQ-5D-3L questionnaire on 49,169 participants were included.

Age was categorized into <30, 30–39, 40–49, 50–59, 60–69, 70–79 and ≥80 years. BMI (weight in kilograms divided by a square meters of height) was categorized as underweight (<18.5), normal weight (18.5–24.9), overweight (25.0–29.9) and obesity class I (30–34.9), II (35.0–39.9) or III (40+) [28].

Data analysis

Descriptive analysis was performed on age, sex, BMI categories and data on EQ-5D-3L dimensions. The prevalence of problems reported by patients were presented by register (patient group) and by diagnosis, at baseline and 1-year follow-up, and for data from the general population. In addition, to make comparisons taking age and sex difference into account, problems reported among patients in the different registers and the general population were presented for women and men categorized by age groups.

Descriptive statistics (mean (SD) and median (IQR)) were also used to present EQ VAS score at baseline, at 1-year follow-up, as well as for the change in score, by register, diagnosis, intervention and by age group, sex, and BMI category in each register. An analysis of covariance (ANCOVA) was performed to make comparisons of the estimated mean EQ VAS scores by sex, adjusted for age in each register.

For the data at baseline and at 1-year follow-up, a two-level random intercept model (as there could be variations based on the register a diagnosis is grouped in) was conducted to assess how each diagnosis in the different registers influences mean EQ VAS score with the general population data as reference group. This analysis was performed on the pooled (combined) data of the all the patient groups and the general population for each of the baseline and the 1-year follow-up. The estimates were adjusted for age group and sex. In the statistical analyses, a 95% confidence interval (CI) was employed.

For peer review only - http://bmjopen.bmj.com/site/about/guidelines.xhtml

The methods described above for analyses of the NQR data were applied on the general population data.

Paretian Classification of Health Change (PCHC), which categorizes changes in health states over time into *no problem*, *no change*, *improved*, *worsened* and *mixed* was performed using baseline and 1-year follow-up data on health states [29]. Individuals who reported the same health state at both time points (including, but not limited to, health state 11111, i.e. no problems in any of the dimensions) were included in the *no change* category. Improvement in any of the EQ-5D-3L dimensions with no deterioration in the remaining ones leads to categorization as *improved.* On the contrary, a worsening in any of the EQ-5D-3L dimensions with no improvement in the remaining dimensions was categorized as *worsened.* Health states characterized by mixed types of changes (both improved and worsened) were grouped in the *mixed* category [29]. By using this categorisation, the proportion of improvements and worsening, as well as the other categories, were calculated for each diagnosis. Similarly, the mean self-assessed EQ VAS scores in these PCHC groups at baseline and 1-year follow-up were calculated according to diagnosis.

As a sensitivity analysis, the demographic characteristics of patients with complete baseline and 1-year follow up data included in the analysis, and those with missing records at 1-year follow-up were compared for each register.

In order to assess the HRQoL profile of patients with complete data at baseline (regardless of follow-up status) and also patients with complete data at 1-year follow-up (regardless of their baseline status), prevalence of problems reported on the EQ-5D-3L dimensions and mean EQ VAS score were calculated. In addition, the EQ-5D-3L data were summarized as EQ-5D-3L indices using the Swedish experience-based EQ-5D-3L TTO and VAS value sets [24].

Patient and public involvement

Neither patients nor the general public were involved in the design, conduct, reporting, or dissemination plans of our research.

For peer review only - http://bmjopen.bmj.com/site/about/guidelines.xhtml

Results

Demographic characteristics

A total of 266,241 patient records and 49,169 records from general population were included in the study (Supplementary Table S1). The mean age of included participants ranged from 29.8 years in patients with cruciate ligament injury, to 73.7 years for patients with respiratory failure and 72.8 years in heart failure patients (Table 1). Most of the patients were in their 50s to 70s. However, among heart failure patients nearly one-third were 80 years or older and among the cruciate ligament injury patients the majority were below 30 years of age. The mean age of the general population was 46.2 years (Table 1). A majority of the participants were women, with the exception of patients with psoriasis, heart failure, as well as ankle and cruciate ligament injury. In almost all of the NQRs, a majority of patients had a BMI in the normal weight and overweight categories (Table 1; Supplementary Table S2).

Problems reported on the EQ-5D-3L dimensions

Table 2 presents the prevalence of problems reported on the EQ-5D-3L dimensions across NQRs, at baseline and 1-year follow-up. At both time points, similar to the general population, most problems reported were in the pain/discomfort dimension, with the exception of the respiratory failure patients, where most problems reported concerned mobility. Patients in BOA and those with spine, ankle, knee and hip conditions had the highest prevalence of pain/discomfort of the NQRs included. At the 1-year follow-up, the highest proportion of problems reported were among BOA, rheumatology and spine patients. In most registers, at both baseline and 1-year follow-up, problems with mobility were the second most prevalent.

At both time points, the highest proportion of severe problems reported were regarding pain/discomfort. This was the case for all registers as well as in the general population, with the exception of patients with fracture and respiratory failure, for which the highest proportion of severe problems concerned the usual activities dimension. In addition, patients with cruciate ligament injury at 1-year follow-up reported the highest proportion of severe problems in the anxiety/depression dimension (Table 2).

The prevalence of problems reported in the EQ-5D-3L dimensions by patients according to specific diagnoses across NQRs is presented at baseline and at 1-year follow-up (Supplementary Table S3). For all diagnoses, with exception of COPD and pulmonary fibrosis, most problems were reported with pain/discomfort. This was most commonly seen at baseline in the intervention-based registers covering musculoskeletal diseases, with the

For peer review only - http://bmjopen.bmj.com/site/about/guidelines.xhtml

highest proportion among patients concerning a diagnosis of rheumatoid arthritis in the knee (100%). Patients with rheumatoid arthritis of the ankle had the highest prevalence of severe problems in the pain/discomfort (69%) dimension. Among patients with COPD and pulmonary fibrosis, the highest prevalence of problems was in the mobility dimension. At 1-year follow-up, the highest proportion of severe problems were reported in the pain/discomfort dimension for most diagnoses, except for patients with lower extremity and axial fractures and for patients with COPD and pulmonary fibrosis.

Severe problems in the anxiety/depression dimension were most prevalent among patients with COPD, both at baseline (9%) and 1-year follow-up (10%). The response, “confined to bed”, within the mobility dimension was very uncommon among all diagnoses, with the largest proportion occurring in patients with disc hernia (5%) at baseline and those with fracture on hip and thigh (5%) as well as COPD and pulmonary fibrosis (5%) at 1-year follow-up. The prevalence of severe problems with self-care was highest at 1-year follow-up in patients with a hip or femoral fracture (10%) (Supplementary Table S3).

Furthermore, the proportion of problems reported on the EQ-5D-3L among women and men categorized by age groups in the 11 NQRs at baseline and 1-year follow-up and in the general population is presented in supplementary material Figure S1 and S2.

Paretian Classification of Health Change (PCHC)

Figure 1 presents the proportions of patients by PCHC group for the different diagnoses. The highest proportions of improvements were shown for patients in the intervention-based ankle, hip, knee and spine registers. For most of the other diagnoses, the highest proportions of patients were classified as *improved*. The respiratory failure patient group was an exception, of whom most were classified as *worsened*. Another exception was the fracture patient group where the highest proportion of patients were classified as *worsened*, as their baseline data were collected by recall of the week before the fracture occurred. Most fracture patients had a better health state at baseline than at 1-year follow-up. The heart failure patients had similar proportions of patients classified as *improved* and *worsened*.

Mean self-assessed EQ VAS score

Mean (Table 3) and median (Supplementary Table S4) self-assessed EQ VAS scores by sex, age groups, and BMI categories are described for each register at baseline, 1-year follow-up, the change over the period, and for the general population. The highest mean score at both time points was found among fracture patients (84.7 (baseline) and 77.7 (1 year)

For peer review only - http://bmjopen.bmj.com/site/about/guidelines.xhtml

respectively). The lowest scores were found among spine patients (47.8) at baseline, and respiratory failure patients (49.1) at 1-year follow-up. The mean EQ VAS score in the general population was 79.5. The scores were found to have increased in all patients at the 1-year follow-up, with the exception of respiratory failure patients and fracture patients. The highest increases were recorded among hip (20.5) and spine surgery (20.1) patients.

Similar to the general population, in most registers, women had statistically significantly lower mean EQ VAS scores after adjustment for age in the ANCOVA. Exceptions were seen among fracture and respiratory failure patients at baseline and BOA, fracture, heart failure and respiratory failure patients at 1-year follow-up (Table 4). The mean EQ VAS score showed greater improvements among women in most of the registers, with the exception of ankle, cruciate ligament injury, heart and respiratory failure patients.

In most registers with BMI data, the highest mean EQ VAS scores were found in the normal and overweight categories, at both time points. However, no clear pattern was shown in terms of the change in mean EQ VAS score (Table 3).

Figure 2 describes mean EQ VAS score by diagnosis at baseline, and 1-year follow-up. Mean EQ VAS scores had more similarity among diagnoses within the same patient group (register) than with diagnoses in other patient groups. Some differences were shown by diagnosis in some patient groups such as fracture (e.g. hip and thigh) and hip (e.g. inflammatory joint disorder) which were illustrated to be statistically significant in the results of the two-level random intercept model in Figure 3. For most of the diagnoses, EQ VAS scores increased over the follow-up period; however, they decreased among patients with respiratory failure and fracture.

Figure 3 illustrates the two-level random intercept model estimates of mean EQ VAS scores for the different diagnoses, adjusted for age and sex, with the general population as a reference group. At baseline, patients with diagnoses other than fractures showed statistically significantly lower mean EQ VAS scores as compared with the general population. At 1-year follow-up, all patients, except those with BOA hip, fracture, and respiratory failure diagnosis showed improvement in mean EQ VAS, while remaining lower than in the general population with the exception of fracture patients. A table form of the regression analysis is available in supplementary Table S5.

Figure 4 presents mean EQ VAS scores, at baseline and 1-year follow-up, in the various PCHC categories within the different diagnoses. Mean EQ VAS scores showed corresponding

For peer review only - http://bmjopen.bmj.com/site/about/guidelines.xhtml

increases and decreases in the categories, *improved* and *worsened*. The largest increases in the *improved* category were found among diagnoses in the hip and spine patient groups. In the *worsened* category, diagnoses in the fracture patient groups showed the largest decrements, based on their recall of their health status before the fracture occurred. In the categories, *no change* and *mixed*, increase in mean EQ VAS score was found in all diagnoses, with the exception of the fracture and respiratory failure patient groups, which instead showed decrements.

A comparison of patient records with complete data at baseline and at 1-year follow-up with data from patients with only baseline showed that higher proportions of women and older patients had complete baseline and 1-year follow-up data in many of the registers (Results available on request).

Problems reported on EQ-5D-3L dimensions and mean self-assessed EQ VAS score among patients at baseline (regardless of follow up status), among those with 1-year follow-up data (regardless of baseline status), and for the general population were calculated (Supplementary Table S6 and Supplementary Table S7). The tables also present EQ-5D-3L indices based on the Swedish experience-based TTO and VAS value sets [26]. The results presented in Table S6 can serve as EQ-5D-3L reference data for patients with these diagnoses, with the exception of the fracture patients as their baseline data comes from before the fracture date. The pattern of problems reported, and mean EQ VAS scores, were comparable to those reported at the corresponding time point among patients with data both at baseline and at 1-year follow-up.

For peer review only - http://bmjopen.bmj.com/site/about/guidelines.xhtml

Discussion

Main findings

This study documented self-reported health status in different patient groups, and for specific diagnoses in the NQRs, as well as in the general population. Higher proportions of problems and lower self-assessed EQ VAS scores (worse overall HRQoL) were reported among patients with diagnoses with more symptoms experienced captured by the EQ-5D-3L dimensions. In general, most problems were reported in the pain/discomfort dimension.

Comparisons with other studies

The proportions of any problems reported on the EQ-5D-3L dimensions, and distribution between *some* and *severe* levels of problem, seem to vary depending on the differences in the symptoms of the diseases as well as the way in which they are experienced by the patients [30–34]. Specifically, patients in the intervention-based registers, such as spine, hip, and knee, showed the highest proportions of problems in the EQ-5D-3L dimensions. This was particularly the case regarding the pain/discomfort dimension, which reflects the pain symptoms associated with the diseases [31–34]. Pain/discomfort was similarly the dimension with the highest proportion of problems reported, also in the general population. Substantially less reported problems were noted after surgical interventions in patients from intervention-based registers. The high proportions of problems within the mobility and usual activities dimensions in COPD and pulmonary fibrosis patients might be due to limitations in mobility and other functions and, in some cases, the need to use breathing equipment. Other studies of patients with respiratory diseases also reported comparably high proportions of problems within the mobility, usual activities and pain/discomfort dimensions [30,35].

The EQ-5D-3L dimensions for which higher proportions of problems were reported seem to be partly determined by the type of symptoms/manifestations of the disease in patients, as indicated above. For instance, when comparing spine and psoriasis patients, the proportion and severity of problems in both the pain/discomfort and mobility dimensions were much lower for the psoriasis patients. This provided evidence for the ability of the EQ-5D-3L to discriminate among conditions that affect specific aspects of health differently, although some dimensions more relevant to some diseases may not be covered. Even in comparison to heart failure patients, who were on average much older than spine patients, higher proportions of problems with pain/discomfort and mobility were reported in spine patients. The low prevalence of severe problems (“confined to bed”) with mobility might indicate the importance of applying the five-level EQ-5D-5L among these patient groups [36].

For peer review only - http://bmjopen.bmj.com/site/about/guidelines.xhtml

Regarding the anxiety/depression dimension, in most of the registers fewer than half of the patients reported problems, with the exception of spine, cruciate ligament injury, respiratory failure and psoriasis patients at baseline, and respiratory failure patients at 1-year follow-up. Compared to the general population, the proportion of problems with anxiety/depression was higher than in most of the registers at baseline. NQRs in which patients reported higher proportions of problems mainly on the pain/discomfort and mobility dimensions also had higher proportions of problems with anxiety/depression. This may be related to the pain, reduced walking ability and impaired ability to perform everyday activities they reported, which might affect the mood dimension. The interrelated nature of the EQ-5D dimensions has been reported for the EQ-5D-5L assessing causal and effect relationships, where empirical evidence showed mobility to be a causal indicator [37]. The greatest problems in the mood dimension were reported among respiratory and spine patients at baseline and 1-year follow-up, and in rheumatology patients at 1-year follow-up. This could be related to the very restrictive nature of the diseases [31,35,38]. Other studies have also shown that more than half of patients with severe COPD, as well as those undergoing spinal surgery, report problems on the anxiety/depression dimension [39,40].

When comparing problems reported among patients in the different registers on the EQ-5D-3L dimensions to the general population, one must take into account that there could be comorbid illnesses among patients and morbidities in the general population. Inline with this, public health survey reports and studies showed that, among the general population sample in the present study, considerable levels of different health problems were reported including cardiovascular diseases, diabetes, mental health problems such as depression, reports of pain in the neck, shoulders, arms and back; as well as allergic eye and nose problems [41–43].

The longitudinal change in reported problems in the dimensions showed that, while improvement was observed in most of the NQRs, larger improvements were reported in intervention-based registers. These patients also reported the highest proportions of problems at baseline and were subsequently provided with surgical interventions. Among these were hip, ankle, knee and spine patients. This pattern was also observed in the specific diagnoses within the various patient groups and PCHC categories. PCHC has been employed in other studies to document change over time in various diseases, such as heart failure, prostate cancer, diabetes mellitus and overactive bladder [44–48]. The change for the fracture patients cannot be compared directly to changes for other patient groups due to the recall technique used for health state before injury.

For peer review only - http://bmjopen.bmj.com/site/about/guidelines.xhtml

The mean self-assessed EQ VAS scores showed variation across NQRs and diagnoses, with the lowest scores reported among patient groups with symptoms captured by the EQ-5D-3L dimensions. In almost all registers, the EQ VAS scores were lower, both at baseline and at 1-year follow-up, compared to findings in the general population data, for which the mean score was 79.5 [24]. The EQ VAS scores from the registers demonstrated consistency with problems reported in the EQ-5D-3L dimensions. For example, spine and respiratory failure patients, who reported the highest proportions of problems at baseline, also had the lowest mean EQ VAS scores. In a study that assessed the performance of EQ-5D among patients with irritable bowel syndrome, EQ VAS score also was found to differ by level of severity [49]. The change in mean EQ VAS score also corresponded with changes in the problems reported in the descriptive system. This was shown by the largest increases in EQ VAS score among spine and hip patients. As the baseline level determines the room for improvement, the low baseline scores among these patient groups may have partly contributed to the large increments shown. The findings were in line with previous studies demonstrating the consistency of the EQ-5D descriptive system with the mean EQ VAS score [4,50].

The consistent pattern of changes in the prevalence of problems reported in the EQ-5D-3L and the corresponding mean EQ VAS scores was also shown in the PCHC categories. This was particularly evident in an increase in the proportion of patients that were classified as *improved*, and a decrease in the proportion classified as *worsened*, in terms of EQ VAS scores. However, as also discussed in previous studies [4,51], a pattern which might partly be attributed to the broader construct in EQ VAS than the EQ-5D-3L descriptive system, was shown in the *no problem* category. In this category, mean EQ VAS scores increased over the 1-year follow-up, though they differed by diagnosis. The PCHC is potentially useful as a simple means of summarising changes using profile data without need for a value set. But, if there is a high proportion of patients classified as *mixed*, the method is less useful. PCHC does not inform on the magnitude of improvement or worsening in HRQoL.

The lower mean EQ VAS scores, consistently lower among women in most of the NQRs, were similar to findings reported in the literature among patients with different diseases, as well as in the general population [52–54]. However, comparable findings on EQ VAS by sex were also reported [55]. Greater improvement was nevertheless found among women in many of the patient groups over the period up to the 1-year follow-up.

The variation in EQ VAS scores may be due to differences in the symptoms experienced in the different patient groups, as indicated by age- and sex-adjusted two-level random intercept

For peer review only - http://bmjopen.bmj.com/site/about/guidelines.xhtml

model estimates. In most diagnoses, lower EQ VAS score than in the general population were shown at baseline, and these improved at 1-year follow-up. The mean EQ VAS score across diagnoses also showed a general pattern of more similar scores among diagnoses within the same NQR (patient group) than those across the different NQRs. One possible reason for this could be attributed to the similarity of diagnoses and symptoms in the same register.

Strengths and limitations of the study

One of the strengths of the present study is the coverage of several diagnoses across different patient groups in the NQRs. The use of large data sets, providing information on the HRQoL of patients, together with data from the general population, made it possible to conduct relevant comparisons. Furthermore, the study assessed longitudinal change in HRQoL of patients from baseline to 1-year follow-up, making comparisons across the different NQRs. In addition, the study adds new data on patients with diagnoses that have previously been less studied. One limitation of the present study may be related to possible differences in the reported HRQoL arising due to different modes of data collection employed across NQRs. A second limitation relates to comorbidities among patients beside the main diagnoses and morbidities in the general population and how this may affect the comparison in the HRQoL across patient groups and with the general population data, not addressed fully in the present study. Another limitation relates to the inclusion of patients having both data at baseline and at 1-year follow-up, while excluding non-respondents, those with only baseline or 1-year follow-up data, and patients with incomplete entries. This may introduce bias. However, separate analyses of baseline data (regardless of 1-year follow-up) and 1-year follow-up data (regardless of baseline status) showed findings consistent with those included in the main analysis. Furthermore, in a study among patients who underwent THR, 1-year and 6-years follow-up data showed generally comparable prevalence of problems in EQ-5D-3L dimensions and similar mean EQ VAS scores [56]. Analyses of non-responders at the 1-year follow-up showed statistically significant but small differences in sex and age, as compared to respondents with follow-up data in many registers.

Implications of the findings

The present study contributes by documenting HRQoL among several patient groups, as compared to the general population. It adds to the literature in the area where studies covering various diagnoses have not been widely conducted. By using routinely collected comprehensive data from a number of NQRs in Sweden, the study also adds to previous findings on the consistency between problems reported on the EQ-5D-3L dimensions and the

For peer review only - http://bmjopen.bmj.com/site/about/guidelines.xhtml

EQ VAS. This is a useful addition to the literature, showing the importance of using the EQ VAS to describe the overall health from the patient perspective, using relatively concise data. This can be valuable in monitoring the health status and progress of different subgroups (age, sex, clinical stages) of patients. The present study demonstrated an important feature of the EQ-5D-3L dimensions and the EQ VAS, showing differences across diagnoses.

For peer review only

For peer review only - http://bmjopen.bmj.com/site/about/guidelines.xhtml

Conclusions

The present study contributes to the literature by documenting self-reported health status for several different patient groups and diagnoses using data collected through the EQ-5D-3L questionnaire, and comparing these with data on the general population. The consistency between problems reported on the EQ-5D-3L dimension and the EQ VAS score demonstrated component corresponding patterns at baseline and at 1-year follow-up.

This study shows the important role of PROs in routine clinical care, to assess and follow-up health status and progress within different groups of patients. Together with clinical data, this could provide crucial information for understanding and improving the patients' health status.

Contributors: KB and OR, conceived the study. FST, OR, KB, ND, DP, EN and members of the SWEQR Study Group (AA, ME, MF, PF, ÅJ, ML, MM, MR, BR, MSE, JV, AWD) designed the study. FST performed the data analysis and preliminary interpretation of the data. KB, OR, ND, DP and EN supervised data analysis and interpretation of findings. FST drafted the manuscript. KB, OR, ND, DP, EN and members of the SWEQR Study Group (AA, ME, MF, PF, ÅJ, ML, MM, MR, BR, MSE, JV, AWD) revised the draft manuscript for important intellectual content. The corresponding author attests that all the authors in the list fulfil the authorship criteria and no other who fulfil the criteria were left out. All authors approved the submission of the manuscript. All the authors agree to be responsible for all aspects of the manuscript.

Funding: This research project is supported by a grant from The EuroQol Research Foundation (EQ Project # 2016480) and Region Stockholm (former Stockholm County Council) (# 4-3464/2018), Stockholm, Sweden. The funding bodies did not influence the design, conduct and reporting of the study.

Competing interests: KB, ND and DP are members of the EuroQol Group. KB reports grants from EuroQol Research Foundation, grants from Region Stockholm, during the conduct of the study; personal fees from Region Stockholm, outside the submitted work. ML reports personal fees from Lundbeck pharmaceuticals, outside the submitted work. OR reports institutional compensation for educational consultancy from Link Sweden; institutional compensation for research consultancy from Pfizer, outside the submitted work. AA, ME, MF, PF, ÅJ, MM, EN, MR, BR, MSE, FST, AWD and JV declare no competing interests.

Ethical approval: Ethical approval (# 1185-18/2019-00812 and #2020-04369) was obtained from the Regional Ethics Review Board in Gothenburg, Sweden, for the data from the

For peer review only - http://bmjopen.bmj.com/site/about/guidelines.xhtml

National Quality Registers and from the Swedish Ethical Review Authority (# 2020-03090) for the general population data.

Data availability statement: Data sharing is not possible according to Swedish law.

Author License: I, the Submitting Author has the right to grant and does grant on behalf of all authors of the Work (as defined in the author licence), an exclusive licence and/or a non-exclusive licence for contributions from authors who are: i) UK Crown employees; ii) where BMJ has agreed a CC-BY licence shall apply, and/or iii) in accordance with the terms applicable for US Federal Government officers or employees acting as part of their official duties; on a worldwide, perpetual, irrevocable, royalty-free basis to BMJ Publishing Group Ltd ("BMJ") its licensees.

For peer review only

For peer review only - http://bmjopen.bmj.com/site/about/guidelines.xhtml

References

1 Appleby J, Devlin N, Parkin D. *Using Patient Reported Outcomes to Improve Health Care*. John Wiley & Sons 2015.

2 Brooks R, Boye KS, Slaap B. EQ-5D: a plea for accurate nomenclature. *Journal of Patient-Reported Outcomes* 2020;**4**:52. doi:10.1186/s41687-020-00222-9

3 Rabin R, de Charro F. EQ-5D: a measure of health status from the EuroQol Group. *Ann Med* 2001;**33**:337–43. doi:10.3109/07853890109002087

4 Feng Y, Parkin D, Devlin NJ. Assessing the performance of the EQ-VAS in the NHS PROMs programme. *Qual Life Res* 2014;**23**:977–89.

5 Parkin D, Rice N, Jacoby A, *et al.* Use of a visual analogue scale in a daily patient diary: modelling cross-sectional time-series data on health-related quality of life. *Soc Sci Med* 2004;**59**:351–60. doi:10.1016/j.socscimed.2003.10.015

6 Sari S, Bilberg R, Søgaard Nielsen A, *et al.* The effect of exercise as adjunctive treatment on quality of life for individuals with alcohol use disorders: a randomized controlled trial. *BMC Public Health* 2019;**19**:727. doi:10.1186/s12889-019-7083-8

7 Boczor S, Daubmann A, Eisele M, *et al.* Quality of life assessment in patients with heart failure: validity of the German version of the generic EQ-5D-5L™. *BMC Public Health* 2019;**19**:1464. doi:10.1186/s12889-019-7623-2

8 Roman A, Barbera JA, Castillo MJ, *et al.* Health-Related Quality of Life in a National Cohort of Patients With Pulmonary Arterial Hypertension or Chronic Thromboembolic Pulmonary Hypertension. *Arch Bronconeumol* 2013;**49**:181–8. doi:10.1016/j.arbr.2012.12.002

9 Tan Z, Liang Y, Liu S, *et al.* Health-related quality of life as measured with EQ-5D among populations with and without specific chronic conditions: a population-based survey in Shaanxi Province, China. *PLoS ONE* 2013;**8**:e65958. doi:10.1371/journal.pone.0065958

10 Stark RG, Reitmeir P, Leidl R, *et al.* Validity, reliability, and responsiveness of the EQ-5D in inflammatory bowel disease in Germany. *Inflamm Bowel Dis* 2010;**16**:42–51. doi:10.1002/ibd.20989

11 Szentes BL, Schwarzkopf L, Kirsch F, *et al.* Measuring quality of life in COPD patients: comparing disease-specific supplements to the EQ-5D-5L. *Expert Rev Pharmacoecon Outcomes Res* 2019;:1–7. doi:10.1080/14737167.2019.1662302

12 von Vogelsang A-C, Burström K, Wengström Y, *et al.* Health-Related Quality of Life 10 Years After Intracranial Aneurysm Rupture: A Retrospective Cohort Study Using EQ-5D. *Neurosurgery* 2013;**72**:397–406. doi:10.1227/NEU.0b013e3182804686

13 Guyatt GH, Ferrans CE, Halyard MY, *et al.* Exploration of the value of health-related quality-of-life information from clinical research and into clinical practice. *Mayo Clin Proc* 2007;**82**:1229–39. doi:10.4065/82.10.1229

14 Nationella Kvalitetsregister. About the National Quality Registries. 2019.http://kvalitetsregister.se/englishpages/aboutqualityregistries.2422.html (accessed 22 Jul 2020).

For peer review only - http://bmjopen.bmj.com/site/about/guidelines.xhtml

15 Emilsson L, Lindahl B, Köster M, *et al.* Review of 103 Swedish Healthcare Quality Registries. *J Intern Med* 2015;**277**:94–136. doi:10.1111/joim.12303

16 Whynes DK. Does the correspondence between EQ-5D health state description and VAS score vary by medical condition? *Health Qual Life Outcomes* 2013;**11**:155.

17 Fredriksson M, Halford C, Eldh AC, *et al.* Are data from national quality registries used in quality improvement at Swedish hospital clinics? *Int J Qual Health Care* 2017;**29**:909–15. doi:10.1093/intqhc/mzx132

18 Lindgren JV, Wretenberg P, Kärrholm J, *et al.* Patient-reported outcome is influenced by surgical approach in total hip replacement: a study of the Swedish Hip Arthroplasty Register including 42,233 patients. *Bone Joint J* 2014;**96-B**:590–6. doi:10.1302/0301-620X.96B5.32341

19 Djärv T, Bremer A, Herlitz J, *et al.* Health-related quality of life after surviving an out-of-hospital compared to an in-hospital cardiac arrest: A Swedish population-based registry study. *Resuscitation* 2020;**151**:77–84. doi:10.1016/j.resuscitation.2020.04.002

20 Krupic F, Garellick G, Gordon M, *et al.* Different patient-reported outcomes in immigrants and patients born in Sweden. *Acta Orthopaedica* 2014;**85**:221–8. doi:10.3109/17453674.2014.919556

21 Berghammer M, Karlsson J, Ekman I, *et al.* Self-reported health status (EQ-5D) in adults with congenital heart disease. *Int J Cardiol* 2013;**165**:537–43. doi:10.1016/j.ijcard.2011.10.002

22 Teni FS, Rolfson O, Devlin N, *et al.* Variations in Patients' Overall Assessment of Their Health Across and Within Disease Groups Using the EQ-5D Questionnaire: Protocol for a Longitudinal Study in the Swedish National Quality Registers. *JMIR Research Protocols* 2021;**10**:e27669. doi:10.2196/27669

23 Benchimol EI, Smeeth L, Guttmann A, *et al.* The REporting of studies Conducted using Observational Routinely-collected health Data (RECORD) Statement. *PLOS Medicine* 2015;**12**:e1001885. doi:10.1371/journal.pmed.1001885

24 Burström K, Sun S, Gerdtham U-G, *et al.* Swedish experience-based value sets for EQ-5D health states. *Qual Life Res* 2014;**23**:431–42. doi:10.1007/s11136-013-0496-4

25 Lindgren A, Björk J, Stroh E, *et al.* Adult asthma and traffic exposure at residential address, workplace address, and self-reported daily time outdoor in traffic: A two-stage case-control study. *BMC Public Health* 2010;**10**:716. doi:10.1186/1471-2458-10-716

26 Svensson AC, Fredlund P, Laflamme L, *et al.* Cohort profile: The Stockholm Public Health Cohort. *Int J Epidemiol* 2013;**42**:1263–72. doi:10.1093/ije/dys126

27 Stockholms läns landsting. Hälsoenkät 2006-En undersökning om hälsa och levnadsförhållanden i Stockholms län. 2006.http://dok.slso.sll.se/CES/FHG/Folkhalsoarbete/Halsa%20Stockholm/Enkat-2006unga.pdf (accessed 22 Aug 2021).

28 WHO consultation on obesity. Obesity: Preventing and managing the global epidemic, Report of a WHO Consultation. Geneva, Switzerland: : WHO 2000. http://whqlibdoc.who.int/trs/WHO_TRS_894.pdf. (accessed 22 Jul 2020).

For peer review only - http://bmjopen.bmj.com/site/about/guidelines.xhtml

29 Devlin NJ, Parkin D, Browne J. Patient-reported outcome measures in the NHS: new methods for analysing and reporting EQ-5D data. *Health Econ* 2010;**19**:886–905. doi:10.1002/hec.1608

30 Hazell M, Frank T, Frank P. Health related quality of life in individuals with asthma related symptoms. *Respiratory Medicine* 2003;**97**:1211–8. doi:10.1016/S0954-6111(03)00249-X

31 Melancia JL, Francisco AF, Antunes JL. Spinal stenosis. *Handb Clin Neurol* 2014;**119**:541–9. doi:10.1016/B978-0-7020-4086-3.00035-7

32 Humphreys SC, Eck JC. Clinical Evaluation and Treatment Options for Herniated Lumbar Disc. *AFP* 1999;**59**:575.

33 Lespasio MJ, Sultan AA, Piuzzi NS, *et al.* Hip Osteoarthritis: A Primer. *Perm J* 2018;**22**. doi:10.7812/TPP/17-084

34 Lespasio MJ, Piuzzi NS, Husni ME, *et al.* Knee Osteoarthritis: A Primer. *Perm J* 2017;**21**. doi:10.7812/TPP/16-183

35 Kim S-H, Oh YM, Jo M-W. Health-related quality of life in chronic obstructive pulmonary disease patients in Korea. *Health Qual Life Outcomes* 2014;**12**:57. doi:10.1186/1477-7525-12-57

36 Herdman M, Gudex C, Lloyd A, *et al.* Development and preliminary testing of the new five-level version of EQ-5D (EQ-5D-5L). *Qual Life Res* 2011;**20**:1727–36. doi:10.1007/s11136-011-9903-x

37 Gamst-Klaussen T, Gudex C, Olsen JA. Exploring the causal and effect nature of EQ-5D dimensions: an application of confirmatory tetrad analysis and confirmatory factor analysis. *Health Qual Life Outcomes* 2018;**16**:153. doi:10.1186/s12955-018-0975-y

38 Lee DM, Weinblatt ME. Rheumatoid arthritis. *The Lancet* 2001;**358**:903–11. doi:10.1016/S0140-6736(01)06075-5

39 Chotai S, Khan I, Nian H, *et al.* Utility of Anxiety/Depression Domain of EQ-5D to Define Psychological Distress in Spine Surgery. *World Neurosurg* 2019;**126**:e1075–80. doi:10.1016/j.wneu.2019.02.211

40 Menn P, Weber N, Holle R. Health-related quality of life in patients with severe COPD hospitalized for exacerbations - comparing EQ-5D, SF-12 and SGRQ. *Health Qual Life Outcomes* 2010;**8**:39. doi:10.1186/1477-7525-8-39

41 Rosvall M, Khan FA, Nilsson M, *et al.* Hälsoförhållanden i Skåne - Folkhälsoenkät Skåne 2004. Malmö: : Socialmedicinska enheten, Region Skåne 2005. https://utveckling.skane.se/siteassets/publikationer_dokument/fh_2004_rapport.pdf (accessed 22 Aug 2021).

42 Stockholms läns landsting. Folkhälsan i Stockholms län 2007- Folkhälsorapport 2007. Stockholm, Sweden: : Centrum för folkhälsa 2007. http://dok.slso.sll.se/CES/FHG/Folkhalsoarbete/Halsa%20Stockholm/folkhalsorapport.2007.pdf (accessed 23 Aug 2021).

43 Leijon O, Wahlström J, Mulder M. Prevalence of Self-Reported Neck-Shoulder-Arm Pain and Concurrent Low Back Pain or Psychological Distress: Time-Trends in a General Population, 1990–2006. *Spine* 2009;**34**:1863–8. doi:10.1097/BRS.0b013e3181ab3397

For peer review only - http://bmjopen.bmj.com/site/about/guidelines.xhtml

For peer review only

44 Jonsson Å, Orwelius L, Dahlstrom U, *et al.* Evaluation of the usefulness of EQ-5D as a patient-reported outcome measure using the Paretian classification of health change among patients with chronic heart failure. *Journal of Patient-Reported Outcomes* 2020;**4**:50. doi:10.1186/s41687-020-00216-7

45 Devlin N, Herdman M, Pavesi M, *et al.* Health-related quality of life effects of enzalutamide in patients with metastatic castration-resistant prostate cancer: an in-depth post hoc analysis of EQ-5D data from the PREVAIL trial. *Health Qual Life Outcomes* 2017;**15**. doi:10.1186/s12955-017-0704-y

46 Pavesi M, Devlin N, Hakimi Z, *et al.* Understanding the effects on HR-QoL of treatment for overactive bladder: a detailed analysis of EQ-5D clinical trial data for mirabegron. *J Med Econ* 2013;**16**:866–76. doi:10.3111/13696998.2013.802240

47 McClure NS, Sayah FA, Ohinmaa A, *et al.* Minimally Important Difference of the EQ-5D-5L Index Score in Adults with Type 2 Diabetes. *Value Health* 2018;**21**:1090–7. doi:10.1016/j.jval.2018.02.007

48 Hakimi Z, Herdman M, Pavesi M, *et al.* Using EQ-5D-3L and OAB-5D to assess changes in the health-related quality of life of men with lower urinary tract symptoms associated with benign prostatic hyperplasia. *Qual Life Res* 2017;**26**:1187–95. doi:10.1007/s11136-016-1460-x

49 Bushnell DM, Martin ML, Ricci J-F, *et al.* Performance of the EQ-5D in patients with irritable bowel syndrome. *Value Health* 2006;**9**:90–7. doi:10.1111/j.1524-4733.2006.00086.x

50 Whynes DK. Correspondence between EQ-5D health state classifications and EQ VAS scores. *Health Qual Life Outcomes* 2008;**6**:94. doi:10.1186/1477-7525-6-94

51 McCaffrey N, Kaambwa B, Currow DC, *et al.* Health-related quality of life measured using the EQ-5D–5L: South Australian population norms. *Health Qual Life Outcomes* 2016;**14**:133.

52 Burström K, Johannesson M, Diderichsen F. Health-related quality of life by disease and socio-economic group in the general population in Sweden. *Health Policy* 2001;**55**:51–69.

53 Faxén UL, Hage C, Donal E, *et al.* Patient reported outcome in HFpEF: Sex-specific differences in quality of life and association with outcome. *International Journal of Cardiology* 2018;**267**:128–32. doi:10.1016/j.ijcard.2018.04.102

54 Janssen MF, Szende A, Cabases J, *et al.* Population norms for the EQ-5D-3L: a cross-country analysis of population surveys for 20 countries. *Eur J Health Econ* 2019;**20**:205–16. doi:10.1007/s10198-018-0955-5

55 Stavem K, Augestad LA, Kristiansen IS, *et al.* General population norms for the EQ-5D-3 L in Norway: comparison of postal and web surveys. *Health Qual Life Outcomes* 2018;**16**. doi:10.1186/s12955-018-1029-1

56 Teni FS, Burström K, Berg J, *et al.* Predictive ability of the American Society of Anaesthesiologists physical status classification system on health-related quality of life of patients after total hip replacement: comparisons across eight EQ-5D-3L value sets. *BMC Musculoskeletal Disorders* 2020;**21**:441. doi:10.1186/s12891-020-03399-8

Supplementary material 1: Supplementary tables

For peer review only - http://bmjopen.bmj.com/site/about/guidelines.xhtml

Tables and figures

Table 1: Demographic characteristics and Body Mass Index (BMI) in the 11 National Quality Registers (NQRs) and in the general population

Variable	Intervention-based registers							Diagnosis-based registers				General population
	Spine	Hip	Knee	Ankle	Cruciate ligament	BOA	Fracture	Heart failure	Respiratory failure	Psoriasis	Rheumatology	
	n=48,960	n=90,660	n=16,324	n=700	n=8,430	n=13,647	n=50,892	n=1,436	n=1,050	n=2,680	n=31,462	n=49,169
	%	%	%	%	%	%	%	%	%	%	%	%
**Age in years** [mean (SD)]	58.8 (15.3)	68.5 (10.3)	68.9 (8.7)	63.2 (11.7)	29.8 (10.0)	65.5 (9.1)	62.1 (18.1)	72.8 (11.1)	73.7 (7.7)	50.3 (14.8)	54.3 (15.7)	46.2 (15.3)
**Age group**												
<30	1,674 (3.4)	122 (0.1)	1 (0.0)	10 (1.4)	4,964 (58.9)	6 (0.0)	3,793 (7.5)	2 (0.1)	-	251 (9.4)	2,593 (8.2)	8,160 (16.6)
30-39	4,652 (9.5)	625 (0.7)	10 (0.1)	21 (3.0)	1,797 (21.3)	76 (0.6)	2,870 (5.6)	7 (0.5)	1 (0.1)	422 (15.7)	3,483 (11.1)	9,708 (19.7)
40-49	7,695(15.7)	3,394 (3.7)	278 (1.7)	52 (7.4)	1,299 (15.4)	624 (4.6)	4,651 (9.1)	37 (2.6)	1 (0.1)	581 (21.7)	5,353 (17.0)	9,964 (20.3)
50-59	8,964(18.3)	11,766 (13.0)	2,136 (13.1)	145 (20.7)	347 (4.1)	2,537(18.6)	8,216 (16.1)	137 (9.5)	41 (3.9)	616 (23.0)	6,721 (21.4)	10,491 (21.3)
60-69	11,835(24.2)	30,285 (33.4)	5,814 (35.6)	233 (33.3)	22 (0.3)	5,849 (42.9)	11,918 (23.4)	294 (20.5)	265 (25.2)	568 (21.2)	6,893(25.1)	7,602 (15.5)
70-79	10,999 (22.5)	31,869 (35.2)	6,296 (38.6)	215 (30.7)	1 (0.0)	3,836 (28.1)	10,799 (21.2)	512 (35.7)	494 (47.0)	201 (7.5)	4,448 (14.1)	2,978 (6.1)
80+	3,141 (6.4)	12,599 (13.9)	1,789 (11.0)	24 (3.4)	-	719 (5.3)	8,645 (17.0)	447 (31.1)	248 (23.6)	41 (1.5)	971 (3.1)	266 (0.5)
**Sex**												
Women	25,328 (51.7)	51,263 (56.5)	9,392 (57.5)	311 (44.4)	3,772 (44.7)	9,844 (72.1)	33,781 (66.4)	507(35.3)	623 (59.3)	994 (37.1)	20,668 (65.7)	27,700 (56.3)
**BMI***	n=45,343	n=69,206	n=15,937	n=447	n=4,331	n=13,366			n=868	n=2,620		n=22,094
Underweight	264 (0.6)	501 (0.7)	26 (0.2)	3 (0.7)	14 (0.3)	40 (0.3)	-	-	118 (13.6)	36 (1.4)	-	461 (2.1)
Normal weight	15,371 (33.9)	22,103 (31.9)	3,106 (19.5)	97 (21.7)	2,547 (58.8)	3,728 (27.9)	-	-	361 (41.6)	760 (29.0)	-	12,599 (57.0)
Overweight	19,823 (43.7)	30,164 (43.6)	7,240 (45.4)	200 (44.7)	1,466 (33.8)	5,875 (44.0)	-	-	220 (25.3)	999 (38.1)	-	6,837 (30.9)
Obesity class I	8,089 (17.8)	12,596 (18.2)	4,217 (26.5)	107 (23.9)	252 (5.8)	2,672 (20.0)	-	-	110 (12.7)	534 (20.4)	-	1,683 (7.6)
Obesity class II	1,549 (3.4)	3,200 (4.6)	1,138 (7.1)	32 (7.2)	39 (0.9)	801 (6.0)	-	-	46 (5.3)	202 (7.7)	-	366 (1.7)
Obesity class III	247 (0.5)	642 (0.9)	210 (1.3)	8 (1.8)	13 (0.3)	250 (1.9)	-	-	13 (1.5)	89 (3.4)	-	148 (0.7)
Missing (count)	3,617	21,454	387	253	4,099	281	-	-	182	60	-	27,075

BMI categories: Underweight: <18.5; Normal weight: 18.5–24.9; Overweight: 25.0–29.9; Obesity class I: 30.0–34.9; Obesity class II: 35.0–39.9; Obesity class III: ≥40

*Percentage based on non-missing values

For peer review only - http://bmjopen.bmj.com/site/about/guidelines.xhtml

Table 2: Prevalence of problems reported on EQ-5D-3L, at baseline and 1-year follow-up in the 11 National Quality Registers (NQRs) and in the general population

	Patient group in NQRs/	Severity level	EQ-5D-3L dimension									
			Baseline					1-year follow-up				
			Mobility	Self-care	Usual activities	Pain/ discomfort	Anxiety/ depression	Mobility	Self-care	Usual activities	Pain/ discomfort	Anxiety/ depression
			%	%	%	%	%	%	%	%	%	%
Intervention-based registers	**Spine** (n=48,960)	Level 1	7,397(15.1)	39,123 (**79.9**)	13,691 (28.0)	302 (0.6)	20,660 (42.2)	27,881 (**56.9**)	44,819 (**91.5**)	32,140 (**65.6**)	11,179 (22.8)	30,539 (**62.4**)
		Level 2	40,622 (**83.0**)	9,251 (18.9)	26,059 (**53.2**)	24,076 (49.2)	25,351 (**51.8**)	20,950 (42.8)	3,857 (7.9)	14,637 (29.9)	30,954 (**63.2**)	16,377 (33.4)
		Level 3	941 (1.9)	586 (1.2)	9,210 (18.8)	24,582 (**50.2**)	2,949 (6.0)	129 (0.3)	284 (0.6)	2,183 (4.5)	6,827 (13.9)	2,044 (4.2)
	**Hip** (n=90,660)	Level 1	7,340 (8.1)	70,483 (**77.7**)	36,364 (40.1)	1,400 (1.5)	54,120 (**59.7**)	55,970 (**61.7**)	83,800 (**92.4**)	70,683 (**78.0**)	41,270 (45.5)	71,102 (**78.4**)
		Level 2	83,026 (**91.6**)	19,307 (21.3)	45,371 (**50.0**)	52,519 (**57.9**)	33,597 (37.1)	34,565 (38.1)	6,319 (7.0)	18,161 (20.0)	45,422 (**50.1**)	18,188 (20.1)
		Level 3	294 (0.3)	870 (1.0)	8,925 (9.8)	36,741 (40.5)	2,943 (3.2)	125 (0.1)	541 (0.6)	1,816 (2.0)	3,968 (4.4)	1,370 (1.5)
	**Knee** (n=16,324)	Level 1	1,895 (11.6)	15,241 (**93.4**)	8,719 (**53.4**)	290 (1.8)	10,669 (**65.4**)	10,249 (**62.8**)	15,522 (**95.1**)	12,696 (**77.8**)	5,884 (36.0)	12,830 (**78.6**)
		Level 2	14,388 (**88.1**)	938 (5.7)	6,742 (41.3)	10,328 (**63.3**)	5,261 (32.2)	6,048 (37.0)	710 (4.3)	3,354 (20.5)	9,588 (**58.7**)	3,222 (19.7)
		Level 3	41 (0.3)	145 (0.9)	863 (5.3)	5,706 (35.0)	394 (2.4)	27 (0.2)	92 (0.6)	274 (1.7)	852 (5.2)	272 (1.7)
	**Ankle** (n=700)	Level 1	29 (4.1)	617 (**88.1**)	264 (37.7)	8 (1.1)	404 (**57.7**)	253 (36.1)	641 (**91.6**)	448 (**64.0**)	170 (24.3)	503 (**71.9**)
		Level 2	659 (**94.1**)	76 (10.9)	346 (**49.4**)	351 (**50.1**)	266 (38.0)	443 (**63.3**)	51 (7.3)	223 (31.9)	459 (**65.6**)	180 (25.7)
		Level 3	12 (1.7)	7 (1.0)	90 (12.9)	341 (48.7)	30 (4.3)	4 (0.6)	8 (1.1)	29 (4.1)	71 (10.1)	17 (2.4)
	**Cruciate ligament** (n=8,430)	Level 1	5,645 (**67.0**)	8,209 (**97.4**)	4,503 (**53.4**)	1,294 (15.3)	4,190 (**49.7**)	7,308 (**86.7**)	8,337 (**98.9**)	6,663 (**79.0**)	2,943 (34.9)	5,501 (**65.3**)
		Level 2	2,762 (32.8)	178 (2.1)	3,172 (37.6)	6,658 (**79.0**)	3,779 (44.8)	1,117 (13.3)	69 (0.8)	1,636 (19.4)	5,223 (**62.0**)	2,649 (31.4)
		Level 3	23 (0.3)	43 (0.5)	755 (9.0)	478 (5.7)	461 (5.5)	5 (0.1)	24 (0.3)	131 (1.6)	264 (3.1)	280 (3.3)
	**BOA** (n=13,647)	Level 1	5,781 (42.4)	13,120 (**96.1**)	10,274 (**75.3**)	351 (2.6)	8,981 (**65.8**)	7,096 (**52.0**)	13,093 (**95.9**)	10,880 (**79.7**)	1,264 (9.3)	9,553 (**70.0**)
		Level 2	7,854 (**57.6**)	470 (3.4)	3,200 (23.4)	11,755 (**86.1**)	4,473 (32.8)	6,542 (47.9)	494 (3.6)	2,646 (19.4)	11,278 (**82.6**)	3,930 (28.8)
		Level 3	12 (0.1)	57 (0.4)	173 (1.3)	1,541 (11.3)	193 (1.4)	9 (0.1)	60 (0.4)	121 (0.9)	1,105 (8.1)	164 (1.2)
	**Fracture** (n=50,892)	Level 1	41,022 (**80.6**)	45,512 (**89.4**)	42,073 (**82.7**)	32,363 (**63.6**)	40,462 (**79.5**)	35,314 (**69.4**)	44,852 (**88.1**)	38,161 (**75.0**)	20,558 (40.4)	38,181 (**75.0**)
		Level 2	9,542 (18.7)	4,373 (8.6)	5,815 (11.4)	16,658 (32.7)	9,419 (18.5)	14,970 (29.4)	4,575 (9.0)	9,864 (19.4)	28,059 (**55.1**)	11,566 (22.7)
		Level 3	328 (0.6)	1,007 (2.0)	3,004 (5.9)	1,871 (3.7)	1,011 (2.0)	608 (1.2)	1,465 (2.9)	2,867 (5.6)	2,275 (4.5)	1,145 (2.2)
Diagnosis-based registers	**Heart failure** (n=1,436)	Level 1	866 (**60.3**)	1,315 (**91.6**)	1,041 (**72.5**)	689 (**48.0**)	865 (**60.2**)	837 (**58.3**)	1,298 (**90.4**)	1,025 (**71.4**)	685 (**47.7**)	896 (**62.4**)
		Level 2	567(39.5)	116 (8.1)	355 (24.7)	679 (47.3)	521 (36.3)	593 (41.3)	126 (8.8)	371 (25.8)	655 (45.6)	499 (34.7)
		Level 3	3 (0.2)	5 (0.3)	40 (2.8)	68 (4.7)	50 (3.5)	6 (0.4)	12 (0.8)	40 (2.8)	96 (6.7)	41 (2.9)
	**Respiratory failure** (n=1,050)	Level 1	243 (23.1)	650 (**61.9**)	272 (25.9)	305 (29.0)	432 (41.1)	187 (17.8)	551 (**52.5**)	211 (20.1)	256 (24.4)	387 (36.9)
		Level 2	786 (**74.9**)	343 (32.7)	557 (**53.0**)	600 (**57.1**)	532 (**50.7**)	814 (**77.5**)	409 (39.0)	526 (**50.1**)	627 (**59.7**)	559 (**53.2**)
		Level 3	21 (2.0)	57 (5.4)	221 (21.0)	145 (13.8)	86 (8.2)	49 (4.7)	90 (8.6)	313 (29.8)	167 (15.9)	104 (9.9)
	**Psoriasis** (n=2,680)	Level 1	2,064 (**77.0**)	2,554 (**95.3**)	2,183 (**81.5**)	885 (33.0)	1,318 (**49.2**)	2,151 (**80.3**)	2,578 (**96.2**)	2,329 (86.**9**)	1,231 (45.9)	1,725 (**64.4**)
		Level 2	615 (22.9)	115 (4.3)	454 (16.9)	1,536 (**57.3**)	1,185 (44.2)	526 (19.6)	95 (3.5)	312 (11.6)	1,307(**48.8**)	847 (31.6)
		Level 3	1 (0.0)	11 (0.4)	43 (1.6)	259 (9.7)	177 (6.6)	3 (0.1)	7 (0.3)	39 (1.5)	142 (5.3)	108 (4.0)
	**Rheumatology** (n=31,462)	Level 1	15,883 (**50.5**)	25,394 (**80.7**)	18,304 (**58.2**)	3,891 (12.4)	16,917 (**53.8**)	17,585 (**55.9**)	26,523 (**84.3**)	20,443 (**65.0**)	5,373 (17.1)	18,408 (**58.5**)
		Level 2	15,506 (49.3)	5,718 (18.2)	11,645 (37.0)	22,703 (**72.2**)	13,014 (41.4)	13,776 (43.8)	4,591 (14.6)	9,873 (31.4)	22,206 (**70.6**)	11,553 (36.7)
		Level 3	73 (0.2)	350 (1.1)	1,513 (4.8)	4,868 (15.5)	1,531 (4.9)	101 (0.3)	348 (1.1)	1,146 (3.6)	3,883 (12.3)	1,501 (4.8)
**General population** (n=49,169)		Level 1	44,279 (**90.1**)	48,371 (**98.4**)	44,848 (**91.2**)	24,946 (**50.8**)	32,721 (**66.5**)	-	-	-	-	-
		Level 2	4,840 (9.8)	600 (1.2)	3,785 (7.7)	22,185 (45.1)	15,126 (30.8)	-	-	-	-	-
		Level 3	50 (0.1)	198 (0.4)	536 (1.1)	2038 (4.1)	1,322 (2.7)	-	-	-	-	-

The highest proportion in bold; Level 1: No problems; Level 2: Some/moderate problems; Level 3: confined to bed/unable to/extreme problems; Proportions rounded off to one decimal using MS Excel

Table 3: Mean (SD) EQ VAS score at baseline, 1-year follow-up and the change in the 9 National Quality Registers (NQRs) and in the general population

Time	Variable	Mean EQ VAS score (SD)									
		Intervention-based registers							Diagnosis-based registers		General population
		Spine	Hip	Knee	Ankle	Cruciate ligament	BOA	Fracture	Heart failure	Respiratory failure	
**Baseline**	**Overall**	47.8 (22.1)	56.2 (22.2)	64.6 (22.1)	55.8 (21.4)	62.8 (23.0)	68.3 (18.6)	84.7 (18.4)	65.4 (18.9)	51.1 (21.3)	79.5 (18.3)
	**Sex**										
	Men	50.4 (22.1)	59.6 (21.3)	68.1 (20.9)	58.5 (20.5)	63.9 (23.0)	70.8 (17.4)	86.1 (17.0)	66.5 (18.9)	50.9 (20.2)	80.4 (17.4)
	Women	45.2 (21.8)	53.7 (22.5)	62.0 (22.5)	52.5 (22.2)	61.4 (23.0)	67.3 (18.9)	84.0 (19.0)	62.9 (18.7)	51.2 (22.0)	78.8 (18.9)
	**Age group**										
	<30	46.6 (21.3)	46.2 (24.3)	-	49.9 (17.5)	63.3 (23.1)	71.3 (2.5)	90.3 (13.8)	-	-	81.2 (16.8)
	30-39	45.1 (22.0)	52.2 (22.3)	58.9 (25.6)	45.6 (18.1)	63.4 (22.4)	62.6 (22.2)	88.8 (14.7)	74.0 (20.7)	-	80.9 (17.1)
	40-49	45.9 (21.7)	54.3 (22.5)	56.7 (23.3)	51.4 (21.3)	60.7 (23.5)	63.9 (19.6)	88.3 (15.6)	67.2 (16.6)	-	79.4 (18,3)
	50-59	46.8 (22.3)	55.0 (22.6)	60.6 (23.4)	55.0 (20.7)	60.7 (22.8)	66.1 (19.5)	87.6 (16.3)	62.1 (19.7)	47.4 (27.8)	78.5 (19.2)
	60-69	48.7 (22.2)	56.8 (22.6)	64.5 (22.5)	56.4 (21.8)	59.0 (28.0)	69.3 (18.3)	87.4 (16.4)	69.5 (19.8)	50.0 (21.2)	78.5 (19.4)
	70-79	49.9 (22.2)	57.1 (22.7)	66.3 (21.3)	58.7 (22.0)	-	69.3 (18.0)	83.3 (18.6)	65.2 (19.4)	50.7 (20.0)	76.1 (19.3)
	80+	49.6 (21.7)	54.8 (21.4)	64.8 (20.7)	50.3 (17.6)	-	66.6 (18.1)	71.2 (22.1)	63.2 (16.8)	53.8 (22.5)	71.1 22.3()
	**BMI**										
	Underweight	42.6 (22.0)	53.6 (22.1)	62.3 (24.9)	40.7 (29.9)	72.6 (20.6)	64.8 (20.4)	-	-	48.9 (21.6)	78.2 (19.1)
	Normal weight	48.1 (22.2)	57.9 (22.2)	66.7 (21.9)	56.1 (23.3)	64.8 (23.0)	71.1 (17.9)	-	-	52.5 (21.9)	82.1 (16.1)
	Overweight	48.6 (22.0)	57.6 (21.9)	66.3 (21.7)	57.4 (19.8)	63.7 (22.5)	69.2 (18.2)	-	-	52.0 (19.1)	78.7 (17.9)
	Obesity class I	46.8 (21.7)	54.3 (22.1)	62.4 (21.9)	54.5 (20.9)	59.2 (23.3)	65.1 (19.2)	-	-	51.4 (21.2)	72.9 (20.3)
	Obesity class II	43.8 (22.3)	51.6 (22.8)	58.7 (23.1)	53.0 (19.9)	51.4 (24.2)	63.2 (18.8)	-	-	54.1 (19.2)	68.4 (21.1)
	Obesity class III	44.0 (21.8)	49.4 (23.6)	57.5 (23.5)	40.5 (14.5)	66.2 (20.2)	56.7 (19.3)	-	-	55.6 (17.3)	67.5 (21.9)
**1-year follow-up**	**Overall**	67.9 (22.5)	76.7 (20.0)	76.2 (19.4)	70.2 (18.9)	74.5 (19.9)	70.4 (18.7)	77.7 (20.4)	67.9 (18.8)	49.1 (20.4)	-
	**Sex**										-
	Men	69.4 (22.0)	78.6 (19.0)	77.8 (18.8)	72.3 (17.1)	75.3 (20.1)	70.8 (18.3)	78.7 (19.8)	68.4 (19.0)	49.4 (19.9)	-
	Women	66.5 (22.9)	75.3 (20.7)	75.1 (19.8)	67.6 (20.5)	73.6 (19.5)	70.3 (18.9)	77.2 (20.8)	65.4 (18.2)	48.9 (20.7)	-
	**Age group**										-
	<30	73.2 (20.3)	74.2 (20.1)	-	62.5 (23.7)	74.5 (19.8)	69.5 (28.2)	82.5 (17.1)	-	-	-
	30-39	70.9 (21.7)	77.4 (20.4)	75.5 (20.6)	64.4 (20.4)	74.5 (19.5)	71.7 (20.9)	82.0 (17.4)	76.0 (19.5)	-	-
	40-49	70.5 (22.6)	78.8 (19.5)	73.3 (20.7)	67.3 (17.4)	74.8 (20.4)	69.8 (19.1)	81.2 (17.7)	71.7 (17.4)	-	-
	50-59	68.3 (23.1)	78.5 (19.8)	73.6 (20.8)	69.4 (20.2)	73.4 (21.1)	69.5 (19.7)	80.7 (18.6)	70.5 (18.1)	48.8 (22.7)	-
	60-69	68.5 (22.3)	79.1 (19.4)	78.0 (18.6)	72.6 (17.2)	79.7 (19.1)	71.9 (18.1)	81.3 (18.3)	72.1 (17.8)	47.7 (20.5)	-
	70-79	64.8 (22.3)	75.8 (20.1)	76.5 (19.4)	71.0 (18.6)	-	69.9 (18.5)	76.1 (21.0)	66.6 (18.4)	49.1 (19.6)	-
	80+	60.7 (21.5)	71.1 (20.5)	73.1 (19.6)	58.6 (23.0)	-	64.9 (19.3)	63.1 (23.0)	63.0 (19.5)	50.3 (21.4)	-
	**BMI**										-
	Underweight	66.2 (24.0)	72.7 (22.0)	70.3 (16.9)	51.7 (16.1)	70.8 (19.8)	69.4 (14.7)	-	-	44.9 (20.7)	-
	Normal weight	70.3 (22.2)	78.6 (19.6)	77.9 (18.9)	68.9 (19.2)	75.6 (20.0)	73.9 (17.6)	-	-	49.9 (20.5)	-
	Overweight	68.6 (22.1)	77.7 (19.4)	77.6 (18.9)	72.4 (17.6)	74.6 (20.5)	70.9 (18.4)	-	-	50.9 (20.2)	-
	Obesity class I	64.5 (22.9)	74.5 (20.6)	74.4 (19.7)	67.3 (18.4)	72.1 (20.9)	66.9 (19.3)	-	-	48.5 (20.8)	-
	Obesity class II	60.3 (23.3)	70.9 (21.6)	72.7 (20.2)	69.1 (22.8)	64.8 (25.6)	65.7 (18.9)	-	-	47.2 (16.0)	-
	Obesity class III	61.4 (24.8)	68.1 (23.0)	66.7 (22.6)	39.4 (18.6)	61.8 (27.2)	58.6 (21.1)	-	-	54.3 (15.1)	-

For peer review only - http://bmjopen.bmj.com/site/about/guidelines.xhtml

**Change**	**Overall**	20.1 (27.1)	20.5 (25.8)	11.7 (24.6)	14.4 (24.7)	11.8 (26.4)	2.1 (18.7)	-7.0 (19.4)	2.1 (18.3)	-2.0 (24.1)	-
	**Sex**										-
	Men	19.0 (26.7)	19.0 (24.5)	9.7 (23.5)	13.8 (22.5)	11.4 (26.4)	0.0 (17.3)	-7.4 (18.9)	1.9 (18.3)	-1.4 (24.1)	-
	Women	21.2 (26.4)	21.6 (26.7)	13.1 (25.2)	15.1 (27.2)	12.2 (26.3)	3.0 (19.2)	-6.8 (19.6)	2.5 (18.2)	-2.3 (24.2)	-
	**Age group**										-
	<30	26.6 (26.3)	28.0 (29.0)	-	12.6 (32.3)	11.3 (26.6)	-1.8 (27.5)	-7.8 (19.5)	-	-	-
	30-39	25.9 (27.3)	25.2 (25.4)	16.6 (21.3)	18.8 (17.8)	11.2 (24.6)	9.0 (17.9)	-6.8 (18.3)	2.0 (11.0)	-	-
	40-49	24.6 (27.4)	24.5 (26.0)	16.6 (27.4)	15.9 (25.7)	14.1 (27.2)	5.9 (19.0)	-7.1 (18.4)	4.5 (19.2)	-	-
	50-59	21.4 (27.2)	23.5 (25.9)	13.0 (26.0)	14.4 (25.1)	12.7 (27.9)	3.4 (19.2)	-6.9 (18.8)	8.4 (19.1)	1.4 (26.1)	-
	60-69	19.8 (26.7)	22.3 (25.8)	13.6 (24.6)	16.2 (25.0)	20.6 (31.4)	2.6 (18.6)	-6.1 (18.2)	2.6 (17.5)	-2.3 (22.9)	-
	70-79	15.0 (26.1)	18.7 (25.5)	10.2 (23.9)	12.3 (24.3)	-	0.6 (18.4)	-7.2 (19.7)	1.4 (19.2)	-1.6 (23.9)	-
	80+	11.1 (25.8)	16.3 (25.6)	8.4 (23.8)	8.3 (23.3)	-	-1.7 (17.8)	-8.1 (22.3)	-0.3 (16.8)	-3.5 (25.8)	-
	**BMI**										-
	Underweight	23.6 (29.0)	19.1 (26.1)	8.0 (24.2)	11.0 (13.9)	-1.7 (22.0)	4.6 (20.9)	-	-	-4.0 (23.5)	-
	Normal weight	22.2 (27.4)	20.8 (25.5)	11.2 (23.5)	12.8 (25.9)	10.8 (26.6)	2.8 (17.6)	-	-	-2.6 (24.2)	-
	Overweight	20.0 (26.7)	20.1 (25.3)	11.3 (24.1)	14.9 (23.1)	10.9 (27.2)	1.8 (18.5)	-	-	-1.1 (23.0)	-
	Obesity class I	17.7 (27.2)	20.2 (26.4)	12.0 (25.2)	12.8 (23.9)	12.9 (27.1)	1.8 (20.0)	-	-	-2.9 (24.6)	-
	Obesity class II	16.5 (26.9)	19.3 (27.4)	14.0 (26.7)	16.1 (34.0)	13.4 (28.5)	2.5 (20.3)	-	-	-6.9 (21.0)	-
	Obesity class III	17.3 (28.7)	18.7 (28.6)	9.2 (28.5)	-1.1 (17.0)	-4.3 (21.1)	1.9 (18.6)	-	-	-1.3 (13.6)	-

BMI categories: Underweight: <18.5; Normal weight: 18.5–24.9; Overweight: 25.0–29.9; Obesity class I: 30.0–34.9; Obesity class II: 35.0–39.9; Obesity class III: ≥40

For peer review only

Table 4: Analysis of covariance testing difference in mean EQ VAS value by sex adjusted for age at baseline, 1-year and change in the 9 NQRS and in the general population

Time	Variable	Adjusted mean EQ VAS value (95% CI)									
		Intervention-based registers						Diagnosis-based registers			General population
		Spine	Hip	Knee	Ankle	Cruciate ligament	BOA	Fracture	Heart failure	Respiratory failure	
**Baseline**	**Sex**										
	Men	50.5 (50.2-50.8)	59.7 (59.5-59.9)	68.1 (67.6-68.6)	58.4 (56.2-60.6)	63.9 (63.2-64.6)	70.7 (69.9-71.5)	84.7 (84.4-85.0)	66.5 (65.1-67.9)	50.8 (48.3-53.3)	80.6 (80.3-80.8)
	Women	45.2 (44.9-45.5)	53.6 (53.4-53.8)	62.0 (61.5-62.4)	52.6 (50.2-55.0)	61.4 (60.6-62.1)	67.3 (66.8-67.8)	84.7 (84.5-84.9)	63.0 (60.9-65.0)	51.2 (49.2-53.2)	78.7 (78.4-78.9)
**1-year follow-up**	**Sex**										
	Men	69.3 (69.0-69.6)	78.3 (78.1-78.5)	77.8 (74.7-75.5)	72.3 (70.4-74.2)	75.3 (74.8-75.9)	70.9 (69.7-70.8)	77.2 (76.9-77.5)	68.2 (66.8-69.5)	49.4 (47.0-51.8)	-
	Women	66.5 (66.2-66.8)	75.5 (75.3-75.7)	75.1 (77.4-78.3)	67.7 (65.5-69.8)	73.6 (72.9-74.2)	70.3 (70.1-71.7)	78.0 (77.7-78.2)	65.9 (63.9-67.9)	48.9 (47.0-50.8)	-
**Change**	**Sex**										
	Men	18.7 (21.0-21.7)	18.6 (18.4-18.9)	9.7 (9.1-10.3)	13.9 (12.2-17.9)	11.4 (10.7-12.2)	0.2 (-0.7-1.0)	-7.5 (-7.8--7.2)	1.7 (0.3-3.0)	-1.4 (-4.2-1.4)	-
	Women	21.3 (18.5-19.2)	21.9 (21.6-22.1)	13.1 (12.6-13.6)	15.1 (11.4-16.4)	12.2 (11.3-13.0)	2.9 (2.4-3.5)	-6.8 (-7.0--6.5)	2.9 (1.0-4.9)	-2.4 (-4.6--0.1)	-

For peer review only

For peer review only - http://bmjopen.bmj.com/site/about/guidelines.xhtml

Figure 1: Proportion of patients by Paretian Classification of Health Change (PCHC), by diagnosis in the 11 National Quality Registers (NQRs)

Figure 2: Mean EQ VAS score by diagnosis at baseline and 1-year follow-up in the 9 National Quality Registers (NQRs) and the general population

Figure 3: Two-level random intercept model estimates of EQ VAS score by diagnosis adjusted for age and sex at baseline and 1-year in the 9 National Quality Registers (NQRs) [reference group: general population]

Figure 4: Mean EQ VAS score by Paretian Classification of Health Change (PCHC) by diagnosis in the 9 National Quality Registers (NQRs)

For peer review only

For peer review only - http://bmjopen.bmj.com/site/about/guidelines.xhtml

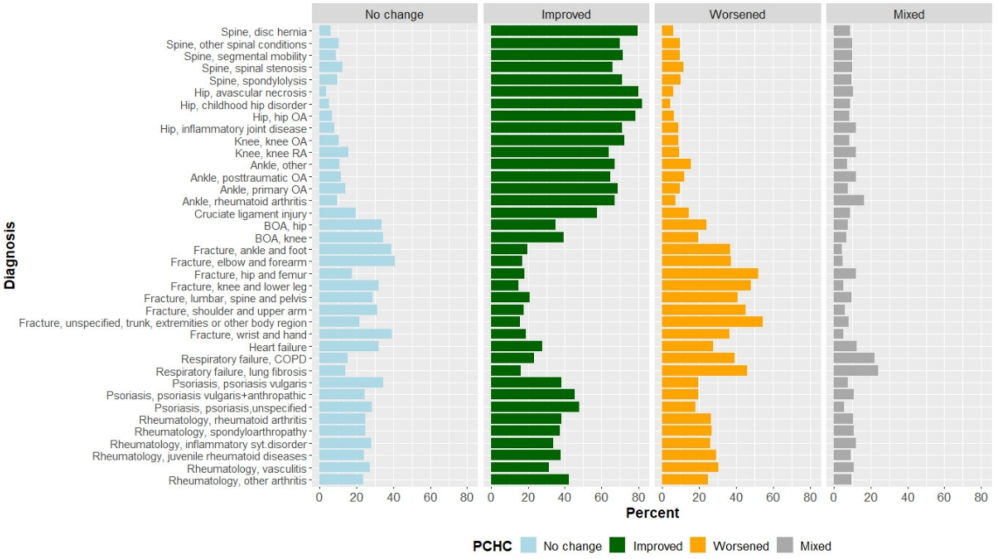

Figure 1: Proportion of patients by Paretian Classification of Health Change (PCHC), by diagnosis in the 11 National Quality Registers (NQRs)

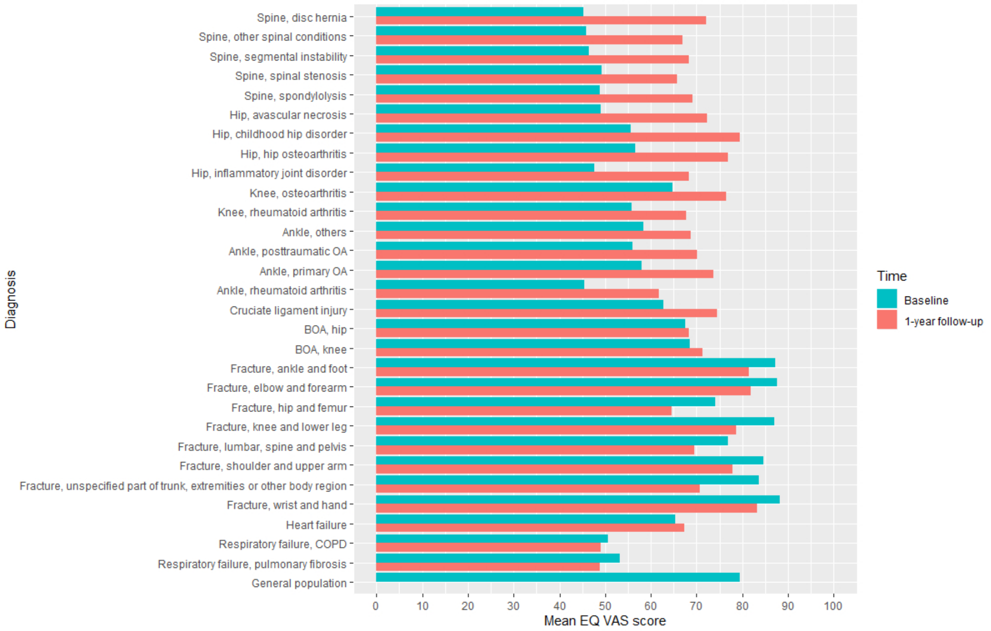

Figure 2: Mean EQ VAS score by diagnosis at baseline and 1-year follow-up in the 9 National Quality Registers (NQRs) and the general population

735x467mm (38 x 38 DPI)

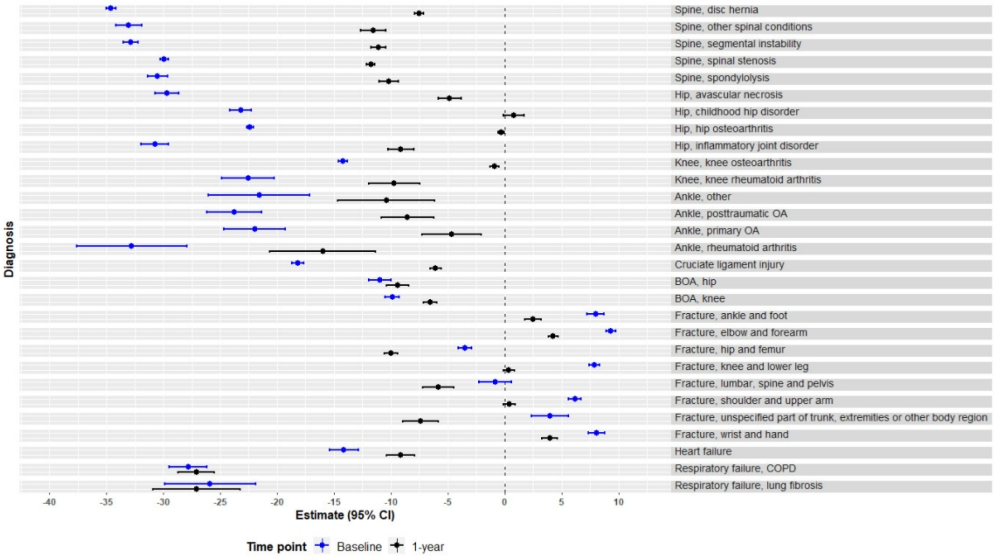

Figure 3: Two-level random intercept model estimates of EQ VAS score by diagnosis adjusted for age and sex at baseline and 1-year in the 9 National Quality Registers (NQRs) [reference group: general population]

928x525mm (37 x 37 DPI)

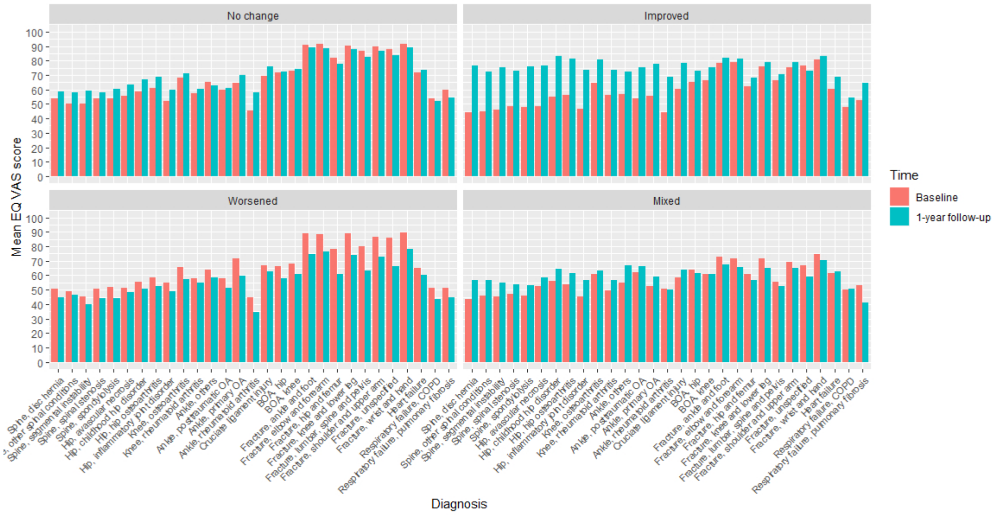

Figure 4: Mean EQ VAS score by Paretian Classification of Health Change (PCHC) by diagnosis in the 9 National Quality Registers (NQRs)

774x401mm (38 x 38 DPI)

Table S1: Sampling procedure followed in combining the data sets from each of the 11 National Quality Registers (NQRs), baseline to 1-year follow-up and the general population data

Category	Patient groups in NQRs	A	B	C	D	E
		n (%)	n (%)	n (%)	n (%)	n (%)
Intervention-based registers	Spine	53,418 (15.8)	53,015 (15.9)	51,047 (15.7)	48,960 (15.5)	44,196 (17.6)
	Hip	95,887 (22.3)	93,684 (28.0)	90,669 (27.9)	90,660 (28.7)	90,658 (36.1)
	Knee	17,600 (5.2)	16,324 (4.9)	16,324 (5.0)	16,324 (5.2)	16,324 (6.5)
	Ankle	794 (0.2)	789 (0.2)	725 (0.2)	700 (0.2)	668 (0.3)
	Cruciate ligament	10,532 (3.1)	10,532 (3.2)	10,465 (3.2)	8,430 (2.7)	8,155 (3.2)
	Osteoarthritis (BOA)	15,238 (4.5)	14,877 (4.5)	13,965 (4.3)	13,647 (4.3)	6,690 (2.7)
	Fracture	55,459 (16.4)	55,428 (16.6)	54,108 (16.6)	50,892 (16.1)	40,787 (16.2)
Diagnosis-based registers	Heart failure	1,789 (0.5)	1,746 (0.5)	1,691 (0.5)	1,436 (0.5)	1,044 (0.4)
	Respiratory failure	1,287 (0.4)	1,063 (0.3)	1,050 (0.3)	1,050 (0.3)	725 (0.3)
	Psoriasis	3,041 (0.9)	2,878 (0.9)	2,789 (0.9)	2,680 (0.8)	-
	Rheumatology	34,036 ()	33,686 (10.1)	32,565 (10.0)	31,462 (10.0)	-
NQRs (total, count)		289,081 (85.4)	284,022 (85.0)	275,398 (84.6)	266,241 (84.4	209,247 (83.4)
General population data		49,499 (14.6)	49,999 (15.0)	49,999 (15.4)	49,169 (15.6	41,761 (16.6)
Total		338,580 (100.0)	334,021 (100.0)	325,397 (100.0)	315,410 (100.0)	251,008 (100.0)

A= Data on baseline and 1-year follow-up
B= After selection of data with information on diagnosis and intervention
C= After de-duplication of records occurring in more than one patient group (duplicate records taken out keeping one record)
D= Included in the analyses after exclusion of missing values on age at baseline, or on any of the EQ-5D dimensions
E= Included in the analyses of EQ VAS values
BOA=Better management of osteoarthritis register, referred to as BOA in whole manuscript
Proportions rounded off to one decimal using MS Excel

For peer review only

Table S2: Demographic characteristics of patients across diagnoses in the 11 National Quality Registers (NQRs)

Patient group in NQRs		Diagnosis	Age (years) Mean (SD)	Age group							Sex
				<30	30-39	40-49	50-59	60-69	70-79	80+	Women
				n (%)	n (%)	n (%)	n (%)	n (%)	n (%)	n (%)	n (%)
Intervention-based registers	Spine	Disc hernia (n=12,582)	46.1 (13.5)	1,216 (9.7)	3,089 (24.6)	3,708 (29.5)	2,312 (18.4)	1,519 (12.1)	624 (5.0)	114 (0.9)	5,784 (46.0)
		Other spinal conditions (n=1,431)	58.2 (13.1)	32 (2.2)	98 (6.8)	207 (14.5)	382 (26.7)	411 (28.7)	260 (18.2)	41 (2.9)	891 (62.3)
		Segmental instability (n=4,290)	45.6 (10.2)	245 (5.7)	932 (21.7)	1,674 (39.0)	1,034 (24.1)	345 (8.0)	59 (1.4)	1 (0.0)	2,326 (54.2)
		Spinal stenosis (n=28,194)	67.2 (10.5)	56 (0.2)	245 (0.9)	1,390 (4.9)	4,518 (16.0)	9,101 (32.3)	9,914 (35.2)	2,970 (10.5)	15,046 (53.4)
		Spondylolysis (n=2,463)	50.9 (12.5)	125 (5.1)	288 (11.7)	716 (29.1)	718 (29.2)	459 (18.6)	142 (5.8)	15 (0.6)	1,281 (52.0)
	Hip	Avascular necrosis (n=1,594)	66.0 (13.6)	13 (0.8)	68 (4.3)	127 (8.0)	228 (14.3)	444 (27.9)	458 (28.7)	256 (16.1)	935 (58.7)
		Childhood hip disorder (n=1,846)	54.5 (12.4)	23 (1.2)	169 (9.2)	492 (26.7)	533 (28.9)	390 (21.1)	199 (10.8)	40 (2.2)	1,139 (61.7)
		Hip osteoarthritis (n=86,023)	68.9 (9.9)	61 (0.1)	348 (0.4)	2,706 (3.1)	10,814 (12.6)	29,020 (33.7)	30,868 (35.9)	12,206 (14.2)	48,329 (56.2)
		Inflammatory joint disorder (n=1,197)	64.5 (12.5)	25 (2.1)	40 (3.3)	69 (5.8)	191 (16.0)	431 (36.0)	344 (28.7)	97 (8.1)	860 (71.8)
	Knee	Knee osteoarthritis (n=16,020)	69.0 (8.7)	0 (0.0)	9 (0.1)	262 (1.6)	2085 (13.0)	5,695 (35.5)	6,203 (38.7)	1,766 (11.0)	9,173 (57.3)
		Knee rheumatoid arthritis (n=304)	66.2 (10.2)	1 (0.3)	1 (0.3)	16 (5.3)	51 (16.8)	119 (39.1)	93 (30.6)	23 (7.6)	219 (72.0)
	Ankle	Others (n=85)	57.7 (12.6)	1 (1.2)	7 (8.2)	14 (16.5)	22 (25.9)	26 (30.6)	14 (16.5)	1 (1.2)	31 (36.5)
		Posttraumatic OA (n=308)	62.0 (12.1)	7 (2.3)	10 (3.2)	24 (7.8)	76 (24.7)	98 (31.8)	83 (26.9)	10 (3.2)	134 (43.5)
		Primary OA (n=234)	67.1 (8.9)	0 (0.0)	1 (0.4)	6 (2.6)	37 (15.8)	85 (36.3)	92 (39.3)	13 (5.6)	86 (36.8)
		Rheumatoid arthritis (n=73)	62.1 (13.4)	2 (2.7)	3 (4.1)	8 (11.0)	10 (13.7)	24 (32.9)	26 (35.6)	0 (0.0)	60 (82.2)
	Cruciate ligament	Cruciate ligament injury (n=8,430)	29.8 (10.0)	4,964 (58.9)	1,797 (21.3)	1,299 (15.4)	347 (4.1)	22 (0.3)	1 (0.0)	0 (0.0)	3,772 (44.7)
	BOA	BOA hip (n=3,440)	65.9 (9.2)	1 (0.0)	27 (0.8)	158 (4.6)	552 (16.0)	1,484 (43.1)	1,043 (30.3)	175 (5.1)	2474 (71.9)
		BOA knee (n=10.207)	65.4 (9.1)	5 (0.0)	49 (0.5)	466 (4.6)	1,985 (19.4)	4,365 (42.8)	2,793 (27.4)	544 (5.3)	7370 (72.2)
	Fracture	Ankle and foot (n=3,728)	51.7 (17.4)	530 (14.3)	424 (11.4)	605 (16.3)	819 (22.0)	738 (19.8)	439 (11.8)	163 (4.4)	2,208 (59.4)
		Elbow and forearm (n=13,996)	61.6 (15.9)	794 (5.7)	708 (5.1)	1,189 (8.5)	2,678 (19.1)	4,071 (29.1)	2,999 (21.4)	1,557 (11.1)	10942 (78.2)
		Hip and femur (n=8,378)	77.2 (12.0)	51 (0.6)	58 (0.7)	116 (1.4)	377 (4.5)	1,233 (14.7)	2,442 (29.1)	4,101 (48.9)	5655 (67.5)
		Knee and lower leg (n=10,866)	57.4 (17.0)	960 (8.8)	804 (7.4)	1,377 (12.7)	2,180 (20.1)	2,774 (25.5)	1,962 (18.1)	809 (7.4)	6714 (61.8)
		Lumbar, spine and pelvis (n=1,128)	72.2 (16.8)	36 (3.2)	31 (2.7)	44 (3.9)	111 (9.8)	153 (13.6)	299 (26.5)	454 (40.2)	780 (69.1)
		Shoulder and upper arm (n=7,835)	63.6 (16.6)	464 (5.9)	317 (4.0)	621 (7.9)	1,195 (15.3)	2,064 (26.3)	1,946 (24.8)	1,228 (15.7)	5,291(67.5)
		Unspecified part of trunk, extremities or other body region (n=749)	61.3 (19.2)	76 (10.1)	43 (5.7)	74 (9.9)	106 (14.2)	142 (19.0)	189 (25.2)	119 (15.9)	327 (43.7)
		Wrist and hand (n=4,222)	49.9 (19.2)	882 (20.9)	485 (11.5)	625 (14.8)	750 (17.8)	743 (17.6)	523 (12.4)	214 (5.1)	1,864 (44.1)

For peer review only

Diagnosis-based registers	**Heart failure**	Heart failure (n=1,436)	72.8 (11.1)	2 (0.5)	7 (2.6)	37 (9.5)	137 (20.5)	294 (35.7)	512 (31.1)	447 (0.5)	507 (35.3)
	**Respiratory failure**	COPD (n=900)	73.3 (7.6)	0 (0.0)	0 (0.0)	1 (0.1)	39 (4.3)	237 (26.3)	425 (47.2)	198 (22.0)	555 (61.7)
		Lung fibrosis (n=150)	75.9 (7.7)	0 (0.7)	1 (1.3)	(0.0)	2 (18.7)	28 (46.0)	69 (33.3)	50 (0.7)	68 (45.3)
	**Psoriasis**	Psoriasis vulgaris (n=1,704)	49.4 (15.2)	191 (11.2)	273 (16.0)	370 (21.7)	374 (21.9)	346 (20.3)	123 (7.2)	27 (1.6)	576 (33.8)
		Psoriasis vulgaris + anthropathic (n=537)	51.8 (13.2)	21 (3.9)	82 (15.3)	131 (24.4)	132 (24.6)	127 (23.6)	37 (6.9)	7 (1.3)	237 (44.1)
		Psoriasis unspecified (n=439)	51.6 (14.9)	39 (8.9)	67 (15.3)	80 (18.2)	110 (25.1)	95 (21.6)	41 (9.3)	7 (1.6)	181 (41.2)
	**Rheumatol-ogy**	Rheumatoid arthritis (n=17,870)	59.2 (13.9)	585 (3.3)	1,233 (6.9)	2,362 (13.2)	3,846 (21.5)	5,604 (31.4)	3,430 (19.2)	810 (4.5)	12,954 (72.5)
		Spondyloarthropathy (n=8,782)	47.6 (13.9)	1,009 (11.5)	1,596 (18.2)	2,228 (25.4)	2,058 (23.4)	1,380 (15.7)	448 (5.1)	63 (0.7)	4,271 (48.6)
		Inflammatory systemic disorder (n=1,614)	51.9 (15.8)	155 (9.6)	243 (15.1)	295 (18.3)	337 (20.9)	349 (21.6)	206 (12.8)	29 (1.8)	1,249 (77.4)
		Juvenile rheumatoid diseases (n=1,088)	29.7 (12.8)	676 (62.1)	186 (17.1)	134 (12.3)	43 (4.5)	33 (3.0)	9 (0.8)	1 (0.1)	865 (79.5)
		Vasculitis (n=410)	60.0 (14.0)	22 (5.4)	18 (4.4)	40 (9.8)	66 (16.1)	149 (36.3)	105 (25.6)	10 (2.4)	234 (57.1)
		Other arthritis (n=1,698)	54.0 (15.9)	146 (8.6)	207 (12.2)	294 (17.3)	365 (21.5)	378 (22.3)	250 (14.7)	58 (3.4)	1,095 (64.5)

*Percent based on non-missing values;
Proportions rounded off to one decimal using MS Excel

For peer review only

Table S3: Prevalence of reported problems on EQ-5D-3L by diagnosis, at baseline and 1-year follow-up in the 11 National Quality Registers (NQRs) and in the general population

	Patient group in NQRs	Diagnosis	Severity level	Baseline EQ-5D-3L dimension					1-year follow-up EQ-5D-3L dimension				
				Mobility	Self-care	Usual activities	Pain/ discomfort	Anxiety/ depression	Mobility	Self-care	Usual activities	Pain/ discomfort	Anxiety/ Depression
				n (%)	n (%)	n (%)	n (%)	n (%)	n (%)	n (%)	n (%)	n (%)	n (%)
Intervention-based registers	Spine	Disc hernia (n=12,582)	Level 1	1,996 (15.9)	8,535 (**67.8**)	2,353 (18.7)	89 (0.7)	4,603 (36.6)	9,258 (**73.6**)	11,659 (**92.7**)	9,015 (**71.6**)	3,596 (28.6)	8,144 (**64.7**)
			Level 2	9,931 (**78.9**)	3,788 (30.1)	6,373 (**50.7**)	5,470 (43.5)	6,954 (**55.3**)	3,293 (26.2)	864 (6.9)	3,154 (25.1)	7,799 (**62.0**)	3,903 (31.0)
			Level 3	655 (5.2)	259 (2.1)	3,856 (30.6)	7,023 (**55.8**)	1,025 (8.1)	31 (0.2)	59 (0.5)	413 (3.3)	1,187 (9.4)	535 (4.3)
		Other spinal conditions (n=1,431)	Level 1	279 (19.5)	1,144 (**79.9**)	414 (28.9)	14 (1.0)	586 (41.0)	813 (**56.8**)	1,311 (**91.6**)	905 (**63.2**)	328 (22.9)	893 (**62.4**)
			Level 2	1,113 (**77.8**)	272 (19.0)	689 (**48.1**)	610 (42.6)	742 (**51.9**)	612 (42.8)	111 (7.8)	451 (31.5)	869 (**60.7**)	472 (33.0)
			Level 3	39 (2.7)	15 (1.0)	328 (22.9)	807 **56.4**)	103 (7.2)	6 (0.4)	9 (0.6)	75 (5.2)	234 (16.4)	66 (4.6)
		Segmental instability (n=4,290)	Level 1	1,419 (33.1)	3,277 (**76.4**)	900 (21.0)	21 (0.5)	1,514 (35.3)	3,084 (**71.9**)	3,902 (**91.0**)	2,641 (**61.6**)	977 (22.8)	2,525 (**58.9**)
			Level 2	2,843 (**66.3**)	984 (22.9)	2,463 (**57.4**)	2,160 (**50.3**)	2,462 (**57.4**)	1,192 (27.8)	371 (8.6)	1,364 (31.8)	2,663 (**62.1**)	1,537 (35.8)
			Level 3	28 (0.7)	29 (0.7)	927 (21.6)	2,109 (49.2)	314 (7.3)	14 (0.3)	17 (0.4)	285 (6.6)	650 (15.2)	228 (5.3)
		Spinal stenosis (n=28,194)	Level 1	3,028 (10.7)	24,047 (**85.3**)	9321 (33.1)	162 (0.6)	12,977 (46.0)	13,069 (46.4)	25,673 (**91.1**)	17,978 (**63.8**)	5,697 (20.2)	17,464 (**61.9**)
			Level 2	24,966 (**88.6**)	3,877 (13.8)	15,161 (**53.8**)	14,520 (**51.5**)	13,874 (**49.2**)	15,049 (**53.4**)	2,330 (8.3)	8,926 (31.7)	18,071 (**64.1**)	9,650 (34.2)
			Level 3	200 (0.7)	270 (1.0)	3,712 (13.2)	13,512 (47.9)	1,343 (4.8)	76 (0.3)	191 (0.7)	1,290 (4.6)	4426 (15.7)	1,080 (3.8)
		Spondylolysis (n=2,463)	Level 1	675 (27.4)	2,120 (**86.1**)	703 (28.5)	16 (0.6)	980 (39.8)	1,657 (**67.3**)	2,274 (**92.3**)	1,601 (**65.0**)	581 (23.6)	1,513 (**61.4**)
			Level 2	1,769 (**71.8**)	330 (13.4)	1,373 (**55.7**)	1,316 (**53.4**)	1,319 (**53.6**)	804 (32.6)	181 (7.3)	742 (30.1)	1,552 (**63.0**)	815 (33.1)
			Level 3	19 (0.8)	13 (0.5)	387 (15.7)	1,131 (45.9)	164 (6.7)	2 (0.1)	8 (0.3)	120 (4.9)	330 (13.4)	135 (5.5)
	Hip	Avascular necrosis (n=594)	Level 1	79 (5.0)	985 (**61.8**)	358 (22.5)	30 (1.9)	741 (**46.5**)	857 (**53.8**)	1,383 (**86.8**)	1,085 (**68.1**)	624 (39.1)	1,076 (**67.5**)
			Level 2	1,476 (**92.6**)	570 (35.8)	838 (**52.6**)	592 (37.1)	716 (44.9)	732 (45.9)	198 (12.4)	434 (27.2)	869 (**54.5**)	477 (29.9)
			Level 3	39 (2.4)	39 (2.4)	398 (25.0)	972 (**61.0**)	137 (8.6)	5 (0.3)	13 (0.8)	75 (4.7)	101 (6.3)	41 (2.6)
		Childhood hip disorder (n=1,846)	Level 1	127 (6.9)	1,390 (**75.3**)	653 (35.4)	22 (1.2)	1,014 (**54.9**)	1,278 (**69.2**)	1,672 (**90.6**)	1,449 (**78.5**)	989 (**53.6**)	1,463 (**79.3**)
			Level 2	1,707 (**92.5**)	438 (23.7)	976 (**52.9**)	998 (**54.1**)	758 (41.1)	568 (30.8)	162 (8.8)	348 (18.9)	781 (42.3)	345 (18.7)
			Level 3	12 (0.7)	18 (1.0)	217 (11.8)	826 (44.7)	74 (4.0)	0 (0.0)	12 (0.7)	49 (2.7)	76 (4.1)	38 (2.1)
		Hip osteoarthritis (n=86,023)	Level 1	7,079 (8.2)	67,411 (**78.4**)	35,009 (40.7)	1,335 (1.6)	51,727 (**60.1**)	53,290 (**61.9**)	79,823 (**92.8**)	67,451 (**78.4**)	39,300 (45.7)	67,755 (**78.8**)
			Level 2	78,719 (**91.5**)	17,838 (20.7)	42,897 (**49.9**)	50,371 (**58.6**)	31,621 (36.8)	32,620 (37.9)	5,719 (6.6)	16,947 (19.7)	43,037 (**50.0**)	17,003 (19.8)
			Level 3	225 (0.3)	774 (0.9)	8,117 (9.4)	34,317 (39.9)	2,675 (3.1)	113 (0.1)	481 (0.6)	1,625 (1.9)	3,686 (4.3)	1,265 (1.5)
		Inflammatory joint disorder (n=1,197)	Level 1	55 (4.6)	697 (**58.2**)	344 (28.7)	13 (1.1)	638 (**53.3**)	545 (45.5)	922 (**77.0**)	698 (**58.3**)	357 (29.8)	808 (**67.5**)
			Level 2	1,124 (**93.9**)	461 (38.5)	660 (**55.1**)	558 (46.6)	502 (41.9)	645 (**53.9**)	240 (20.1)	432 (36.1)	735 (**61.4**)	363 (30.3)
			Level 3	18 (1.5)	39 (3.3)	193 (16.1)	626 (**52.3**)	57 (4.8)	7 (0.6)	35 (2.9)	67 (5.6)	105 (8.8)	26 (2.2)
	Knee	Knee osteoarthritis (n=16,020)	Level 1	1,884 (11.8)	15,010 (**93.7**)	8,605 (**53.7**)	290 (1.8)	10,484 (**65.4**)	10,127 (**63.2**)	15,275 (**95.3**)	12,513 (**78.1**)	5,818 (36.3)	12,620 (**78.8**)
			Level 2	14,097 (**88.0**)	869 (5.4)	6,585 (41.1)	10,139 (**63.3**)	5,155 (32.2)	5,867 (36.6)	661 (4.1)	3,246 (20.3)	9,380 (**58.6**)	3,133 (19.6)
			Level 3	39 (0.2)	141 (0.9)	830 (5.2)	5,591 (34.9)	381 (2.4)	26 (0.2)	84 (0.5)	261 (1.6)	822 (5.1)	267 (1.7)
		Knee rheumatoid arthritis (n=304)	Level 1	11 (3.6)	231 (**76.0**)	114 (37.5)	0 (0.0)	185 (**60.9**)	122 (40.1)	247 (**81.3**)	183 (**60.2**)	66 (21.7)	210 (**69.1**)
			Level 2	291 (**95.7**)	69 (22.7)	157 (**51.6**)	189 (**62.2**)	106 (34.9)	181 (**59.5**)	49 (16.1)	108 (35.5)	208 (**68.4**)	89 (29.3)
			Level 3	2 (0.7)	4 (1.3)	33 (10.9)	115 (37.8)	13 (4.3)	1 (0.3)	8 (2.6)	13 (4.3)	30 (9.9)	5 (1.6)

Intervention-based registers	Ankle	Other (n=85)	Level 1	6 (7.1)	73 (**85.9**)	31 (36.5)	2 (2.4)	46 (**54.1**)	27 (31.8)	75 (**88.2**)	47 (**55.3**)	22 (25.9)	56 (**65.9**)
			Level 2	77 (**90.6**)	11 (12.9)	39 (**45.9**)	42 (**49.4**)	36 (42.4)	57 (**67.1**)	9 (10.6)	33 (38.8)	56 (**65.9**)	29 (34.1)
			Level 3	2 (2.4)	1 (1.2)	15 (17.6)	41 (48.2)	3 (3.5)	1 (1.2)	1 (1.2)	5 (5.9)	7 (8.2)	0 (0.0)
		Posttraumatic osteoarthritis (n=308)	Level 1	16 (5.2)	285 (**92.5**)	125 (40.6)	4 (1.3)	166 (**53.9**)	109 (35.4)	290 (**94.2**)	200 (**64.9**)	68 (22.1)	217 (**70.5**)
			Level 2	288 (**93.5**)	20 (6.5)	147 (**47.7**)	168 (**54.5**)	123 (39.9)	198 (**64.3**)	15 (4.9)	93 (30.2)	211 (**68.5**)	76 (24.7)
			Level 3	4 (1.3)	3 (1.0)	36 (11.7)	136 (44.2)	19 (6.2)	1 (0.3)	3 (1.0)	15 (4.9)	29 (9.4)	15 (4.9)
		Primary osteoarthritis (n=234)	Level 1	7 (3.0)	214 (**91.5**)	91 (38.9)	2 (0.9)	152 (**65.0**)	97 (41.5)	219 (**93.6**)	165 (**70.5**)	70 (29.9)	182 (**77.8**)
			Level 2	224 (**95.7**)	18 (7.7)	118 (**50.4**)	118 (**50.4**)	78 (33.3)	136 (58.1)	13 (5.6)	65 (27.8)	137 (**58.5**)	51 (21.8)
			Level 3	3 (1.3)	2 (0.9)	25 (10.7)	114 (48.7)	4 (1.7)	1 (0.4)	2 (0.9)	4 (1.7)	27 (11.5)	1 (0.4)
		Rheumatoid arthritis (n=73)	Level 1	70 (0.0)	45 (**61.6**)	17 (23.3)	0 (0.0)	40 (**54.8**)	20 (27.4)	57 (**78.1**)	36 (**49.3**)	10 (13.7)	48 (**65.8**)
			Level 2	3 (**95.9**)	27 (37.0)	42 (**57.5**)	23 (31.5)	29 (39.7)	52 (**71.2**)	14 (19.2)	32 (43.8)	55 (**75.3**)	24 (32.9)
			Level 3	6 (4.1)	1 (1.4)	14 (19.2)	50 (**68.5**)	4 (5.5)	1 (1.4)	2 (2.7)	5 (6.8)	8 (11.0)	1 (1.4)
	**Cruciate ligament**	Cruciate ligament injury (n=8,430)	Level 1	5,645 (**67.0**)	8,209 (**97.4**)	4,503 (**53.4**)	1,294 (15.3)	4,190 (**49.7**)	7,308 (**86.7**)	8,337 (**98.9**)	6,663 (**79.0**)	2,943 (34.9)	5,501 (**65.3**)
			Level 2	2,762 (32.8)	178 (2.1)	3,172 (37.6)	6,658 (**79.0**)	3,779 (44.8)	1,117 (13.3)	69 (0.8)	1,636 (19.4)	5,223 (**62.0**)	2,649 (31.4)
			Level 3	23 (0.3)	43 (0.5)	755 (9.0)	478 (5.7)	461 (5.5)	5 (0.1)	24 (0.3)	131 (1.6)	264 (3.1)	280 (3.3)
	**BOA**	BOA hip (n=3,440)	Level 1	1,499 (43.6)	3,234 (**94.0**)	2,600 (**75.6**)	61 (1.8)	2,230 (**64.8**)	1,708 (**49.7**)	3,209 (**93.3**)	2,662 (**77.4**)	261 (7.6)	2,305 (**67.0**)
			Level 2	1,938 (**56.3**)	195 (5.7)	799 (23.2)	3,014 (**87.6**)	1,159 (33.7)	1,729 (50.3)	218 (6.3)	741 (21.5)	2,874 (**83.5**)	1,096 (31.9)
			Level 3	3 (0.1)	11 (0.3)	41 (1.2)	365 (10.6)	51 (1.5)	3 (0.1)	13 (0.4)	37 (1.1)	305 (8.9)	39 (1.1)
		BOA knee (n=10,207)	Level 1	4,282 (42.0)	9,886 (**96.9**)	7,674 (**75.2**)	290 (2.8)	6,751 (**66.1**)	5,388 (**52.8**)	9,884 (**96.8**)	8,218 (**80.5**)	1,003 (9.8)	7,248 (**71.0**)
			Level 2	5,916 (**58.0**)	275 (2.7)	2,401 (23.5)	8,741 (**85.6**)	3,314 (32.5)	4,813 (47.2)	276 (2.7)	1,905 (18.7)	8,404 (**82.3**)	2,834 (27.8)
			Level 3	9 (0.1)	46 (0.5)	132 (1.3)	1,176 (11.5)	142 (1.4)	6 (0.1)	47 (0.5)	84 (0.8)	800 (7.8)	125 (1.2)
	**Fracture**	Ankle and foot (n=3,718)	Level 1	3,048 (**82.0**)	3,570 (**96.0**)	3,265 (**87.8**)	2,549 (**68.6**)	3,095 (**83.2**)	2,717 (**73.1**)	3,578 (**96.2**)	3,112 (**83.7**)	1,832 (**49.3**)	3,005 (**80.8**)
			Level 2	655 (17.6)	126 (3.4)	322 (8.7)	1,058 (28.5)	556 (15.0)	991 (26.7)	106 (2.9)	537 (14.4)	1,733 (46.6)	645 (17.3)
			Level 3	15 (0.4)	22 (0.6)	131 (3.5)	111 (3.0)	67 (1.8)	10 (0.3)	34 (0.9)	69 (1.9)	153 (4.1)	68 (1.8)
		Elbow and forearm (n=13,996)	Level 1	12,548 (**89.7**)	12,981 (**92.7**)	12,304 (**87.9**)	9,502 (**67.9**)	11,699 (**83.6**)	12,104 (**86.5**)	13,107 (**93.6**)	11,649 (**83.2**)	6,686 (47.8)	11,390 (**81.4**)
			Level 2	1,434 (10.2)	885 (6.3)	1,189 (8.5)	4,161 (29.7)	2,127 (15.2)	1,848 (13.2)	748 (5.3)	2,026 (14.5)	6,924 (**49.5**)	2,406 (17.2)
			Level 3	14 (0.1)	130 (0.9)	503 (3.6)	333 (2.4)	170 (1.2)	44 (0.3)	141 (1.0)	321 (2.3)	386 (2.8)	200 (1.4)
		Hip and femur (n=8,378)	Level 1	4,490 (**53.6**)	6,379 (**76.1**)	5,444 (**65.0**)	3,740 (44.6)	5,486 (**65.5**)	2,419 (28.9)	5,679 (**67.8**)	4,243 (**50.6**)	2,286 (27.3)	4,818 (**57.5**)
			Level 2	3,746 (44.7)	1,526 (18.2)	1,846 (22.0)	4,092 (**48.8**)	2,570 (30.7)	5,584 (**66.7**)	1,879 (22.4)	2,636 (31.5)	5,458 (**65.1**)	3,205 (38.3)
			Level 3	142 (1.7)	473 (5.6)	1,088 (13.0)	546 (6.5)	322 (3.8)	375 (4.5)	820 (9.8)	1,499 (17.9)	634 (7.6)	355 (4.2)
		Knee and lower leg (n=10,866)	Level 1	9,117 (**83.9**)	10,177 (**93.7**)	9,499 (**87.4**)	7,736 (**71.2**)	9,029 (**83.1**)	7,023 (**64.6**)	10,228 (**94.1**)	8,584 (**79.0**)	4,212 (38.8)	8,354 (**76.9**)
			Level 2	1,661 (15.3)	570 (5.2)	871 (8.0)	2,835 (26.1)	1,657 (15.2)	3,762 (34.6)	471 (4.3)	1,939 (17.8)	6,208 (**57.1**)	2,286 (21.0)
			Level 3	88 (0.8)	119 (1.1)	496 (4.6)	295 (2.7)	180 (1.7)	81 (0.7)	167 (1.5)	343 (3.2)	446 (4.1)	226 (2.1)
		Lumbar spine and pelvis (n=1,128)	Level 1	708 (**62.8**)	902 (**80.0**)	775 (**68.7**)	547 (48.5)	790 (**70.0**)	548 (48.6)	77.5 (**77.5**)	682 (**60.5**)	412 (36.5)	741 (**65.7**)
			Level 2	400 (35.5)	179 (15.9)	236 (20.9)	478 (42.4)	293 (26.0)	561 (**49.7**)	16.1 (16.1)	296 (26.2)	621 (**55.1**)	348 (30.9)
			Level 3	20 (1.8)	47 (4.2)	117 (10.4)	103 (9.1)	45 (4.0)	19 (1.7)	6.4 (6.4)	150 (13.3)	95 (8.4)	39 (3.5)
		Shoulder and upper arm (n=7,835)	Level 1	6,615 (**84.4**)	6,926 (**88.4**)	6,547 (**83.6**)	4,891 (**62.4**)	6,248 (**79.7**)	6,237 (**79.6**)	6,721 (**85.8**)	5,877 (**75.0**)	2,912 (37.2)	5,876 (**75.0**)
			Level 2	1,189 (15.2)	743 (9.5)	863 (11.0)	2,607 (33.3)	1,447 (18.5)	1,548 (19.8)	940 (12.0)	1,616 (20.6)	4,573 (**58.4**)	1,794 (22.9)
			Level 3	31 (0.4)	166 (2.1)	425 (5.4)	337 (4.3)	140 (1.8)	50 (0.6)	174 (2.2)	342 (4.4)	350 (4.5)	165 (2.1)
		Unspecified part of trunk, extremities or other body region (n=749)	Level 1	569 (**76.0**)	655 (**87.4**)	583 (**77.8**)	448 (**59.8**)	577 (**77.0**)	463 (**61.8**)	641 (**85.6**)	470 (**62.8**)	172 (23.0)	492 (**65.7**)
			Level 2	171 (22.8)	78 (10.4)	99 (13.2)	249 (33.2)	147 (19.6)	273 (36.4)	86 (11.5)	211 (28.2)	494 (**66.0**)	224 (29.9)
			Level 3	9 (1.2)	16 (2.1)	67 (8.9)	52 (6.9)	25 (3.3)	13 (1.7)	22 (2.9)	68 (9.1)	83 (11.1)	33 (4.4)
		Wrist and hand (n=4,222)	Level 1	3,927 (**93.0**)	3,922 (**92.9**)	3,656 (**86.6**)	2,950 (**69.9**)	3,538 (**83.8**)	3,803 (**90.1**)	4,024 (**95.3**)	3,544 (**83.9**)	2,046 (**48.5**)	3,505 (**83.0**)
			Level 2	286 (6.8)	266 (6.3)	389 (9.2)	1,178 (27.9)	622 (14.7)	403 (9.5)	163 (3.9)	603 (14.3)	2,048 (**48.5**)	658 (15.6)
			Level 3	9 (0.2)	34 (0.8)	177 (4.2)	94 (2.2)	62 (1.5)	16 (0.4)	35 (0.8)	75 (1.8)	128 (3.0)	59 (1.4)

Diagnosis-based registers

**Heart failure**	Heart failure (n=1,436)	Level 1	866 **(60.3)**	1,315 **(91.6)**	1,041 **(72.5)**	689 **(48.0)**	865 **(60.2)**	837 **(58.3)**	1,298 (90.**4**)	1,025 **(71.4)**	685 **(47.7)**	896 **(62.4)**
		Level 2	567 (39.5)	116 (8.1)	355 (24.7)	679 (47.3)	521 (36.3)	593 (41.3)	126 (8.8)	371 (25.8)	655 (45.6)	499 (34.7)
		Level 3	3 (0.2)	5 (0.3)	40 (2.8)	68 (4.7)	50 (3.5)	6 (0.4)	12 (0.8)	40 (2.8)	96 (6.7)	41 (2.9)
**Respiratory failure**	COPD (n=900)	Level 1	199 (22.1)	551 **(61.2)**	215 (23.9)	250 (27.8)	357 (39.7)	162 (18.0)	479 **(53.2)**	179 (19.9)	212 (23.6)	320 (35.6)
		Level 2	683 **(75.9)**	296 (32.9)	492 **(54.7)**	517 **(57.4)**	463 **(51.4)**	696 **(77.3)**	343 (38.1)	465 **(51.7)**	541 **(60.1)**	489 **(54.3)**
		Level 3	18 (2.0)	53 (5.9)	193 (21.4)	133 (14.8)	80 (8.9)	42 (4.7)	78 (8.7)	256 (28.4)	147 (16.3)	91 (10.1)
	Lung fibrosis (n=150)	Level 1	44 (29.3)	99 **(66.0)**	57 (38.0)	55 (36.7)	75 **(50.0)**	25 (16.7)	72 **(48.0)**	32 (21.3)	44 (29.3)	67 (44.7)
		Level 2	103 **(68.7)**	47 (31.3)	65 **(43.3)**	83 **(55.3)**	69 (46.0)	118 **(78.7)**	66 (44.0)	61 **(40.7)**	86 **(57.3)**	70 **(46.7)**
		Level 3	3 (2.0)	4 (2.7)	28 (18.7)	12 (8.0)	6 (4.0)	7 (4.7)	12 (8.0)	57 (38.0)	20 (13.3)	13 (8.7)
**Psoriasis**	Psoriasis vulgaris (n=1,704)	Level 1	1,425 **(83.6)**	1,645 **(96.5)**	1,469 **(86.2)**	666 (39.1)	881 **(51.7)**	1,444 **(84.7)**	1,659 **(97.4)**	1,525 **(89.5)**	884 **(51.9)**	1,113 **(65.3)**
		Level 2	279 (16.4)	54 (3.2)	214 (12.6)	914 **(53.6)**	719 (42.2)	258 (15.1)	41 (2.4)	161 (9.4)	755 (44.3)	529 (31.0)
		Level 3	0 (0.0)	5 (0.3)	21 (1.2)	124 (7.3)	104 (6.1)	2 (0.1)	4 (0.2)	18 (1.1)	65 (3.8)	62 (3.6)
	Psoriasis vulgaris + anthropathic (n=537)	Level 1	311 **(57.9)**	484 **(90.1)**	368 **(68.5)**	91 (16.9)	234 (43.6)	356 **(66.3)**	494 **(92.0)**	422 **(78.6)**	141 (26.3)	314 **(58.5)**
		Level 2	226 (42.1)	50 (9.3)	154 (28.7)	359 **(66.9)**	262 **(48.8)**	180 (33.5)	41 (7.6)	101 (18.8)	342 **(63.7)**	194 (36.1)
		Level 3	0 (0.0)	3 (0.6)	15 (2.8)	87 (16.2)	41 (7.6)	1 (0.2)	2 (0.4)	14 (2.6)	54 (10.1)	29 (5.4)
	Psoriasis unspecified (n=439)	Level 1	328 **(74.7)**	425 **(96.8)**	346 **(78.8)**	128 (29.2)	203 (46.2)	351 **(80.0)**	425 **(96.8)**	382 **(87.0)**	206 (46.9)	298 **(67.9)**
		Level 2	110 (25.1)	11 (2.5)	86 (19.6)	263 **(59.9)**	204 **(46.5)**	88 (20.0)	13 (3.0)	50 (11.4)	210 **(47.8)**	124 (28.2)
		Level 3	1 (0.2)	3 (0.7)	7 (1.6)	48 (10.9)	32 (7.3)	0 (0.0)	1 (0.2)	7 (1.6)	23 (5.2)	17 (3.9)
**Rheumatology**	Rheumatoid arthritis (n=17,870)	Level 1	8,758 (49.0)	14,099 **(78.9)**	10,564 **(59.1)**	2,203 (12.3)	10,107 **(56.6)**	9,741 **(54.5)**	14,912 **(83.4)**	11,825 **(66.2)**	3,173 (17.8)	10,970 **(61.4)**
		Level 2	9,065 **(50.7)**	3,539 (19.8)	6,519 (36.5)	13,138 **(73.5)**	7,046 (39.4)	8,060 (45.1)	2,727 (15.3)	5,447 (30.5)	12,734 **(71.3)**	6,196 (34.7)
		Level 3	47 (0.3)	232 (1.3)	787 (4.4)	2,529 (14.2)	717 (4.0)	69 (0.4)	231 (1.3)	598 (3.3)	1,963 (11.0)	704 (3.9)
	Spondyloarthropathy (n=8,782)	Level 1	4,548 **(51.8)**	7,292 **(83.0)**	4,929 **(56.1)**	955 (10.9)	4,316 **(49.1)**	5,051 **(57.5)**	7,470 **(85.1)**	5,502 **(62.7)**	1,263 (14.4)	4,733 **(53.9)**
		Level 2	4,225 (48.1)	1,422 (16.2)	3,392 (38.6)	6,154 **(70.1)**	3,900 (44.4)	3,714 (42.3)	1,245 (14.2)	2,925 (33.3)	6,189 **(70.5)**	3,489 (39.7)
		Level 3	9 (0.1)	68 (0.8)	461 (5.2)	1,673 (19.1)	566 (6.4)	17 (0.2)	67 (0.8)	355 (4.0)	1,330 (15.1)	560 (6.4)
	Inflammatory systemic disorder (n=1,614)	Level 1	920 **(57.0)**	1,355 **(84.0)**	942 **(58.4)**	302 (18.7)	792 **(49.1)**	969 **(60.0)**	1,393 **(86.3)**	1,009 **(62.5)**	341 (21.1)	834 **(51.7)**
		Level 2	686 (42.5)	245 (15.2)	563 (34.9)	1,103 **(68.3)**	730 (45.2)	639 (39.6)	207 (12.8)	521 (32.3)	1,084 **(67.2)**	683 (42.3)
		Level 3	8 (0.5)	14 (0.9)	109 (6.8)	209 (12.9)	92 (5.7)	6 (0.4)	14 (0.9)	84 (5.2)	189 (11.7)	97 (6.0)
	Juvenile rheumatoid diseases (n=1,088)	Level 1	552 **(50.7)**	866 **(79.6)**	611 **(56.2)**	158 (14.5)	545 **(50.1)**	593 **(54.5)**	879 **(80.8)**	660 **(60.7)**	198 (18.2)	571 **(52.5)**
		Level 2	532 (48.9)	205 (18.8)	425 (39.1)	755 **(69.4)**	482 (44.3)	490 (45.0)	193 (17.7)	377 (34.7)	731 **(67.2)**	458 (42.1)
		Level 3	4 (0.4)	17 (1.6)	52 (4.8)	175 (16.1)	61 (5.6)	5 (0.5)	16 (1.5)	51 (4.7)	159 (14.6)	59 (5.4)
	Vasculitis (n=410)	Level 1	249 **(60.7)**	368 **(89.8)**	276 **(67.3)**	91 (22.2)	227 **(55.4)**	239 **(58.3)**	374 **(91.2)**	279 **(68.0)**	96 (23.4)	241 **(58.8)**
		Level 2	161 (39.3)	34 (8.3)	117 (28.5)	285 **(69.5)**	169 (41.2)	170 (41.5)	29 (7.1)	123 (30.0)	275 **(67.1)**	154 (37.6)
		Level 3	0 (0.0)	8 (2.0)	17 (4.1)	34 (8.3)	14 (3.4)	1 (0.2)	7 (1.7)	8 (2.0)	39 (9.5)	15 (3.7)
	Other arthritis (n=1698)	Level 1	856 **(50.4)**	1,414 **(83.3)**	982 **(57.8)**	182 (10.7)	930 **(54.8)**	992 **(58.4)**	1,495 **(88.0)**	1,168 **(68.8)**	302 (17.8)	1,059 **(62.4)**
		Level 2	837 (49.3)	273 (16.1)	629 (37.0)	1,268 **(74.7)**	687 (40.5)	703 (41.4)	190 (11.2)	480 (28.3)	1,193 **(70.3)**	573 (33.7)
		Level 3	5 (0.3)	11 (0.6)	87 (5.1)	248 (14.6)	81 (4.8)	3 (0.2)	13 (0.8)	50 (2.9)	203 (12.0)	66 (3.9)
**General population data** (n=49,169)		Level 1	44,279 **(90.1)**	48,371 **(98.4)**	44,848 **(91.2)**	24,946 **(50.8)**	32,721 **(66.5)**	-	-	-	-	-
		Level 2	4,840 (9.8)	600 (1.2)	3,785 (7.7)	22,185 (45.1)	15,126 (30.8)	-	-	-	-	-
		Level 3	50 (0.1)	198 (0.4)	536 (1.1)	2,038 (4.1)	1,322 (2.7)	-	-	-	-	-

The highest proportion in bold; Level 1: No problems; Level 2: Some/moderate problems; Level 3: confined to bed/unable to/extreme problems; Proportions rounded off to one decimal using MS Excel

For peer review only - http://bmjopen.bmj.com/site/about/guidelines.xhtml

Table S4: Median (IQR) EQ VAS value at baseline, 1-year follow-up and the change in the 9 National Quality Registers (NQRs) and in the general population

Time	Variable	Median EQ VAS value (IQR)									
		Intervention-based registers							Diagnosis-based registers		General population
		Spine	Hip	Knee	Ankle	Cruciate ligament	BOA	Fracture	Heart failure	Respiratory failure	
**Baseline**	**Overall**	50.0 (35.0)	58.0 (35.0)	70.0 (30.0)	57.5 (30.0)	68.0 (32.0)	70.0 (23.0)	90.0 (20.0)	70.0 (30.0)	50.0 (25.0)	85.0 (15.0)
	**Sex**										
	Men	50.0 (40.0)	60.0 (30.0)	71.0 (32.0)	60.0 (35.0)	70.0 (30.0)	75.0 (25.0)	90.0 (20.0)	70.0 (25.0)	50.0 (25.0)	85.0 (15.0)
	Women	45.0 (30.0)	50.0 (31.0)	65.0 (30.0)	50.0 (32.5)	66.0 (34.0)	70.0 (25.0)	90.0 (21.0)	60.0 (25.0)	50.0 (28.0)	84.0 (20.0)
	**Age group**										
	<30	45.0 (35.0)	43.0 (40.8)	-	52.5 (23.8)	69.0 (31.0)	70.0 (1.3)	95.0 (15.0)	-	-	85.0 (15.0)
	30-39	41.0 (30.0)	50.0 (35.0)	60.5 (36.8)	48.0 (26.3)	68.0 (30.0)	65.0 (30.0)	93.0 (15.0)	70.0 (30.0)	-	85.0 (15.0)
	40-49	45.0 (32.0)	55.0 (35.0)	59.5 (40.0)	47.5 (30.0)	65.0 (36.0)	70.0 (30.0)	92.0 (15.0)	67.5 (20.0)	-	85.0 (15.0)
	50-59	50.0 (35.0)	55.0 (34.0)	62.0 (39.0)	55.0 (35.0)	66.0 (34.0)	70.0 (30.0)	92.0 (20.0)	62.0 (25.0)	50.0 (45.0)	84.0 (20.0)
	60-69	50.0 (36.5)	60.0 (35.0)	70.0 (30.0)	55.0 (35.0)	69.0 (40.0)	70.0 (27.0)	91.0 (20.0)	70.0 (20.0)	50.0 (29.0)	83.0 (20.0)
	70-79	50.0 (40.0)	60.0 (35.0)	70.0 (31.0)	65.0 (30.8)	-	70.0 (20.3)	90.0 (24.0)	70.0 (30.0)	50.0 (25.0)	80.0 (20.0)
	80+	50.0 (36.0)	52.0 (30.0)	70.0 (30.0)	50.0 (30.0)	-	70.0 (27.0)	75.0 (35.0)	60.0 (25.0)	50.0 (30.0)	80.0 (40.0)
	**BMI (Kg/m^2)**										
	Under-weight	40.0 (34.8)	50.0 (30.0)	70.0 (25.3)	27.0 (27.5)	76.5 (30.0)	67.5 (20.0)	-	-	50.0 (26.3)	80.0 (20.0)
	Normal	50.0 (35.0)	60.0 (35.0)	70.0 (33.0)	60.0 (31.2)	70.0 (31.0)	75.0 (25.0)	-	-	50.0 (30.0)	85.0 (15.0)
	Over-weight	50.0 (36.0)	60.0 (35.0)	70.0 (31.0)	60.0 (32.0)	70.0 (31.0)	70.0 (20.0)	-	-	50.0 (25.0)	80.0 (20.0)
	Obesity class I	50.0 (35.0)	53.0 (30.0)	65.0 (30.0)	55.0 (30.0)	62.0 (38.0)	69.0 (30.0)	-	-	50.0 (29.0)	80.0 (30.0)
	Obesity class II	40.0 (33.0)	50.0 (37.0)	60.0 (38.0)	50.0 (25.0)	62.0 (37.5)	65.0 (29.0)	-	-	50.0 (25.0)	75.0 (35.0)
	Obesity class III	40.0 (30.0)	50.0 (40.0)	60.0 (32.3)	42.0 (22.5)	64.5 (16.5)	58.0 (30.0)	-	-	60.0 (20.5)	70.0 (35.0)
**1-year follow-up**	**Overall**	70.0 (35.0)	80.0 (23.0)	80.0 (20.0)	75.0 (22.0)	80.0 (24.0)	75.0 (25.0)	80.0 (23.0)	70.0 (20.3)	50.0 (28.0)	-
	**Sex**										
	Men	75.0 (28.0)	81.0 (23.0)	80.0 (20.0)	75.0 (20.0)	80.0 (23.0)	75.0 (25.0)	84.0 (24.0)	70.0 (20.0)	50.0 (25.0)	-
	Women	70.0 (35.0)	80.0 (30.0)	80.0 (25.0)	70.0 (26.5)	78.0 (24.0)	74.0 (25.0)	80.0 (23.0)	70.0 (30.0)	50.0 (25.0)	-
	**Age group**										
	<30	80.0 (25.0)	80.0 (23.8)	-	67.5 (42.3)	80.0 (24.0)	76.5 (26.0)	88.0 (20.0)	-	-	-
	30-39	75.0 (29.0)	80.0 (20.0)	82.5 (10.0)	70.0 (25.5)	80.0 (25.0)	78.0 (15.0)	88.0 (20.0)	90.0 (30.0)	-	-
	40-49	75.0 (30.0)	85.0 (23.0)	75.0 (28.0)	70.0 (27.5)	80.0 (23.0)	70.0 (23.0)	85.0 (20.0)	73.0 (20.0)	-	-
	50-59	75.0 (36.0)	83.0 (24.0)	80.0 (28.0)	75.0 (20.0)	78.0 (24.0)	73.0 (25.0)	85.0 (25.0)	70.0 (24.0)	40.0 (35.0)	-
	60-69	73.0 (34.0)	85.0 (25.0)	80.0 (20.0)	75.0 (20.8)	84.0 (22.0)	74.0 (25.0)	86.0 (21.0)	75.0 (24.3)	50.0 (25.0)	-
	70-79	70.0 (30.0)	80.0 (25.0)	80.0 (20.0)	75.0 (25.0)	-	75.0 (26.0)	80.0 (25.0)	70.0 (25.0)	50.0 (27.3)	-
	80+	60.0 (29.0)	75.0 (34.0)	78.0 (30.0)	60.0 (30.0)	-	71.0 (30.0)	65.0 (30.0)	65.0 (30.0)	50.0 (23.8)	-
	**BMI**										
	Underweight	70.0 (38.5)	76.0 (30.0)	70.0 (18.8)	45.0 (15.0)	78.5 (25.5)	74.5 (17.8)	-	-	42.5 (26.3)	-
	Normal weight	75.0 (30.0)	85.0 (25.0)	80.0 (20.0)	75.0 (20.0)	80.0 (23.0)	78.0 (24.0)	-	-	50.0 (25.0)	-
	Over-weight	75.0 (31.0)	80.0 (21.0)	80.0 (20.0)	75.0 (10.0)	80.0 (25.0)	75.0 (25.0)	-	-	50.0 (23.0)	-
	Obesity class I	70.0 (30.0)	80.0 (30.0)	80.0 (25.0)	70.0 (23.5)	75.0 (25.0)	70.0 (29.0)	-	-	50.0 (29.5)	-
	Obesity class II	65.0 (40.0)	75.0 (30.0)	75.0 (30.0)	75.0 (37.5)	75.0 (32.0)	70.0 (28.0)	-	-	50.0 (23.8)	-
	Obesity class III	66.0 (36.3)	70.0 (37.8)	70.0 (34.0)	35.0 (21.3)	68.0 (34.8)	60.0 (35.0)	-	-	50.0 (15.0)	-

For peer review only

**Change**	**Overall**	20.0 (40.0)	20.0 (35.0)	10.0 (26.0)	11.5 (30.0)	10.0 (30.0)	0.0 (19.0)	-5.0 (15.0)	0.0 (20.0)	0.0 (28.0)	-
	**Sex**										
	Men	17.0 (36.0)	18.0 (32.0)	8.0 (22.0)	10.0 (30.0)	10.0 (29.0)	0.0 (20.0)	-5.0 (15.0)	0.0 (16.0)	0.0 (25.0)	-
	Women	20.0 (38.0)	20.0 (35.0)	10.0 (31.0)	15.0 (35.0)	10.0 (30.0)	2.0 (21.0)	-5.0 (15.0)	0.0 (22.0)	0.0 (30.0)	-
	**Age group**										
	<30	25.0 (35.0)	30.0 (40.0)	-	12.5 (45.3)	9.0 (30.0)	4.0 (22.3)	-5.0 (15.0)	-	-	-
	30-39	25.0 (37.0)	24.0 (31.0)	14.5 (35.3)	17.5 (22.3)	10.0 (29.0)	10.0 (21.0)	-5.0 (15.0)	0.0 (0.0)	-	-
	40-49	25.0 (40.0)	24.0 (35.0)	15.0 (35.0)	20.0 (27.5)	13.0 (32.0)	5.0 (20.0)	-5.0 (15.0)	7.5 (14.0)	-	-
	50-59	20.0 (36.0)	20.0 (34.0)	10.0 (31.0)	15.0 (30.0)	10.0 (27.0)	4.0 (20.0)	-5.0 (15.0)	9.0 (20.0)	-10.0 (25.0)	-
	60-69	20.0 (38.0)	20.0 (35.0)	10.0 (29.0)	15.0 (32.0)	7.0 (41.0)	1.0 (18.0)	-5.0 (13.0)	0.0 (15.0)	0.0 (28.3)	-
	70-79	13.0 (30.0)	18.0 (35.0)	9.0 (25.0)	10.0 (25.0)	-	0.0 (20.0)	-5.0 (15.0)	0.0 (20.0)	0.0 (29.5)	-
	80+	10.0 (33.0)	15.0 (31.0)	7.0 (25.0)	10.0 (27.0)	-	0.0 (21.0)	-8.0 (22.0)	0.0 (20.0)	0.0 (28.3)	-
	**BMI**										
	Underweight	20.0 (36.5)	20.0 (30.0)	9.5 (27.5)	18.0 (12.5)	1.5 (22.3)	-0.5 (21.5)	-	-	-5.0 (28.5)	-
	Normal weight	20.0 (35.0)	20.0 (34.0)	10.0 (26.0)	10.0 (30.0)	9.0 (30.0)	1.0 (15.0)	-	-	0.0 (30.0)	-
	Over-weight	20.0 (39.0)	20.0 (32.0)	10.0 (26.0)	12.5 (30.0)	9.0 (30.0)	0.0 (19.0)	-	-	0.0 (22.8)	-
	Obesity class I	16.0 (35.0)	20.0 (39.0)	10.0 (30.0)	13.0 (30.0)	11.0 (35.0)	0.0 (23.0)	-	-	0.0 (33.0)	-
	Obesity class II	15.0 (35.0)	20.0 (39.0)	10.0 (32.0)	25.0 (42.5)	18.0 (35.5)	3.0 (25.0)	-	-	-2.5 (30.0)	-
	Obesity class III	15.0 (40.0)	20.0 (40.0)	10.0 (38.0)	0.0 (13.8)	-4.0 (27.5)	4.0 (20.0)	-	-	0.0 (20.0)	-

BMI categories: Underweight: <18.5; Normal weight: 18.5–24.9; Overweight: 25.0–29.9; Obesity class I: 30.0–34.9; Obesity class II: 35.0–39.9; Obesity class III: ≥40

For peer review only

Table S5: Two-level random intercept model of predictive effect of diagnoses on EQ VAS score at baseline and at 1-year follow-up, adjusted for sex and age

Variable	Baseline		1-year follow-up	
**Category**	**Estimate**	**SE**	**Estimate**	**SE**
Intercept	83.58	0.1894	82.01	0.1822
Spine, disc hernia	-34.61	0.2170	-7.52	0.2087
Spine, other spinal conditions	-33.03	0.5872	-11.57	0.5647
Spine, segmental instability	-32.85	0.3414	-11.07	0.3283
Spine, spinal stenosis	-29.91	0.1816	-11.77	0.1746
Spine, spondylolysis	-30.49	0.4469	-10.18	0.4298
Hip, avascular necrosis	-29.65	0.5306	-4.84	0.5104
Hip, childhood hip disorder	-23.21	0.4920	0.80	0.4731
Hip, hip osteoarthritis	-22.37	0.1474	-0.30	0.1418
Hip, inflammatory joint disorder	-30.74	0.6082	-9.12	0.5850
Knee osteoarthritis	-14.22	0.2088	-0.91	0.2008
Knee rheumatoid arthritis	-22.56	1.1891	-9.71	1.1436
Ankle, others	-21.57	2.2660	-10.41	2.1790
Ankle, posttraumatic OA	-23.77	1.2164	-8.54	1.1698
Ankle, primary OA	-21.97	1.3805	-4.64	1.3277
Ankle, rheumatoid arthritis	-32.78	2.4670	-15.98	2.3726
Cruciate ligament injury	-18.18	0.2627	-6.08	0.2527
BOA hip	-10.97	0.5088	-9.42	0.4893
BOA knee	-9.88	0.3193	-6.55	0.3071
Fracture, ankle and foot	7.98	0.3836	2.46	0.3689
Fracture, elbow and forearm	9.33	0.2245	4.25	0.2159
Fracture, hip and femur	-3.47	0.3032	-9.98	0.2916
Fracture, knee and lower leg	7.88	0.2427	0.37	0.2334
Fracture, lumbar, spine and pelvis	-0.83	0.7293	-5.84	0.7014
Fracture, shoulder and upper arm	6.16	0.2855	0.40	0.2746
Fracture, unspecified part of trunk, extremities or other body region	3.98	0.8303	-7.40	0.7986
Fracture, wrist and hand	8.06	0.3601	3.97	0.3463
Heart failure	-14.14	0.6524	-9.14	0.6274
Respiratory failure, COPD	-27.81	0.8386	-27.11	0.8065
Respiratory failure, pulmonary fibrosis	-25.88	2.0265	-27.06	1.9490
n	251,008		251,008	
AIC	2,231,535		2,211,956	
BIC	2,231,942		2,212,363	

AIC: Akaike information criterion; AIC: Baysian information criterion; SE: Standard error; reference group: General population

For peer review only

For peer review only - http://bmjopen.bmj.com/site/about/guidelines.xhtml

Table S6: Prevalence of reported problems on EQ-5D-3L by diagnosis and mean EQ VAS, and EQ-5D indices (TTO, VAS) among patients with complete data at baseline regardless of follow-up status and in the general population

	Patient group in NQRs	Diagnosis	Severity level	Mobility	Self-care	Usual activities	Pain/ discomfort	Anxiety/ depression	Mean (SD)		
				n (%)	n (%)	n (%)	n (%)	n (%)	EQ VAS*	EQ-5D index (TTO) **	EQ-5D index (VAS) **
Intervention-based registers	**Spine**	Disc hernia (n=19,214)	Level 1	2,925 (15.2)	12,853 (**66.9**)	3,464 (18.0)	135 (0.7)	6,634 (34.5)			
			Level 2	15,295 (**79.6**)	5,958 (31.0)	9,815 (**51.1**)	8,181 (42.6)	10,811 (**56.3**)			
			Level 3	994 (5.2)	403 (2.1)	5,935 (30.9)	10,898 (**56.7**)	1,769 (9.2)	45.0 (22.5)	0.66 (0.13)	45.2 (15.9)
		Other spinal conditions (n=2,209)	Level 1	435 (19.7)	1,746 (**79.0**)	615 (27.8)	21 (1.0)	876 (39.7)			
			Level 2	1,714 (**77.6**)	435 (19.7)	1,085 (**49.1**)	922 (41.7)	1,150 (**52.1**)			
			Level 3	60 (2.7)	28 (1.3)	509 (23.0)	1,266 (**57.3**)	183 (8.3)	45.8 (22.9)	0.69 (0.13)	47.9 (16.8)
		Segmental instability (n=5,892)	Level 1	1,851 (31.4)	4,473 (**75.9**)	1,184 (20.1)	28 (0.5)	1,965 (33.4)			
			Level 2	4,000 (**67.9**)	1,374 (23.3)	3,402 (**57.7**)	2,852 (48.4)	3,442 (**58.4**)			
			Level 3	41 (0.7)	45 (0.8)	1,306 (22.2)	3,012 (**51.1**)	485 (8.2)	45.8 (20.9)	0.69 (0.12)	48.3 (16.3)
		Spinal stenosis (n=38,449)	Level 1	3,985 (10.4)	32,361 (**84.2**)	12,289 (32.0)	237 (0.6)	17,157 (44.6)			
			Level 2	34,146 (**88.8**)	5,703 (14.8)	20,734 (**53.9**)	19,231 (**50.0**)	19,155 (**49.8**)			
			Level 3	318 (0.8)	385 (1.0)	5,426 (14.1)	18,981 (49.4)	2,137 (5.6)	48.6 (22.2)	0.71 (0.12)	50.0 (15.8)
		Spondylolysis (n=3,362)	Level 1	887 (26.4)	2,859 (**85.0**)	928 (27.6)	21 (0.6)	1,271 (37.8)			
			Level 2	2,449 (**72.8**)	482 (14.3)	1,877 (**55.8**)	1,723 (**51.2**)	1,847 (**54.9**)			
			Level 3	26 (0.8)	21 (0.6)	557 (16.6)	1,618 (48.1)	244 (7.3)	48.1 (21.8)	0.71 (0.13)	50.1 (16.7)
	**Hip**	Avascular necrosis (n=2,070)	Level 1	106 (5.1)	1,252 (**60.5**)	455 (22.0)	40 (1.9)	930 (44.9)			
			Level 2	1,908 (**92.2**)	761 (36.8)	1,065 (**51.4**)	748 (36.1)	958 (**46.3**)			
			Level 3	56 (2.7)	57 (2.8)	550 (26.6)	1,282 (**61.9**)	182 (8.8)	49.0 (24.0)	0.66 (0.13)	45.4 (15.8)
		Childhood hip disorder (n=2,294)	Level 1	165 (7.2)	1,698 (**74.0**)	776 (33.8)	26 (1.1)	1,226 (**53.4**)			
			Level 2	2,110 (**92.0**)	567 (24.7)	1,227 (**53.5**)	1,210 (**52.7**)	963 (42.0)			
			Level 3	19 (0.8)	29 (1.3)	291 (12.7)	1,058 (46.1)	105 (4.6)	55.2 (22.8)	0.71 (0.12)	51.4 (15.8)
		Hip osteoarthritis (n=103,286)	Level 1	8,320 (8.1)	80,304 (**77.7**)	41,136 (39.8)	1,562 (1.5)	61,196 (**59.2**)			
			Level 2	94,660 (**91.6**)	21,997 (21.3)	51,946 (**50.3**)	59,506 (**57.6**)	38,663 (37.4)			
			Level 3	306 (0.3)	985 (1.0)	10,204 (9.9)	42,218 (40.9)	3,427 (3.3)	56.3 (22.2)	0.73 (0.11)	54.0 (15.4)
		Inflammatory joint disorder (n=1,377)	Level 1	64 (4.6)	795 (**57.7**)	388 (28.2)	15 (1.1)	723 (**52.5**)			
			Level 2	1,292 (**93.8**)	538 (39.1)	762 (**55.3**)	634 (46.0)	586 (42.6)			
			Level 3	21 (1.5)	44 (3.2)	227 (16.5)	728 (**52.9**)	68 (4.9)	47.6 (22.6)	0.69 (0.12)	49.0 (15.2)
	**Knee**	Knee osteoarthritis (n=22,612)	Level 1	2,511 (11.1)	20,989 (**92.8**)	11,596 (**51.3**)	404 (1.8)	14,393 (**63.7**)			
			Level 2	20,047 (**88.7**)	1,408 (6.2)	9,705 (42.9)	14,073 (**62.2**)	7,604 (33.6)			
			Level 3	54 (0.2)	215 (1.0)	1,311 (5.8)	8,135 (36.0)	615 (2.7)	63.9 (22.4)	0.76 (0.11)	57.3 (15.3)
		Knee rheumatoid arthritis (n=450)	Level 1	14 (3.1)	330 (**73.3**)	160 (35.6)	2 (0.4)	261 (**58.0**)			
			Level 2	433 (**96.2**)	116 (25.8)	241 (**53.6**)	256 (**56.9**)	171 (38.0)			
			Level 3	3 (0.7)	4 (0.9)	49 (10.9)	192 (42.7)	18 (4.0)	53.8 (22.2)	0.72 (0.11)	52.3 (15.3)

For peer review only - http://bmjopen.bmj.com/site/about/guidelines.xhtml

Intervention-based registers	**Ankle**	Other (n=147)	Level 1	7 (4.8)	120 (**81.6**)	43 (29.3)	8 (2.4)	73 (**49.7**)			
			Level 2	133 (**90.5**)	23 (15.6)	71 (**48.3**)	72 (**49.4**)	64 (43.5)			
			Level 3	7 (4.8)	4 (2.7)	33 (22.4)	67 (48.2)	10 (6.8)	56.3 (22.5)	0.70 (0.12)	49.4 (16.6)
		Posttraumatic osteoarthritis (n=517)	Level 1	26 (5.0)	476 (**92.1**)	194 (37.5)	4 (1.3)	273 (**52.8**)			
			Level 2	487 (**94.2**)	36 (7.0)	260 (**50.3**)	276 (**54.5**)	214 (41.4)			
			Level 3	4 (0.8)	5 (1.0)	63 (12.2)	237 (44.2)	30 (5.8)	56.9 (20.9)	0.72 (0.12)	51.5 (15.5)
		Primary osteoarthritis (n=374)	Level 1	9 (2.4)	339 (**90.6**)	141 (37.7)	4 (0.9)	236 (**63.1**)			
			Level 2	360 (**96.3**)	32 (8.6)	193 (**51.6**)	196 (**50.4**)	127 (34.0)			
			Level 3	5 (1.3)	3 (0.8)	40 (10.7)	174 (48.7)	11 (2.9)	57.5 (22.0)	0.73 (0.11)	52.7 (14.8)
		Rheumatoid arthritis (n=94)	Level 1	0 (0.0)	60 (**63.8**)	22 (23.4)	0 (0.0)	52 (**55.3**)			
			Level 2	91 (**96.8**)	32 (34.0)	57 (**60.6**)	33 (31.5)	37 (39.4)			
			Level 3	3 (3.2)	2 (2.1)	15 (16.0)	61 (**68.5**)	5 (5.3)	47.9 (22.4)	0.68 (0.11)	46.6 (14.2)
	**Cruciate ligament**	Cruciate ligament injury (n=18,530)	Level 1	12,195 (**65.8**)	17,997 (**97.1**)	9,878 (**53.3**)	2,851 (15.4)	9,046 (**48.8**)			
			Level 2	6,285 (33.9)	423 (2.3)	6,846 (36.9)	14,599 (**78.8**)	8,359 (45.1)			
			Level 3	50 (0.3)	110 (0.6)	1,806 (9.7)	1,080 (5.8)	1,125 (6.1)	62.4 (22.8)	0.82 (0.12)	65.6 (16.4)
	**BOA**	BOA hip (n=8,044)	Level 1	3,271 (40.7)	7,509 (**93.3**)	5,886 (**73.2**)	183 (2.3)	5,040 (**62.7**)			
			Level 2	4,769 (**59.3**)	503 (6.3)	2,036 (25.3)	6,778 (**84.3**)	2,864 (35.6)			
			Level 3	4 (0.0)	32 (0.4)	122 (1.5)	1,083 (13.5)	140 (1.7)	65.8 (19.1)	0.83 (0.10)	66.7 (14.4)
		BOA knee (n=24,846)	Level 1	9,557 (38.5)	23,888 (**96.1**)	17,812 (**71.7**)	713 (2.9)	15,824 (**63.7**)			
			Level 2	15,270 (**61.5**)	837 (3.4)	6,582 (26.5)	20,510 (**82.5**)	8,505 (34.2)			
			Level 3	19 (0.1)	121 (0.5)	452 (1.8)	3,623 (14.6)	517 (2.1)	66.6 (19.4)	0.83 (0.11)	66.2 (14.9)
	**Fracture**	Ankle and foot (n=6,918)	Level 1	5,522 (**79.8**)	6,567 (**94.9**)	5,916 (**85.5**)	4,639 (**67.1**)	5,590 (**80.8**)			
			Level 2	1,352 (19.5)	290 (4.2)	711 (10.3)	2,034 (29.4)	1,196 (17.3)			
			Level 3	44 (0.6)	61 (0.9)	291 (4.2)	245 (3.5)	132 (1.9)	86.2 (17.3)	0.91 (0.11)	79.4 (15.1)
		Elbow and forearm (n=25,061)	Level 1	22,079 (**88.1**)	22,724 (**90.7**)	21,352 (**85.2**)	16,414 (**65.5**)	20,355 (**81.2**)			
			Level 2	2,938 (11.7)	1972 (7.9)	2,512 (10.0)	7,885 (31.5)	4,321 (17.2)			
			Level 3	44 (0.2)	365 (1.5)	1,197 (4.8)	762 (3.0)	385 (1.5)	86.2 (17.2)	0.91 (0.10)	80.1 (14.1)
		Hip and femur (n=1,609)	Level 1	7,769 (**46.0**)	11,190 (**66.2**)	9,314 (**55.1**)	6,756 (40.0)	9,865 (**58.4**)			
			Level 2	8,535 (50.5)	3,982 (23.6)	4,078 (24.1)	8,738 (**51.7**)	6,023 (35.6)			
			Level 3	600 (3.5)	1,732 (10.2)	3,512 (20.8)	1,410 (8.3)	1,016 (6.0)	68.9 (24.5)	0.80 (0.16)	64.9 (21.0)
		Knee and lower leg (n=18,872)	Level 1	15,311 (**81.1**)	17,313 (**91.7**)	15,987 (**84.7**)	13,022 (**69.0**)	15,195 (**80.5**)			
			Level 2	3,283 (17.4)	1,182 (6.3)	1,736 (9.2)	5,229 (27.7)	3,283 (17.4)			
			Level 3	278 (1.5)	377 (2.0)	1,149 (6.1)	621 (3.3)	394 (2.1)	85.7 (17.9)	0.91 (0.12)	79.3 (15.9)
		Lumbar, spine and pelvis (n=2,405)	Level 1	1,298 (**54.0**)	1,735 (**72.1**)	1,444 (**60.0**)	1,049 (43.6)	1,507 (**62.7**)			
			Level 2	1,058 (44.0)	501 (20.8)	550 (22.9)	1,115 (**46.4**)	765 (31.8)			
			Level 3	49 (2.0)	169 (7.0)	411 (17.1)	241 (10.0)	133 (5.5)	73.0 (24.3)	0.82 (0.16)	67.5 (21.3)
		Shoulder and upper arm (n=14,240)	Level 1	11,546 (**81.1**)	12,159 (**85.4**)	11,323 (**79.5**)	8,519 (**59.8**)	10,806 (**75.9**)			
			Level 2	2,606 (18.3)	1,624 (11.4)	1841 (12.9)	4,995 (35.1)	3,103 (21.8)			
			Level 3	88 (0.6)	457 (3.2)	1,076 (7.6)	726 (5.1)	331 (2.3)	82.8 (20.0)	0.89 (0.12)	77.1 (16.9)
		Unspecified part of trunk, extremities or other body region (n=1,698)	Level 1	1,202 (**70.8**)	1,431 (**84.3**)	1,253 (**73.8**)	947 (**55.8**)	1,207 (**71.1**)			
			Level 2	474 (27.9)	213 (12.5)	263 (15.5)	610 (35.9)	430 (25.3)			
			Level 3	22 (1.3)	54 (3.2)	182 (10.7)	141 (8.3)	61 (3.6)	80.6 (22.0)	0.87 (0.15)	73.7 (19.9)
		Wrist and hand (n=8,215)	Level 1	7,533 (**91.7**)	7,522 (**91.6**)	6,947 (**84.6**)	5,617 (**68.4**)	6,783 (**82.6**)			
			Level 2	661 (8.0)	596 (7.3)	847 (10.3)	2,386 (29.0)	1,288 (15.7)			
			Level 3	21 (0.3)	97 (1.2)	421 (5.1)	212 (2.6)	144 (1.8)	87.6 (16.1)	0.92 (0.10)	80.7 (13.6)

For peer review only - http://bmjopen.bmj.com/site/about/guidelines.xhtml

Diagnosis-based registers	**Heart failure**	Heart failure (n=20,771)	Level 1	12,331 (**59.4**)	18,741 (**90.2**)	14,424 (**69.4**)	10,754 (**51.8**)	12,213 (**58.8**)			
			Level 2	8,334 (40.1)	1,817 (8.7)	5,548 (26.7)	8,920 (42.9)	7,748 (37.3)			
			Level 3	106 (0.5)	213 (1.0)	799 (3.8)	1,097 (5.3)	810 (3.9)	64.6 (19.2)	0.85 (0.12)	71.5 (16.5)
	**Respiratory failure**	COPD (n=3,407)	Level 1	688 (20.2)	1,819 (**53.4**)	830 (24.4)	980 (28.8)	1,267 (37.2)			
			Level 2	2,583 (**75.8**)	1,334 (39.2)	1,724 (**50.6**)	1,941 (**57.0**)	1,830 (**53.7**)			
			Level 3	136 (4.0)	254 (7.5)	853 (25.0)	486 (14.3)	310 (9.1)	47.8 (21.6)	0.72 (0.13)	53.9 (17.4)
		Lung fibrosis (n=892)	Level 1	206 (23.1)	536 (**60.1**)	257 (28.8)	298 (33.4)	405 (45.4)			
			Level 2	655 (**73.4**)	300 (33.6)	413 (**46.3**)	484 (**54.3**)	427 (**47.9**)			
			Level 3	31 (3.5)	56 (6.3)	222 (24.9)	110 (12.3)	60 (6.7)	48.1 (22.0)	0.74 (0.14)	56.6 (17.7)
	**Psoriasis**	Psoriasis vulgaris (n=3,943)	Level 1	3,242 (**82.2**)	3,774 (**95.7**)	3,359 (**85.2**)	1,492 (37.8)	2,000 (**50.7**)			
			Level 2	698 (17.7)	148 (3.8)	522 (13.2)	2,140 (**54.3**)	1,682 (42.7)			
			Level 3	3 (0.1)	21 (0.5)	62 (1.6)	311 (7.9)	261(6.6)	-	0.87 (0.11)	73.3 (15.7)
		Psoriasis vulgaris + anthropathic (n=1,128)	Level 1	667 (**59.1**)	1,018 (**90.2**)	773 (**68.5**)	173 (15.3)	526 (**46.6**)			
			Level 2	459 (40.7)	101 (9.0)	321 (28.5)	780 (**69.1**)	515 (45.7)			
			Level 3	2 (0.2)	9 (0.8)	34 (3.0)	175 (15.5)	87 (7.7)	-	0.82 (0.14)	65.7 (18.2)
		Psoriasis unspecified (n=1,090)	Level 1	810 (**74.3**)	1,017 (**93.3**)	842 (**77.2**)	345 (31.7)	539 (**49.4**)			
			Level 2	278 (25.5)	64 (5.9)	216 (19.8)	627 (**57.5**)	469 (43.0)			
			Level 3	2 (0.2)	9 (0.8)	32 (2.9)	118 (10.8)	82 (7.5)	-	0.85 (0.13)	70.3 (17.8)
	**Rheumatology**	Rheumatoid arthritis (n=29,015)	Level 1	14,345 (**49.4**)	22,963 (**79.1**)	17,249 (**59.4**)	3,709 (12.8)	16,449 (**56.7**)			
			Level 2	14,588 (50.3)	5,674 (19.6)	10,483 (36.1)	21,165 (**72.9**)	11,327 (39.0)			
			Level 3	82 (0.3)	378 (1.3)	1,283 (4.4)	4,141 (14.3)	1,239 (4.3)	-	0.81 (0.13)	65.1 (17.7)
		Spondyloarthropathy (n=15,175)	Level 1	7,892 (**52.0**)	12,697 (**83.7**)	8,672 (**57.1**)	1,622 (10.7)	7,504 (**49.4**)			
			Level 2	7,257 (47.8)	2,382 (15.7)	5,735 (37.8)	10,680 (**70.4**)	6,645 (43.8)			
			Level 3	26 (0.2)	96 (0.6)	768 (5.1)	2,873 (18.9)	1,026 (6.8)	-	0.80 (0.14)	63.1 (18.9)
		Inflammatory systemic disorder (n=2,733)	Level 1	1,597 (**58.4**)	2,308 (**84.4**)	1,635 (**59.8**)	524 (19.2)	1,361 (**49.8**)			
			Level 2	1,121 (41.0)	392 (14.3)	924 (33.8)	1,850 (**67.7**)	1,218 (44.6)			
			Level 3	15 (0.5)	33 (1.2)	174 (6.4)	359 (13.1)	154 (5.6)	-	0.81 (0.14)	65.6 (18.3)
		Juvenile rheumatoid diseases (n=1,872)	Level 1	982 (**52.5**)	1,513 (**80.8**)	1,067 (**57.0**)	287 (15.3)	961 (**51.3**)			
			Level 2	880 (47.0)	324 (17.3)	715 (38.2)	1,281 (**68.4**)	814 (43.5)			
			Level 3	10 (0.5)	35 (1.9)	90 (4.8)	304 (16.2)	97 (5.2)	-	0.80 (0.14)	64.3 (18.6)
		Vasculitis (n=754)	Level 1	456 (**60.5**)	675 (**89.5**)	503 (**66.7**)	179 (23.7)	402 (**53.3**)			
			Level 2	297 (39.4)	68 (9.0)	219 (29.0)	508 (**67.4**)	326 (43.2)			
			Level 3	1 (0.1)	11 (1.5)	32 (4.2)	67 (8.9)	26 (3.4)	-	0.84 (0.12)	68.4 (19.7)
		Other arthritis (n=2,996)	Level 1	1,498 (**50.0**)	2,497 (**83.3**)	1,752 (**58.5**)	322 (10.7)	1,637 (**54.6**)			
			Level 2	1,487 (49.6)	481 (16.1)	1,111 (37.1)	2,245 (**74.9**)	1,198 (40.0)			
			Level 3	11 (0.4)	18 (0.6)	133 (4.4)	429 (14.3)	161 (5.4)	-	0.81 (0.13)	64.5 (17.5)
**General population data** (n=49,169)			Level 1	44,279 (**90.1**)	48,371 (**98.4**)	44,848 (**91.2**)	24,946 (**50.8**)	32,721 (**66.5**)			
			Level 2	4,840 (9.8)	600 (1.2)	3,785 (7.7)	22,185 (45.1)	15,126 (30.8)			
			Level 3	50 (0.1)	198 (0.4)	536 (1.1)	2,038 (4.1)	1,322 (2.7)	79.5 (18.3)	0.91 (0.09)	78.6 (12.9)

*EQ VAS calculated based on a subset of the data set with compete data on EQ VAS; Proportions rounded off to one decimal using MS Excel
** The value sets used to calculate EQ-5D index are based on the EQ-5D-5L TTO and VAS value sets (Burström K, Sun S, Gerdtham U-G, *et al.* Swedish experience-based value sets for EQ-5D health states. *Qual Life Res* 2014;**23**:431–42.)

Table S7: Prevalence of reported problems on EQ-5D-3L by diagnosis and mean EQ VAS, and EQ-5D indices (TTO, VAS) among patients with complete data at 1-year follow-up regardless of baseline status and in the general population

	Patient group in NQRs	Diagnosis	Severity level	Mobility	Self-care	Usual activities	Pain/ discomfort	Anxiety/ depression	Mean (SD)		
				n (%)	n (%)	n (%)	n (%)	n (%)	EQ VAS*	EQ-5D index (TTO)**	EQ-5D index (VAS)**
Intervention-based registers	**Spine**	Disc hernia (n=16,196)	Level 1	11,627 (**71.8**)	14,915 (**92.1**)	11,399 (**70.4**)	4,476 (27.6)	10,272 (**63.4**)			
			Level 2	4,514 (27.9)	1189 (7.3)	4223 (26.1)	10,091 (**62.3**)	5,137 (31.7)			
			Level 3	55 (0.3)	92 (0.6)	574 (3.5)	1,629 (10.1)	787 (4.9)	71.5 (21.5)	0.85 (0.13)	71.0 (18.3)
		Other spinal conditions (n=1,828)	Level 1	1,004 (**54.9**)	1,641 (**89.8**)	1,119 (**61.2**)	392 (21.4)	1,087 (**59.5**)			
			Level 2	816 (44.6)	172 (9.4)	599 (32.8)	1,114 (**60.9**)	639 (35.0)			
			Level 3	8 (0.4)	15 (0.8)	110 (6.0)	322 (17.6)	102 (5.6)	66.0 (23.3)	0.82 (0.14)	66.0 (19.7)
		Segmental instability (n=4,670)	Level 1	3,323 (**71.2**)	4,222 (**90.4**)	2,845 (**60.9**)	1,049 (22.5)	2,734 (**58.5**)			
			Level 2	1,331 (28.5)	429 (9.2)	1,505 (32.2)	2,889 (**61.9**)	1,666 (35.7)			
			Level 3	16 (0.3)	19 (0.4)	320 (6.9)	732 (15.7)	270 (5.8)	68.1 (23.3)	0.83 (0.14)	67.8 (19.7)
		Spinal stenosis (n=32,840)	Level 1	14,943 (45.5)	29,720 (**90.5**)	20,679 (**63.0**)	6,529 (19.9)	20,126 (**61.3**)			
			Level 2	17,793 (**54.2**)	2,845 (8.7)	10,533 (32.1)	21,040 (**64.1**)	11,360 (34.6)			
			Level 3	104 (0.3)	275 (0.8)	1,628 (5.0)	5,271 (16.1)	1,354 (4.1)	65.3 (22.8)	0.82 (0.14)	66.0 (18.8)
		Spondylolysis (n=2,763)	Level 1	1,851(**67.0**)	2,558 (**92.6**)	1,783 (**64.5**)	640 (23.2)	1,690 (**61.2**)			
			Level 2	909 (32.9)	197 (7.1)	852 (30.8)	1,740 (**63.0**)	921 (33.3)			
			Level 3	3 (0.1)	8 (0.3)	128 (4.6)	383 (13.9)	152 (5.5)	68.9 (22.5)	0.84 (0.14)	68.6 (19.1)
	**Hip**	Avascular necrosis (n=2,149)	Level 1	1,069 (49.7)	1,811 (**84.3**)	1,394 (**64.9**)	789 (36.7)	1,415 (**65.8**)			
			Level 2	1,071 (**49.8**)	303 (14.1)	621 (28.9)	1,211 (**56.4**)	676 (31.5)			
			Level 3	9 (0.4)	35 (1.6)	134 (6.2)	149 (6.9)	58 (2.7)	71.0 (22.7)	0.84 (0.13)	69.5 (18.0)
		Childhood hip disorder (n=2,236)	Level 1	1,522 (**68.1**)	2,021 (**90.4**)	1,726 (**77.2**)	1,174 (**52.5**)	1,742 (**77.9**)			
			Level 2	712 (31.8)	198 (8.9)	442 (19.8)	965 (43.2)	444 (19.9)			
			Level 3	2 (0.1)	17 (0.8)	68 (3.0)	97 (4.3)	50 (2.2)	78.9 (19.5)	0.88 (0.12)	75.9 (16.5)
		Hip osteoarthritis (n=101,136)	Level 1	61,928 (**61.2**)	93,392 (**92.3**)	78,639 (**77.8**)	45,889 (45.4)	79,190 (**78.3**)			
			Level 2	39,045 (38.6)	7,113 (7.0)	20,384 (20.2)	50,826 (**50.3**)	20,409 (20.2)			
			Level 3	163 (0.2)	631 (0.6)	2,113 (2.1)	4,421 (4.4)	1,537 (1.5)	76.5 (20.1)	0.88 (0.11)	75.1 (15.6)
		Inflammatory joint disorder (n=1,540)	Level 1	666 (43.2)	1,157 (**75.1**)	866 (**56.2**)	440 (28.6)	1,035 (**67.2**)			
			Level 2	865 (**56.2**)	335 (21.8)	583 (37.9)	956 (**62.1**)	468 (30.4)			
			Level 3	9 (0.6)	48 (3.1)	91 (5.9)	144 (9.4)	37 (2.4)	67.8 (22.1)	0.82 (0.13)	67.1 (17.8)
	**Knee**	Knee osteoarthritis (n=18,231)	Level 1	11,460 (**62.9**)	17,338 (**95.1**)	14,143 (**77.6**)	6,610 (36.3)	14,276 (**78.3**)			
			Level 2	6,740 (37.0)	788 (4.3)	3,768 (20.7)	10,668 (**58.5**)	3,639 (20.0)			
			Level 3	31 (0.2)	105 (0.6)	320 (1.8)	953 (5.2)	316 (1.7)	76.1 (19.5)	0.88 (0.11)	74.5 (15.4)
		Knee rheumatoid arthritis (n=372)	Level 1	140 (37.6)	303 (**81.5**)	208 (**55.9**)	79 (21.2)	245 (**65.9**)			
			Level 2	230 (**61.8**)	60 (16.1)	146 (39.2)	254 (**68.3**)	118 (31.7)			
			Level 3	2 (0.5)	9 (2.4)	18 (4.8)	39 (10.5)	9 (2.4)	67.1 (21.0)	0.81 (0.13)	65.8 (17.4)

For peer review only - http://bmjopen.bmj.com/site/about/guidelines.xhtml

Intervention-based registers	**Ankle**	Other (n=152)	Level 1	41 (27.0)	130 (**85.5**)	82 (**53.9**)	37 (24.3)	92 (**60.5**)			
			Level 2	108 (**71.1**)	21 (13.8)	64 (42.1)	98 (**64.5**)	55 (36.2)			
			Level 3	3 (2.0)	1 (0.7)	6 (3.9)	17 (11.2)	5 (3.3)	65.0 (21.6)	0.80 (0.13)	64.1 (17.9)
		Posttraumatic osteoarthritis (n=510)	Level 1	178 (34.9)	472 (**92.5**)	314 (**61.6**)	120 (23.5)	338 (**66.3**)			
			Level 2	329 (**64.5**)	34 (6.7)	165 (32.4)	328 (**64.3**)	140 (27.5)			
			Level 3	3 (0.6)	4 (0.8)	31 (6.1)	62 (12.2)	32 (6.3)	68.6 (21.3)	0.81 (0.14)	65.8 (19.1)
		Primary osteoarthritis (n=388)	Level 1	166 (42.8)	364 (**93.8**)	277 (**71.4**)	121 (31.2)	302 (**77.8**)			
			Level 2	221 (**57.0**)	22 (5.7)	95 (24.5)	222 (**57.2**)	80 (20.6)			
			Level 3	1 (0.3)	2 (0.5)	16 (4.1)	45 (11.6)	6 (1.5)	72.4 (18.6)	0.85 (0.12)	70.3 (17.6)
		Rheumatoid arthritis (n=117)	Level 1	32 (27.4)	94 (**80.3**)	60 (**51.3**)	25 (21.4)	73 (**62.4**)			
			Level 2	83 (**70.9**)	21 (17.9)	51 (43.6)	78 (**66.7**)	40 (34.2)			
			Level 3	2 (1.7)	2 (1.7)	6 (5.1)	14 (12.0)	4 (3.4)	64.3 (19.9)	0.80 (0.13)	63.7 (17.4)
	**Cruciate ligament**	Cruciate ligament injury (n=15,731)	Level 1	13,492 (**85.8**)	15,534 (**98.7**)	12,269 (**78.0**)	5,356 (34.0)	10,105 (**64.2**)			
			Level 2	2,230 (14.2)	142 (0.9)	3,207 (20.4)	9,812 (**62.4**)	5,085 (32.3)			
			Level 3	9 (0.1)	55 (0.3)	255 (1.6)	563 (3.6)	541 (3.4)	74.2 (20.1)	0.88 (0.11)	75.2 (15.0)
	**BOA**	BOA hip (n=3,437)	Level 1	1,726 (**50.2**)	3,212 (**93.5**)	2,659 (**77.4**)	258 (7.5)	2,301 (**66.9**)			
			Level 2	1,708 (49.7)	214 (6.2)	742 (21.6)	2,887 (**84.0**)	1,093 (31.8)			
			Level 3	3 (0.1)	11 (0.3)	36 (1.0)	292 (8.5)	43 (1.3)	68.0 (18.8)	0.85 (0.10)	69.8 (14.4)
		BOA knee (n=10,248)	Level 1	5,418 (**52.9**)	9,926 (**96.9**)	8,258 (**80.6**)	1,015 (9.9)	7,297 (**71.2**)			
			Level 2	4,824 (47.1)	272 (2.7)	1,906 (18.6)	8,437 (**82.3**)	2,830 (27.6)			
			Level 3	6 (0.1)	50 (0.5)	84 (0.8)	796 (7.8)	121 (1.2)	70.7 (18.6)	0.86 (0.10)	71.2 (14.0)
	**Fracture**	Ankle and foot (n=3,898)	Level 1	2,832 (**72.7**)	3,740 (**95.9**)	3,248 (**83.3**)	1,917 (**49.2**)	3,139 (**80.5**)			
			Level 2	1,055 (27.1)	124 (3.2)	569 (14.6)	1,820 (46.7)	684 (17.5)			
			Level 3	11 (0.3)	34 (0.9)	81 (2.1)	161 (4.1)	75 (1.9)	81.0 (19.0)	0.90 (0.11)	77.3 (15.3)
		Elbow and forearm (n=14,576)	Level 1	12,585 (**86.3**)	13,594 (**93.3**)	12,045 (**82.6**)	6,886 (47.2)	11,816 (**81.1**)			
			Level 2	1,944 (13.3)	828 (5.7)	2,155 (14.8)	7,270 (**49.9**)	2,547 (17.5)			
			Level 3	47 (0.3)	154 (1.1)	376 (2.6)	420 (2.9)	213 (1.5)	81.3 (18.5)	0.90 (0.10)	78.7 (13.6)
		Hip and femur (n=8,858)	Level 1	2,521 (28.5)	5,926 (**66.9**)	4,424 (**49.9**)	2,403 (27.1)	5,072 (**57.3**)			
			Level 2	5,914 (**66.8**)	2,027 (22.9)	2,791 (31.5)	5,770 (**65.1**)	3,407 (38.5)			
			Level 3	423 (4.8)	905 (10.2)	1,643 (18.5)	685 (7.7)	379 (4.3)	64.0 (23.5)	0.78 (0.14)	62.2 (18.8)
		Knee and lower leg (n=11,360)	Level 1	7,279 (**64.1**)	10,644 (**93.7**)	8,896 (**78.3**)	4,384 (38.6)	8,690 (**76.5**)			
			Level 2	3,981 (35.0)	527 (4.6)	2,055 (18.1)	6,507 (**57.3**)	2,424 (21.3)			
			Level 3	100 (0.9)	189 (1.7)	409 (3.6)	469 (4.1)	246 (2.2)	78.2 (19.4)	0.88 (0.12)	74.5 (16.1)
		Lumbar, spine and pelvis (n=1,232)	Level 1	583 (47.3)	945 (**76.7**)	731 (**59.3**)	434 (35.2)	794 (**64.4**)			
			Level 2	626 (**50.8**)	208 (16.9)	332 (26.9)	696 (**56.5**)	390 (31.7)			
			Level 3	23 (1.9)	79 (6.4)	169 (13.7)	102 (8.3)	48 (3.9)	68.7 (23.4)	0.82 (0.15)	67.1 (19.7)
		Shoulder and upper arm (n=8,273)	Level 1	6,566 (**79.4**)	7,041 (**85.1**)	6,141 (**74.2**)	3,055 (36.9)	6,162 (**74.5**)			
			Level 2	1,652 (20.0)	1,035 (12.5)	1,727 (20.9)	4,834 (**58.4**)	1,925 (23.3)			
			Level 3	55 (0.7)	197 (2.4)	405 (4.9)	384 (4.6)	186 (2.2)	77.0 (20.3)	0.88 (0.12)	74.9 (15.9)
		Unspecified part of trunk, extremities or other body region (n=860)	Level 1	513 (**59.7**)	719 (**83.6**)	526 (**61.2**)	190 (22.1)	552 (**64.2**)			
			Level 2	329 (38.3)	111 (12.9)	248 (28.8)	565 (**65.7**)	270 (31.4)			
			Level 3	18 (2.1)	30 (3.5)	86 (10.0)	105 (12.2)	38 (4.4)	69.9 (22.0)	0.83 (0.15)	67.6 (19.6)
		Wrist and hand (n=4,532)	Level 1	4,069 (**89.8**)	4,301 (**94.9**)	3,775 (**83.3**)	2,171 (47.9)	3,745 (82.6)			
			Level 2	445 (9.8)	189 (4.2)	667 (14.7)	2,225 (49.1)	722 (15.9)			
			Level 3	18 (0.4)	42 (0.9)	90 (2.0)	136 (3.0)	65 (1.4)	82.9 (17.7)	0.91 (0.09)	79.4 (13.2)

For peer review only - http://bmjopen.bmj.com/site/about/guidelines.xhtml

Diagnosis-based registers	**Heart failure**	Heart failure (n=1,849)	Level 1	1,094 (**59.2**)	1,680 (**90.9**)	1,338 (**72.4**)	911 (**49.3**)	1,157 (**62.6**)			
			Level 2	748 (40.5)	153 (8.3)	458 (24.8)	819 (44.3)	645 (34.9)			
			Level 3	7 (0.4)	16 (0.9)	53 (2.9)	119 (6.4)	47 (2.5)	66.7 (19.5)	0.86 (0.12)	72.3 (16.3)
	**Respiratory failure**	COPD (n=895)	Level 1	160 (17.9)	472 (**52.7**)	175 (19.6)	208 (23.2)	315 (35.2)			
			Level 2	693 (**77.4**)	344 (38.4)	460 (**51.4**)	541 (**60.4**)	488 (**54.5**)			
			Level 3	42 (4.7)	79 (8.8)	260 (29.1)	146 (16.3)	92 (10.3)	48.1 (20.4)	0.70 (0.14)	51.7 (17.5)
		Lung fibrosis (n=149)	Level 1	25 (16.8)	72 (**48.3**)	32 (21.5)	43 (28.9)	68 (**45.6**)			
			Level 2	118 (**79.2**)	66 (44.3)	61 (**40.9**)	86 (**57.7**)	68 (**45.6**)			
			Level 3	6 (4.0)	11 (7.4)	56 (37.6)	20 (13.4)	13 (8.7)	48.5 (19.7)	0.71 (0.14)	53.1 (18.2)
	**Psoriasis**	Psoriasis vulgaris (n=1,880)	Level 1	1,612 (**85.7**)	1,829 (**97.3**)	1,687 (**89.7**)	1,004 (**53.4**)	1,236 (**65.7**)			
			Level 2	267 (14.2)	48 (2.6)	172 (9.1)	806 (42.9)	573 (30.5)			
			Level 3	1 (0.1)	3 (0.2)	21 (1.1)	70 (3.7)	71 (3.8)	-	0.90 (0.10)	78.0 (13.9)
		Psoriasis vulgaris + anthropathic (n=663)	Level 1	406 (**64.3**)	584 (**92.6**)	497 (**78.8**)	161 (25.5)	358 (**56.7**)			
			Level 2	223 (35.3)	41 (6.5)	118 (18.7)	405 (**64.2**)	236 (37.4)			
			Level 3	2 (0.3)	6 (1.0)	16 (2.5)	65 (10.3)	37 (5.9)	-	0.85 (0.12)	70.3 (17.1)
		Psoriasis unspecified (n=363)	Level 1	283 (**78.0**)	351 (**96.7**)	316 (**87.1**)	160 (44.1)	244 (**67.2**)			
			Level 2	80 (22.0)	11 (3.0)	45 (12.4)	183 (**50.4**)	104 (28.7)			
			Level 3	1,612 (0.0)	1 (0.3)	2 (0.6)	20 (5.5)	15 (4.1)	-	0.89 (0.11)	76.1 (14.9)
	**Rheumatology**	Rheumatoid arthritis (n=18,141)	Level 1	9,904 (54.6)	15,143 (83.5)	12,032 (66.3)	3,199 (17.6)	11,123 (61.3)			
			Level 2	8,165 (45.0)	2,763 (15.2)	5,506 (30.4)	12,974 (71.5)	6,294 (34.7)			
			Level 3	72 (0.4)	235 (1.3)	603 (3.3)	1,968 (10.8)	724 (4.0)	-	0.83 (0.13)	67.9 (17.4)
		Spondyloarthropathy (n=8,916)	Level 1	5,142 (**57.7**)	7,578 (**85.0**)	5,582 (**62.6**)	1,293 (14.5)	4,813 (**54.0**)			
			Level 2	3,757 (42.1)	1,270 (14.2)	2,973 (33.3)	6,277 (**70.4**)	3,533 (39.6)			
			Level 3	17 (0.2)	68 (0.8)	361 (4.0)	1,346 (15.1)	570 (6.4)	-	0.82 (0.14)	65.7 (18.8)
		Inflammatory systemic disorder (n1,654)	Level 1	984 (**59.5**)	1,425 (**86.2**)	1,029 (**62.2**)	350 (21.2)	849 (**51.3**)			
			Level 2	664 (40.1)	214 (12.9)	540 (32.6)	1,109 (**67.0**)	705 (42.6)			
			Level 3	6 (0.4)	15 (0.9)	85 (5.1)	195 (11.8)	100 (6.0)	-	0.82 (0.13)	66.4 (18.2)
		Juvenile rheumatoid diseases (n=1,112)	Level 1	605 (**54.4**)	894 (**80.4**)	670 (**60.3**)	204 (18.3)	580 (**52.2**)			
			Level 2	502 (45.1)	202 (18.2)	387 (34.8)	747 (**67.2**)	470 (42.3)			
			Level 3	5 (0.4)	16 (1.4)	55 (4.9)	161 (14.5)	62 (5.6)	-	0.81 (0.14)	65.3 (18.9)
		Vasculitis (n=413)	Level 1	242 (**58.6**)	375 (**90.8**)	278 (**67.3**)	92 (22.3)	242 (**58.6**)			
			Level 2	170 (41.2)	31 (7.5)	125 (30.3)	281 (**68.0**)	156 (37.8)			
			Level 3	1 (0.2)	7 (1.7)	10 (2.4)	40 (9.7)	15 (3.6)	-	0.84 (0.12)	68.7 (16.6)
		Other arthritis (n=1,728)	Level 1	1,004 (**58.1**)	1,521 (**88.0**)	1,185 (**68.6**)	303 (17.5)	1,066 (**61.7**)			
			Level 2	721 (41.7)	193 (11.2)	493 (28.5)	1,212 (**70.1**)	595 (34.4)			
			Level 3	3 (0.2)	14 (0.8)	50 (2.9)	213 (12.3)	67 (3.9)	-	0.84 (0.13)	68.4 (17.4)
**General population data** (n=49,169)			Level 1	44,279 (**90.1**)	48,371 (**98.4**)	44,848 (**91.2**)	24,946 (**50.8**)	32,721 (**66.5**)			
			Level 2	4,840 (9.8)	600 (1.2)	3,785 (7.7)	22,185 (45.1)	15,126 (30.8)			
			Level 3	50 (0.1)	198 (0.4)	536 (1.1)	2,038 (4.1)	1,322 (2.7)	79.5 (18.3)	0.91 (0.09)	78.6 (12.9)

*EQ VAS calculated based on a subset of the data set with compete data on EQ VAS; Proportions rounded off to one decimal using MS Excel

** The value sets used to calculate EQ-5D index are based on the EQ-5D-5L TTO and VAS value sets (Burström K, Sun S, Gerdtham U-G, *et al.* Swedish experience-based value sets for EQ-5D health states. *Qual Life Res* 2014;**23**:431–42.)

For peer review only - http://bmjopen.bmj.com/site/about/guidelines.xhtml

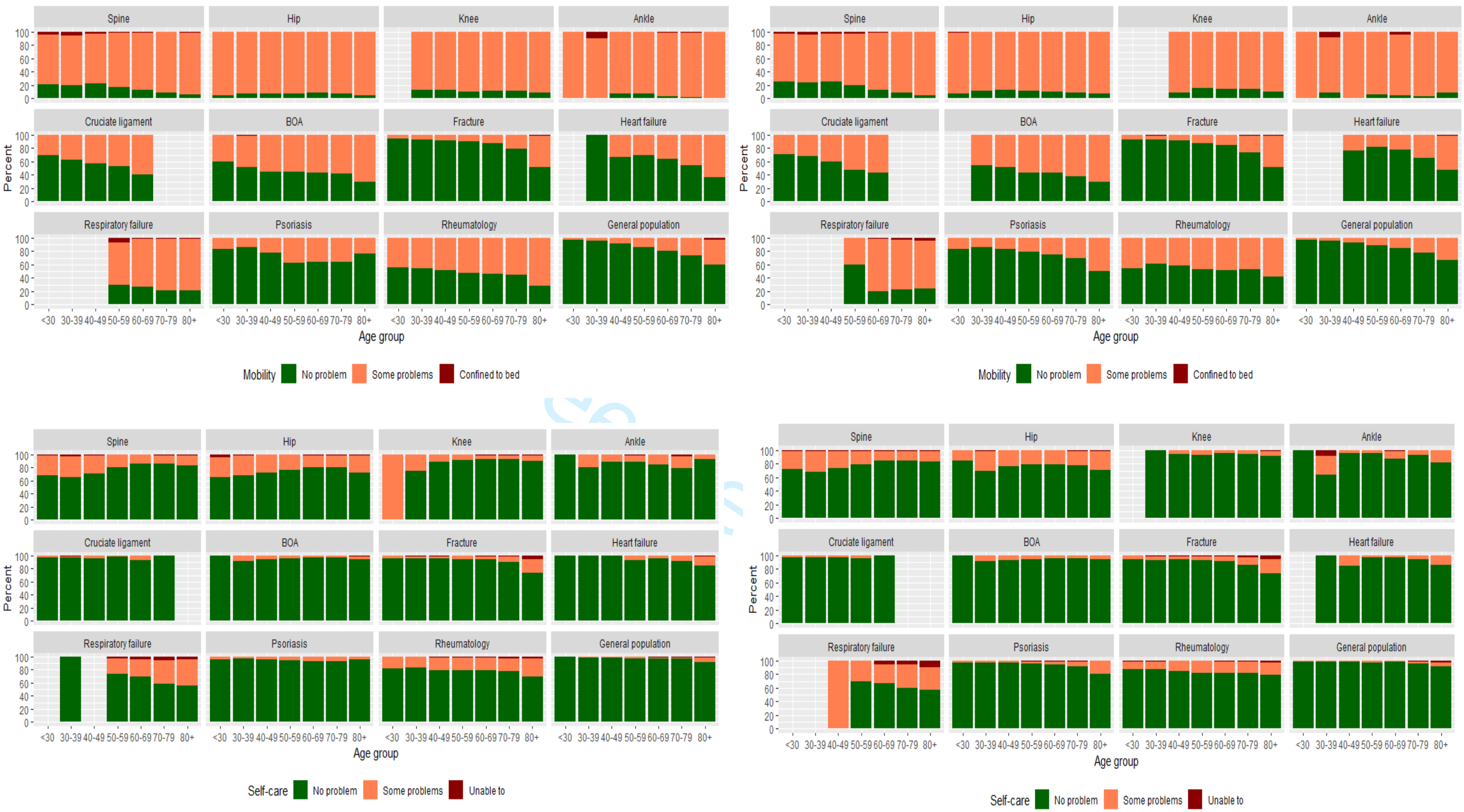
Spine
Hip
Knee
Ankle
Cruciate ligament
BOA
Fracture
Heart failure
Respiratory failure
Psoriasis
Rheumatology
General population
Percent
Age group
<30
30-39
40-49
50-59
60-69
70-79
80+
Mobility
No problem
Some problems
Confined to bed
Self-care
No problem
Some problems
Unable to

Women

Men

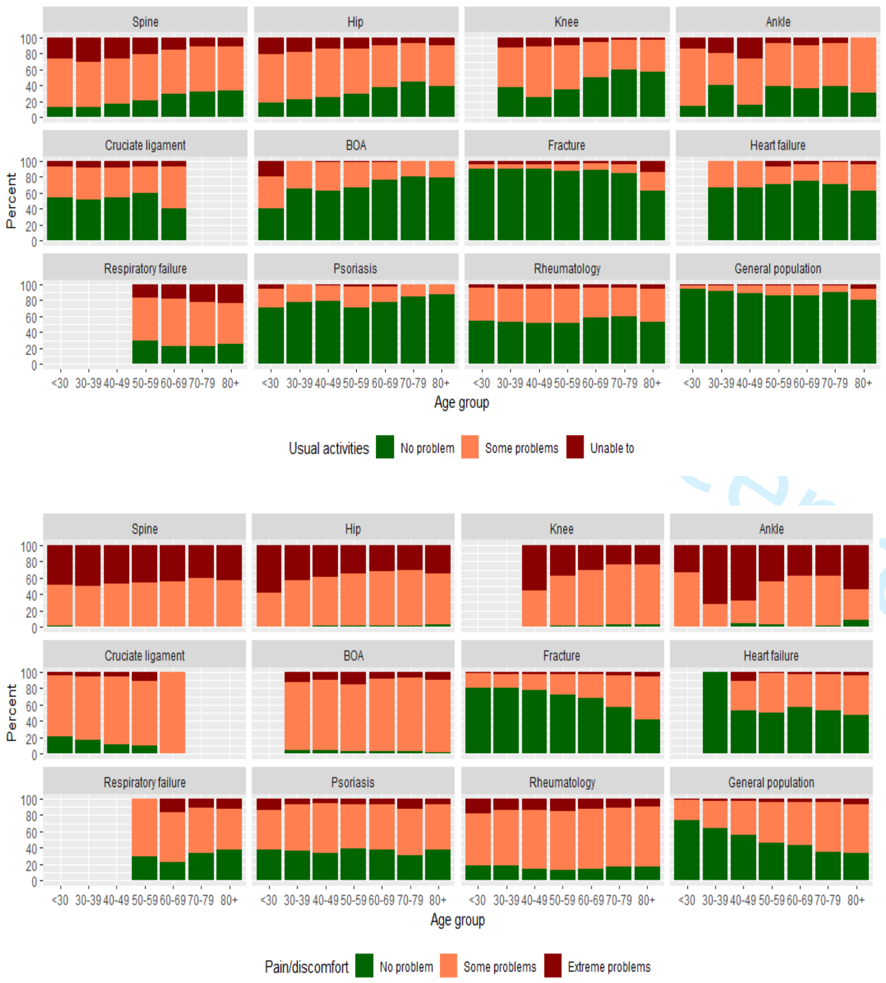

Women

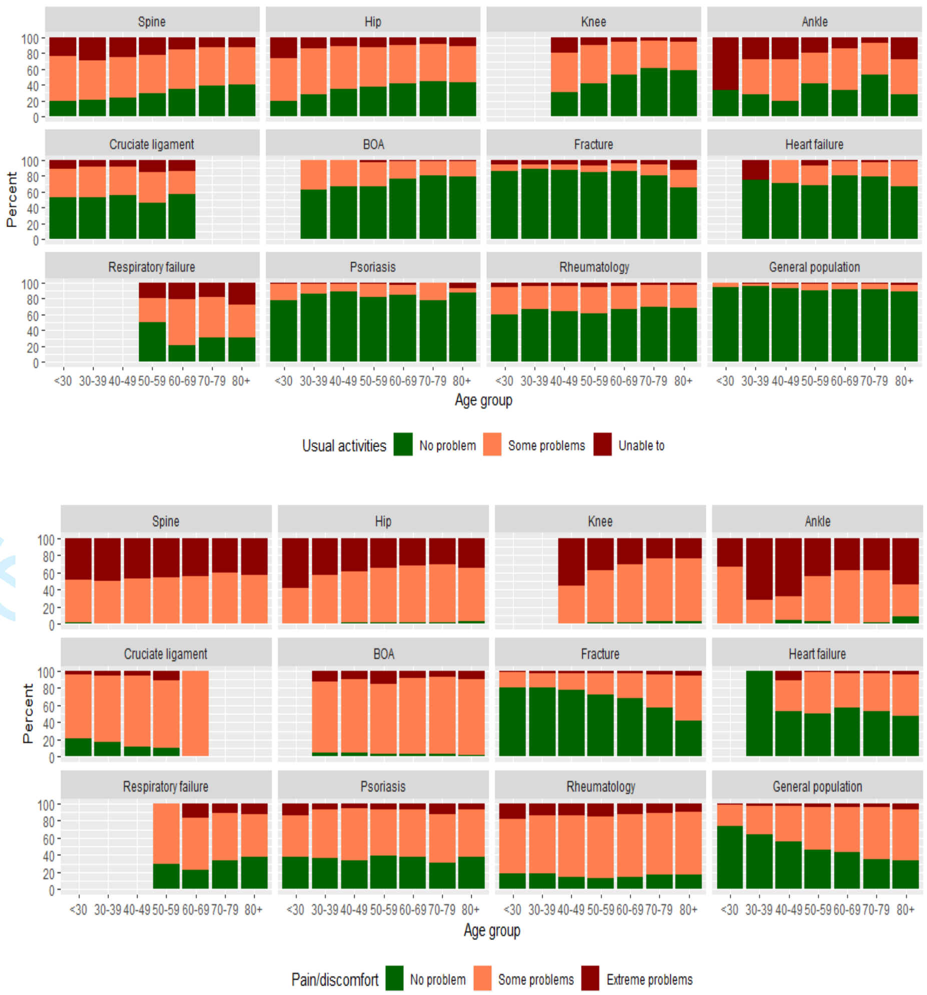

Men

For peer review only - http://bmjopen.bmj.com/site/about/guidelines.xhtml

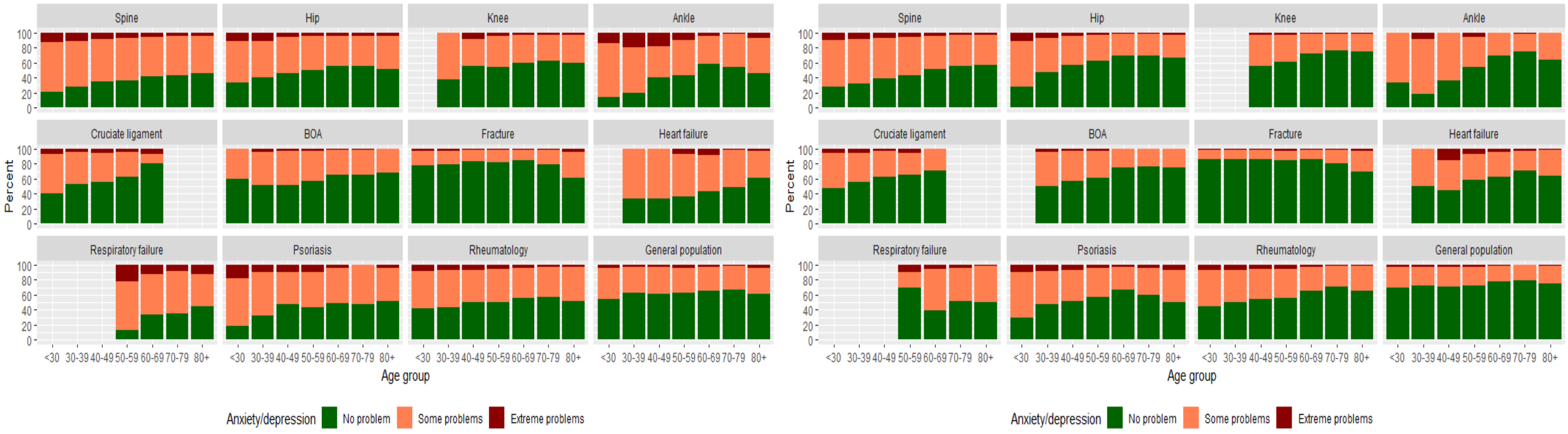

Women Men

Figure S1: Problems reported on the EQ-5D-3L dimensions among patients in the different registers at baseline and the general population by age group and sex

For peer review only - http://bmjopen.bmj.com/site/about/guidelines.xhtml

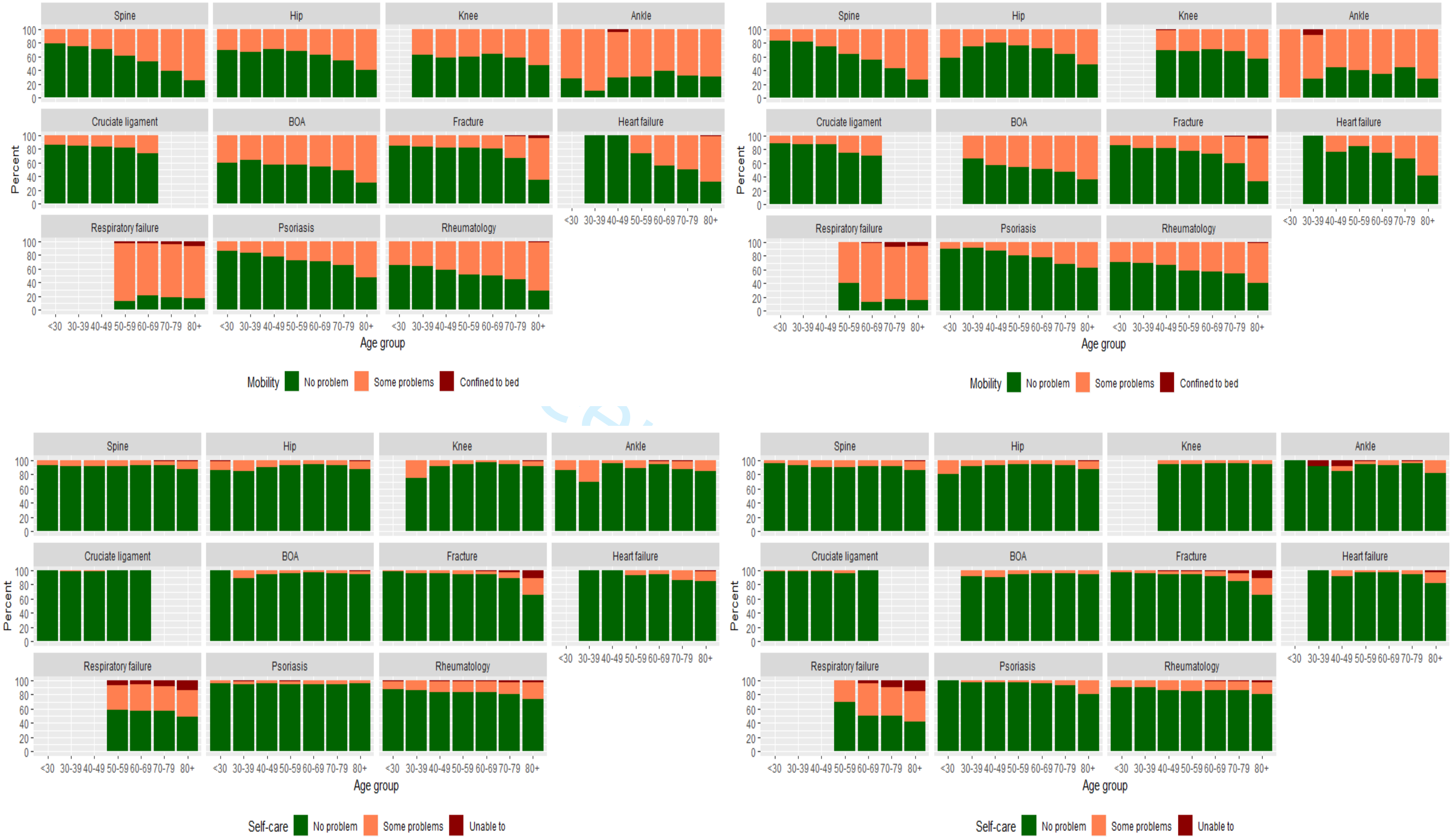
Spine
Hip
Knee
Ankle
Cruciate ligament
BOA
Fracture
Heart failure
Respiratory failure
Psoriasis
Rheumatology
Percent
<30
30-39
40-49
50-59
60-69
70-79
80+
Age group
Mobility
No problem
Some problems
Confined to bed
Self-care
No problem
Some problems
Unable to

Women

Men

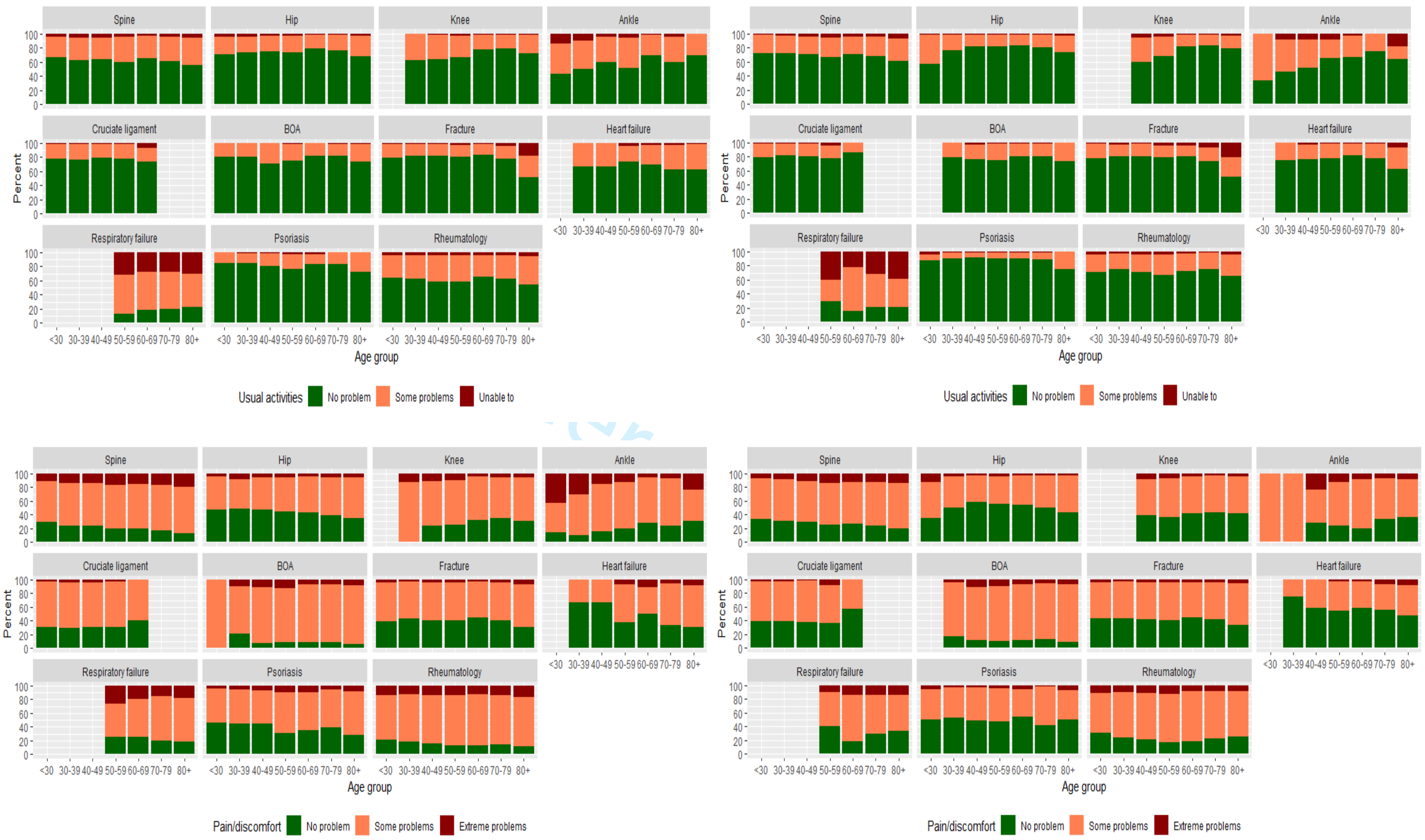
Spine
Hip
Knee
Ankle
Cruciate ligament
BOA
Fracture
Heart failure
Respiratory failure
Psoriasis
Rheumatology
Percent
100
80
60
40
20
0
<30
30-39
40-49
50-59
60-69
70-79
80+
Age group
Usual activities
No problem
Some problems
Unable to
Pain/discomfort
No problem
Some problems
Extreme problems

Women Men

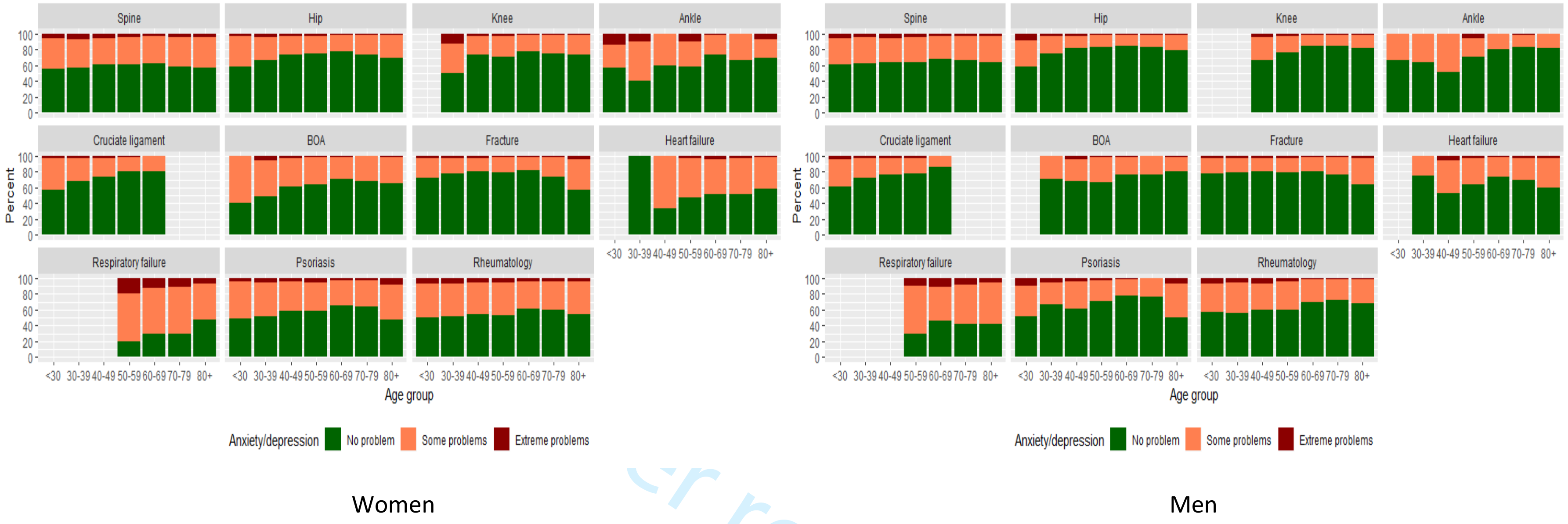

Figure S2: Problems reported on the EQ-5D-3L dimensions among patients in the different registers at 1-year follow-up by age group and sex

For peer review only - http://bmjopen.bmj.com/site/about/guidelines.xhtml

The RECORD statement – checklist of items, extended from the STROBE statement, that should be reported in observational studies using routinely collected health data.

	Item No.	STROBE items	Location in manuscript where items are reported	RECORD items	Location in manuscript where items are reported
**Title and abstract**					
	1	(a) Indicate the study's design with a commonly used term in the title or the abstract (b) Provide in the abstract an informative and balanced summary of what was done and what was found	1-3	RECORD 1.1: The type of data used should be specified in the title or abstract. When possible, the name of the databases used should be included.<br><br>RECORD 1.2: If applicable, the geographic region and timeframe within which the study took place should be reported in the title or abstract.<br><br>RECORD 1.3: If linkage between databases was conducted for the study, this should be clearly stated in the title or abstract.	1-3<br><br>1
**Introduction**					
Background rationale	2	Explain the scientific background and rationale for the investigation being reported	5		
Objectives	3	State specific objectives, including any prespecified hypotheses	6		
**Methods**					
Study Design	4	Present key elements of study design early in the paper	7		
Setting	5	Describe the setting, locations, and relevant dates, including periods of recruitment, exposure, follow-up, and data collection	7		

For peer review only

Participants	6	*(a) Cohort study* - Give the eligibility criteria, and the sources and methods of selection of participants. Describe methods of follow-up<br>*Case-control study* - Give the eligibility criteria, and the sources and methods of case ascertainment and control selection. Give the rationale for the choice of cases and controls<br>*Cross-sectional study* - Give the eligibility criteria, and the sources and methods of selection of participants<br><br>*(b) Cohort study* - For matched studies, give matching criteria and number of exposed and unexposed<br>*Case-control study* - For matched studies, give matching criteria and the number of controls per case	7-8	RECORD 6.1: The methods of study population selection (such as codes or algorithms used to identify subjects) should be listed in detail. If this is not possible, an explanation should be provided.<br><br>RECORD 6.2: Any validation studies of the codes or algorithms used to select the population should be referenced. If validation was conducted for this study and not published elsewhere, detailed methods and results should be provided.<br><br>RECORD 6.3: If the study involved linkage of databases, consider use of a flow diagram or other graphical display to demonstrate the data linkage process, including the number of individuals with linked data at each stage.	7-8
Variables	7	Clearly define all outcomes, exposures, predictors, potential confounders, and effect modifiers. Give diagnostic criteria, if applicable.	8	RECORD 7.1: A complete list of codes and algorithms used to classify exposures, outcomes, confounders, and effect modifiers should be provided. If these cannot be reported, an explanation should be provided.	
Data sources/ measurement	8	For each variable of interest, give sources of data and details of methods of assessment (measurement).<br>Describe comparability of assessment methods if there is more than one group	8		

For peer review only

Bias	9	Describe any efforts to address potential sources of bias	9		
Study size	10	Explain how the study size was arrived at	7-8		
Quantitative variables	11	Explain how quantitative variables were handled in the analyses. If applicable, describe which groupings were chosen, and why	8		
Statistical methods	12	(a) Describe all statistical methods, including those used to control for confounding<br>(b) Describe any methods used to examine subgroups and interactions<br>(c) Explain how missing data were addressed<br>(d) *Cohort study* - If applicable, explain how loss to follow-up was addressed<br>*Case-control study* - If applicable, explain how matching of cases and controls was addressed<br>*Cross-sectional study* - If applicable, describe analytical methods taking account of sampling strategy<br>(e) Describe any sensitivity analyses	8<br>8<br>9<br><br><br><br>9		
Data access and cleaning methods		..		RECORD 12.1: Authors should describe the extent to which the investigators had access to the database population used to create the study population.	

For peer review only

				RECORD 12.2: Authors should provide information on the data cleaning methods used in the study.	7-8
Linkage		..		RECORD 12.3: State whether the study included person-level, institutional-level, or other data linkage across two or more databases. The methods of linkage and methods of linkage quality evaluation should be provided.	
**Results**					
Participants	13	(a) Report the numbers of individuals at each stage of the study (*e.g.*, numbers potentially eligible, examined for eligibility, confirmed eligible, included in the study, completing follow-up, and analysed)<br>(b) Give reasons for non-participation at each stage.<br>(c) Consider use of a flow diagram	7-8	RECORD 13.1: Describe in detail the selection of the persons included in the study (*i.e.*, study population selection) including filtering based on data quality, data availability and linkage. The selection of included persons can be described in the text and/or by means of the study flow diagram.	7-8
Descriptive data	14	(a) Give characteristics of study participants (*e.g.*, demographic, clinical, social) and information on exposures and potential confounders<br>(b) Indicate the number of participants with missing data for each variable of interest<br>(c) *Cohort study* - summarise follow-up time (*e.g.*, average and total amount)	10		
Outcome data	15	*Cohort study* - Report numbers of outcome events or summary measures over time<br>*Case-control study* - Report numbers in each exposure	10		

For peer review only

		category, or summary measures of exposure<br>*Cross-sectional study* - Report numbers of outcome events or summary measures			
Main results	16	(a) Give unadjusted estimates and, if applicable, confounder-adjusted estimates and their precision (e.g., 95% confidence interval). Make clear which confounders were adjusted for and why they were included<br>(b) Report category boundaries when continuous variables were categorized<br>(c) If relevant, consider translating estimates of relative risk into absolute risk for a meaningful time period	10-13		
Other analyses	17	Report other analyses done—e.g., analyses of subgroups and interactions, and sensitivity analyses	Supplementary material		
**Discussion**					
Key results	18	Summarise key results with reference to study objectives	14		
Limitations	19	Discuss limitations of the study, taking into account sources of potential bias or imprecision. Discuss both direction and magnitude of any potential bias	17	RECORD 19.1: Discuss the implications of using data that were not created or collected to answer the specific research question(s). Include discussion of misclassification bias, unmeasured confounding, missing data, and changing eligibility over time, as they pertain to the study being reported.	17-18
Interpretation	20	Give a cautious overall interpretation of results considering objectives,	17-18		

For peer review only - http://bmjopen.bmj.com/site/about/guidelines.xhtml

		limitations, multiplicity of analyses, results from similar studies, and other relevant evidence			
Generalisability	21	Discuss the generalisability (external validity) of the study results	17-19		
**Other Information**					
Funding	22	Give the source of funding and the role of the funders for the present study and, if applicable, for the original study on which the present article is based	19		
Accessibility of protocol, raw data, and programming code		..		RECORD 22.1: Authors should provide information on how to access any supplemental information such as the study protocol, raw data, or programming code.	20

*Reference: Benchimol EI, Smeeth L, Guttmann A, Harron K, Moher D, Petersen I, Sørensen HT, von Elm E, Langan SM, the RECORD Working Committee. The REporting of studies Conducted using Observational Routinely-collected health Data (RECORD) Statement. *PLoS Medicine* 2015; in press.

*Checklist is protected under Creative Commons Attribution (CC BY) license.
